# Development and Evaluation of Benzofuran Oxoacetic Acid Compounds as EPAC1 Activators

**DOI:** 10.1021/acs.jmedchem.5c02974

**Published:** 2026-03-14

**Authors:** David Morgan, Jolanta Wiejak, Frederick G. Powell, Chiara Fitzpatrick, Stephen J. Yarwood, Graeme Barker

**Affiliations:** † Institute of Chemical Sciences, 3120 Heriot-Watt University, Riccarton, Edinburgh EH14 4AS, U.K.; ‡ Institute of Biochemistry, Biophysics and Bioengineering, Heriot-Watt University, Riccarton, Edinburgh EH14 4AS, U.K.

## Abstract

Exchange protein directly activated by cAMP 1 (EPAC1) modulates Rap signaling and fibrosis. We report benzofuran oxoacetic acids as non-nucleotide EPAC1 agonists. Convergent synthesis delivered C2-diversified analogues (overall yields ≈ 3–7%). Fluorescent competition at isolated CNBDs mapped isoform engagement: several analogues favored EPAC1 (e.g., **DM244**, **DM357**, and **DM408**), **DM312** favored EPAC2, and small C2 changes tuned bias. In cells, EPAC1-transfected U2OS assays showed significant Rap1-GTP increases for **DM243**, **DM244,** and **DM245**, with no activation in EPAC2 cells and no detectable protein kinase A activity. In disease-relevant contexts, the series attenuated IL-6/STAT3 signaling in human umbilical vascular endothelial cells and inhibited TGF-β1-induced fibroblast-to-myofibroblast transition (αSMA, Collagen I) with midmicromolar potencies; known drugs, **SB525334** and **nintedanib**, remained more potent, yet nintedanib was markedly more cytotoxic. Across assays, some binding-phenotype disconnects emerged, plausibly reflecting exposure, signaling bias, and cell-context effects. Overall, benzofuran oxoacetic acids provide EPAC-pathway probes with a favorable tolerability window and scope for potency optimization as antifibrotics.

## Graphical abstract

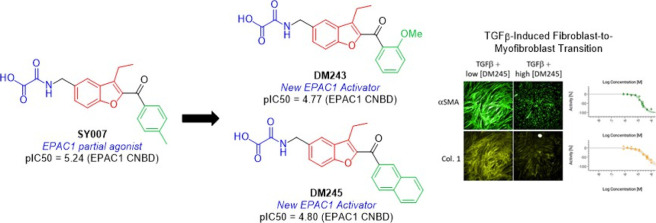

## Introduction

Exchange protein directly activated by cAMP 1 (EPAC1) is a major intracellular effector of the second messenger 3′,5′-cyclic adenosine monophosphate (cAMP) and a guanine nucleotide exchange factor (GEF) for Rap GTPases.^1–3^ By linking cAMP signals to Rap activation, EPAC1 plays a vital role in numerous cellular processes and disease pathways, underpinning its broad therapeutic potential in human pathologies. Notably, EPAC1 activity has been implicated in cardiac function and remodeling, as well as in the regulation of inflammatory and fibrotic responses, highlighting EPAC1 as a promising drug target in conditions ranging from heart disease to chronic inflammation and fibrosis. Its influence on diverse signaling networks and disease processes has made EPAC1 a focal point for drug discovery efforts aiming to modulate cAMP pathways with greater precision than global cAMP-elevating strategies.^1–9^

Like its isoform EPAC2 and the classical cAMP effector protein kinase A (PKA), EPAC1 is activated by cAMP, but strategic ligand design can preferentially activate one target over the others.^10–14^ This isoform-specific targeting is desirable to dissect the complex cAMP signaling hierarchy and to avoid off-target effects from broad cAMP stimulation. A landmark development in this regard was the synthesis of 8-(4-chlorophenylthio)-2′-*O*-methyladenosine-3′,5′-cyclic monophosphate (8-pCPT-2′-*O*-Me-cAMP, also known as D-007).^13,14^ D-007 is a cAMP analogue engineered to selectively activate EPAC proteins while sparing PKA, and it was initially used to determine whether certain cellular responses (e.g., ERK activation) were mediated via cAMP’s PKA pathway or the EPAC–Rap1 pathway.^14^ The design of D-007 exploited a subtle difference in the cyclic nucleotide-binding domain (CNBD) of PKA versus EPAC: a conserved glutamate in PKA’s CNBD forms a critical hydrogen bond with the 2′-hydroxy of cAMP, whereas this position is a glutamine in EPAC1 (and a lysine in EPAC2).^13^ As a result, the 2′-*O*-methyl modification in D-007 disrupts PKA binding but is tolerated by EPAC1 and EPAC2, enabling PKA-sparing activation of EPAC. While D-007 provided a powerful tool for delineating cAMP signaling, it remains a cyclic nucleotide scaffold with limited drug-like properties and does not discriminate between EPAC1 and EPAC2, prompting the search for isoform-selective, noncyclic EPAC activators.^8^

The first breakthrough toward noncyclic EPAC1-selective agonists came from high-throughput screening efforts that yielded I942, an *N*-acylsulfonamide derivative identified as the first noncyclic nucleotide partial agonist of EPAC1.^15^ I942 represented a new class of EPAC1 ligands and a significant milestone in EPAC research, demonstrating that small molecules can directly activate EPAC1 without a cyclic nucleotide structure. Importantly, I942 was found to selectively activate EPAC1 over EPAC2, thereby validating the concept of isoform-specific targeting.^15,16^ Cellular studies confirmed that I942 engages EPAC1 signaling pathways: it promotes EPAC1-mediated Rap1 activation in cells and modulates downstream effects such as upregulating SOCS3 and suppressing interleukin-6 (IL-6)-stimulated JAK/STAT3 signaling in vascular endothelial cells.^17^ These anti-inflammatory actions of I942 underscore the therapeutic relevance of EPAC1 activation in inflammation.^8^ the mechanisms by which I942 induces activation of EPAC1 have been identified using a computational steered molecular dynamics/Markov state modeling approach.^18^ Several analogues of I942 (e.g., **PWO381**, **PWO521**, and **PWO577**) were subsequently developed to explore structure–activity relationships, expanding the toolkit of EPAC1 activators, and **PWO577** demonstrated to promote aortic relaxation in ex vivo mouse models.^19–21^ Nevertheless, I942 and its analogues have inherent limitations: as a partial agonist, I942 cannot fully recapitulate the maximal activation of EPAC1 by cAMP, and its efficacy and pharmacokinetic properties may be suboptimal for therapeutic use. This created a clear rationale for designing new EPAC1 agonists with improved potency, efficacy, and drug-like characteristics.

Considering these challenges, we pursued a novel benzofuran oxoacetic acid scaffold typified by initial hit **SY000** to develop next-generation EPAC1 activators (Figure 1).^22^ The design of these benzofuran oxoacetic acid compounds was guided by the need for isoform selectivity and PKA sparing, as well as the desire to move beyond nucleotide-like structures. We envisioned that a benzofuran heterocycle could mimic the adenine ring of cAMP, while an oxoacetic acid moiety could provide key hydrogen-bonding and electrostatic interactions analogous to cAMP’s ribose-phosphate unit, thus preserving binding affinity for the EPAC1 CNBD.^22^ By incorporating functional groups tailored to exploit EPAC1-specific interactions (for instance, considering the EPAC1–specific Gln residue in the CNBD), we aimed to bias binding toward EPAC1 over EPAC2. This medicinal chemistry strategy was found to yield EPAC1-selective agonists that do not activate EPAC2 or PKA, thereby targeting only the desired branch of the cAMP pathway. An additional motivation for this scaffold was to improve pharmacological properties: small-molecule benzofuran derivatives are generally more stable and cell-permeable than cyclic nucleotides, making them attractive leads for drug development in inflammation and fibrosis, where chronic oral or systemic therapy may be required.

**Figure 1:**
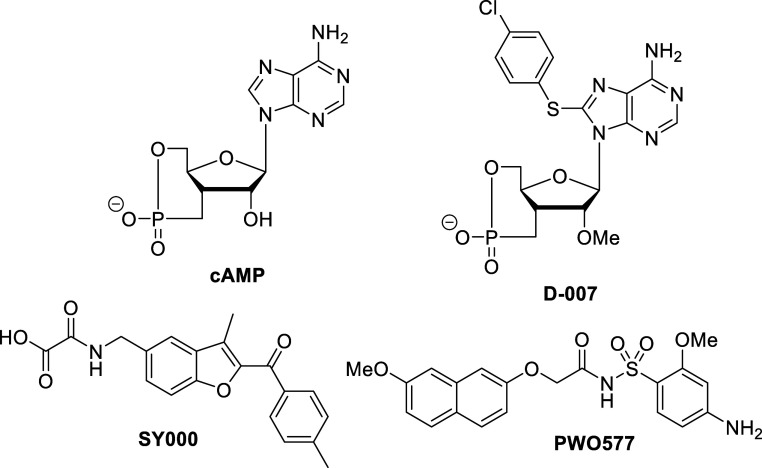
Previously reported EPAC1 activators.

Developing the benzofuran oxoacetic acid series posed considerable synthetic challenges, which we addressed through iterative optimization of our synthetic route. The construction of the fused benzofuran core with an oxoacetic acid functionality required multiple steps that proved low-yielding, particularly during key reduction and oxidation transformations. Early stage modifications at the benzofuran C2 and C3 positions were hampered by limited functional group tolerance in these steps, constraining the scope of attainable analogues. For example, while a first series of C2-aryl ketone analogues was successfully synthesized, attempts to introduce aryl substituents at the C3-position were largely unsuccessful due to incompatibilities with the strong reducing conditions required in the synthetic sequence. Despite these difficulties—including overall yields of only ∼3–7% for the final products—we were able to isolate and characterize a panel of new benzofuran oxoacetic acid compounds for biological evaluation.^22^

Here, we report the development and comprehensive evaluation of these benzofuran oxoacetic acid EPAC1 activators. Biochemical assays demonstrated that many of the new analogues effectively activate EPAC1, with several compounds achieving activation levels comparable to that of SY007, a previously identified potent benzofuran-based EPAC1 agonist. Notably, the new compounds displayed complete isoform selectivity for EPAC1 over EPAC2 and caused no detectable activation of PKA, indicating that the design objectives of isoform specificity and PKA sparing were successfully met. In cellular models relevant to EPAC1’s therapeutic roles, our lead compounds were able to attenuate pro-inflammatory signaling (IL-6/STAT3 pathway) and suppress pro-fibrotic responses (TGF-β1-induced fibroblast-to-myofibroblast transition) at micromolar concentrations. These findings underscore the potential of targeting EPAC1 with isoform-selective agonists as a novel therapeutic strategy for diseases characterized by dysregulated cAMP signaling, particularly those involving inflammation and fibrosis.

## Compound Design

Although initial SAR investigations explored some replacement of the SY007/SY009 *p*-tolyl ring system, substitution patterns on this ring, and particularly ortho-substitution, had not (Figure 2).^22^ We therefore wished to explore chemical space in this area; we also note that the benzofuran 3-substituent was shown to determine EPAC isoform activation selectivity, with **SY009** (R = ^*i*^Pr) a selective EPAC1 activator, while **SY007** (R = Et) binds to the CNBD of both EPAC1 and EPAC2.

**Figure 2:**
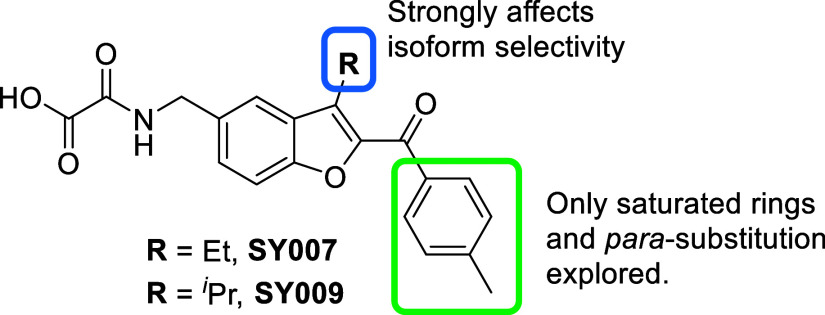
Previous SAR work on benzofuran oxoacetic acid EPAC activators.

The presence of a diarylketone moiety raises concerns for further drug development, and we additionally aimed to explore replacements for this functional group, although we point to several examples of successful drugs featuring biarylketones, including pitofenone, fenofibrate, menbedazole, and tiaprofenic acid.^23–25^ It was hypothesized that a benzofuro­[2,3-*c*]­pyridine core (compound **DM312**, Figure 3) might provide an alternative, with a nitrogen hydrogen bond acceptor replacing the **SY009** ketone oxygen.

**Figure 3:**
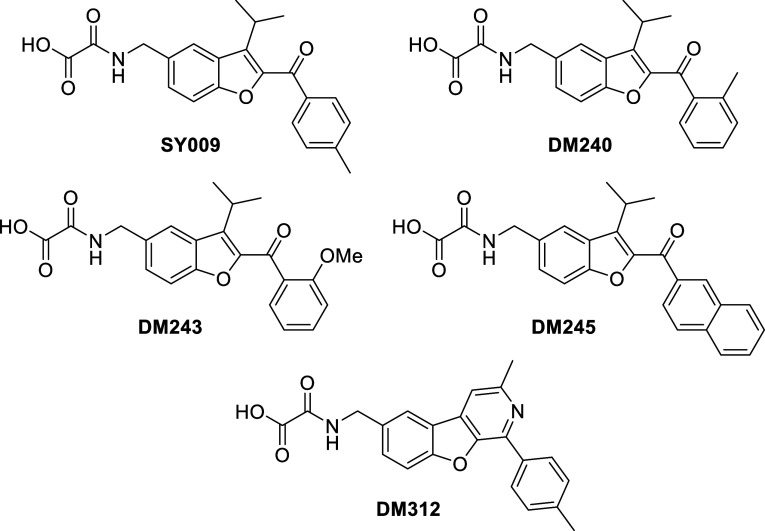
Ketone and nonketone containing EPAC ligands.

Preliminary computational docking modeling against an EPAC1 homology model prepared from an EPAC2-cAMP structure (PDB ID: 4MGK) suggested that **DM312** could occupy the EPAC ligand binding site in the same pose as **SY009** in which the oxoacetic acid acts as a cAMP phosphate mimetic (Figure 4), with the pyridinyl nitrogen and ketone carbonyl oxygen occupying the same volume, and we sought to also synthesize this compound. Selected analogues of **SY009** featuring differing substitution around the terminal ring, including 2-Me (**DM240**), 2-OMe (**DM243**), and a 2-naphthyl (**DM245**) analogues (Figure 3) were also docked, revealing them to occupy the same volume in the cAMP binding pocket at **SY007** and **SY009**, albeit with a 180° rotation of the benzofuran core in the cases of **DM240** and **DM245** (Figure 4).

**Figure 4:**
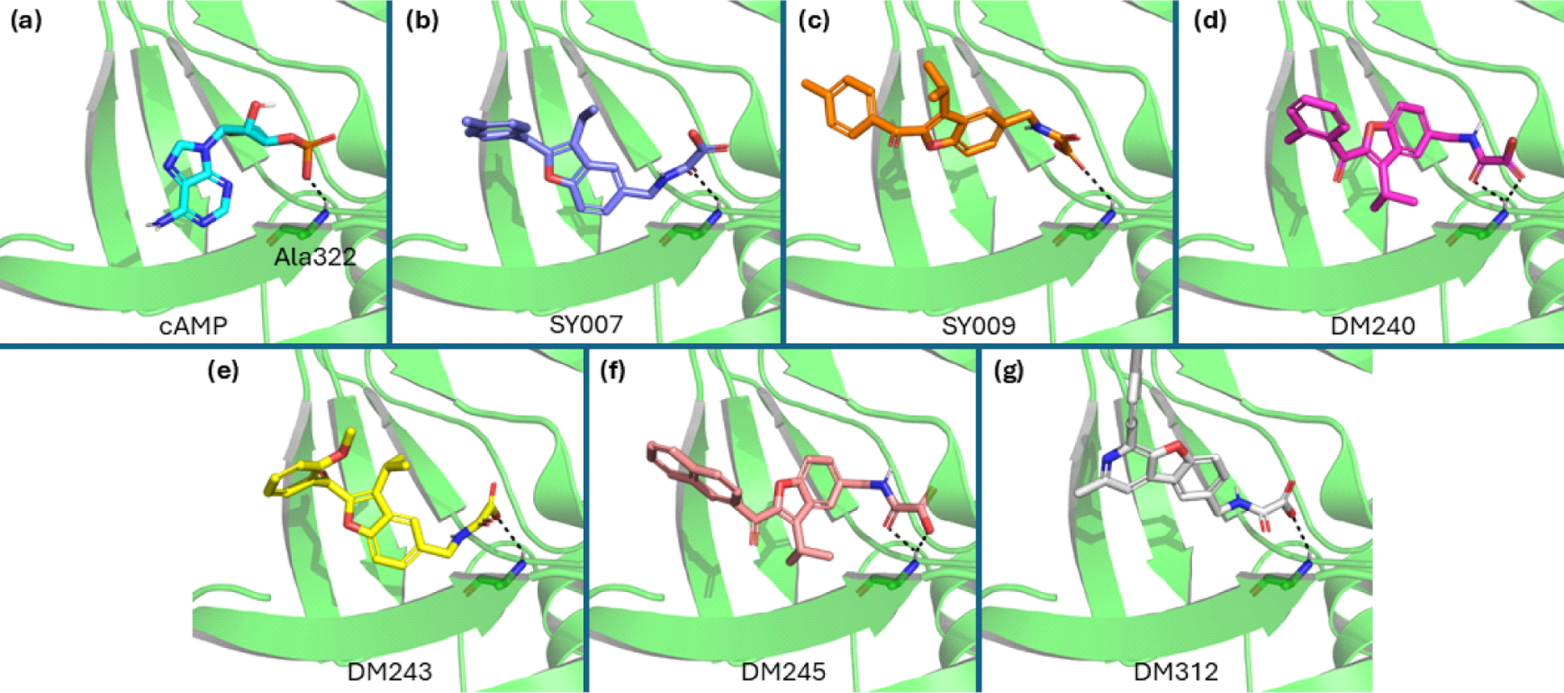
Computational docking of EPAC ligands against an EPAC1 homology model. (a) cAMP (cyan) docked. (b) **SY007** (blue) docked. (c) **SY009** (orange) docked. (d) **DM240** (magenta) docked. (e) **DM243** (yellow) docked. (f) **DM245** (pink) docked. (g) **DM312** (gray) docked.

The selected compounds were also docked against an extant EPAC2 structure (PDB ID: 3CF6) in order to probe relative binding energies and isoform selectivity. With the exception of **DM243**, calculated binding energies for new structures were lower by at least 0.6 kcal mol^–1^ for the EPAC1 homology model vs. EPAC2, suggesting selectivity would be obtained (Table 1). Careful inspection of the docking models did not reveal any clear indication of the structural basis of this selectivity, and we suggest that further computational studies (e.g., using dynamic protein models) will be required to fully elucidate the reasons for isoform selectivity.

**Table 1:** Calculated Binding Energies against an EPAC1 Homology Model and EPAC2 for Selected Benzofuranoxoacetic Acid Ligands

ligand	EPAC1 HM docking score (kcal mol^–1^)	EPAC2 docking score (kcal mol^–1^)
**SY007**	–9.6	–12.4
**SY009**	–10.4	–8.4
**DM240**	–10.4	–9.4
**DM243**	–8.9	–9.7
**DM245**	–10.9	–9.6
**DM312**	–11.3	–10.7

## Synthesis

Compounds **DM239**–**DM245** were synthesized using modified versions of previously published synthesis.^22^ Thus, a series of methyl aryl ketones were brominated using NBS in ≥72% to give **1a**–**1d**. Bromination of 2,4-dimethoxyphenyl methyl ketones gave exclusively products arising from ring halogenation, and the desired product **1e** was instead obtained in 98% yield using CuBr_2_ (Scheme 1).

**Scheme 1:**
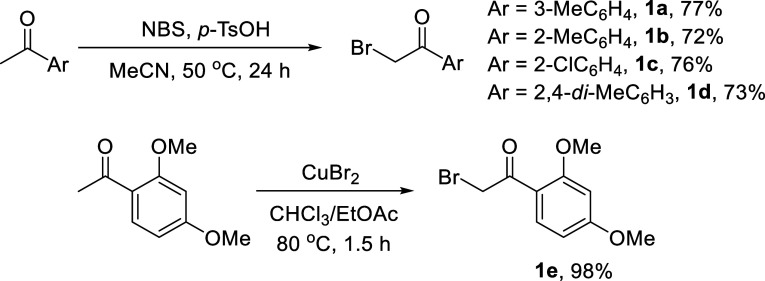


With bromoketones in hand, the benzofuran core of the target structures was assembled using a substitution/ring closing aldol condensation strategy with phenol **2** to give **3a**–**g** in up to 98% yield. Palladium-catalyzed cyanation using Zn­(CN)_2_ gave nitriles **4a**–**g** in up to 92% yield, before global reduction using lithium aluminum hydride gave amino alcohols **5a**–**g** in a modest yield of 35–63%. Reoxidation to the ketone using manganese dioxide also proceeded in a modest yield up to 45% to give **6a**–**g**; 2-chlorophenyl compound **6c** could not be purified and was carried through into the next step impure. Despite the low yields and inconvenience of this global reduction/reoxidation strategy, we were unable to identify effective conditions to selectively reduce nitriles **4a**–**g** to the primary amines while leaving the ketone moiety intact. Similarly, low yields on reoxidation to the ketone are due to competitive oxidation of the primary amine to an arylimine, followed by hydrolysis to aldehyde on aqueous workup; no alternative conditions were found to give improved yields after an exhaustive search. Reaction with ethyl chlorooxoacetate gave **7a**–**g** in up to 69% yield. Finally, ester hydrolysis using NaOH in MeOH/THF gave target compounds **DM239–D245** in up to 99% yields (Scheme 2).

**Scheme 2:**
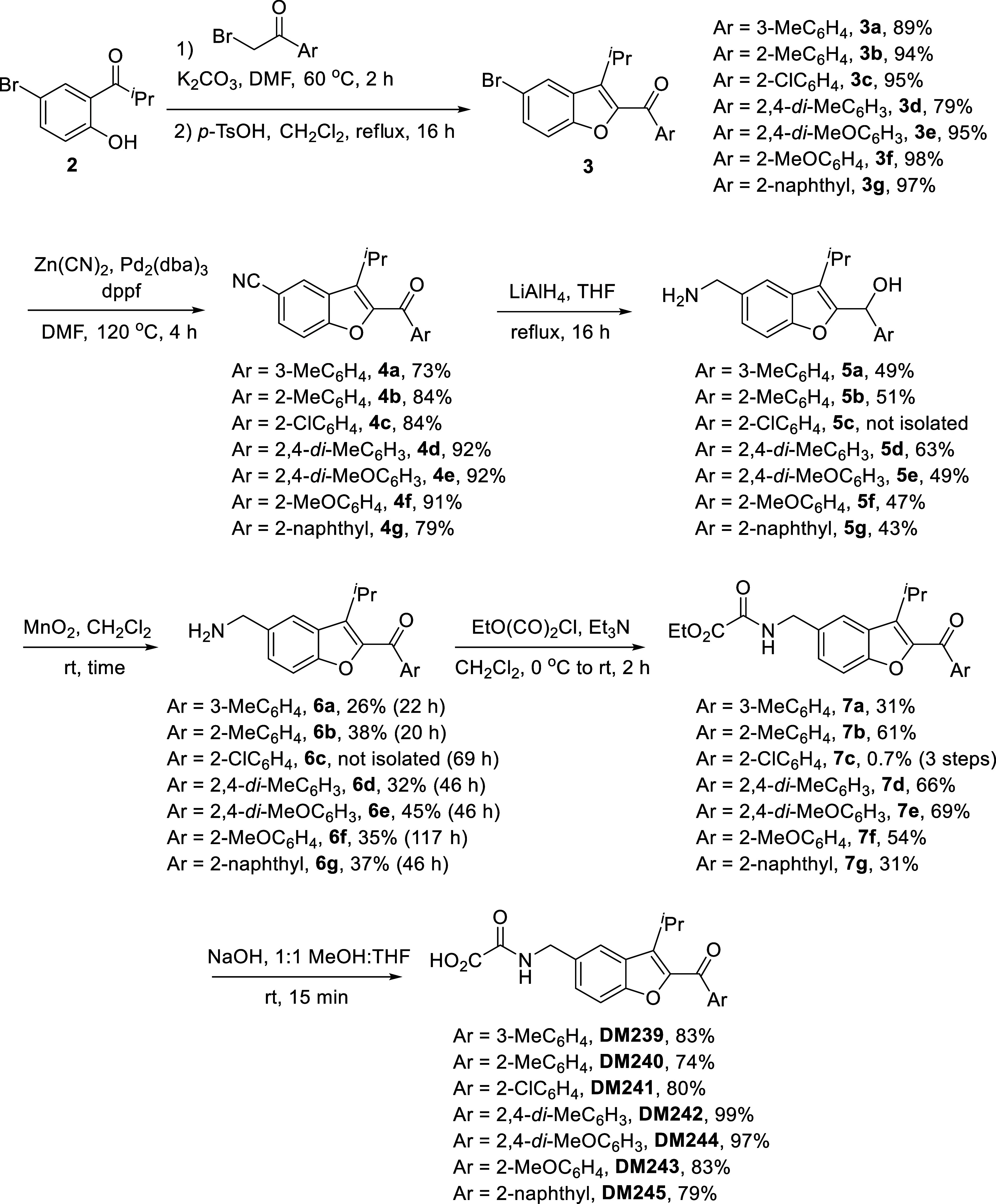


In addition to the low yield of the reduction/reoxidation sequence, the harsh conditions used limit the functional group compatibility and thus the chemical space accessible. Additionally, this route uses toxic zinc cyanide, and the crucial benzofuran 3-substituent (known to control EPAC isoform selectivity),^22^ as well as the terminal aryl ring are installed late in the sequence, making library synthesis time-consuming. We therefore decided to explore a new synthetic route in which both these moieties could be installed onto a late-stage common synthetic intermediate. We identified cyanophenol **13** as a target which could be prepared on a multigram scale, based on an amination of benzaldehydes using *tert*-butyl carbamate reported by Gütschow and co-workers in 2020,^26^ following synthesis of aldehyde **12** as described by Zhang et al. in 2018.^27^ To our delight, synthesis proceeded smoothly: starting from 4-hydroxybenzaldehyde **8**, bromination using Br_2_ gave **9** in 59% yield, followed by benzyl protection of the phenol to give **10** in 93% yield. Cyanation using copper­(I) cyanide then gave **11** in 79% yield. Deprotection of the benzyl ether proceeded in high (77%) yield under standard (Pd/C, H_2_) hydrogenation conditions to give **12**. Finally, reductive amination with *tert*-butyl carbamate gave our target **13** in 79% yield; in total, 5 g of **13** was prepared in 5 steps and 26% overall yield (Scheme 3).

**Scheme 3:**
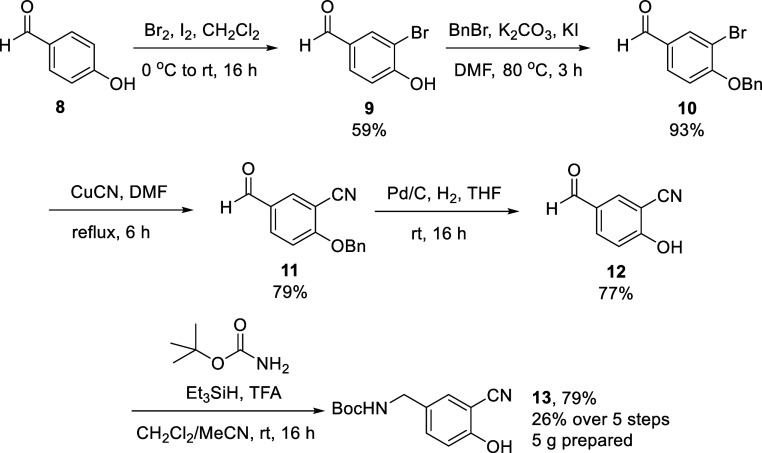


With **13** in hand, we first set out to resynthesize **SY009**. The benzofuran 3-isopropyl substituent was installed using a Grignard reagent to give **14** in 68% yield before substitution/ring closing aldol condensation with a bromoketone, as before, completed the benzofuran core of the target to give **15** in 68% yield. Boc deprotection using trifluoroacetic acid and installation of an oxalyl ester amide proceeded in 43% yield, giving **7h** before ester hydrolysis completed the synthesis in 85% yield; overall, **SY009** was accessed in 4 synthetic operations and 17% yield from common intermediate **13** (Scheme 4).

**Scheme 4:**
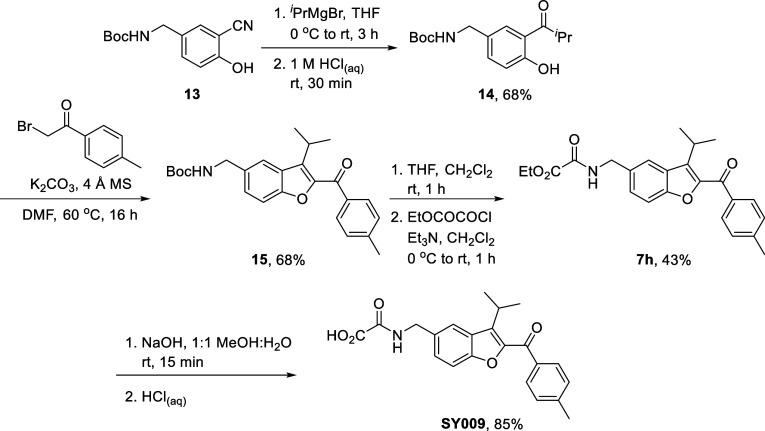


Resynthesis of **SY007** using this route encountered some differences; elimination of H_2_O during ring-closing aldol condensation required facilitation with *p*-TsOH, with **17** being obtained in 69% yield. Additionally, after *N*-Boc deprotection, diamidation with ethyl oxalyl chloride was encountered, with **18** the only product isolated, in 86% yield. Fortunately, one oxalyl amide moiety could be selectively hydrolyzed with LiOH along with the other ester, and **SY007** was obtained in 5 steps and 25% overall yield from common intermediate **13** (Scheme 5).

**Scheme 5:**
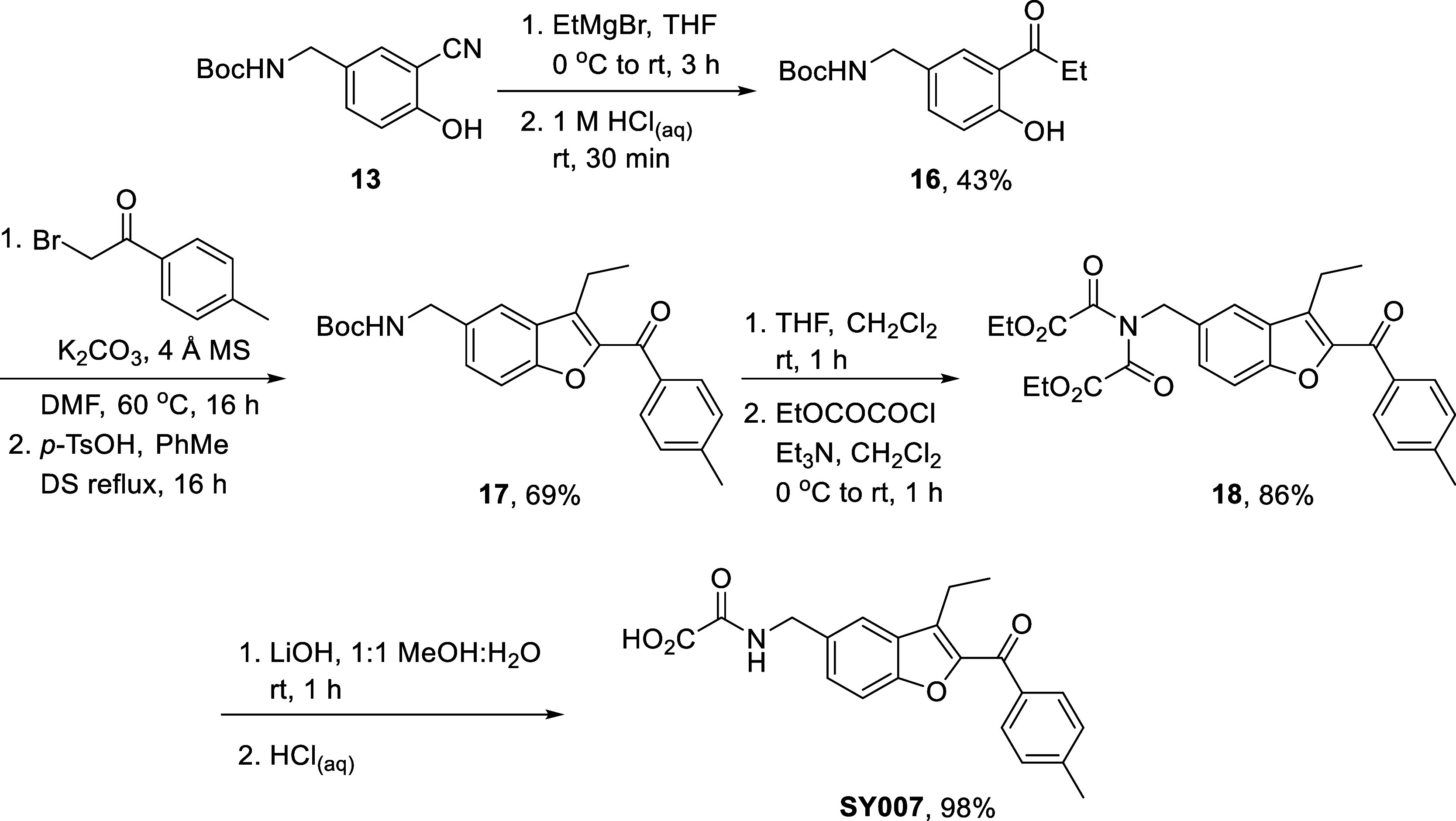


We additionally hoped to synthesize **DM357,** in which the *p*-tolyl ring is replaced by *m*-hydroxyphenyl, and which could not be successfully obtained from our original synthetic route. 3-Hydroxyacetophenone **19** was first protected with *tert*-butyldimethyldsilyl chloride to give **20** in 97% yield, before bromination gave **21** in 94%. From there, substitution/aldol ring closing with **14** (see Scheme 2, above) resulted in concomitant silyl ether deprotection and gave **22** in 51% yield, installation of the ethyl oxalyl amide gave **23** in 73% yield, and the synthesis was completed to give **DM357** in 96% yield (Scheme 6).

**Scheme 6:**
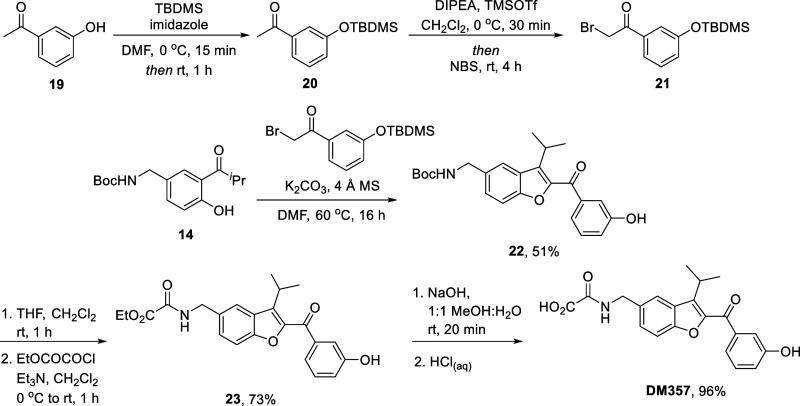


Finally, the synthesis of fused pyridyl compound **DM312** was carried out. Starting from 5-bromo-2-hydroxybenzaldehyde **24**, aldol condensation with acetone gave **25** in 93% yield, with only the *E*-isomer observed by ^1^H NMR. Williamson ether synthesis with **1h** to append the terminal *p*-tolyl ring then gave **26** in quantitative yield. Next, the tricyclic core could be assembled using ammonium acetate, following an intramolecular annulation procedure reported by Duan et al. in 2014, to give **27** in 53% yield.^28^ Pd-catalyzed cyanation then gave **28** in 94%. Nitrile reduction using LiAlH_4_, followed by installation of the ethyl oxalyl amide to give **29,** proceeded in very low (4.6%) yield; *in lieu* of further optimization, we decided to carry a small amount of material forward and revisit this step should the final product have promising EPAC activation properties. Finally, ester hydrolysis gave **DM312** in 74% yield (Scheme 7).

**Scheme 7:**
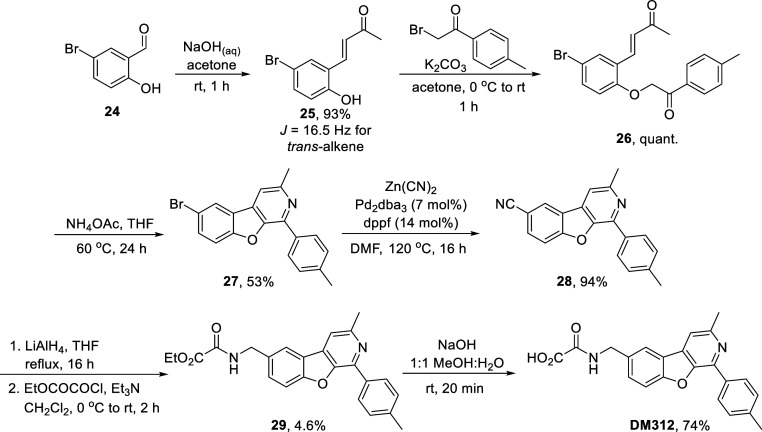


## Results and Discussion

### EPAC1 vs EPAC2 Competition Binding Studies

To examine whether engagement of EPAC isoforms correlates with the cellular performance of the benzofuran oxoacetic acid series (DM compounds), we quantified binding to the isolated cyclic-nucleotide–binding domains (CNBDs) of EPAC1 and EPAC2 using 8-NBD-cAMP competition assays (Figure 5). The canonical EPAC1-selective cAMP analogue **D-007** and the EPAC2-selective analogue **S-220** ^13,14^ reproduced their expected profiles and highlighted assay selectivity, yielding pIC_50_ values of 5.965 at EPAC1-CNBD and 5.850 at EPAC2-CNBD, respectively.

**Figure 5:**
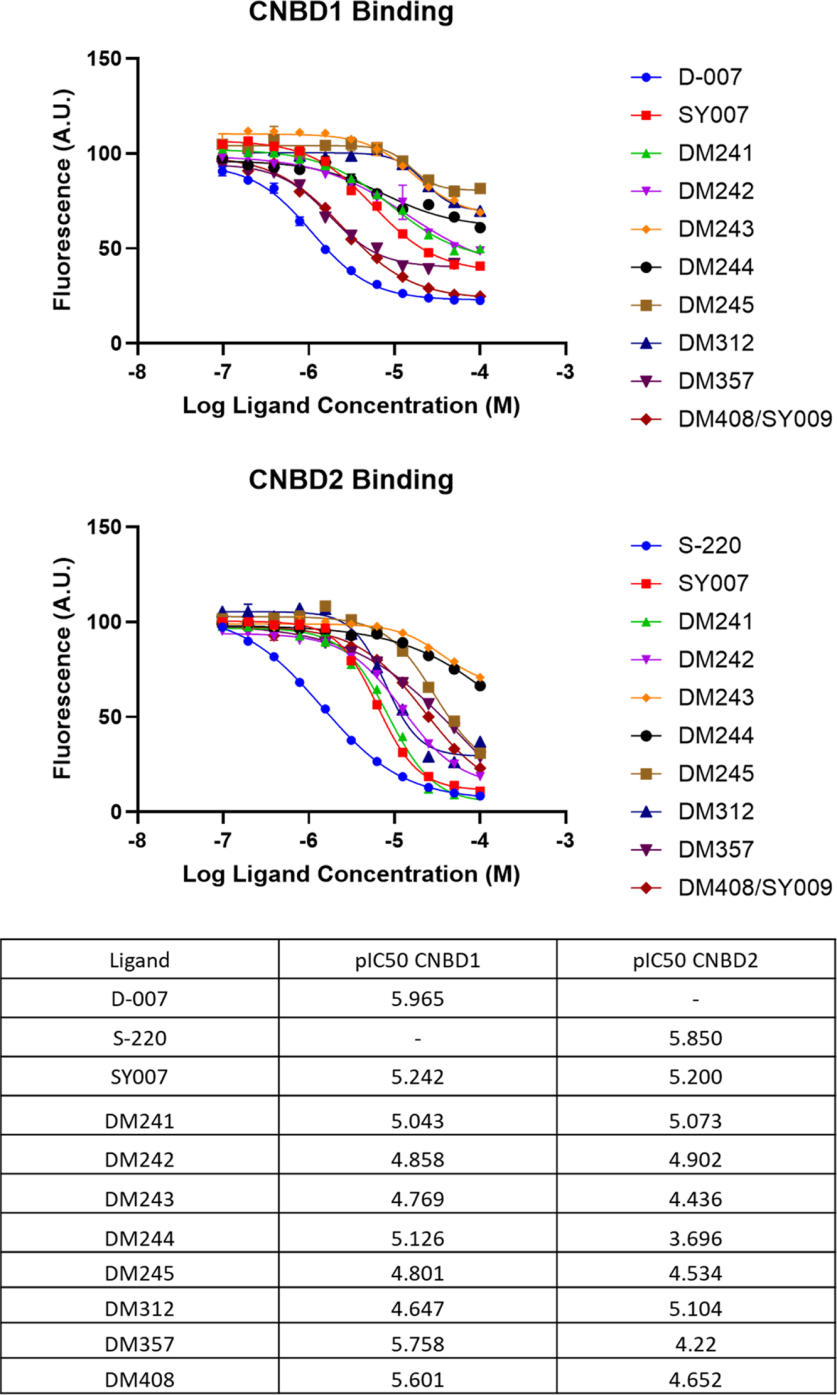
cAMP competition binding to EPAC CNBDs.

Within the DM set, several compounds displaced tracer in a concentration-dependent manner with distinct isoform preferences. The most EPAC1-biased binders were **DM357** (pIC_50_ = 5.758 at EPAC1 vs 4.220 at EPAC2; ∼35-fold preference) and **DM244** (5.126 vs 3.696; ∼27-fold), with **DM408** also favoring EPAC1 (5.601 vs 4.652; ∼9-fold). **DM241** and **DM242** bound both EPAC isoforms with little discrimination (EPAC1/EPAC2 pIC_50_ ≈ 5.04/5.07 and 4.86/4.90, respectively), while **DM243** showed modest EPAC1 preference (4.769 vs 4.436; ∼2-fold). In contrast, **DM312** was EPAC2-biased (4.647 at EPAC1 vs 5.104 at EPAC2; ∼3-fold for EPAC2). The reference analogue **SY007** was essentially nonselective (5.242 vs 5.200). Collectively, these data demonstrate that the series contains both EPAC1-favored (**DM357**, **DM243**, **DM244**, and **DM408**) and EPAC2-favored (**DM312**) chemotypes, providing a useful starting point for isoform-directed optimization rather than suggesting parity with the gold-standard tool ligands.

The binding of benzofuran oxoacetic acids (DM series) to EPAC1-CNBD (top) and EPAC2-CNBD (bottom) was measured by fluorescence competition with 8-NBD-cAMP at a fixed tracer concentration. Increasing concentrations of each compound (legend at right; D-007 and S-220 included as positive controls) displaced tracer, and the decrease in fluorescence (A.U.) was fitted with four-parameter logistic models to obtain pIC_50_ (−log_10_ IC_50_ [M] in Table). Points are mean ± SEM from 3 independent experiments; curves are best-fit 4-point logic functions.

### Evaluation of STAT3 Inhibition

Building on prior evidence that EPAC1 activation by I942 suppresses STAT3 signaling,^17^ we assessed whether benzofuran oxoacetic acid analogues attenuate **IL-6/IL-6Rα**–evoked STAT3 phosphorylation in HUVECs. Immunoblotting confirmed a robust induction of phospho-STAT3 (Tyr705) by **IL-6/IL-6Rα** relative to diluent (Figure 6, upper panel). At 1.0 μM, several analogues reduced this response after normalization to total STAT3 (Figure 6, lower panel). **DM243** produced the largest decrease in the normalized p-STAT3 signal, with **DM244** and **DM245** each reaching statistical significance; **DM357** also significantly lowered phospho-STAT3. By contrast, **DM241**, **DM242,** and **DM312** yielded only partial attenuation that did not achieve significance under one-way analysis of variance (ANOVA) with Dunnett’s posthoc comparison versus the **IL-6/IL-6Rα** condition (ns). Reference EPAC activators (**D-007**,^13,14^ **SY007** ^22^) and the related analogue **DM408/SY009** ^22^ likewise showed nonsignificant reductions relative to **IL-6/IL-6Rα** under these conditions (ns). These results position members of the benzofuran oxoacetic acid series, most notably **DM243**, as EPAC-pathway-linked modulators capable of dampening **IL-6**–driven STAT3 activation at 1.0 μM, while delineating clear activity differences across the series.

**Figure 6:**
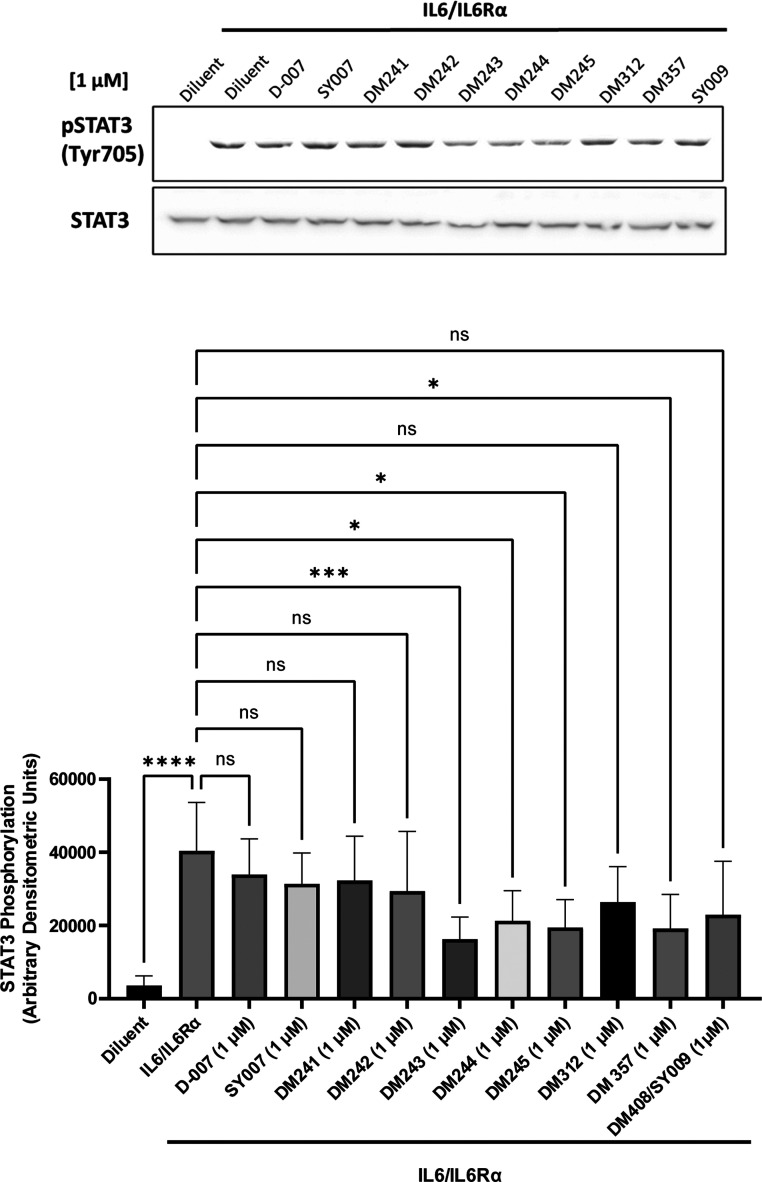
EPAC1 agonists reduce IL-6-driven STAT3 phosphorylation: immunoblot, group-wise densitometry, and pairwise contrasts. Upper panel (immunoblot): human umbilical vascular endothelial cells were stimulated with **IL-6** in the presence of soluble **IL-6Rα** and treated with the indicated benzofuran oxoacetic acid analogues (1 μM) or controls, then lysed for SDS-PAGE and immunoblotting. Phospho-STAT3 (Tyr705) is shown above; total STAT3, used as the loading/normalization control, is shown below. Representative blots are presented. Middle panel (group-wise densitometry vs **IL-6/IL-6Rα**): bars display densitometric quantification of phospho-STAT3 for each condition (arbitrary densitometric units, ADU). Signals were background-subtracted and normalized to total STAT3 from the same lane, then aggregated across independent experiments. Bars show mean ± SEM. Statistical comparisons were performed against the **IL-6/IL-6Rα** condition using one-way ANOVA with Dunnett’s posthoc test. Significance codes: ns (not significant), *p < 0.05, **p < 0.01, ***p < 0.001 (*n* = 5). As expected, **IL-6/IL-6Rα** markedly increased STAT3 phosphorylation over diluent, and several benzofuran analogues reduced the normalized p-STAT3 signal at 1 μM.

### Evaluation of TGFβ-Induced Fibroblast-to-Myofibroblast Transition Inhibition

To evaluate antifibrotic efficacy in a cellular context, we performed **TGF-β1**-induced fibroblast-to-myofibroblast transition (FMT) assays and monitored the canonical readouts αSMA and Collagen I. FMT transition is a key process in various pathological conditions, including fibrosis. This was done given reports that EPAC1 is involved in the suppression of fibrosis.^29,30^ Normal human lung fibroblasts were stimulated with **TGF-β1** for 72 h and treated with a representative benzofuran oxoacetic acid analogue and control stimuli over a logarithmic concentration range (Figure 7). Quantitative high-content imaging with single-cell segmentation was used to compute mean cellular fluorescence per well. Representative fields are shown at low = 1.0 × 10^–6^ M and high = 1.0 × 10^–4^ M (annotated on each tile), together with vehicle-only, no-**TGF-β1** controls; scale bar = 10 μm in all images. Qualitatively, **DM243** reduced stress-fiber-like αSMA architecture and lowered Collagen I signal relative to the **TGF-β1** condition.

**Figure 7:**
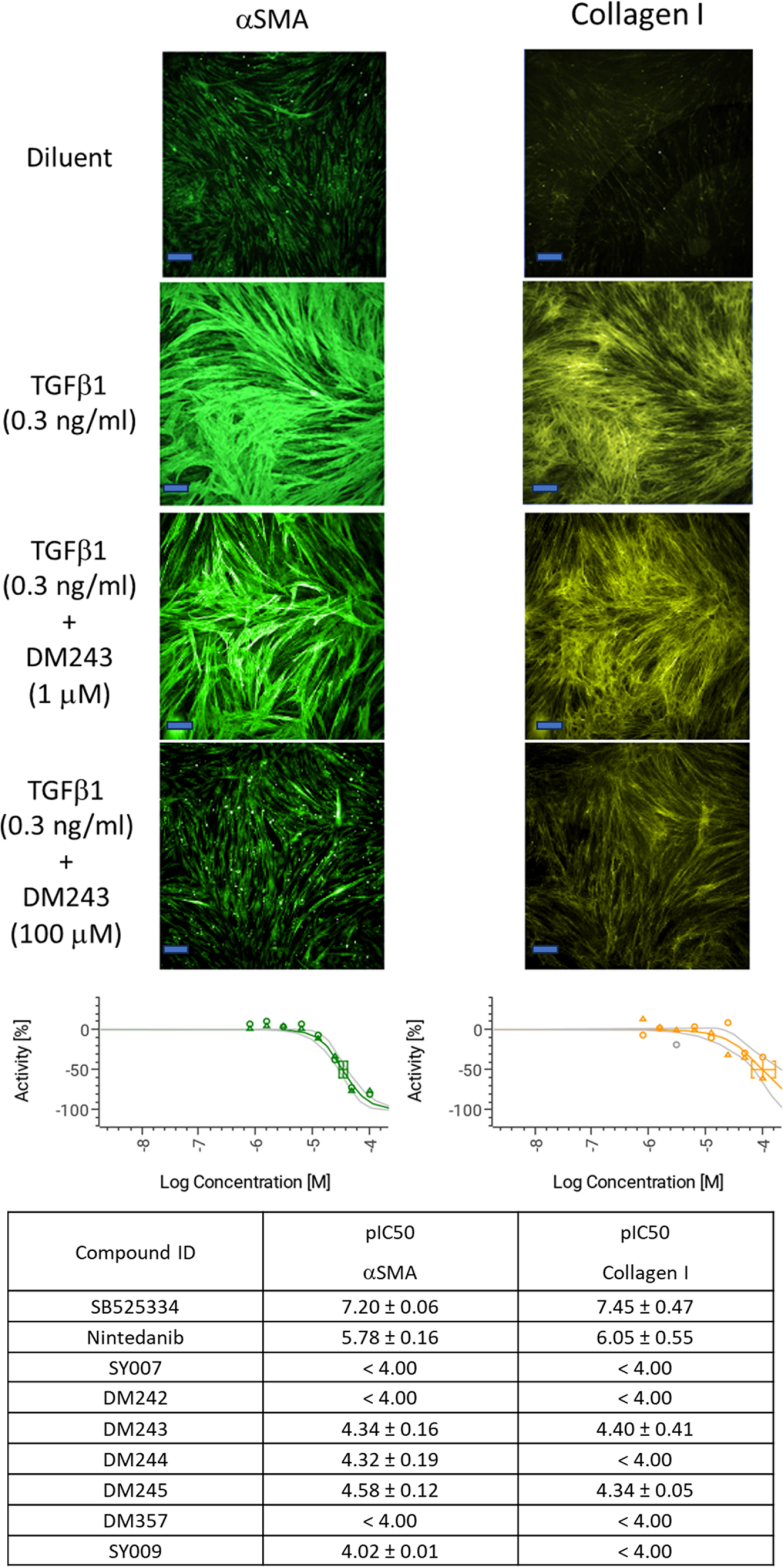
Inhibition of profibrotic TGF-β1 signaling by benzofuran oxoacetic acid analogues.

Dose–response analysis of the **TGF-β1**–induced FMT assay yielded the following potencies (pIC_50_, mean ± SEM) for the two markers, **αSMA** and **Collagen I** (Figure 7 lower panel). The benchmark **TGF-β** receptor inhibitor **SB525334** was the most potent in both readouts (αSMA 7.20 ± 0.06; Collagen I 7.45 ± 0.47), and the approved antifibrotic, JAK/STAT inhibitor, **nintedanib,** was likewise submicromolar (αSMA 5.78 ± 0.16; Collagen I 6.05 ± 0.55), confirming assay performance and dynamic range. Within the benzofuran oxoacetic acid series, **DM245** was the strongest overall (αSMA 4.58 ± 0.12; Collagen I 4.34 ± 0.05), followed by **DM243** with closely matched activity across markers (αSMA 4.34 ± 0.16; Collagen I 4.40 ± 0.41). **DM244** showed selective activity for αSMA (4.32 ± 0.19) with Collagen I < 4.00, whereas **DM408/SY009** was borderline on αSMA (4.02 ± 0.01) and inactive on Collagen I (**<**4.00). **SY007**, **DM242**, and **DM357** were weak performers, each returning pIC_50_ < 4.00 for both markers.

Taken together, these data show that several benzofuran oxoacetic acids elicit clear, concentration-dependent suppression of both **αSMA** and **Collagen I** in the midmicromolar IC_50_ range (e.g., **DM243** and **DM245**), whereas others are inactive at the concentrations tested. Importantly, all new analogues are less potent than the benchmarks (**SB525334**, **nintedanib**) by 1 to 3 orders of magnitude, positioning them as chemically tractable starting points for optimization rather than replacements for established tool inhibitors.

Upper panels (immunofluorescence): normal human lung fibroblasts (Lonza, CC-512) were stimulated with **TGF-β1** for 72 h and treated with **DM243** over a concentration range. Cells were fixed, permeabilized, and stained with antibodies to α-smooth muscle actin (αSMA; left, green) or Collagen I (right, yellow), as detailed in the Methods section. Scale bar = 10 μM. Representative fields are shown. Middle panels (concentration–response analysis): **DM243** effects on αSMA and Collagen I were quantified by high-content imaging, with single-cell segmentation and mean cellular fluorescence intensity per well. Responses were background-subtracted and expressed as % activity relative to **TGF-β1** alone (0% = **TGF-β1** response; −100% = complete suppression). Data points show mean ± SEM from 4 independent experiments; curves are four-parameter logistic fits used to derive pIC_50_ (−log_10_ IC_50_ [M]). Quality control criteria included plate *Z*′ > 0.4, and IC_50_ estimation was performed in Screener (Genedata). Lower panel (data table): Across both markers, several benzofuran oxoacetic acids produced robust, concentration-dependent inhibition, with **DM243** and **DM245** among the most potent; **SY007**, **DM242,** and **DM357** showed weaker activity (pIC_50_ < 4). The **TGF-β** receptor inhibitor **SB525334** and the antifibrotic drug **nintedanib** were included as benchmarks for assay performance.

### Comparative Cytotoxicity versus Nintedanib

Using DAPI nuclear staining as a surrogate for cell number/viability after 72 h exposure (Figure 8), **nintedanib** produced a clear, concentration-dependent loss of intact nuclei across the same concentration window used for the **TGF-β1** FMT assays. In contrast, the benzofuran oxoacetic acids **DM243**, **DM244**, and **DM245** showed minimal change in nuclear counts under matched conditions, yielding shallow or nonmeasurable cytotoxic concentration–response curves within the tested range. Thus, at concentrations effective on profibrotic readouts, these analogues exhibit a markedly improved cytotoxicity profile relative to **nintedanib**.

**Figure 8:**
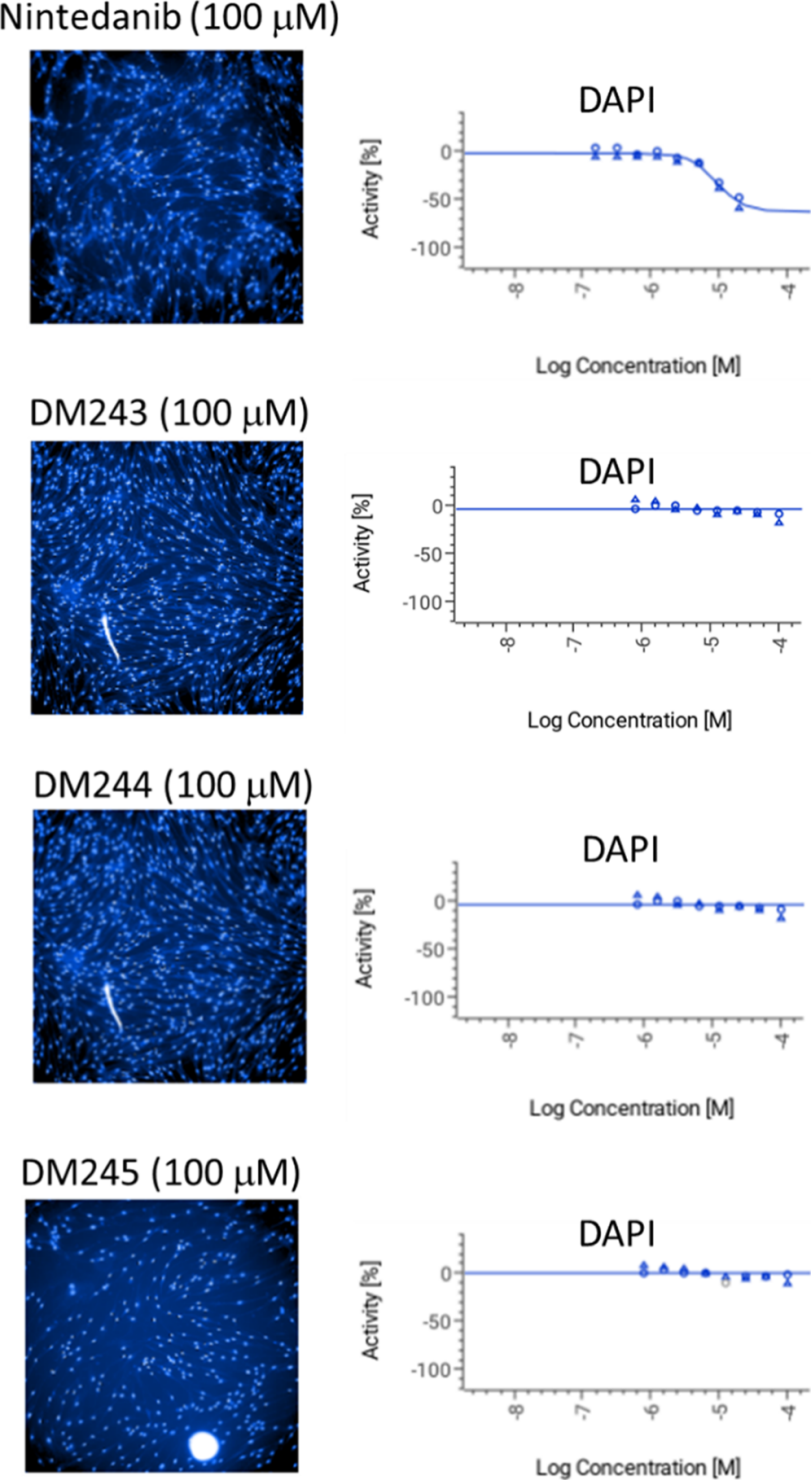
Cytotoxic effects of nintedanib, in comparison with benzofuran oxoacetic acid compounds.

Left panels (DAPI imaging): Normal human lung fibroblasts were exposed for 72 h to **nintedanib** or the indicated benzofuran oxoacetic acid compounds identical to that used in the **TGF-β**1 assays. Cells were fixed and stained with DAPI to visualize nuclei; representative fields are shown to illustrate changes in cell number/morphology. Right panels (concentration–response quantification): Cytotoxicity was assessed by high-content imaging as the count of intact DAPI-positive nuclei per well. Values were background-subtracted and expressed as % activity relative to vehicle controls from matched plates (0% = no change vs vehicle; −100% = complete loss of nuclei). Points represent mean ± SEM from 4 independent experiments; curves are four-parameter logistic fits used to estimate pIC_50_ where measurable. Across the tested range, **nintedanib** produced a clear, concentration-dependent loss of DAPI-positive nuclei, consistent with cytotoxicity. In contrast, the benzofuran oxoacetic acid analogues showed minimal effects on nuclear counts under matched conditions, with shallow or no measurable cytotoxic concentration–response within the tested window. These results indicate that, at concentrations effective on profibrotic readouts, the benzofuran series exhibits substantially lower cytotoxicity than **nintedanib**.

### Evaluation of Rap1 Activation

To test whether cellular EPAC activation mirrors our biochemical and phenotypic findings, we quantified Rap1 nucleotide loading in EPAC-transfected U2OS cells (Figure 8). In EPAC1-expressing cells, several benzofuran oxoacetic acid analogues elevated Rap1-GTP above diluent controls (Figure 9). The largest and statistically significant increases were produced by **DM243**, **DM244**, and **DM245** (one-way ANOVA with Dunnett’s posthoc test versus diluent), whereas **SY007**, **DM357**, and **DM408/SY009** did not differ significantly from diluent under the same conditions. In EPAC2-expressing cells, none of the tested compounds produced a significant change in Rap1-GTP; all comparisons to diluent were not significant. Thus, within this cellular context, the series shows a functional preference for EPAC1 over EPAC2, with **DM243**, **DM244**, and **DM245** emerging as the most effective EPAC1 activators.

**Figure 9:**
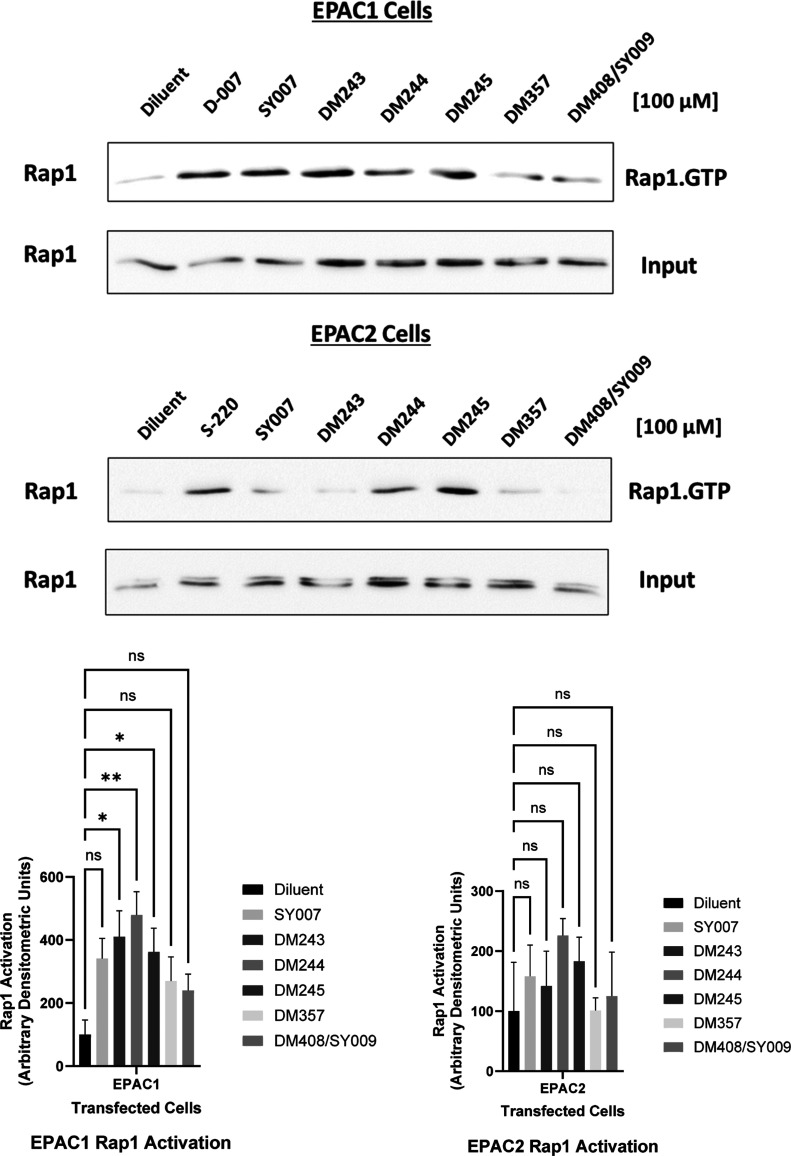
Cellular Rap1 activation by benzofuran oxoacetic acid compounds. Upper panels (immunoblots): EPAC-transfected U2OS cells were treated with the indicated benzofuran oxoacetic acid analogues or controls and lysed for Rap1 pull-down assays. Active Rap1-GTP was isolated using GST–RalGDS-RBD, followed by immunoblotting with a Rap1-specific antibody; total Rap1 in whole-cell lysates is shown as an input/loading reference. Experimental details for cell lines, RalGDS-RBD production, and immunoblotting are provided in the Methods section. Lower panel (densitometry): bars depict quantification of Rap1-GTP from independent experiments (ADU), background-subtracted and normalized to total Rap1 from the corresponding lane (mean ± SEM). Statistical testing used one-way ANOVA with Dunnett’s posthoc comparison to vehicle unless otherwise indicated; significance codes: ns, not significant; *P < 0.05 and **P < 0.01. In EPAC1-expressing cells, **DM243**, **DM244**, and **DM245** produced the greatest increases in Rap1-GTP, consistent with selective cellular activation of the EPAC1–Rap1 pathway. In EPAC2-expressing cells, only modest, nonsignificant elevations were observed for **DM243**, **DM244** and **DM245**, while **DM243** had negligible effect, indicating functional preference for EPAC1 in cells.

### PKA Activation Assays

Benzofuran oxoacetic acid analogues were also evaluated for their potential to activate PKA monitored through the phosphorylation state of a downstream PKA effector, vasodilator-stimulated phosphoprotein (VASP). The adenylate cyclase activator, forskolin, in combination with the PDE4 inhibitor, rolipram (F/R), were used as positive controls in Western blot studies due to their ability to activate PKA and stimulate phosphorylation of VASP (Figure 10). The benzofuran oxoacetic acid analogues induced no PKA activation in EPAC1 transfected U2OS cells and minimal activation in EPAC2 transfected cells, suggesting excellent EPAC/PKA selectivity and potential avoidance of PKA activation side effects.

**Figure 10:**
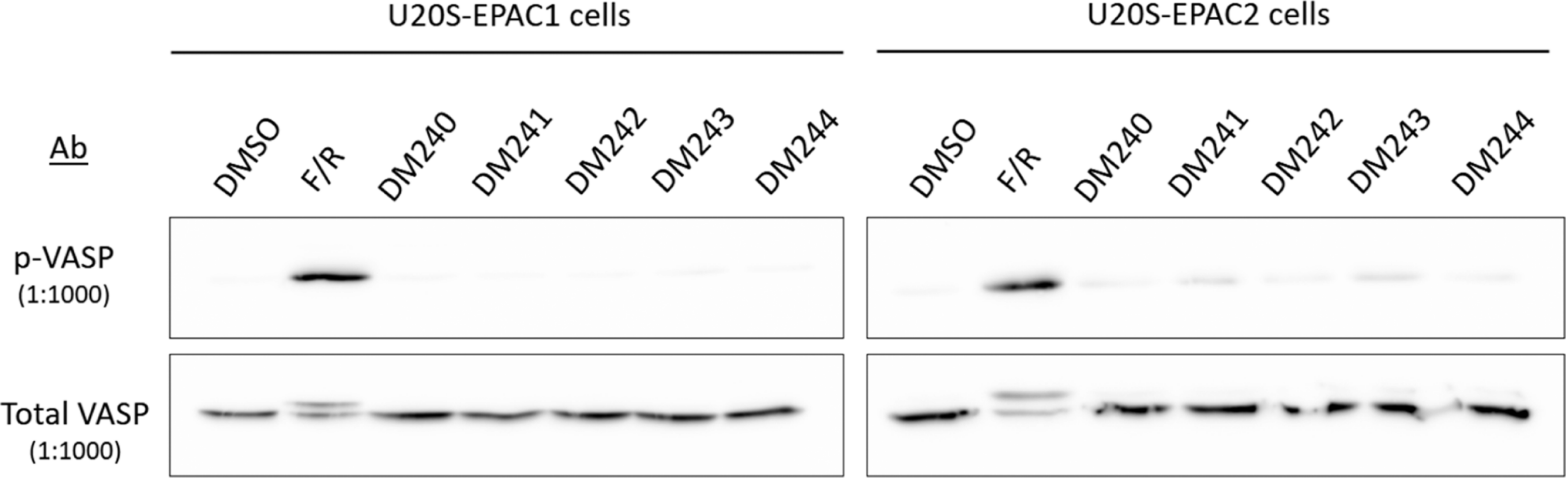
PKA activation assays in U20S-EPAC1/2 transfected cells using phospho-VASP.

U2OS cells stably expressing EPAC1 or EPAC2 were treated with vehicle (DMSO), a positive PKA stimulus (F/R; forskolin + rolipram), or the indicated benzofuran oxoacetic acid analogues (**DM240**–**DM244**; 1 μM), followed by lysis and immunoblotting. Phospho-VASP (Ser157) was used as the readout of PKA activity, with total VASP probed on the same membranes as the loading/normalization control. Representative blots are shown for each cell line (top: *p*-VASP; bottom: total VASP). As expected, F/R produced a robust increase in VASP phosphorylation in both U2OS-EPAC1 and U2OS-EPAC2 cells, confirming assay performance. In contrast, none of the benzofuran oxoacetic acid analogues induced detectable VASP phosphorylation above vehicle in either background, indicating no measurable PKA activation under these conditions. These results support that the DM series does not activate PKA in cells at the concentrations tested and therefore is unlikely to confound EPAC-selective signaling readouts.

## Conclusions

In this study, we report the discovery and characterization of a novel class of benzofuran oxoacetic acid small molecules as selective EPAC1 agonists and establish them as tractable, noncyclic modulators of EPAC signaling and delineate their behavior across biochemical and cellular assays relevant to inflammation and fibrosis. A convergent synthetic route was established to access these analogues, focusing on introducing structural diversity at both the C2 and C3 positions of the benzofuran core. Although this strategy ultimately produced a series of final analogues, the chemistry involved significant challenges. Key transformations required careful control of reductive cyclization and oxidation steps. Attempts to install substituents at the benzofuran C3 position were problematic due to incompatibility with the strong reducing conditions (e.g., LiAlH_4_) required for cyclization, whereas analogous modifications at the C2 position were successful. As a result of these constraints, the overall yields of the final compounds were modest (approximately 3–7%), underscoring the synthetic complexity of this scaffold.

Biochemical engagement of EPAC isoforms was confirmed by tracer competition at isolated cyclic-nucleotide binding domains. As expected, canonical controls reproduced their profiles (**D-007** favoring EPAC1; **S-220** favoring EPAC2), thereby anchoring assay selectivity. Within the new series, small C2 changes tuned isoform preference: **DM244**, **DM357,** and **DM408** were EPAC1-biased, **DM312** was EPAC2-biased, and **DM241**, **DM242,** and **DM243** were near-nonselective with modest biases. These observations validate the scaffold as a platform for isoform direction and indicate that subtle stereoelectronic edits around C2 can shift the EPAC1/EPAC2 balance.

The pharmacological consequences of EPAC1 activation by these compounds were evaluated in cellular models of inflammation and fibrosis. Cellular readouts showed that this biochemical engagement can translate into signaling. In EPAC-transfected U2OS cells, pull-down of Rap1-GTP revealed significant EPAC1-pathway activation by **DM243**, **DM244,** and **DM245**, whereas **SY007**, **DM357,** and **DM408/SY009** did not differ from diluent under the same conditions. In EPAC2-expressing cells, none of the compounds produced a significant change in Rap1-GTP, implying a functional preference for EPAC1 in this cellular context at the exposures tested. These results align with the binding-derived possibility of EPAC1-directed pharmacology and identify **DM243/DM244/DM245** as the most effective cellular EPAC1 activators in our current set.

Downstream biological consequences were evaluated in primary-like systems. In HUVECs, **IL-6/IL-6Rα**-evoked STAT3 phosphorylation decreased most with **DM243** and also reached significance with **DM244**, **DM245,** and **DM357**, whereas **D-007** and **SY007** were not significant at 1 μM. In lung fibroblasts subjected to **TGF-β1**–driven fibroblast-to-myofibroblast transition, concentration–response modeling of high-content imaging identified **DM243** and **DM245** as the most active overall (αSMA pIC_50_ 4.58 ± 0.12 and 4.34 ± 0.16; Collagen I 4.34 ± 0.05 and 4.40 ± 0.41, respectively). **DM244** showed αSMA activity (4.32 ± 0.19) but was <4.00 on Collagen I, while **DM408/SY009** was borderline on αSMA (4.02 ± 0.01) and <4.00 on Collagen I; **SY007**, **DM242,** and **DM357** were weak for both markers. Benchmark controls behaved as expected and were more potent: **SB525334** was sub-100 nM on both markers (αSMA 7.20 ± 0.06; Collagen I 7.45 ± 0.47), and **nintedanib** was low- to submicromolar (αSMA 5.78 ± 0.16; Collagen I 6.05 ± 0.55). Thus, the benzofurans exhibit midmicromolar cellular potency, clearly active but not on par with the benchmarks.

Importantly, cytotoxicity profiling revealed a favorable tolerability window for the series. Across the same concentration range used in the **TGF-β1** assays, **nintedanib** produced a robust, monotonic loss of DAPI-positive nuclei, whereas **DM243**, **DM245,** and **DM312** showed little or no measurable cytotoxic effect. This divergence indicates that, at concentrations effective on profibrotic readouts, the benzofuran oxoacetic acids can separate efficacy from overt toxicity better than **nintedanib** under our conditions, a property that merits further exploration in prolonged assays and coculture models.

The collective data set supports a coherent sequence from target engagement to cellular mechanism while acknowledging divergences between formats. In general, the highest-affinity EPAC1 binders (**DM243**/**DM244**/**DM245**) produce the strongest Rap1 activation in EPAC1-transfected cells and the largest reductions in **IL-6/IL-6Rα**-driven p-STAT3, whereas weaker binders show attenuated or variable effects. Where alignment is imperfect, several mechanisms likely contribute: (i) affinity does not guarantee efficacy, because partial agonism and the efficiency of coupling in the full-length protein can differ from behavior at an isolated CNBD; (ii) cellular factors, including permeability, intracellular accumulation, serum binding, metabolism/efflux, and endogenous EPAC1/EPAC2 levels, modulate effective exposure; (iii) assay architecture differs in sensitivity and dynamic range (e.g., pull-down of Rap1-GTP versus the distal position of p-STAT3 in the EPAC1/Rap axis); and (iv) compound behavior such as solubility limits may compress apparent potencies. Off-target PKA activity was specifically evaluated by phospho-VASP and was not detected under our conditions, arguing against PKA as a confounder. Several inconsistencies are informative. **SY007** and **DM357** exhibit measurable EPAC engagement in vitro yet weak effects on p-STAT3 and FMT end points, consistent with limited cellular exposure and/or partial agonism that fails to propagate to slow phenotypes. Marker asymmetry in FMT, e.g., activity on αSMA without commensurate effects on Collagen I for **DM244**, likely reflects distinct regulation and quantification floors for cytoskeletal versus matrix readouts.

The structure–activity relationship (SAR, Figure 11) emerging from this series is informative for future design. Substituents at the benzofuran C2 position were generally well tolerated and enhanced EPAC1 activity. Installation of arylketone groups bearing methoxy substituents in the ortho-position yielded many of the most potent agonists (e.g., **DM243** and **DM244**), suggesting that this motif is favorably accommodated in the EPAC1 binding site in the active conformation. Concurrently, minor changes in substitution patterns influenced isoform selectivity; for example, **DM243**, bearing a 2-methoxyphenyl terminal ring, achieved full EPAC1 selectivity, whereas **DM244** (bearing 2,4-*di*-methoxyphenyl) caused a small degree of EPAC2 activation. This implies that subtle stereoelectronic differences at the binding interfaces can shift the EPAC1/EPAC2 balance, highlighting the sensitivity of the isoform-specific pocket and guiding future design. Other terminal ring substituents, including 2-methyl (**DM240**), 3-methyl (**DM239**), 2,4-*di*-methyl (**DM242**), 2-chloro (**DM241**), and 3-hydroxyl (**DM357**), gave rise to less potent EPAC1 activation below that previously reported for **SY009**. Interestingly, a 2-naphthyl system (**DM245**) did allow for efficient EPAC1 activation. Notably, our attempt to find an alternative for the benzofuranyl ketone moiety was not successful, with the benzofuropyridine analogue **DM312** failing to achieve meaningful EPAC activation.

**Figure 11:**
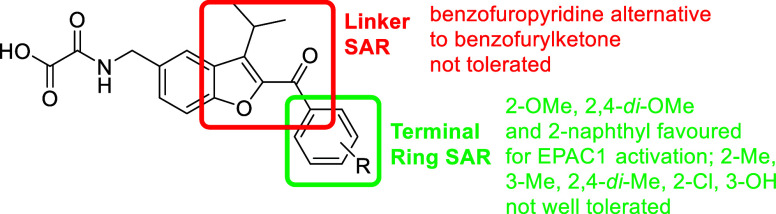
SAR for new compounds.

It is notable that several compounds predicted to bind to the EPAC1 binding site more effectively than **SY009** (e.g., **DM240** and **DM312**) were, in fact, shown to activate EPAC1 to a lesser degree, while one compound predicted to bind with lower affinity than **SY009** and **DM243**, proved to be a potent EPAC1 activator. This underlines the limitations of simple computational docking studies for enzyme activation: in order to activate EPAC1, a compound must bind to the inactive protein conformation and then induce the activating conformational change. While docking against the active conformation can provide a guide to compound design, this is not definitive. For a more accurate prediction of EPAC activator efficacy, higher level computational modeling taking into account the dynamic protein structure must be used.

The synthetic route itself imposes practical constraints on optimization. The multistep sequence, culminating in the harsh LiAlH_4_ reduction, resulted in low overall yields and limited the quantities of each analogue available for testing. This bottleneck restricted the scope of our SAR exploration and slowed the iterative design–make–test cycle. Future efforts will focus on streamlining the synthesis: for example, employing milder cyclization or coupling methods could enable late-stage introduction of diverse substituents. Alternative reductants or protecting-group strategies might permit access to currently inaccessible C3-substituted analogues. These route refinements will be crucial for increasing throughput and for incorporating polar or heterocyclic substituents (which may improve pharmacokinetic profiles) without severe yield penalties.

We note several limitations of the current study. First, the limited yields and throughput meant that only a modest library of analogues was synthesized, so our SAR conclusions are based on a relatively small data set. Second, while our assays demonstrate selective EPAC1 engagement and downstream cellular effects, we lack detailed mechanistic or structural data to explain why these compounds favor EPAC1. Co-crystal structures or advanced modeling would be valuable to reveal the key ligand–protein interactions. Third, our evaluation thus far has been limited to in vitro assays comparing EPAC1 and EPAC2; we have not yet assessed off-target profiles (e.g., other cAMP-binding proteins) or conducted in vivo efficacy and toxicity studies in relevant animal models.

In our view, three conclusions are robust. First, benzofuran oxoacetic acids engage EPAC isoforms in vitro and activate the EPAC1/Rap1 pathway in cells, with **DM243**, **DM244,** and **DM245** as the clearest exemplars. Second, these chemotypes modulate disease-relevant phenotypes, attenuating **IL-6**-driven STAT3 signaling and suppressing **TGF-β1**–induced FMT, while exhibiting substantially lower cytotoxicity than **nintedanib** at efficacious concentrations. Third, the assay-to-assay discrepancies, rather than undermining the approach, map the key levers to engineer: exposure, cellular context, and signaling bias. We therefore regard benzofuran oxoacetic acids as chemically pliable EPAC-pathway probes with a promising safety window and clear room for potency gain. With improved synthetic access, prodrug design, and rigorous linkage of exposure to mechanism, this scaffold could deliver next-generation EPAC1-biased agonists suitable for testing in fibrosis and inflammation models.

## Experimental Section

### General

Cell cultures include normal lung fibroblasts obtained from Lonza (CC512) and *Escherichia coli* One Shot BL21 Star (DE3) sourced from Invitrogen. The study employs LB Medium from Thermofisher Scientific, antibiotics, ampicillin, and kanamycin, which were from Sigma-Aldrich, FGM-2 and FBM media from Lonza, and laboratory reagents such as BSA, Ascorbic Acid, IPTG, and Triton-X-100 from Sigma-Aldrich. Immunoblotting and other assays use primary and secondary antibodies from Cell Signaling Technology, with Hoechst 33342 from Thermo Fisher Scientific for DNA staining, and an 8-NBD-cAMP fluorescent probe from Biolog Life Science Institute for fluorescence-based assays. Unlabeled ligands, including cAMP and various test molecules, as well as DMSO for solvent purposes, are also obtained from Sigma-Aldrich. The study’s key equipment includes a rotary incubator, NanoDrop 2000/2000c from Thermo Fisher Scientific, InCell Analyzer 2200 and Columbus software from GE Healthcare, and automated liquid handlers such as Biomek from Beckman Coulter, Echo 650 from LabCyte, Multidrop from Thermo Fisher Scientific, and Viaflo from Integra Biosciences. The FLUOstar Omega microplate reader from BMG LABTECH is crucial for measuring fluorescence intensity, with assays conducted in black, 96-well plates from Corning. GraphPad Prism 8 software from GraphPad Software facilitates data analysis and curve fitting. The protein purification process leverages Glutathione Sepharose 4B beads from GE Healthcare and various buffers from Sigma-Aldrich and Thermo Fisher Scientific, with centrifugation and sonication equipment from Eppendorf, and Slide-A-Lyzer Dialysis Cassettes from Thermo Fisher Scientific for protein dialysis.

All nonaqueous reactions were carried out under oxygen-free N2 using flame-dried glassware. THF, CH_2_Cl_2_, MeCN, and PhMe were purified by the MBRAUN SPS-800 solvent purification system. Before use, Grignard reagents were titrated against menthol using 2,2-bipyridine as an indicator. Petroleum ether refers to the fraction of petroleum ether boiling in the range 40–60 °C and was purchased in Winchester quantities. Brine refers to a saturated solution. Water is distilled water.

Flash column chromatography was carried out using Matrix silica gel 60 from Fisher Chemicals or Fluorochem. Thin layer chromatography was carried out using commercially available Merck F254 aluminum-backed silica plates, visualized by UV (254 nm) or stained using aqueous acidic KMnO_4_. Amines were visualized using a ninhydrin stain. Proton (300 or 400 MHz) and carbon (75.5 or 101 MHz) NMR spectra were recorded on Bruker AV 300, AV 400, or AVIII HD. Fluorine NMR spectra (282 MHz) were recorded on Bruker AV 300 or AVIII HD. For samples recorded in CDCl_3_, DMSO-*d*_6_, and acetone-*d*_6_ or CD_3_OD, chemical shifts are quoted on parts per million relative to CHCl_3_ (δH 7.26), DMSO-*d*_6_ (δH 3.50, central line of quintet), acetone-*d*_6_ (δH 2.05, central line of quintet) or CD_3_OD (δH 3.31, central line of quintet) and CDCl_3_ (δC 77.0, central line of triplet), acetone-*d*_6_ (δC 29.8, central line of septet), DMSO-*d*_6_ (δC 39.5, central line of septet), and CD_3_OD (δC 49.0, central line of septet). Carbon NMR spectra were recorded with broadband decoupling and assigned using DEPT experiments. Coupling constants (*J*) are quoted in Hertz. Melting points were obtained on a Stuart Scientific SMP 10 at ambient pressure. Infrared spectra were recorded on a PerkinElmer Spectrum 100 FT-IR Universal ATR Sampling Accessory, deposited neat to a diamond/ZnSe plate. Mass spectra were obtained at the EPSRC UK National Mass Spectrometry Facility at Swansea University and SIRCAMS at the University of Edinburgh. Purity of compounds was verified using HPLC analysis, run on a Shimadzu HPLC system (model: Nexera-*I* LC-2040C 3D Plus). HPLC analysis conditions: Shimadzu Shim-pack GISS C18 (150 × 4.6 mm), flow rate 1.0 mL/min, UV detection at 254 nm, linear gradient from 10% acetonitrile in water (0.1% TFA) to 50% acetonitrile over 3 min, followed by 6 min at 50% acetonitrile, then 50% to 100% acetonitrile over 3 min. All biologically evaluated compounds are >95% pure.

### Protein Expression and Purification

For generating *E. coli* stock plates containing protein expression, 5 mL of LB medium with 100 μg/mL ampicillin or 50 μg/mL kanamycin was inoculated using a bacterial glycerol stock, incubated at 37 °C for 16 h at 200 rpm. The next day, 200 μL and 50 μL of the culture were plated on selective agar plates with the same antibiotics and incubated overnight at 37 °C. Single colonies were picked to inoculate 5 mL of selective LB broth, incubated at 37 °C and 300 rpm for 8 h. These cultures were then diluted 1:100 into 100 mL of fresh selective LB medium and incubated for 16 h under the same conditions. The bacteria were harvested by centrifugation at 6000*g* for 15 min at 4 °C. Plasmid DNA isolation followed using a Qiagen Plasmid Maxi Kit, with the isolated DNA dissolved in 250 μL of TE Buffer, pH 8.0, and its concentration adjusted to 1 mg/mL. The plasmid solutions were stored at −20 °C. Recombinant proteins, including GST-fusion proteins of EPAC1 and EPAC2 cyclic nucleotide-binding domains (CNBDs) and RalGDS-RBD, were produced in *E. coli* using pGEX series vectors, transformed into *E. coli* BL21 Star (DE3), and plated on selective LB agar with ampicillin. Following overnight incubation, stock plates were stored at 4 °C. For protein expression, bacterial colonies were used to inoculate LB medium with ampicillin, incubated overnight at 37 °C and 200 rpm, diluted 1:20 in fresh medium, and induced with IPTG for overnight growth at 19 °C. Cells were harvested, washed, and lysed for protein purification using Glutathione Sepharose 4B beads, followed by elution and dialysis. The purified proteins were analyzed, quantified, aliquoted, snap-frozen, and stored at −80 °C for later use.

### Computational Docking Modeling

A homology model of EPAC1 in the active conformation was prepared using the SWISS-MODEL Web server.^31,32^ An X-ray crystallographic structure of EPAC2-cAMP complex (PDB accession code 4MGK) was used as a template. The EPAC1 homology model does not feature residues 1–218 as the equivalent residues are absent from the EPAC2 template. This region corresponds to the *N*-terminal DEP domain, which is not implicated in the binding of cAMP or our ligands to EPAC1. The EPAC1 homology model PDB file was converted to the AutoDock PDBQT file format using AutoDockTools v1.5.6 (ADT). Gasteiger charges were computed, and nonpolar hydrogen atoms were merged. Key residues within the cNBD were modeled with flexible side chains (F274, V291, T502, L304, F310, L313, R321, I325, M354, R355, L356, and E357). A separate PDBQT file was prepared for the flexible side chain atoms using ADT. For EPAC2 docking studies, an X-ray crystallographic structure of an EPAC2-cAMP complex (PDB accession code 3CF6) was utilized for the docking of ligands against EPAC2. The 3CF6 crystal structure contains *Mus musculus* EPAC2, though this is adequately similar to human EPAC2 for use in docking calculations (100% sequence homology within 10 Å of bound cAMP). The downloaded PDB structure was converted to AutoDock PDBQT format using ADT in the same way as the EPAC1 homology model, taking care to first delete crystallographic cAMP and water molecules. The side chains for the residues at the same loci as those listed for EPAC1 were modeled as flexible (F367, V384, C395, L397, F403, L406, R414, I418, V447, R448, L449, and K450).

SMILES strings for each ligand were generated using an online tool,^33^ and these were subsequently converted to ligand PDB files using BioSimSpace.^34^ Ligands were modeled as neutral. The ligand PDB files were converted to PDBQT format using ADT. All rotatable bonds were modeled as such, except for primary amide bonds, which were restricted to the lowest energy conformation of a 180 ° torsion angle between the amide O and H atoms.

Protein–ligand docking modeling was carried out with AutoDock Vina v1.2.3, using the default Vina scoring function.^35^ The search space was restricted to a 40 Å cube centered on the cNBD. The largest allowable energy difference between the best and worst ranked binding pose was 8 kcal/mol. Each calculation generated 100 docked poses. The output PDBQT file was converted to a PDB format using OpenBabel v3.1.1 and visualized in PyMOL v2.5.2.^36^

#### 2-Bromo-1-(*m*-tolyl)­ethan-1-one (**1a**)

A solution of 1-(*m*-tolyl)­ethan-1-one (2.47 mL, 14.9 mmol), NBS (3.98 g, 22.4 mmol), and *p*-TsOH (2.83 g, 14.9 mmol) in MeCN (10 mL) was stirred and heated at 60 °C for 16 h. The solution was evaporated under reduced pressure, then NaHCO_3(aq)_ was added, and the organics were extracted with CH_2_Cl_2_ (×3). The organic layers were combined, dried (MgSO_4_), and evaporated under reduced pressure to give the crude product. Purification by flash column chromatography on silica with petroleum ether/EtOAc (99:1) as eluent gave product **1a** as a colorless oil (2.45 g, 77%); *R*_F_ 0.2 (petroleum ether/EtOAc 99:1); IR (film) 2967, 1679 (C=O str), 1600, 1569, 1455, 1382, 1290, 1259, 1207, 1185, 1006, 978, 735 cm^–1^; ^1^H NMR (300 MHz, CDCl_3_): δ 7.70–7.65 (m, 1H, Ar), 7.43 (td, *J* = 7.5, 1.5 Hz, 1H, Ar), 7.33–7.27 (m, 2H, Ar), 4.42 (s, 2H, C*H*_2_Br), 2.53 (s, 3H, C*H*_3_); ^13^C NMR (75.5 MHz, CDCl_3_): δ 194.3 (C), 139.9 (C), 134.6 (C), 132.5 (CH), 132.5 (CH), 129.1 (CH), 125.9 (CH), 33.8 (CH_2_), 21.6 (CH_3_). Spectroscopic data are consistent with those reported in the literature.^37^

#### 2-Bromo-1-(*o*-tolyl)­ethan-1-one (**1b**)

A solution of 1-(*o*-tolyl)­ethan-1-one (1.50 g, 11.1 mmol), NBS (2.59 g, 19.4 mmol), and *p*-TsOH (2.13 g, 11.1 mmol) in MeCN (10 mL) was stirred and heated at 60 °C for 16 h. The solution was evaporated under reduced pressure, then NaHCO_3(aq)_ was added, and the organics were extracted with CH_2_Cl_2_ (×3). The organic layers were combined, dried (MgSO_4_), and evaporated under reduced pressure to give the crude product. Purification by flash column chromatography on silica with petroleum ether/EtOAc (99:1) as eluent gave product **1b** as a colorless oil (1.69 g, 72%); *R*_F_ 0.2 (petroleum ether/EtOAc 99:1); IR (film) 2967, 1679 (C=O str), 1600, 1569, 1455, 1382, 1290, 1259, 1207, 1185, 1006, 978, 735 cm^–1^; ^1^H NMR (300 MHz, CDCl_3_): δ 7.70–7.65 (m, 1H, Ar), 7.43 (td, *J* = 7.5, 1.5 Hz, 1H, Ar), 7.33–7.27 (m, 2H, Ar), 4.42 (s, 2H, CH_2_Br), 2.53 (s, 3H, CH_3_); ^13^C NMR (75.5 MHz, CDCl_3_): δ 194.3 (C), 139.9 (C), 134.6 (C), 132.5 (CH), 132.5 (CH), 129.1 (CH), 125.9 (CH), 33.8 (CH_2_), 21.6 (CH_3_). Spectroscopic data are consistent with those reported in the literature.^37^

#### 2-Bromo-1-(2-chlorophenyl)­ethan-1-one (**1c**)

A solution of 1-(2-chlorophenyl)­ethan-1-one (1.50 g, 9.7 mmol), NBS (2.25 g, 12.6 mmol), and *p*-TsOH (1.85 g, 9.7 mmol) in MeCN (10 mL) was stirred and heated at 60 °C for 16 h. The solution was evaporated under reduced pressure, then NaHCO_3(aq)_ was added, and the organics were extracted with CH_2_Cl_2_ (×3). The organic layers were combined, dried (MgSO_4_), and evaporated under reduced pressure to give the crude product. Purification by flash column chromatography on silica with petroleum ether/EtOAc (99:1) as eluent gave product **1c** as a colorless oil (1.72 g, 76%); *R*_F_ 0.2 (petroleum ether/EtOAc 99:1); IR (film) 2937, 1695 (C=O str), 1589, 1469, 1433, 1386, 1285, 1253, 1193, 1119, 1065, 988 cm^–1^; ^1^H NMR (300 MHz, CDCl_3_): δ 7.59–7.55 (m, 1H, Ar), 7.47–7.44 (m, 2H, Ar), 7.40–7.33 (m, 1H, Ar), 4.52 (s, 2H, CH_2_); ^13^C NMR (75.5 MHz, CDCl_3_): δ 194.2 (C), 136.4 (C), 132.9 (CH), 131.4 (C), 130.7 (CH), 130.4 (CH), 127.3 (CH), 34.6 (CH_2_). Spectroscopic data are consistent with those reported in the literature.^37^

#### 2-Bromo-1-(2,4-dimethylphenyl)­ethan-1-one (**1d**)

A solution of 1-(2,4-dimethylphenyl)­ethan-1-one (1.50 g, 10.1 mmol), NBS (2.34 g, 13.2 mmol), and *p*-TsOH (1.93 g, 10.1 mmol) in MeCN (10 mL) was stirred and heated at 60 °C for 16 h. The solution was evaporated under reduced pressure, then NaHCO_3(aq)_ was added, and the organics were extracted with CH_2_Cl_2_ (×3). The organic layers were combined, dried (MgSO_4_), and evaporated under reduced pressure to give the crude product. Purification by flash column chromatography on silica with petroleum ether/EtOAc (99:1) as eluent gave product **1d** as a colorless oil (1.68 g, 73%); R_F_ 0.2 (petroleum ether/EtOAc 99:1); IR (film) 2970, 2922, 1684 (C=O str), 1608, 1562, 1495, 1439, 1381, 1291, 1198, 1134, 1028, 983, 805 cm^–1^; ^1^H NMR (300 MHz, CDCl_3_): δ 7.61 (d, *J* = 8.0 Hz, 1H, Ar), 7.12–7.07 (m, 2H, Ar), 4.41 (s, 2H, CH_2_), 2.52 (s, 3H, CH_3_), 2.37 (s, 3H, CH_3_); ^13^C NMR (75.5 MHz, CDCl_3_): δ 193.6 (C), 143.4 (C), 140.5 (C), 133.4 (CH), 131.5 (C), 129.8 (CH), 126.6 (CH), 33.7 (CH_2_), 21.8 (CH_3_), 21.6 (CH_3_). Spectroscopic data are consistent with those reported in the literature.^38^

#### 2-Bromo-1-(2,4-dimethoxyphenyl)­ethan-1-one (**1e**)

A solution of 1-(2,4-dimethoxyphenyl)­ethan-1-one (1.00 g, 5.5 mmol) in CHCl_3_ (80 mL) was added to a stirred suspension of CuBr_2_ (2.60 g, 11.6 mmol) in EtOAc (65 mL) at reflux. The solution was stirred at reflux for a further 2 h. The resulting mixture was filtered and evaporated under reduced pressure. To the residue, EtOAc (40 mL) and NaHCO_3_ (40 mL) were added, and the layers were separated. The organic layer was washed with NaHCO_3_ (40 mL) and brine (40 mL), dried (MgSO_4_), and evaporated under reduced pressure to give the crude product. Purification by flash column chromatography on silica with petroleum ether/EtOAc (90:10) as eluent gave product **1e** as a violet solid (1.39 g, 98%); *R*_F_ 0.2 (petroleum ether/EtOAc 90:10); mp 99–101 °C; IR (solid) 3012, 2947, 2842, 1661 (C=O str), 1595, 1574, 1456, 1334, 1272, 1251, 1211, 1173, 1126, 1112, 1020, 989, 828 cm^–1^; ^1^H NMR (400 MHz, CDCl_3_): δ 7.90 (d, *J* = 9.0 Hz, 1H, Ar), 6.56 (dd, *J* = 9.0, 2.0 Hz, 1H, Ar), 6.46 (d, *J* = 2.0 Hz, 1H, Ar), 4.57 (s, 2H, CH_2_), 3.92 (s, 3H, CH_3_), 3.86 (s, 3H, CH_3_); ^13^C NMR (101 MHz, CDCl_3_): δ 190.3 (C), 165.5 (C), 161.0 (C), 133.9 (CH), 118.0 (C), 106.0 (CH), 98.4 (CH), 55.8 (CH_3_), 55.8 (CH_3_), 38.1 (CH_2_); Spectroscopic data are consistent with those reported in the literature.^39^

#### 1-(5-Bromo-2-hydroxyphenyl)-2-methylpropan-1-one (**2**)

*iso*-Propylmagnesium bromide (2.9 M in 2-MeTHF, 7.76 mL, 22.5 mmol) was added dropwise to a stirred solution of 5-bromo-2-hydroxybenzonitrile (1.49 g, 7.5 mmol) in THF (10 mL) at 0 °C. The resulting solution was allowed to warm to rt and stirred for a further 2 h. The solution was then cooled to 0 °C, and water (1.5 mL) and 6 M HCl (2 mL) were added. Then, the mixture was stirred and heated at reflux for 1 h before being allowed to cool to rt. EtOAc (40 mL) and water (10 mL) were added, and the layers were separated. The organic layer was washed with water (10 mL) and brine (20 mL), dried (MgSO4), and evaporated under reduced pressure to give the crude product. Purification by flash column chromatography on silica with petroleum ether/EtOAc (90:10) as eluent gave ketone **2** as a yellow oil (1.66 g, 91%); *R*_F_ 0.6 (petroleum ether/EtOAc 90:10); IR (film) 2973, 2935, 2873, 1639 (C=O str), 1606, 1568, 1466, 1405, 1385, 1361, 1338, 1287, 1266, 1236, 1191, 1159, 1100, 1080, 984, 875, 825 cm^–1^; ^1^H NMR (300 MHz, CDCl_3_): δ 12.45 (s, 1H, OH), 7.92 (d, *J* = 2.5 Hz, 1H, Ar), 7.57 (dd, *J* = 9.0, 2.5 Hz, 1H, Ar), 6.94 (d, *J* = 9.0 Hz, 1H, Ar), 3.57 (sept, *J* = 7.0 Hz, 1H, CH), 1.29 (d, *J* = 7.0 Hz, 6H, CH*Me*_2_); ^13^C NMR (75.5 MHz, CDCl_3_): δ 209.9 (C), 162.1 (C), 138.9 (CH), 132.1 (CH), 120.8 (CH), 119.4 (C), 110.4 (C), 35.1 (CH), 19.2 (CH_3_). Spectroscopic data consistent with those reported in the literature.^40^

#### (5-Bromo-3-isopropylbenzofuran-2-yl)­(*m*-tolyl)­methanone (**3a**)

A solution of 1-(5-bromo-2-hydroxyphenyl)­propan-1-one **2** (1.22 g, 5.0 mmol), 2-bromo-1-(*m*-tolyl)­ethan-1-one **1a** (1.28 g, 6.0 mmol), and K_2_CO_3_ (1.38 g, 10 mmol) in DMF (15 mL) was stirred and heated at 60 °C for 2 h. The resulting solution was allowed to cool to rt then EtOAc and water were added, and the layers were separated. The organic layer was washed with water and brine, dried (MgSO_4_), and evaporated under reduced pressure. Toluene (15 mL) and *p*-TsOH (95 mg, 0.5 mmol) were added to the residue, and the resulting solution was stirred and heated at reflux under a Dean–Stark trap for 16 h. The resulting solution was allowed to cool to rt, then CH_2_Cl_2_ was added, and the solution was washed with saturated NaHCO_3(aq)_ (×2) and brine, dried (MgSO_4_), and evaporated under reduced pressure to give the crude product. Purification by flash column chromatography on silica with petroleum ether/EtOAc (97:3) as eluent gave product **3a** as a yellow oil (1.59 g, 89%); *R*_F_ 0.2 (petroleum ether/EtOAc 97:3); IR (film) 2967, 2928, 2870, 2360, 2341, 1646 (C=O str), 1553, 1303, 1265, 974 cm^–1^; ^1^H NMR (300 MHz, CDCl_3_): δ 8.02 (d, *J* = 2.0 Hz, 1H, Ar), 7.82–7.76 (m, 2H, Ar), 7.54 (dd, *J* = 9.0, 2.0 Hz, 1H, Ar), 7.46–7.36 (m, 3H, Ar), 3.90 (sept, *J* = 7.0 Hz, 1H, CH), 2.45 (s, 3H, CMe), 1.46 (d, *J* = 7.0 Hz, 6H, CHMe_2_); ^13^C NMR (75.5 MHz, CDCl_3_): δ 186.7 (C), 153.4 (C), 147.9 (C), 138.3 (C), 137.9 (C), 135.0 (C), 133.9 (CH), 130.6 (CH), 130.3 (CH), 129.2 (C), 128.3 (CH), 127.2 (CH), 125.8 (CH), 116.2 (C), 114.2 (CH), 25.5 (CH), 22.2 (CH_3_), 21.6 (CH_3_).

#### (5-Bromo-3-isopropylbenzofuran-2-yl)­(*o*-tolyl)­methanone (**3b**)

A solution of 1-(5-bromo-2-hydroxyphenyl)­propan-1-one **2** (1.22 g, 5.0 mmol), 2-bromo-1-(*o*-tolyl)­ethan-1-one **1b** (1.28 g, 6.0 mmol), and K_2_CO_3_ (1.38 g, 10 mmol) in DMF (15 mL) was stirred and heated at 60 °C for 2 h. The resulting solution was allowed to cool to rt then EtOAc and water were added, and the layers were separated. The organic layer was washed with water and brine, dried (MgSO_4_), and evaporated under reduced pressure. Toluene (15 mL) and *p*-TsOH (95 mg, 0.5 mmol) were added to the residue, and the resulting solution was stirred and heated at reflux under a Dean–Stark trap for 16 h. The resulting solution was allowed to cool to rt, then CH_2_Cl_2_ was added, and the solution was washed with saturated NaHCO_3(aq)_ (×2) and brine, dried (MgSO_4_), and evaporated under reduced pressure to give the crude product. Purification by flash column chromatography on silica with petroleum ether/EtOAc (97:3) as eluent gave product **3b** as a yellow oil (1.67 g, 94%); *R*_F_ 0.2 (petroleum ether/EtOAc 97:3); IR (film) 2967, 2929, 2871, 2360, 2341, 1652 (C=O str), 1553, 1302, 1258, 964 cm^–1^; ^1^H NMR (300 MHz, CDCl_3_): δ 8.00 (d, *J* = 2.0 Hz, 1H, Ar), 7.52 (dd, *J* = 9.0, 2.0 Hz, 1H, Ar), 7.49–7.39 (m, 2H, Ar), 7.35 (d, *J* = 9.0 Hz, 1H, Ar), 7.30 (d, *J* = 7.5 Hz, 2H, Ar), 3.82 (sept, *J* = 7.0 Hz, 1H, C*H*(CH_3_)_2_), 2.37 (s, 3H, CC*H*_3_), 1.44 (d, *J* = 7.0 Hz, 6H, CH­(C*H*_3_)_2_); ^13^C NMR (75.5 MHz, CDCl_3_): δ 189.4 (C), 153.6 (C), 148.0 (C), 138.6 (C), 137.2 (C), 135.2 (C), 131.2 (CH), 131.1 (CH), 130.9 (CH), 129.3 (C), 128.8 (CH), 126.0 (CH), 125.6 (CH), 116.2 (C), 114.3 (CH), 25.4 (CH), 22.2 (CH_3_), 19.9 (CH_3_).

#### (5-Bromo-3-isopropylbenzofuran-2-yl)­(2-chlorophenyl)­methanone (**3c**)

A solution of 1-(5-bromo-2-hydroxyphenyl)­propan-1-one **2** (1.22 g, 5.0 mmol), 2-bromo-1-(2-chlorophenyl)­ethan-1-one **1c** (1.40 g, 6.0 mmol), and K_2_CO_3_ (1.38 g, 10 mmol) in DMF (15 mL) was stirred and heated at 60 °C for 2 h. The resulting solution was allowed to cool to rt then EtOAc and water were added, and the layers were separated. The organic layer was washed with water and brine, dried (MgSO_4_), and evaporated under reduced pressure. Toluene (15 mL) and *p*-TsOH (95 mg, 0.5 mmol) were added to the residue, and the resulting solution was stirred and heated at reflux under a Dean–Stark trap for 16 h. The resulting solution was allowed to cool to rt, then CH_2_Cl_2_ was added, and the solution was washed with saturated NaHCO_3(aq)_ (×2) and brine, dried (MgSO_4_), and evaporated under reduced pressure to give the crude product. Purification by flash column chromatography on silica with petroleum ether/EtOAc (98:2) as eluent gave product **3c** as a yellow solid (1.79 g, 95%); *R*_F_ 0.2 (petroleum ether/EtOAc 98:2); mp 118–120 °C; IR (solid) 2973, 2865, 2359, 1656 (C=O str), 1561, 1464, 1434, 1355, 1304, 1276, 1249, 1223, 1073, 965, 885 cm^–1^; ^1^H NMR (300 MHz, CDCl_3_): δ 8.01 (d, *J* = 2.0 Hz, 1H, Ar), 7.53 (dd, *J* = 9.0, 2.0 Hz, 1H, Ar), 7.50–7.36 (m, 4H, Ar), 7.33 (d, *J* = 9.0 Hz, 1H, Ar), 3.91 (sept, *J* = 7.0 Hz, 1H, C*H*(CH_3_)_2_), 1.46 (d, *J* = 7.0 Hz, 6H, CH­(C*H*_3_)_2_); ^13^C NMR (75.5 MHz, CDCl_3_): δ 186.4 (C), 153.8 (C), 147.4 (C), 138.7 (C), 135.9 (C), 131.9 (C), 131.9 (CH), 131.2 (CH), 130.2 (CH), 129.4 (CH), 129.3 (C), 126.9 (CH), 126.1 (CH), 116.3 (C), 114.4 (CH), 25.4 (CH), 22.1 (CH_3_).

#### (5-Bromo-3-isopropylbenzofuran-2-yl)­(2,4-dimethylphenyl)­methanone (**3d**)

A solution of 1-(5-bromo-2-hydroxyphenyl)­propan-1-one **2** (1.18 g, 4.86 mmol), 2-bromo-1-(2,4-dimethylphenyl)­ethan-1-one **1d** (1.33 g, 5.83 mmol), and K_2_CO_3_ (1.34 g, 9.72 mmol) in DMF (15 mL) was stirred and heated at 60 °C for 2 h. The resulting solution was allowed to cool to rt then EtOAc and water were added, and the layers were separated. The organic layer was washed with water and brine, dried (MgSO_4_), and evaporated under reduced pressure. Toluene (15 mL) and *p*-TsOH (93 mg, 0.49 mmol) were added to the residue, and the resulting solution was stirred and heated at reflux under a Dean–Stark trap for 16 h. The resulting solution was allowed to cool to rt, then CH_2_Cl_2_ was added, and the solution was washed with saturated NaHCO_3(aq)_ (×2) and brine, dried (MgSO_4_), and evaporated under reduced pressure to give the crude product. Purification by flash column chromatography on silica with petroleum ether/EtOAc (98:2) as eluent gave product **3d** as a yellow oil (1.42 g, 79%); *R*_F_ 0.2 (petroleum ether/EtOAc 98:2); IR (film) 2966, 2928, 2360, 2341, 1651 (C=O str), 1553, 1303, 1259, 1059, 967, 803 cm^–1^; ^1^H NMR (300 MHz, CDCl_3_): δ 8.00 (dd, *J* = 2.0, 0.5 Hz, 1H, Ar), 7.51 (dd, *J* = 9.0, 2.0 Hz, 1H, Ar), 7.39 (d, *J* = 8.0 Hz, 1H, Ar), 7.35 (dd, *J* = 9.0, 0.5 Hz, 1H, Ar), 7.14–7.07 (m, 2H, Ar), 3.83 (sept, *J* = 7.0 Hz, 1H, C*H*(CH_3_)_2_), 2.40 (s, 3H, CC*H*_3_), 2.37 (s, 3H, CCH_3_), 1.44 (d, *J* = 7.0 Hz, 6H, CH­(C*H*_3_)_2_); ^13^C NMR (75.5 MHz, CDCl_3_): δ 189.2 (C), 153.5 (C), 148.3 (C), 141.7 (C), 137.7 (C), 135.7 (C), 134.7 (C), 132.2 (CH), 130.7 (CH), 129.5 (CH), 129.3 (C), 126.2 (CH), 125.9 (CH), 116.1 (C), 114.3 (CH), 25.4 (CH), 22.2 (CH_3_), 21.7 (CH_3_), 20.0 (CH_3_).

#### (5-Bromo-3-isopropylbenzofuran-2-yl)­(2,4-dimethoxyphenyl)­methanone (**3e**)

A solution of 1-(5-bromo-2-hydroxyphenyl)­propan-1-one **2** (977 mg, 4.02 mmol), 2-bromo-1-(2,4-dimethoxyphenyl)­ethan-1-one **1d** (1.25 g, 4.82 mmol), and K_2_CO_3_ (1.11 g, 8.04 mmol) in DMF (15 mL) was stirred and heated at 60 °C for 2 h. The resulting solution was allowed to cool to rt then EtOAc and water were added, and the layers were separated. The organic layer was washed with water and brine, dried (MgSO_4_), and evaporated under reduced pressure. Toluene (15 mL) and *p*-TsOH (76 mg, 0.40 mmol) were added to the residue, and the resulting solution was stirred and heated at reflux under a Dean–Stark trap for 16 h. The resulting solution was allowed to cool to rt, then CH_2_Cl_2_ was added, and the solution was washed with saturated NaHCO_3(aq)_ (×2) and brine, dried (MgSO_4_), and evaporated under reduced pressure to give the crude product. Purification by flash column chromatography on silica with petroleum ether/EtOAc (95:5) as eluent gave product **3e** as a yellow solid (1.26 g, 74%); *R*_F_ 0.2 (petroleum ether/EtOAc 95:5); mp 95–98 °C; IR (solid) 2965, 2871, 2360, 2341, 1641 (C=O str), 1599, 1274, 1208, 966, 818 cm^–1^; ^1^H NMR (300 MHz, CDCl_3_): δ 7.97 (d, *J* = 2.0 Hz, 1H, Ar), 7.51 (d, *J* = 8.5 Hz, 1H, Ar), 7.48 (dd, *J* = 9.0, 2.0 Hz, 1H, Ar), 7.32 (d, *J* = 9.0 Hz, 1H, Ar), 6.58 (dd, *J* = 8.5, 2.5 Hz, 1H, Ar), 6.51 (d, *J* = 2.5 Hz, 1H), 3.88 (s, 3H, OC*H*_3_), 3.81 (hept, *J* = 7.0 Hz, 1H, C*H*(CH_3_)_2_), 3.71 (s, 3H, OC*H*_3_), 1.43 (d, *J* = 7.0 Hz, 6H, CH­(C*H*_3_)_2_); ^13^C NMR (75.5 MHz, CDCl_3_): δ 186.0 (C), 164.2 (C), 160.3 (C), 153.3 (C), 149.1 (C), 132.8 (C), 132.3 (CH), 130.1 (CH), 129.5 (C), 125.7 (CH), 122.0 (C), 115.8 (C), 113.9 (CH), 105.0 (CH), 99.0 (CH), 56.0 (CH_3_), 55.7 (CH_3_), 25.2 (CH), 22.2 (CH_3_).

#### (5-Bromo-3-isopropylbenzofuran-2-yl)­(2-methoxyphenyl)­methanone (**3f**)

A solution of 1-(5-bromo-2-hydroxyphenyl)­propan-1-one **2** (1.22 g, 5.0 mmol), commercially available 2-bromo-1-(2-methoxyphenyl)­ethan-1-one **1f** (1.37 g, 6.0 mmol) and K_2_CO_3_ (1.38 g, 10 mmol) in DMF (15 mL) was stirred and heated at 60 °C for 2 h. The resulting solution was allowed to cool to rt then EtOAc and water were added, and the layers were separated. The organic layer was washed with water and brine, dried (MgSO_4_), and evaporated under reduced pressure. Toluene (15 mL) and *p*-TsOH (95 mg, 0.5 mmol) were added to the residue, and the resulting solution was stirred and heated at reflux under a Dean–Stark trap for 16 h. The resulting solution was allowed to cool to rt, then CH_2_Cl_2_ was added, and the solution was washed with saturated NaHCO_3(aq)_ (×2) and brine, dried (MgSO_4_), and evaporated under reduced pressure to give the crude product. Purification by flash column chromatography on silica with petroleum ether/EtOAc (95:5) as eluent gave product **3f** as a yellow oil (1.84 g, 98%); *R*_F_ 0.2 (petroleum ether/EtOAc 95:5); IR (film) 2966, 2360, 2341, 1652 (C=O str), 1598, 1556, 1464, 1434, 1356, 1303, 1246, 965, 753 cm^–1^; ^1^H NMR (300 MHz, CDCl_3_): δ 8.02 (d, *J* = 2.0 Hz, 1H, Ar), 7.59–7.47 (m, 3H, Ar), 7.35 (d, *J* = 9.0 Hz, 1H, Ar), 7.10 (td, *J* = 7.5, 1.0 Hz, 1H, Ar), 7.03 (d, *J* = 8.5 Hz, 1H, Ar), 3.88 (sept, *J* = 7.0 Hz, 1H, C*H*(CH_3_)_2_), 3.77 (s, 3H, OC*H*_3_), 1.47 (d, *J* = 7.0 Hz, 6H, CH­(C*H*_3_)_2_); ^13^C NMR (75.5 MHz, CDCl_3_): δ 187.3 (C), 158.0 (C), 153.5 (C), 148.5 (C), 133.9 (C), 132.8 (CH), 130.5 (CH), 129.7 (CH), 129.4 (C), 129.1 (C), 125.9 (CH), 120.7 (CH), 115.9 (C), 114.1 (CH), 111.7 (CH), 55.9 (CH_3_), 25.2 (CH), 22.2 (CH_3_).

#### (5-Bromo-3-isopropylbenzofuran-2-yl)­(naphthalen-2-yl)­methanone (**3g**)

A solution of 1-(5-bromo-2-hydroxyphenyl)­propan-1-one **2** (1.22 g, 5.0 mmol), commercially available 2-bromo-1-(naphthalen-2-yl)­ethan-1-one **1g** (1.49 g, 6.0 mmol), and K_2_CO_3_ (1.38 g, 10 mmol) in DMF (15 mL) was stirred and heated at 60 °C for 2 h. The resulting solution was allowed to cool to rt then EtOAc and water were added, and the layers were separated. The organic layer was washed with water and brine, dried (MgSO_4_), and evaporated under reduced pressure. Toluene (15 mL) and *p*-TsOH (95 mg, 0.5 mmol) were added to the residue, and the resulting solution was stirred and heated at reflux under a Dean–Stark trap for 16 h. The resulting solution was allowed to cool to rt, then CH_2_Cl_2_ was added, and the solution was washed with saturated NaHCO_3(aq)_ (×2) and brine, dried (MgSO_4_), and evaporated under reduced pressure to give the crude product. Purification by flash column chromatography on silica with petroleum ether/EtOAc (98:2) as eluent gave product **3g** as a yellow solid (1.911 g, 97%); *R*_F_ 0.2 (petroleum ether/EtOAc 98:2); mp 90–93 °C; IR (ATR) 2970, 1649 (C=O Str), 1622, 1560, 1462, 1362, 1307, 1261, 1217, 1199, 1149, 1110, 1060, 993, 942, 914, 868, 827 cm^–1^; ^1^H NMR (300 MHz, CDCl_3_): δ 8.56 (s, 1H), 8.09–7.89 (m, 5H, Ar), 7.67–7.54 (m, 3H, Ar), 7.44 (d, *J* = 9.0 Hz, 1H, Ar), 3.97 (hept, *J* = 7.0 Hz, 1H, C*H*(CH_3_)_2_), 1.49 (d, *J* = 7.0 Hz, 6H, CH­(C*H*_3_)_2_); ^13^C NMR (75.5 MHz, CDCl_3_): δ 186.3 (C), 153.5 (C), 148.1 (C), 135.6 (C), 135.2 (C), 135.2 (C), 132.5 (C), 131.9 (CH), 130.6 (CH), 129.8 (CH), 129.3, 128.7 (CH), 128.4 (CH), 128.0 (CH), 126.9 (CH), 125.9 (CH), 125.4 (CH), 116.3, 114.2 (CH), 25.6 (CH), 22.2 (CH_3_).

#### 3-Isopropyl-2-(3-methylbenzoyl)­benzofuran-5-carbonitrile (**4a**)

A solution of (5-bromo-3-isopropylbenzofuran-2-yl)­(*m*-tolyl)­methanone **3a** (1.46 g, 4.08 mmol), Zn­(CN)_2_ (575 mg, 4.89 mmol), Pd_2_dba_3_ (262 mg, 0.29 mmol), and dppf (317 mg, 0.57 mmol) in DMF (20 mL) was stirred and heated at 120 °C for 4 h. The resulting solution was filtered over Celite and washed with EtOAc. 1.8 M NH_4_OH_(aq)_ was added to the filtrate, and the layers were separated. The organic layer was washed with 1.8 M NH_4_OH_(aq)_ and brine, then dried (MgSO_4_) and evaporated under reduced pressure to give the crude product. Purification by flash column chromatography on silica with petroleum ether/EtOAc (95:5) as eluent gave product **4a** as an off-white solid (906 mg, 73%); *R*_F_ 0.2 (petroleum ether/EtOAc 95:5); mp 123–125 °C; IR (solid) 2969, 2930, 2360, 2341, 2226 (C≡N str), 1634 (C=O str), 1598, 1548, 1283, 1269, 940 cm^–1^; ^1^H NMR (300 MHz, CDCl_3_): δ 8.25 (dd, *J* = 1.5, 0.5 Hz, 1H, Ar), 7.81–7.75 (m, 2H, Ar), 7.71 (dd, *J* = 8.5, 1.5 Hz, 1H, Ar), 7.63 (dd, *J* = 8.5, 0.5 Hz, 1H, Ar), 7.48–7.37 (m, 2H, Ar), 3.91 (sept, *J* = 7.0 Hz, 1H, C*H*(CH_3_)_2_), 2.45 (s, 3H, CC*H*_3_), 1.47 (d, *J* = 7.0 Hz, 6H, CH­(C*H*_3_)_2_); ^13^C NMR (75 MHz, CDCl_3_): δ 186.4 (C), 156.0 (C), 148.6 (C), 138.4 (C), 137.5 (C), 134.8 (C), 134.2 (CH), 130.4 (CH), 130.2 (CH), 128.6 (CH), 128.4 (CH), 127.9 (C), 127.1 (CH), 119.1 (C), 114.0 (CH), 107.2 (C), 25.4 (CH), 22.2 (CH_3_), 21.5 (CH_3_).

#### 3-Isopropyl-2-(2-methylbenzoyl)­benzofuran-5-carbonitrile (**4b**)

A solution of (5-bromo-3-isopropylbenzofuran-2-yl)­(*o*-tolyl)­methanone **3b** (1.66 g, 4.65 mmol), Zn­(CN)_2_ (655 mg, 5.58 mmol), Pd_2_dba_3_ (298 mg, 0.33 mmol), and dppf (361 mg, 0.65 mmol) in DMF (20 mL) was stirred and heated at 120 °C for 4 h. The resulting solution was filtered over Celite and washed with EtOAc. 1.8 M NH_4_OH_(aq)_ was added to the filtrate, and the layers were separated. The organic layer was washed with 1.8 M NH_4_OH_(aq)_ and brine and then dried (MgSO_4_) and evaporated under reduced pressure to give the crude product. Purification by flash column chromatography on silica with petroleum ether/EtOAc (95:5) as eluent gave product **4b** as a brown oil (1.20 g, 73%); *R*_F_ 0.2 (petroleum ether/EtOAc 95:5); IR (film) 2969, 2930, 2360, 2341, 2228 (C≡N str), 1655 (C=O str), 1557, 1466, 1309, 1267, 1236, 1046, 977 cm^–1^; ^1^H NMR (300 MHz, CDCl_3_): δ 8.24 (dd, *J* = 1.5, 0.5 Hz, 1H, Ar), 7.69 (dd, *J* = 8.5, 1.5 Hz, 1H, Ar), 7.56 (dd, *J* = 8.5, 0.5 Hz, 1H, Ar), 7.50–7.41 (m, 2H, Ar), 7.35–7.27 (m, 2H, Ar), 3.85 (sept, *J* = 7.0 Hz, 1H, C*H*(CH_3_)_2_), 2.39 (s, 3H, CC*H*_3_), 1.46 (d, *J* = 7.0 Hz, 6H, CH­(C*H*_3_)_2_); ^13^C NMR (75.5 MHz, CDCl_3_): δ 189.1 (C), 156.2 (C), 148.7 (C), 138.1 (C), 137.4 (C), 135.1 (C), 131.4 (CH), 131.4 (CH), 130.7 (CH), 128.9 (CH), 128.8 (CH), 127.9 (C), 125.6 (CH), 119.1 (C), 114.1 (CH), 107.3 (C), 25.3 (CH), 22.2 (CH_3_), 19.9 (CH_3_).

#### 2-(2-Chlorobenzoyl)-3-isopropylbenzofuran-5-carbonitrile (**4c**)

A solution of (5-bromo-3-isopropylbenzofuran-2-yl)­(2-chlorophenyl)­methanone **3c** (1.61 g, 4.27 mmol), Zn­(CN)_2_ (599 mg, 5.10 mmol), Pd_2_dba_3_ (275 mg, 0.30 mmol), and dppf (333 mg, 0.60 mmol) in DMF (20 mL) was stirred and heated at 120 °C for 4 h. The resulting solution was filtered over Celite and washed with EtOAc. 1.8 M NH_4_OH_(aq)_ was added to the filtrate, and the layers were separated. The organic layer was washed with 1.8 M NH_4_OH_(aq)_ and brine, then dried (MgSO_4_), and evaporated under reduced pressure to give the crude product. Purification by flash column chromatography on silica with petroleum ether/EtOAc (95:5) as eluent gave product **4c** as a tan solid (1.167 g, 84%); *R*_F_ 0.2 (petroleum ether/EtOAc 95:5); mp 123–125 °C; IR (ATR) 2968, 2228 (C≡N str), 1672 (C=O str), 1564, 1465, 1433, 1363, 1311, 1276, 1230, 1069, 1043, 977, 905, 812 cm^–1^; ^1^H NMR (300 MHz, CDCl_3_): δ 8.25 (dd, *J* = 1.5, 0.5 Hz, 1H, Ar), 7.69 (dd, *J* = 8.5, 1.5 Hz, 1H, Ar), 7.54 (dd, *J* = 8.5, 0.5 Hz, 1H, Ar), 7.53–7.38 (m, 4H, Ar), 3.94 (hept, *J* = 7.0 Hz, 1H, C*H*(CH_3_)_2_), 1.48 (d, *J* = 7.0 Hz, 6H, CH­(C*H*_3_)_2_); ^13^C NMR (75.5 MHz, CDCl_3_): δ 186.2 (C), 156.3 (C), 148.0 (C), 138.3 (C), 135.8 (C), 132.1 (CH), 131.9 (C), 130.9 (CH), 130.2 (CH), 129.5 (CH), 128.9 (CH), 127.9 (C), 127.0 (CH), 119.0 (C), 114.1 (CH), 107.3 (C), 25.3 (CH), 22.1 (CH_3_).

#### 2-(2,4-Dimethylbenzoyl)-3-isopropylbenzofuran-5-carbonitrile (**4d**)

A solution of (5-romo-3-isopropylbenzofuran-2-yl)­(2,4-dimethylphenyl)­methanone **3d** (1.45 g, 3.90 mmol), Zn­(CN)_2_ (550 mg, 4.70 mmol), Pd_2_dba_3_ (247 mg, 0.27 mmol), and dppf (305 mg, 0.55 mmol) in DMF (20 mL) was stirred and heated at 120 °C for 4 h. The resulting solution was filtered over Celite and washed with EtOAc. 1.8 M NH_4_OH_(aq)_ was added to the filtrate, and the layers were separated. The organic layer was washed with 1.8 M NH_4_OH_(aq)_ and brine and then dried (MgSO_4_) and evaporated under reduced pressure to give the crude product. Purification by flash column chromatography on silica with petroleum ether/EtOAc (95:5) as eluent gave product **4d** as an brown solid (1.136 g, 92%); *R*_F_ 0.2 (petroleum ether/EtOAc 95:5); mp 90–93 °C; IR (ATR) 2966, 2928, 2870, 2227 (C≡N str), 1644 (C=O str), 1608, 1557, 1445, 1365, 1311, 1268, 1229, 1121, 1091, 1047, 979, 893, 877, 811 cm^–1^; ^1^H NMR (300 MHz, CDCl_3_): δ 8.23 (dd, *J* = 1.5, 0.5 Hz, 1H, Ar), 7.68 (dd, *J* = 8.5, 1.5 Hz, 1H, Ar), 7.56 (dd, *J* = 8.5, 0.5 Hz, 1H, Ar), 7.39 (d, *J* = 8.0 Hz, 1H, Ar), 7.17–7.07 (m, 2H, Ar), 3.84 (hept, *J* = 7.0 Hz, 1H, C*H*(CH_3_)_2_), 2.41 (s, 3H, CH_3_), 2.39 (s, 3H, CH_3_), 1.46 (d, *J* = 7.0 Hz, 6H, CH­(C*H*_3_)_2_); ^13^C NMR (75.5 MHz, CDCl_3_): δ 188.8 (C), 156.2 (C), 149.0 (C), 142.2 (C), 138.0 (C), 135.2 (C), 134.6 (C), 132.4 (CH), 130.5 (CH), 129.7 (CH), 128.7 (CH), 128.0 (C), 126.3 (CH), 119.2 (C), 114.0 (CH), 107.2 (C), 25.3 (CH), 22.2 (CH_3_), 21.7 (CH_3_), 20.0 (CH_3_).

#### 2-(2,4-Dimethoxybenzoyl)-3-isopropylbenzofuran-5-carbonitrile (**4e**)

A solution of (5-bromo-3-isopropylbenzofuran-2-yl)­(2,4-dimethoxyphenyl)­methanone **3e** (1.24 g, 3.27 mmol), Zn­(CN)_2_ (433 mg, 3.69 mmol), Pd_2_dba_3_ (197 mg, 0.22 mmol), and dppf (238 mg, 0.43 mmol) in DMF (20 mL) was stirred and heated at 120 °C for 4 h. The resulting solution was filtered over Celite and washed with EtOAc. 1.8 M NH_4_OH_(aq)_ was added to the filtrate, and the layers were separated. The organic layer was washed with 1.8 M NH_4_OH_(aq)_ and brine, then dried (MgSO_4_), and evaporated under reduced pressure to give the crude product. Purification by flash column chromatography on silica with petroleum ether/EtOAc (80:20) as eluent gave product **4e** as an yellow solid (983 mg, 92%); *R*_F_ 0.2 (petroleum ether/EtOAc 80:20); mp 123–126 °C; IR (ATR) 2973, 2871, 2847, 2228 (C≡N str), 1640 (C=O str), 1604, 1583, 1462, 1416, 1278, 1262, 1213, 1174, 1109, 1054, 1022, 979, 904, 822, 811 cm^–1^; ^1^H NMR (300 MHz, CDCl_3_): δ 8.20 (dd, *J* = 1.5, 0.5 Hz, 1H, Ar), 7.65 (dd, *J* = 8.5, 1.5 Hz, 1H, Ar), 7.56–7.50 (m, 2H, Ar), 6.59 (dd, *J* = 8.5, 2.0 Hz, 1H, Ar), 6.51 (d, *J* = 2.0 Hz, 1H, Ar), 3.90 (s, 3H, OCH_3_), 3.82 (hept, *J* = 7.0 Hz, 1H, C*H*(CH_3_)_2_), 3.70 (s, 3H, OCH_3_), 1.45 (d, *J* = 7.0 Hz, 6H, CH­(C*H*_3_)_2_); ^13^C NMR (75.5 MHz, CDCl_3_): δ 185.6 (C), 164.6 (C), 160.5 (C), 156.0 (C), 149.9 (C), 132.5 (C), 132.5 (CH), 130.1 (CH), 128.5 (CH), 128.2 (C), 121.6 (C), 119.3 (C), 113.6 (CH), 106.8 (C), 105.2 (CH), 99.0 (CH), 56.0 (CH_3_), 55.8 (CH_3_), 25.2 (CH), 22.3 (CH_3_).

#### 3-Isopropyl-2-(2-methoxybenzoyl)­benzofuran-5-carbonitrile (**4f**)

A solution of (5-bromo-3-isopropylbenzofuran-2-yl)­(2-methoxyphenyl)­methanone **3f** (1.74 g, 4.70 mmol), Zn­(CN)_2_ (662 mg, 5.64 mmol), Pd_2_dba_3_ (302 mg, 0.33 mmol), and dppf (366 mg, 0.66 mmol) in DMF (20 mL) was stirred and heated at 120 °C for 4 h. The resulting solution was filtered over Celite and washed with EtOAc. 1.8 M NH_4_OH_(aq)_ was added to the filtrate, and the layers were separated. The organic layer was washed with 1.8 M NH_4_OH_(aq)_ and brine, then dried (MgSO_4_), and evaporated under reduced pressure to give the crude product. Purification by flash column chromatography on silica with petroleum ether/EtOAc (90:10) as eluent gave product **4f** as an yellow solid (1.365 g, 91%); *R*_F_ 0.2 (petroleum ether/EtOAc 90:10); mp 99–102 °C; IR (ATR) 2966, 2939, 2838, 2227 (C≡N str), 1663 (C=O str), 1600, 1562, 1486, 1462, 1435, 1362, 1310, 1291, 1269, 1243, 1228, 1167, 1113, 1089, 1056, 1043, 1020, 977, 906, 812 cm^–1^; ^1^H NMR (300 MHz, CDCl_3_): δ 8.22 (dd, *J* = 1.5, 0.5 Hz, 1H, Ar), 7.66 (dd, *J* = 8.5, 1.5 Hz, 1H, Ar), 7.57–7.44 (m, 3H, Ar), 7.08 (td, *J* = 7.5, 1.0 Hz, 1H, Ar), 7.01 (d, *J* = 8.5 Hz, 1H, Ar), 3.87 (hept, *J* = 7.0 Hz, 1H, C*H*(CH_3_)_2_), 3.73 (s, 3H, OCH_3_), 1.45 (d, *J* = 7.0 Hz, 6H, CH­(C*H*_3_)_2_); ^13^C NMR (75.5 MHz, CDCl_3_): δ 187.1 (C), 158.1 (C), 156.2 (C), 149.3 (C), 133.7 (C), 133.3 (CH), 130.4 (CH), 129.9 (CH), 128.8 (CH), 128.7 (C), 128.1 (C), 120.8 (CH), 119.2 (C), 113.8 (CH), 111.8 (CH), 107.0 (C), 56.0 (CH_3_), 25.2 (CH), 22.2 (CH_3_).

#### 2-(2-Naphthoyl)-3-isopropylbenzofuran-5-carbonitrile (**4g**)

A solution of (5-bromo-3-isopropylbenzofuran-2-yl)­(naphthalen-2-yl)­methanone **3g** (1.81 g, 4.60 mmol), Zn­(CN)_2_ (648 mg, 5.52 mmol), Pd_2_dba_3_ (293 mg, 0.32 mmol), and dppf (357 mg, 0.64 mmol) in DMF (20 mL) was stirred and heated at 120 °C for 4 h. The resulting solution was filtered over Celite and washed with EtOAc. 1.8 M NH_4_OH_(aq)_ was added to the filtrate, and the layers were separated. The organic layer was washed with 1.8 M NH_4_OH_(aq)_ and brine, then dried (MgSO_4_), and evaporated under reduced pressure to give the crude product. Purification by flash column chromatography on silica with petroleum ether/EtOAc (95:5) as eluent gave product **4g** as a tan solid (1.233 mg, 79%); *R*_F_ 0.2 (petroleum ether/EtOAc 95:5); mp 142–145 °C; IR (ATR) 2971, 2228 (C≡N str), 1652 (C=O str), 1623, 1567, 1468, 1364, 1309, 1268, 1221, 1202, 1169, 1122, 1090, 1054, 994, 955, 917, 822 cm^–1^; ^1^H NMR (300 MHz, CDCl_3_): δ 8.55 (d, *J* = 1.5 Hz, 1H, Ar), 8.28 (dd, *J* = 1.5, 0.5 Hz, 1H, Ar), 8.06 (dd, *J* = 8.5, 1.5 Hz, 1H, Ar), 8.01–7.86 (m, 3H, Ar), 7.73 (dd, *J* = 8.5, 1.5 Hz, 1H, Ar), 7.69–7.53 (m, 3H, Ar), 3.98 (hept, *J* = 7.0 Hz, 1H, C*H*(CH_3_)_2_), 1.51 (d, *J* = 7.0 Hz, 6H, CH­(C*H*_3_)_2_); ^13^C NMR (75.5 MHz, CDCl_3_): δ 186.0 (C), 156.1 (C), 148.7 (C), 135.7 (C), 135.1 (C), 134.7 (C), 132.5 (C), 132.0 (CH), 130.5 (CH), 129.8 (CH), 128.9 (CH), 128.6 (CH), 128.5 (CH), 128.0 (CH), 127.9 (C), 127.1 (CH), 125.2 (CH), 119.1 (C), 114.0 (CH), 107.3 (C), 25.5 (CH), 22.2 (CH_3_).

#### (5-(Aminomethyl)-3-isopropylbenzofuran-2-yl)­(*m*-tolyl)­methanol (**5a**)

A solution of 3-isopropyl-2-(3-methylbenzoyl)­benzofuran-5-carbonitrile **4a** (856 mg, 2.82 mmol) in THF (15 mL) was added dropwise to a stirred suspension of LiAlH_4_ (642 mg, 16.94 mmol) in THF (15 mL) at 0 °C. The resulting solution was allowed to warm to rt before heating at reflux for 16 h. The solution was then cooled to 0 °C and quenched sequentially with water (0.64 mL), 1 M NaOH_(aq)_ (1.28 mL), and water (0.64 mL), dried (Na_2_SO_4_), filtered over Celite, washed with CH_2_Cl_2_/MeOH (95:5), and evaporated under reduced pressure to give the crude product. Purification by flash column chromatography on silica with CH_2_Cl_2_ → CH_2_Cl_2_/MeOH (98:2) → CH_2_Cl_2_/MeOH/NH_4_OH_(aq)_ (96:3:1) as eluent gave product **5a** as an yellow oil (424 mg, 49%); *R*_F_ 0.2 (CH_2_Cl_2_/MeOH/NH_4_OH_(aq)_ 96:3:1); IR (ATR) 3356 (N–H str), 3288 (N–H str), 3027 (O–H str), 2962, 2927, 2868, 1465, 1445, 1255, 1030, 881, 863, 802 cm^–1^; ^1^H NMR (300 MHz, CDCl_3_): δ 7.59 (dd, *J* = 2.0, 0.5 Hz, 1H, Ar), 7.36 (dd, *J* = 8.5, 0.5 Hz, 1H, Ar), 7.29–7.07 (m, 5H, Ar), 6.03 (s, 1H, C*H*OH), 3.94 (s, 2H, CH_2_NH_2_), 3.27 (hept, *J* = 7.0 Hz, 1H, C*H*(CH_3_)_2_), 2.34 (s, 3H, C*H*_3_), 1.75 (s, 2H, CH_2_N*H*_2_), 1.44 (d, *J* = 7.0 Hz, 3H, CH­(C­(*H*_A_)_3_)), 1.42 (d, *J* = 7.0 Hz, 3H, CH­(C­(*H*_B_)_3_)); ^13^C NMR (75.5 MHz, CDCl_3_): δ 153.8 (C), 151.4 (C), 141.3 (C), 138.3 (C), 137.4 (C), 128.7 (CH), 128.5 (CH), 128.0 (C), 127.0 (CH), 123.7 (CH), 123.4 (CH), 122.5 (C), 119.6 (CH), 111.7 (CH), 68.5 (CH), 46.9 (CH_2_), 25.4 (CH), 22.8 (CH_3_), 22.7 (CH_3_), 21.6 (CH_3_).

#### (5-(Aminomethyl)-3-isopropylbenzofuran-2-yl)­(*o*-tolyl)­methanol (**5b**)

A solution of 3-isopropyl-2-(3-methylbenzoyl)­benzofuran-5-carbonitrile **4b** (1.14 g, 3.75 mmol) in THF (15 mL) was added dropwise to a stirred suspension of LiAlH_4_ (853 mg, 22.48 mmol) in THF (15 mL) at 0 °C. The resulting solution was allowed to warm to rt before heating at reflux for 16 h. The solution was then cooled to 0 °C and quenched sequentially with water (0.85 mL), 1 M NaOH_(aq)_ (1.7 mL), and water (0.85 mL), dried (Na_2_SO_4_), filtered over Celite, washed with CH_2_Cl_2_/MeOH (95:5), and evaporated under reduced pressure to give the crude product. Purification by flash column chromatography on silica with CH_2_Cl_2_ → CH_2_Cl_2_/MeOH (98:2) → CH_2_Cl_2_/MeOH/NH_4_OH_(aq)_ (96:3:1) as eluent gave product **5b** as an yellow oil (588 mg, 51%); *R*_F_ 0.2 (CH_2_Cl_2_/MeOH/NH_4_OH_(aq)_ 96:3:1); IR (ATR) 3356 (N–H str), 3288 (N–H str), 3024 (O–H str), 2962, 2928, 2869, 1580, 1460, 1445, 1251, 1026, 889, 873, 813 741 cm^–1^; ^1^H NMR (300 MHz, CDCl_3_): δ 7.70–7.63 (m, 1H, Ar), 7.53 (s, 1H, Ar), 7.31 (d, *J* = 8.5 Hz, 1H, Ar), 7.29–7.06 (m, 4H, Ar), 6.16 (s, 1H, C*H*OH), 3.84 (s, 2H, C*H*_2_NH_2_), 3.22 (hept, *J* = 7.0 Hz, 1H, C*H*(CH_3_)_2_), 2.29 (s, 3H, C*H*_3_), 1.38 (d, *J* = 7.0 Hz, 3H, CH­(C­(*H*_A_)_3_)), 1.37 (d, *J* = 7.0 Hz, 3H, CH­(C­(*H*_B_)_3_)); ^13^C NMR (75.5 MHz, CDCl_3_): δ 153.7 (C), 151.3 (C), 139.5 (C), 136.9 (C), 135.2 (C), 130.4 (CH), 128.0 (C), 127.7 (CH), 126.6 (CH), 126.1 (CH), 123.5 (CH), 122.2 (C), 119.7 (CH), 111.7 (CH), 65.8 (CH), 46.6 (CH2), 25.3 (CH), 22.5 (CH_3_), 22.3 (CH_3_), 19.3 (CH_3_).

#### (5-(Aminomethyl)-3-isopropylbenzofuran-2-yl)­(2,4-dimethylphenyl)­methanol (**5d**)

2-(2,4-Dimethylbenzoyl)-3-isopropylbenzofuran-5-carbonitrile **4d** (983 mg, 2.65 mmol) in THF (15 mL) was added dropwise to a stirred suspension of LiAlH_4_ (603 mg, 15.89 mmol) in THF (15 mL) at 0 °C. The resulting solution was allowed to warm to rt before heating at reflux for 16 h. The solution was then cooled to 0 °C and quenched sequentially with water (0.6 mL), 1 M NaOH_(aq)_ (1.2 mL), and water (0.6 mL), dried (Na_2_SO_4_), filtered over Celite, washed with CH_2_Cl_2_/MeOH (95:5), and evaporated under reduced pressure to give the crude product. Purification by flash column chromatography on silica with CH_2_Cl_2_ → CH_2_Cl_2_/MeOH (98:2) → CH_2_Cl_2_/MeOH/NH_4_OH_(aq)_ (96:3:1) as eluent gave product **5d** as a waxy white solid (628 mg, 63%); *R*_F_ 0.2 (CH_2_Cl_2_/MeOH/NH4OH_(aq)_ 96:3:1); IR (ATR) 3356 (N–H str), 3289 (N–H str), 3016 (O–H str), 2962, 2924, 2869, 1612, 1578, 1496, 1445, 1379, 1364, 1252, 1030, 887, 873, 805, 756 cm^–1^; ^1^H NMR (300 MHz, CDCl_3_): δ 7.59 (s, 1H, Ar), 7.52 (d, *J* = 8.0 Hz, 1H, Ar), 7.38 (d, *J* = 8.5 Hz, 1H, Ar), 7.19 (d, *J* = 8.5 Hz, 1H, Ar), 7.08 (d, *J* = 8.0 Hz, 1H, Ar), 7.01 (s, 1H, Ar), 6.18 (s, 1H, C*H*OH), 3.93 (s, 2H, C*H*_2_NH_2_), 3.26 (hept, *J* = 7.0 Hz, 1H, C*H*(CH_3_)_2_), 2.35 (s, 3H, CH_3_), 2.33 (s, 3H, CH_3_), 1.42 (d, *J* = 7.0 Hz, 3H, CH­(C­(*H*_A_)_3_)), 1.41 (d, *J* = 7.0 Hz, 3H, CH­(C­(*H*_B_)_3_)); ^13^C NMR (75.5 MHz, CDCl_3_): δ 153.7 (C), 151.2 (C), 137.5 (C), 137.2 (C), 136.4 (C), 135.2 (C), 131.4 (CH), 128.0 (C), 126.9 (CH), 126.6 (CH), 123.6 (CH), 122.2 (C), 119.7 (CH), 111.7 (CH), 66.0 (CH), 46.8 (CH_2_), 25.3 (CH), 22.6 (CH_3_), 22.4 (CH_3_), 21.1 (CH_3_), 19.3 (CH_3_).

#### (5-(Aminomethyl)-3-isopropylbenzofuran-2-yl)­(2,4-dimethoxyphenyl)­methanol (**5e**)

2-(2,4-Dimethoxybenzoyl)-3-isopropylbenzofuran-5-carbonitrile **4e** (869 mg, 2.49 mmol) in THF (25 mL) was added dropwise to a stirred suspension of LiAlH_4_ (566 mg, 22.94 mmol) in THF (25 mL) at 0 °C. The resulting solution was allowed to warm to rt before heating at reflux for 16 h. The solution was then cooled to 0 °C and quenched sequentially with water (0.57 mL), 1 M NaOH_(aq)_ (1.14 mL), and water (0.57 mL), dried (Na_2_SO_4_), filtered over Celite, washed with CH_2_Cl_2_/MeOH (95:5), and evaporated under reduced pressure to give the crude product. Recrystallization from CH_2_Cl_2_/hexane gave product **5e** as an off-white solid (430 mg, 49%); mp 159–162 °C; IR (ATR) 3361 (N–H str), 3298 (N–H str), 3081 (O–H str), 2962, 2933, 2869, 2836, 1710, 1662, 1611, 1588, 1504, 1465, 1362, 1290, 1254, 1206, 1179, 1156, 1120, 1035, 832, 801 cm^–1^; ^1^H NMR (300 MHz, CDCl_3_): δ 7.57 (dd, *J* = 2.0, 0.5 Hz, 1H, Ar), 7.37 (d, *J* = 8.5 Hz, 1H, Ar), 7.32 (d, *J* = 8.0 Hz, 1H, Ar), 7.17 (dd, *J* = 8.5, 2.0 Hz, 1H, Ar), 6.50–6.44 (m, 2H, Ar), 6.30 (s, 1H, C*H*OH), 3.93 (s, 2H, C*H*_2_NH_2_), 3.83 (s, 3H, OC*H*_3_), 3.79 (s, 3H, OC*H*_3_), 3.28 (hept, *J* = 7.0 Hz, 1H, C*H*(CH_3_)_2_), 1.42 (d, *J* = 7.0 Hz, 3H, CH­(C­(*H*_A_)_3_)), 1.37 (d, *J* = 7.0 Hz, 3H, CH­(C­(*H*_B_)_3_)); ^13^C NMR (75.5 MHz, CDCl_3_): δ 160.7 (C), 157.7 (C), 153.8 (C), 151.2 (C), 137.2 (C), 128.4 (CH), 128.1 (C), 123.4 (CH), 122.2 (C), 122.0 (C), 119.6 (CH), 111.7 (CH), 104.4 (CH), 98.7 (CH), 64.0 (CH), 55.6 (CH_3_), 55.5 (CH_3_), 46.9 (CH_2_), 25.3 (CH), 22.6 (CH_3_), 22.5 (CH_3_).

#### (5-(Aminomethyl)-3-isopropylbenzofuran-2-yl)­(2-methoxyphenyl)­methanol (**5f**)

3-Isopropyl-2-(2-methoxybenzoyl)­benzofuran-5-carbonitrile **4f** (1.22 g, 3.82 mmol) in THF (25 mL) was added dropwise to a stirred suspension of LiAlH_4_ (871 mg, 22.9 mmol) in THF (25 mL) at 0 °C. The resulting solution was allowed to warm to rt before heating at reflux for 16 h. The solution was then cooled to 0 °C and quenched sequentially with water (0.87 mL), 1 M NaOH_(aq)_ (1.74 mL), and water (0.87 mL), dried (Na_2_SO_4_), filtered over Celite, washed with CH_2_Cl_2_:MeOH (95:5), and evaporated under reduced pressure to give the crude product. Recrystallization from CH_2_Cl_2_/Hexane gave product **5f** as an off-white solid (580 mg, 47%); mp 134–136 °C; IR (ATR) 3358 (N–H str), 3293 (N–H str), 3050 (O–H str), 2961, 2930, 2869, 2836, 1600, 1588, 1489, 1463, 1438, 1239, 1183, 1046, 1027, 889, 873, 813, 753, 733 cm^–1^; ^1^H NMR (300 MHz, CDCl_3_): δ 7.57 (dd, *J* = 2.0, 0.5 Hz, 1H, Ar), 7.47 (dd, *J* = 7.5, 2.0 Hz, 1H, Ar), 7.36 (dd, *J* = 8.5, 0.5 Hz, 1H, Ar), 7.27 (ddd, *J* = 8.0, 7.5, 2.0 Hz, 1H, Ar), 7.16 (dd, *J* = 8.5, 2.0 Hz, 1H, Ar), 6.97 (td, *J* = 7.5, 1.0 Hz, 1H, Ar), 6.87 (dd, *J* = 8.0, 1.0 Hz, 1H, Ar), 6.36 (s, 1H, C*H*OH), 3.92 (s, 2H, C*H*_2_NH_2_), 3.84 (s, 3H, OC*H*_3_), 3.31 (hept, *J* = 7.0 Hz, 1H, C*H*(CH_3_)_2_), 1.43 (d, *J* = 7.0 Hz, 3H, CH­(C­(*H*_A_)_3_)), 1.38 (d, *J* = 7.0 Hz, 3H, CH­(C­(*H*_B_)_3_)); ^13^C NMR (75.5 MHz, CDCl_3_): δ 156.5 (C), 153.8 (C), 151.0 (C), 137.2 (C), 129.5 (C), 129.1 (CH), 128.1 (C), 127.7 (CH), 123.4 (CH), 122.2 (C), 120.9 (CH), 119.6 (CH), 111.7 (CH), 110.5 (CH), 64.2 (CH), 55.5 (CH_3_), 46.9 (CH_2_), 25.4 (CH), 22.6 (CH_3_), 22.5 (CH_3_).

#### (5-(Aminomethyl)-3-isopropylbenzofuran-2-yl)­(naphthalen-2-yl)­methanol (**5g**)

2-(2-Naphthoyl)-3-isopropylbenzofuran-5-carbonitrile **4g** (1.23 g, 3.63 mmol) in THF (25 mL) was added dropwise to a stirred suspension of LiAlH_4_ (827 mg, 21.80 mmol) in THF (25 mL) at 0 °C. The resulting solution was allowed to warm to rt before heating at reflux for 16 h. The solution was then cooled to 0 °C and quenched sequentially with water (0.83 mL), 1 M NaOH_(aq)_ (1.66 mL), and water (0.83 mL), dried (Na_2_SO_4_), filtered over Celite, washed with CH_2_Cl_2_/MeOH (95:5), and evaporated under reduced pressure to give the crude product. Purification by flash column chromatography on silica with CH_2_Cl_2_ → CH_2_Cl_2_/MeOH (98:2) → CH_2_Cl_2_/MeOH/NH_4_OH_(aq)_ (96:3:1) as eluent gave product **5g** as an orange oil (544 mg, 43%); *R*_F_ 0.2 (CH_2_Cl_2_/MeOH/NH_4_OH_(aq)_ 96:3:1); IR (ATR) 3355 (N–H str), 3290 (N–H str), 3053 (O–H str), 2963, 2928, 2870, 1635, 1601, 1507, 1469, 1445, 1364, 1264, 1156, 1120, 1091, 1030, 854, 819, 736 cm^–1^; ^1^H NMR (300 MHz, CDCl_3_): δ 7.93 (s, 1H, Ar), 7.86–7.78 (m, 3H, Ar), 7.62–7.59 (m, 1H, Ar), 7.52 (dd, *J* = 8.5, 2.0 Hz, 1H, Ar), 7.49–7.45 (m, 2H, Ar), 7.35 (d, *J* = 8.5 Hz, 1H, Ar), 7.20 (dd, *J* = 8.5, 2.0 Hz, 1H, Ar), 6.23 (s, 1H, C*H*OH), 3.95 (s, 2H, C*H*_2_NH_2_), 3.33 (hept, *J* = 7.0 Hz, 1H, C*H*(CH_3_)_2_), 1.46 (d, *J* = 7.0 Hz, 3H, CH­(C­(*H*_A_)_3_)), 1.45 (d, *J* = 7.0 Hz, 3H, CH­(C­(*H*_B_)_3_)); ^13^C NMR (75.5 MHz, CDCl_3_): δ 153.9 (C), 137.5 (C), 133.4 (C), 133.1 (C), 128.4 (C), 128.4 (CH), 128.3 (CH), 128.0 (C), 127.8 (CH), 126.4 (CH), 126.2 (C), 126.2 (CH), 125.0 (CH), 124.6 (CH), 123.9 (CH), 122.9 (C), 119.7 (CH), 111.7 (CH), 68.6 (CH), 46.8 (CH_2_), 25.4 (CH), 22.8 (CH_3_), 22.7 (CH_3_).

#### (5-(Aminomethyl)-3-isopropylbenzofuran-2-yl)­(*m*-tolyl)­methanone (**6a**)

A solution of (5-(aminomethyl)-3-isopropylbenzofuran-2-yl)­(*m*-tolyl)­methanol **5a** (420 mg, 1.36 mmol) and MnO_2_ (1.77 g, 20.36 mmol) in CH_2_Cl_2_ (14 mL) was stirred at rt for 22 h, and the reaction was monitored by TLC. The solution was filtered over Celite, washed with CH_2_Cl_2_/MeOH (95:5), and evaporated under reduced pressure to give the crude product. Purification by flash column chromatography on silica with CH_2_Cl_2_ → CH_2_Cl_2_/MeOH (99:1) → CH_2_Cl_2_/MeOH/NH_4_OH_(aq)_ (96:3:1) as eluent gave product **6a** as an orange oil (109 mg, 26%); *R*_F_ 0.2 (CH_2_Cl_2_/MeOH/NH_4_OH_(aq)_ 96:3:1); IR (ATR) 2966, 2928, 2871, 1645 (C=O str), 1603, 1584, 1556, 1463, 1363, 1312, 1284, 1268, 1058, 748 cm^–1^; ^1^H NMR (300 MHz, CDCl_3_): δ 7.85–7.77 (m, 3H, Ar), 7.50 (dd, *J* = 8.5, 0.5 Hz, 1H, Ar), 7.45–7.38 (m, 3H, Ar), 4.01 (s, 2H, C*H*_2_NH_2_), 3.93 (hept, *J* = 7.0 Hz, 1H, C*H*(CH_3_)_2_), 2.44 (s, 3H, C*H*_3_), 1.49 (d, *J* = 7.0 Hz, 6H, CH­(C*H*_3_)_2_); ^13^C NMR (75.5 MHz, CDCl_3_): δ 186.94 (C), 153.98 (C), 147.48 (C), 138.29 (C × 2), 138.20 (C), 135.99 (C), 133.58 (CH), 130.31 (CH), 128.26 (CH), 127.47 (C), 127.45 (C), 127.15 (CH), 121.41 (CH), 112.70 (CH), 46.75 (CH_2_), 25.58 (CH), 22.24 (CH_3_), 21.55 (CH_3_).

#### (5-(Aminomethyl)-3-isopropylbenzofuran-2-yl)­(*o*-tolyl)­methanone (**6b**)

A solution of (5-(aminomethyl)-3-isopropylbenzofuran-2-yl)­(*o*-tolyl)­methanol **5a** (569 mg, 1.84 mmol) and MnO_2_ (2.4 g, 27.6 mmol) in CH_2_Cl_2_ (18 mL) was stirred at rt for 20 h, and the reaction was monitored by TLC. The solution was filtered over Celite, washed with CH_2_Cl_2_/MeOH (95:5), and evaporated under reduced pressure to give the crude product. Purification by flash column chromatography on silica with CH_2_Cl_2_ → CH_2_Cl_2_/MeOH (99:1) → CH_2_Cl_2_/MeOH/NH_4_OH_(aq)_ (96:3:1) as eluent gave product **6b** as an orange-yellow oil (214 mg, 38%); *R*_F_ 0.2 (CH_2_Cl_2_/MeOH/NH_4_OH_(aq)_ 96:3:1); IR (ATR) 2966, 2930, 2871, 1650 (C=O str), 1555, 1461, 1364, 1310, 1265, 1055, 910, 743 cm^–1^; ^1^H NMR (300 MHz, CDCl_3_): δ 7.82–7.78 (m, 1H, Ar), 7.50–7.36 (m, 4H, Ar), 7.32–7.27 (m, 2H, Ar), 3.99 (s, 2H, C*H*_2_NH_2_), 3.85 (hept, *J* = 7.0 Hz, 1H, C*H*(CH_3_)_2_), 2.37 (s, 3H, C*H*_3_), 1.47 (d, *J* = 7.0 Hz, 6H, CH­(C*H*_3_)_2_); ^13^C NMR (75.5 MHz, CDCl_3_): δ 189.7 (C), 154.2 (C), 147.6 (C), 139.1 (C), 138.3 (C), 137.0 (C), 136.2 (C), 131.1 (CH), 130.8 (CH), 128.7 (CH), 127.8 (CH), 127.5 (C), 125.5 (CH), 121.6 (CH), 112.8 (CH), 46.7 (CH_2_), 25.5 (CH), 22.2 (CH_3_), 19.8 (CH_3_).

#### (5-(Aminomethyl)-3-isopropylbenzofuran-2-yl)­(2,4-dimethylphenyl)­methanone (**6d**)

A solution of (5-(aminomethyl)-3-isopropylbenzofuran-2-yl)­(2,4-dimethylphenyl)­methanol **5d** (596 mg, 1.84 mmol) and MnO_2_ (2.4 g, 27.6 mmol) in CH_2_Cl_2_ (18 mL) was stirred at rt for 46 h, and the reaction was monitored by TLC. The solution was filtered over Celite, washed with CH_2_Cl_2_/MeOH (95:5), and evaporated under reduced pressure to give the crude product. Purification by flash column chromatography on silica with CH_2_Cl_2_ → CH_2_Cl_2_/MeOH (99:1) → CH_2_Cl_2_/MeOH/NH_4_OH_(aq)_ (96:3:1) as eluent gave product **6d** as an orange-yellow oil (188 mg, 32%); *R*_F_ 0.2 (CH_2_Cl_2_/MeOH/NH_4_OH_(aq)_ 96:3:1); IR (ATR) 2966, 2928, 2872, 1648 (C=O str), 1612, 1556, 1462, 1310, 1266, 1230, 1051, 898, 825 cm^–1^; ^1^H NMR (300 MHz, CDCl_3_): δ 7.84–7.76 (m, 1H, Ar), 7.47–7.35 (m, 3H, Ar), 7.14–7.04 (m, 2H, Ar), 3.98 (s, 2H, C*H*_2_NH_2_), 3.87 (hept, *J* = 7.0 Hz, 1H, C*H*(CH_3_)_2_), 2.39 (s, 3H, CH_3_), 2.36 (s, 3H, CH_3_), 1.47 (d, *J* = 7.1 Hz, 6H, CH­(C*H*_3_)_2_); ^13^C NMR (75.5 MHz, CDCl_3_): δ 189.4 (C), 154.1 (C), 147.7 (C), 141.3 (C), 138.2 (C), 137.4 (C), 136.1 (C), 135.6 (C), 132.0 (CH), 129.3 (CH), 127.6 (CH), 127.5 (C), 126.1 (CH), 121.4 (CH), 112.7 (CH), 46.7 (CH_2_), 25.4 (CH), 22.2 (CH_3_), 21.6 (CH_3_), 19.9 (CH_3_).

#### (5-(Aminomethyl)-3-isopropylbenzofuran-2-yl)­(2,4-dimethoxyphenyl)­methanone (**6e**)

A solution of (5-(aminomethyl)-3-isopropylbenzofuran-2-yl)­(2,4-dimethoxyphenyl)­methanol **5e** (420 mg, 1.18 mmol) and MnO_2_ (1.54 g, 17.7 mmol) in CH_2_Cl_2_ (12 mL) was stirred at rt for 46 h, and the reaction was monitored by TLC. The solution was filtered over Celite, washed with CH_2_Cl_2_/MeOH (95:5), and evaporated under reduced pressure to give the crude product. Purification by flash column chromatography on silica with CH_2_Cl_2_ → CH_2_Cl_2_/MeOH (99:1) → CH_2_Cl_2_/MeOH/NH_4_OH_(aq)_ (96:3:1) as eluent gave product **6e** as an orange-yellow oil (186 mg, 45%); *R*_F_ 0.2 (CH_2_Cl_2_/MeOH/NH_4_OH_(aq)_ 96:3:1); IR (ATR) 2966, 2934, 2872, 2839, 1642 (C=O str), 1603, 1576, 1463, 1310, 1272, 1210, 1161, 1126, 1030 cm^–1^; ^1^H NMR (300 MHz, CDCl_3_): δ 7.77 (dd, *J* = 1.5, 1.0 Hz, 1H, Ar), 7.50 (d, *J* = 8.5 Hz, 1H, Ar), 7.41 (dd, *J* = 8.5, 1.0 Hz, 1H, Ar), 7.36 (dd, *J* = 8.5, 1.5 Hz, 1H, Ar), 6.57 (dd, *J* = 8.5, 2.0 Hz, 1H, Ar), 6.52 (d, *J* = 2.0 Hz, 1H, Ar), 3.98 (s, 2H, C*H*_2_NH_2_), 3.88 (s, 3H, OC*H*_3_), 3.86 (hept, *J* = 7.0 Hz, 1H, C*H*(CH_3_)_2_), 3.72 (s, 3H, OC*H*_3_), 1.46 (d, *J* = 7.0 Hz, 6H, CH­(C*H*_3_)_2_); ^13^C NMR (75.5 MHz, CDCl_3_): δ 186.2 (C), 163.9 (C), 160.2 (C), 153.9 (C), 148.5 (C), 137.9 (C), 134.0 (C), 132.1 (CH), 127.7 (C), 127.1 (CH), 122.3 (C), 121.3 (CH), 112.5 (CH), 104.8 (CH), 99.0 (CH), 55.9 (CH_3_), 55.6 (CH_3_), 46.8 (CH_2_), 25.3 (CH), 22.3 (CH_3_).

#### (5-(Aminomethyl)-3-isopropylbenzofuran-2-yl)­(2-methoxyphenyl)­methanone (**6f**)

A solution of (5-(aminomethyl)-3-isopropylbenzofuran-2-yl)­(2-methoxyphenyl)­methanol **5f** (570 mg, 1.75 mmol) and MnO_2_ (2.28 g, 26.3 mmol) in CH_2_Cl_2_ (18 mL) was stirred at rt for 117 h, and the reaction was monitored by TLC. The solution was filtered over Celite, washed with CH_2_Cl_2_/MeOH (95:5), and evaporated under reduced pressure to give the crude product. Purification by flash column chromatography on silica with CH_2_Cl_2_ → CH_2_Cl_2_/MeOH (99:1) → CH_2_Cl_2_/MeOH/NH_4_OH_(aq)_ (96:3:1) as eluent gave product **6f** as an orange-yellow oil (199 mg, 35%); *R*_F_ 0.2 (CH_2_Cl_2_/MeOH/NH_4_OH_(aq)_ 96:3:1); IR (ATR) 2966, 2933, 2872, 2837, 1650 (C=O str), 1599, 1581, 1559, 1488, 1464, 1435, 1293, 1249, 1044, 1024, 911, 755 cm^–1^; ^1^H NMR (300 MHz, CDCl_3_): δ 7.81–7.76 (m, 1H, Ar), 7.53–7.43 (m, 2H, Ar), 7.40–7.37 (m, 2H, Ar), 7.06 (td, *J* = 7.5, 0.9 Hz, 1H, Ar), 7.00 (d, *J* = 8.4 Hz, 1H, Ar), 3.98 (s, 2H, C*H*_2_NH_2_), 3.88 (hept, *J* = 7.0 Hz, 1H, C*H*(CH_3_)_2_), 3.75 (s, 3H, OCH_3_), 1.46 (d, *J* = 7.0 Hz, 6H, CH­(C*H*_3_)_2_); ^13^C NMR (75.5 MHz, CDCl_3_): δ 187.4 (C), 157.9 (C), 154.1 (C), 148.0 (C), 138.0 (C), 135.1 (C), 132.5 (CH), 129.7 (CH), 129.5 (C), 127.6 (C), 127.5 (CH), 121.6 (CH), 120.6 (CH), 112.6 (CH), 111.7 (CH), 56.0 (CH_3_), 46.8 (CH_2_), 25.4 (CH), 22.2 (CH_3_).

#### (5-(Aminomethyl)-3-isopropylbenzofuran-2-yl)­(naphthalen-2-yl)­methanone (**6g**)

A solution of (5-(aminomethyl)-3-isopropylbenzofuran-2-yl)­(naphthalen-2-yl)­methanol **5g** (356 mg, 1.03 mmol) and MnO_2_ (1.34 g, 15.5 mmol) in CH_2_Cl_2_ (10 mL) was stirred at rt for 46 h, and the reaction was monitored by TLC. The solution was filtered over Celite, washed with CH_2_Cl_2_/MeOH (95:5), and evaporated under reduced pressure to give the crude product. Purification by flash column chromatography on silica with CH_2_Cl_2_ → CH_2_Cl_2_/MeOH (99:1) → CH_2_Cl_2_/MeOH/NH_4_OH_(aq)_ (96:3:1) as eluent gave product **6g** as an orange-yellow oil (130 mg, 37%); *R*_F_ 0.2 (CH_2_Cl_2_/MeOH/NH_4_OH_(aq)_ 96:3:1); IR (ATR) 3059, 2967, 2931, 2872, 1694, 1644 (C=O), 1626, 1556, 1465, 1365, 1273, 1225, 1122, 1052, 822, 781, 760 cm^–1^; ^1^H NMR (300 MHz, CDCl_3_): δ 8.60–8.56 (m, 1H, Ar), 8.08 (dd, *J* = 8.5, 1.5 Hz, 1H, Ar), 8.01–7.88 (m, 3H, Ar), 7.85 (dd, *J* = 1.5, 1.0 Hz, 1H, Ar), 7.66–7.50 (m, 3H, Ar), 7.45 (dd, *J* = 8.5, 1.5 Hz, 1H, Ar), 4.04 (hept, *J* = 7.0 Hz, 1H, C*H*(CH_3_)_2_), 4.02 (s, 2H, C*H*_2_NH_2_), 1.52 (d, *J* = 7.0 Hz, 6H, CH­(C*H*_3_)_2_); ^13^C NMR (75.5 MHz, CDCl_3_): δ 186.5 (C), 154.1 (C), 147.6 (C), 138.3 (C), 136.2 (C), 135.5 (C), 135.5 (C), 132.6 (C), 131.8 (CH), 129.8 (CH), 128.5 (CH), 128.2 (CH), 128.0 (CH), 127.6 (CH), 127.5 (C), 126.8 (CH), 125.6 (CH), 121.5 (CH), 112.7 (CH), 46.8 (CH_2_), 25.7 (CH), 22.3 (CH_3_).

#### Ethyl 2-(((3-Isopropyl-2-(3-methylbenzoyl)­benzofuran-5-yl)­methyl)­amino)-2-oxoacetate (**7a**)

Ethyl oxalyl chloride (34 μL, 0.3 mmol) was added dropwise to a stirred solution of (5- (aminomethyl)-3-isopropylbenzofuran-2-yl)­(*m*-tolyl)­methanone **6a** (78 mg, 0.25 mmol) and TEA (71 μL, 0.51 mmol) in CH_2_Cl_2_ (2.5 mL) at 0 °C. The resulting solution was allowed to warm to rt and stirred for a further 2 h. CH_2_Cl_2_ (5 mL) and water (5 mL) were added, and the layers were separated. The organic layer was dried (MgSO_4_) and evaporated under reduced pressure to give the crude product. Purification by flash column chromatography on silica with petroleum ether/EtOAc (75:25) as eluent gave product **7a** as a yellow oil (32 mg, 31%); *R*_F_ 0.2 (petroleum ether/EtOAc 75:25); IR (ATR) 3342 (N–H str), 2969, 2931, 1734 (C=O str), 1690 (C=O str), 1645 (C=O str), 1557, 1463, 1368, 1302, 1269, 1209, 1168, 1095, 1059, 1021, 808, 748 cm^–1^; ^1^H NMR (300 MHz, CDCl_3_): δ 7.84–7.75 (m, 3H, Ar), 7.51 (dd, *J* = 8.5, 0.5 Hz, 1H, Ar), 7.44–7.38 (m, 3H, Ar), 4.64 (d, *J* = 6.0 Hz, 2H, C*H*_2_NH), 4.35 (q, *J* = 7.0 Hz, 2H, OC*H*_2_CH_3_), 3.92 (hept, *J* = 7.0 Hz, 1H, C*H*(CH_3_)_2_), 2.44 (s, 3H, C*H*_3_), 1.47 (d, *J* = 7.0 Hz, 6H, CH­(C*H*_3_)_2_), 1.38 (t, *J* = 7.0 Hz, 3H, OCH_2_C*H*_3_); ^13^C NMR (101 MHz, CDCl_3_): δ 186.8 (C), 160.8 (C), 156.6 (C), 154.3 (C), 147.7 (C), 138.2 (C), 138.1 (C), 135.7 (C), 133.7 (CH), 131.8 (C), 130.3 (CH), 128.3 (CH), 128.0 (CH), 127.6 (C), 127.1 (CH), 123.0 (CH), 113.2 (CH), 63.5 (CH_2_), 44.2 (CH_2_), 25.5 (CH), 22.2 (CH_3_), 21.5 (CH_3_), 14.1 (CH_3_).

#### Ethyl 2-(((3-Isopropyl-2-(2-methylbenzoyl)­benzofuran-5-yl)­methyl)­amino)-2-oxoacetate (**7b**)

Ethyl oxalyl chloride (92 μL, 0.8 mmol) was added dropwise to a stirred solution of (5- (aminomethyl)-3-isopropylbenzofuran-2-yl)­(*o*-tolyl)­methanone **6b** (210 mg, 0.68 mmol) and TEA (190 μL, 1.37 mmol) in CH_2_Cl_2_ (7 mL) at 0 °C. The resulting solution was allowed to warm to rt and stirred for a further 2 h. CH_2_Cl_2_ (10 mL) and water (10 mL) were added, and the layers were separated. The organic layer was dried (MgSO_4_) and evaporated under reduced pressure to give the crude product. Purification by flash column chromatography on silica with petroleum ether/EtOAc (75:25) as eluent gave product **7b** as a yellow oil (164 mg, 61%); *R*_F_ 0.2 (petroleum ether/EtOAc 75:25); IR (ATR) 3330 (N–H str), 2967, 2932, 2873, 1734 (C=O str), 1688 (C=O str), 1651 (C=O str), 1553, 1462, 1367, 1301, 1264, 1210, 1095, 1055, 1020, 973, 909, 730, 662 cm^–1^; ^1^H NMR (300 MHz, CDCl_3_): δ 7.81 (dd, *J* = 1.5, 1.0 Hz, 1H, Ar), 7.49–7.34 (m, 4H, Ar), 7.29 (d, *J* = 7.5 Hz, 2H, Ar), 4.62 (d, *J* = 6.0 Hz, 2H, C*H*_2_NH), 4.35 (q, *J* = 7.0 Hz, 2H, OC*H*_2_CH_3_), 3.83 (hept, *J* = 7.0 Hz, 1H, C*H*(CH_3_)_2_), 2.36 (s, 3H, C*H*_3_), 1.44 (d, *J* = 7.0 Hz, 6H, CH­(C*H*_3_)_2_), 1.38 (t, *J* = 7.0 Hz, 3H, OCH_2_C*H*_3_); ^13^C NMR (101 MHz, CDCl_3_): δ 189.5 (C), 160.7 (C), 156.6 (C), 154.5 (C), 147.8 (C), 138.8 (C), 137.0 (C), 135.9 (C), 131.9 (C), 131.1 (CH), 130.9 (CH), 128.7 (CH), 128.3 (CH), 127.7 (C), 125.5 (CH), 123.2 (CH), 113.3 (CH), 63.5 (CH_2_), 44.2 (CH_2_), 25.4 (CH), 22.2 (CH_3_), 19.8 (CH_3_), 14.1 (CH_3_).

#### Ethyl 2-(((2-(2-Chlorobenzoyl)-3-isopropylbenzofuran-5-yl)­methyl)­amino)-2-oxoacetate (**7c**)

2-(2-Chlorobenzoyl)-3-isopropylbenzofuran-5-carbonitrile **4c** (1.088 g, 3.36 mmol) in THF (15 mL) was added dropwise to a stirred suspension of LiAlH_4_ (765 mg, 20.2 mmol) in THF (15 mL) at 0 °C. The resulting solution was allowed to warm to rt before heating at reflux for 16 h. The solution was then cooled to 0 °C and quenched sequentially with water (0.77 mL), 1 M NaOH_(aq)_ (1.54 mL), and water (0.77 mL), dried (Na_2_SO_4_), filtered over Celite, washed with CH_2_Cl_2_/MeOH (95:5), and evaporated under reduced pressure to give the crude product. Purification by flash column chromatography on silica with CH_2_Cl_2_ → CH_2_Cl_2_/MeOH (98:2) → CH_2_Cl_2_/MeOH/NH_4_OH_(aq)_ (96:3:1) as eluent gave the impure amino alcohol **5c** (391 mg) that was used without further purification.

A solution of impure amino alcohol **5c** (391 mg, 1.2 mmol) and MnO_2_ (1.54 g, 17.7 mmol) in CH_2_Cl_2_ (12 mL) was stirred at rt for 69 h, and the reaction was monitored by TLC. The solution was filtered over Celite, washed with CH_2_Cl_2_/MeOH (95:5), and evaporated under reduced pressure to give the crude product. Purification by flash column chromatography on silica with CH_2_Cl_2_ → CH_2_Cl_2_/MeOH (99:1) → CH_2_Cl_2_/MeOH/NH_4_OH_(aq)_ (96:3:1) as eluent gave the impure amino ketone **6c** (116 mg), which was used without further purification.

Ethyl oxalyl chloride (47 μL, 0.42 mmol) was added dropwise to a stirred solution of crude amine **6c** (116 mg, 0.35 mmol) and TEA (99 μL, 0.71 mmol) in CH_2_Cl_2_ (3.5 mL) at 0 °C. The resulting solution was allowed to warm to rt and stirred for a further 2 h. CH_2_Cl_2_ (10 mL) and water (10 mL) were added, and the layers were separated. The organic layer was dried (MgSO_4_) and evaporated under reduced pressure to give the crude product. Purification by flash column chromatography on silica with petroleum ether/EtOAc (80:20) as eluent gave product **7c** as a yellow oil (10 mg, 0.7%); *R*_F_ 0.2 (petroleum ether/EtOAc 80:20); IR (ATR) 3342 (N–H str), 2970, 2933, 1735 (C=O str), 1690 (C=O str), 1646 (C=O str), 1554, 1303, 1266, 1212, 1055, 1021, 909, 730, 698 cm^–1^; ^1^H NMR (300 MHz, CDCl_3_): δ 8.05–7.97 (m, 2H, Ar), 7.82 (dd, *J* = 1.5, 0.5 Hz, 1H, Ar), 7.65–7.39 (m, 4H, Ar), 4.64 (d, *J* = 6.0 Hz, 2H, C*H*_2_NH), 4.37 (q, *J* = 7.0 Hz, 2H, OC*H*_2_CH_3_), 3.98 (hept, *J* = 7.0 Hz, 1H, C*H*(CH_3_)_2_), 1.48 (d, *J* = 7.0 Hz, 6H, CH­(C*H*_3_)_2_), 1.39 (t, *J* = 7.0 Hz, 3H, OCH_2_C*H*_3_); ^13^C NMR (101 MHz, CDCl_3_): δ 186.5 (C), 160.8 (C), 156.6 (C), 154.3 (C), 147.7 (C), 138.0 (C), 136.1 (C), 132.9 (CH), 131.9 (C), 129.9 (CH), 128.4 (CH), 128.1 (CH), 127.7 (C), 123.1 (CH), 113.2 (CH), 63.5 (CH_2_), 44.3 (CH_2_), 25.5 (CH), 22.2 (CH_3_), 14.1 (CH_3_).

#### Ethyl 2-(((2-(2,4-Dimethylbenzoyl)-3-isopropylbenzofuran-5-yl)­methyl)­amino)-2-oxoacetate (**7d**)

Ethyl oxalyl chloride (77 μL, 0.69 mmol) was added dropwise to a stirred solution of (5-(aminomethyl)-3-isopropylbenzofuran-2-yl)­(2,4-dimethylphenyl)­methanone **6d** (185 mg, 0.58 mmol) and TEA (160 μL, 1.15 mmol) in CH_2_Cl_2_ (6 mL) at 0 °C. The resulting solution was allowed to warm to rt and stirred for a further 2 h. CH_2_Cl_2_ (10 mL) and water (10 mL) were added, and the layers were separated. The organic layer was dried (MgSO_4_) and evaporated under reduced pressure to give the crude product. Purification by flash column chromatography on silica with petroleum ether/EtOAc (70:30) as eluent gave product **7d** as a yellow oil (162 mg, 66%); *R*_F_ 0.2 (petroleum ether/EtOAc 70:30); IR (ATR) 3291 (N–H str), 2967, 1733 (C=O str), 1687 (C=O str), 1656 (C=O str), 1563, 1303, 1291, 1269, 1220, 1054, 1024, 976, 822, 813, 777, 720, 699, 681, 624 cm^–1^; ^1^H NMR (300 MHz, CDCl_3_): δ 7.79 (dd, *J* = 2.0, 1.0 Hz, 1H, Ar), 7.45 (dd, *J* = 8.5, 0.5 Hz, 1H, Ar), 7.41–7.35 (m, 2H, Ar), 7.14–7.06 (m, 2H, Ar), 4.62 (d, *J* = 6.0 Hz, 2H, C*H*_2_NH), 4.36 (q, *J* = 7.0 Hz, 2H, OC*H*_2_CH_3_), 3.85 (hept, *J* = 7.0 Hz, 1H, C*H*(CH_3_)_2_), 2.40 (s, 3H, CH_3_), 2.36 (s, 3H, CH_3_), 1.45 (d, *J* = 7.0 Hz, 6H, CH­(C*H*_3_)_2_), 1.39 (t, *J* = 7.0 Hz, 3H, OCH_2_C*H*_3_); ^13^C NMR (101 MHz, CDCl_3_): δ 189.3 (C), 160.8 (C), 156.6 (C), 154.5 (C), 148.1 (C), 141.5 (C), 137.6 (C), 135.9 (C), 135.4 (C), 132.1 (CH), 131.8 (C), 129.4 (CH), 128.1 (CH), 127.8 (C), 126.2 (CH), 123.1 (CH), 113.3 (CH), 63.5 (CH_2_), 44.3 (CH_2_), 25.4 (CH), 22.3 (CH_3_), 21.6 (CH_3_), 19.9 (CH_3_), 14.1 (CH_3_).

#### Ethyl 2-(((2-(2,4-Dimethoxybenzoyl)-3-isopropylbenzofuran-5-yl)­methyl)­amino)-2-oxoacetate (**7e**)

Ethyl oxalyl chloride (45 μL, 0.40 mmol) was added dropwise to a stirred solution of (5-(aminomethyl)-3-isopropylbenzofuran-2-yl)­(2,4-dimethoxyphenyl)­methanone **6e** (117 mg, 0.33 mmol) and TEA (92 μL, 0.66 mmol) in CH_2_Cl_2_ (3 mL) at 0 °C. The resulting solution was allowed to warm to rt and stirred for a further 2 h. CH_2_Cl_2_ (10 mL) and water (10 mL) were added, and the layers were separated. The organic layer was dried (MgSO_4_) and evaporated under reduced pressure to give the crude product. Purification by flash column chromatography on silica with petroleum ether/EtOAc (70:30) as eluent gave product **7e** as a yellow oil (103 mg, 69%); *R*_F_ 0.2 (petroleum ether/EtOAc 70:30); IR (ATR) 3336 (N–H str), 2968, 2937, 2873, 1734 (C=O str), 1693 (C=O str), 1642 (C=O str), 1602, 1574, 1504, 1463, 1439, 1418, 1367, 1306, 1272, 1210, 1162, 1126, 1025, 974, 906, 730 cm^–1^; ^1^H NMR (300 MHz, CDCl_3_): δ 7.77 (dd, *J* = 1.5, 1.0 Hz, 1H, Ar), 7.49 (d, *J* = 8.5 Hz, 1H, Ar), 7.41 (d, *J* = 8.5 Hz, 1H, Ar), 7.34 (dd, *J* = 8.5, 1.5 Hz, 1H, Ar), 6.56 (dd, *J* = 8.5, 2.5 Hz, 1H, Ar), 6.50 (d, *J* = 2.5 Hz, 1H, Ar), 4.61 (d, *J* = 6.0 Hz, 2H, C*H*_2_NH), 4.34 (q, *J* = 7.0 Hz, 2H, OC*H*_2_CH_3_), 3.87 (s, 3H, OC*H*_3_), 3.83 (hept, *J* = 7.0 Hz, 1H, C*H*(CH_3_)_2_), 3.70 (s, 3H, OC*H*_3_), 1.43 (d, *J* = 7.0 Hz, 6H, CH­(C*H*_3_)_2_), 1.37 (t, *J* = 7.0 Hz, 3H, OCH_2_C*H*_3_); ^13^C NMR (101 MHz, CDCl_3_): δ 186.1 (C), 164.0 (C), 160.8 (C), 160.2 (C), 156.5 (C), 154.2 (C), 148.8 (C), 133.6 (C), 132.2 (CH), 131.4 (C), 127.9 (C), 127.5 (CH), 122.9 (CH), 122.1 (C), 112.9 (CH), 104.9 (CH), 98.9 (CH), 63.4 (CH_2_), 55.9 (CH_3_), 55.6 (CH_3_), 44.2 (CH_2_), 25.3 (CH), 22.2 (CH_3_), 14.1 (CH_3_).

#### Ethyl 2-(((3-Isopropyl-2-(2-methoxybenzoyl)­benzofuran-5-yl)­methyl)­amino)-2-oxoacetate (**7f**)

Ethyl oxalyl chloride (42 μL, 0.38 mmol) was added dropwise to a stirred solution of (5-(aminomethyl)-3-isopropylbenzofuran-2-yl)­(2-methoxyphenyl)­methanone **6f** (102 mg, 0.32 mmol) and TEA (88 μL, 0.63 mmol) in CH_2_Cl_2_ (3 mL) at 0 °C. The resulting solution was allowed to warm to rt and stirred for a further 2 h. CH_2_Cl_2_ (10 mL) and water (10 mL) were added, and the layers were separated. The organic layer was dried (MgSO_4_) and evaporated under reduced pressure to give the crude product. Purification by flash column chromatography on silica with petroleum ether/EtOAc (70:30) as eluent gave product **7f** as a yellow oil (74 mg, 54%); *R*_F_ 0.2 (petroleum ether/EtOAc 70:30); IR (ATR) 3337 (N–H str), 2967, 2933, 2873, 1736 (C=O str), 1691 (C=O str), 1650 (C=O str), 1599, 1557, 1487, 1463, 1435, 1367, 1294, 1248, 1210, 1021, 909, 755, 730 cm^–1^; ^1^H NMR (300 MHz, CDCl_3_): δ 7.79 (dd, *J* = 1.5, 1.0 Hz, 1H, Ar), 7.54–7.41 (m, 3H, Ar), 7.36 (dd, *J* = 8.5, 1.5 Hz, 1H, Ar), 7.09–6.98 (m, 2H, Ar), 4.61 (d, *J* = 6.0 Hz, 2H, C*H*_2_NH), 4.35 (q, *J* = 7.0 Hz, 2H, OC*H*_2_CH_3_), 3.86 (hept, *J* = 7.0 Hz, 1H, C*H*(CH_3_)_2_), 3.73 (s, 3H, OC*H*_3_), 1.44 (d, *J* = 7.0 Hz, 6H, CH­(C*H*_3_)­2), 1.38 (t, *J* = 7.0 Hz, 3H, OCH_2_C*H*_3_); ^13^C NMR (101 MHz, CDCl_3_): δ 187.4 (C), 160.8 (C), 157.9 (C), 156.6 (C), 154.4 (C), 148.3 (C), 134.7 (C), 132.7 (CH), 131.5 (C), 129.7 (CH), 129.3 (C), 128.0 (CH), 127.8 (C), 123.1 (CH), 120.7 (CH), 113.1 (CH), 111.7 (CH), 63.5 (CH_2_), 55.9 (CH_3_), 44.3 (CH_2_), 25.3 (CH), 22.2 (CH_3_), 14.1 (CH_3_).

#### Ethyl 2-(((2-(2-Naphthoyl)-3-isopropylbenzofuran-5-yl)­methyl)­amino)-2-oxoacetate (**7g**)

Ethyl oxalyl chloride (34 μL, 0.3 mmol) was added dropwise to a stirred solution of (5-(aminomethyl)-3-isopropylbenzofuran-2-yl)­(naphthalen-2-yl)­methanone **6g** (85 mg, 0.25 mmol), and TEA (70 μL, 0.5 mmol) in CH_2_Cl_2_ (2.5 mL) at 0 °C. The resulting solution was allowed to warm to rt and stirred for a further 2 h. CH_2_Cl_2_ (10 mL) and water (10 mL) were added, and the layers were separated. The organic layer was dried (MgSO_4_) and evaporated under reduced pressure to give the crude product. Purification by flash column chromatography on silica with petroleum ether/EtOAc (80:20) as eluent gave product **7g** as a yellow oil (34 mg, 31%); *R*_F_ 0.2 (petroleum ether/EtOAc 80:20); IR (ATR) 3342 (N–H str), 3059, 2968, 2933, 2872, 1734 (C=O str), 1694 (C=O str), 1644 (C=O str), 1625, 1557, 1464, 1368, 1302, 1288, 1272, 1213, 1195, 1171, 1122, 1020, 909, 781, 760, 731 cm^–1^; ^1^H NMR (300 MHz, CDCl_3_): δ 8.60–8.53 (m, 1H, Ar), 8.06 (dd, *J* = 8.5, 1.5 Hz, 1H, Ar), 8.01–7.82 (m, 4H, Ar), 7.66–7.51 (m, 3H, Ar), 7.43 (dd, *J* = 8.5, 2.0 Hz, 1H, Ar), 4.66 (d, *J* = 6.0 Hz, 2H, C*H*_2_NH), 4.37 (q, *J* = 7.0 Hz, 2H, OC*H*_2_CH_3_), 3.99 (hept, *J* = 7.0 Hz, 1H, C*H*(CH_3_)_2_), 1.50 (d, *J* = 7.0 Hz, 6H, CH­(C*H*_3_)_2_), 1.39 (t, *J* = 7.0 Hz, 3H, OCH_2_C*H*_3_); ^13^C NMR (75.5 MHz, CDCl_3_): δ 186.4 (C), 160.8 (C), 156.6 (C), 154.4 (C), 147.8 (C), 135.9 (C), 135.6 (C), 135.3 (C), 132.5 (C), 131.9 (C), 131.8 (CH), 129.8 (CH), 128.6 (CH), 128.3 (CH), 128.0 (CH), 127.9 (CH), 127.7 (C), 126.9 (CH), 125.5 (CH), 123.0 (CH), 113.2 (CH), 63.5 (CH_2_), 44.2 (CH_2_), 25.6 (CH), 22.3 (CH_3_), 14.1 (CH_3_).

#### 2-(((3-Isopropyl-2-(3-methylbenzoyl)­benzofuran-5-yl)­methyl)­amino)-2-oxoacetic Acid (**DM239**)

A solution of ethyl 2-(((3-isopropyl-2-(3-methylbenzoyl)­benzofuran-5-yl)­methyl)­amino)-2-oxoacetate **7a** (32 mg, 0.08 mmol) and NaOH (7 mg, 0.16 mmol) in MeOH/THF (1:1, 0.8 mL) was stirred at rt for 20 min. The solution was then acidified with 1 M HCl_(aq)_ (3 mL). Water (10 mL) was added, and organics were extracted with CH_2_Cl_2_ (3 × 10 mL). The organic layers were combined, dried (MgSO_4_), and evaporated under reduced pressure to give product **DM239** as an amorphous solid (25 mg, 83%); IR (ATR) 3069 (O–H str, N–H Str), 2956, 2923, 2852, 1692 (C=O str), 1643 (C=O str), 1538, 1487, 1438, 1365, 1208, 1053, 1005, 874 cm^–1^; ^1^H NMR (300 MHz, CDCl_3_): δ 7.83–7.76 (m, 3H, Ar), 7.53 (d, *J* = 8.5 Hz, 1H, Ar), 7.45–7.36 (m, 3H, Ar), 4.65 (d, *J* = 6.0 Hz, 2H, C*H*_2_NH), 3.92 (hept, *J* = 7.0 Hz, 1H, C*H*(CH_3_)_2_), 2.44 (s, 3H, CC*H*_3_), 1.47 (d, *J* = 7.0 Hz, 6H, CH­(C*H*_3_)_2_); MS (ESI) *m*/*z*: 381 [(M + H)^+^, 32]; HRMS (ESI) *m*/*z*: C_22_H_21_NO_5_ ([M + H]^+^) calcd for 381.2159; found, 381.2160.

#### 2-(((3-Isopropyl-2-(2-methylbenzoyl)­benzofuran-5-yl)­methyl)­amino)-2-oxoacetic Acid (**DM240**)

A solution of ethyl 2-(((3-isopropyl-2-(2-methylbenzoyl)­benzofuran-5-yl)­methyl)­amino)-2-oxoacetate **7b** (164 mg, 0.4 mmol) and NaOH (32 mg, 0.8 mmol) in MeOH/THF (1:1, 4 mL) was stirred at rt for 20 min. The solution was then acidified with 1 M HCl_(aq)_ (3 mL). Water (10 mL) was added, and organics were extracted with CH_2_Cl_2_ (3 × 10 mL). The organic layers were combined, dried (MgSO_4_), and evaporated under reduced pressure to give product **DM240** as an off-white solid (112 mg, 74%); mp 118–120 °C, IR (ATR) 3305 (O–H str, N–H str), 2965, 2928, 2872, 1646 (C=O), 1551, 1292, 1264, 1231, 1046, 973, 908, 742 cm^–1^; ^1^H NMR (300 MHz, CDCl_3_): δ 7.81 (s, 1H, Ar), 7.49–7.37 (m, 3H, Ar), 7.32–7.26 (m, 3H, Ar), 4.62 (s, 2H, C*H*_2_NH), 3.82 (hept, *J* = 7.0 Hz, 1H, C*H*(CH_3_)_2_), 2.36 (s, 3H, CC*H*_3_), 1.44 (d, *J* = 7.0 Hz, 6H, CH­(C*H*_3_)_2_)); MS (ESI) *m*/*z*: 381 [(M + H)^+^, 32]; HRMS (ESI) *m*/*z*: C_22_H_21_NO_5_ ([M + H]^+^) calcd for 381.2159; found, 381.2160.

#### 2-(((2-(2-Chlorobenzoyl)-3-isopropylbenzofuran-5-yl)­methyl)­amino)-2-oxoacetic Acid (**DM241**)

A solution of ethyl 2-(((2-(2-chlorobenzoyl)-3-isopropylbenzofuran-5-yl)­methyl)­amino)-2-oxoacetate **7c** (10 mg, 0.025 mmol) and NaOH (2 mg, 0.05 mmol) in MeOH/THF (1:1, 0.25 mL) was stirred at rt for 20 min. The solution was then acidified with 1 M HCl_(aq)_ (3 mL). Water (10 mL) was added, and organics were extracted with CH_2_Cl_2_ (3 × 10 mL). The organic layers were combined, dried (MgSO_4_), and evaporated under reduced pressure to give product **DM241** as an amorphous solid (8 mg, 80%); IR (ATR) 3305 (O–H str, N–H str), 2966, 2928, 1744 (C=O str), 1683 (C=O str), 1644 (C=O str), 1611, 1549, 1461, 1352, 1312, 1289, 1265, 1228, 1124, 1094, 1050, 1011, 974, 936, 896, 875, 824, 810, 726 cm^–1^; ^1^H NMR (300 MHz, CDCl_3_): δ 8.01 (d, *J* = 7.5 Hz, 2H, Ar), 7.82 (s, 1H, Ar), 7.65–7.47 (m, 4H, Ar), 4.64 (s, 2H, C*H*_2_NH), 3.98 (hept, *J* = 7.0 Hz, 1H, C*H*(CH_3_)_2_), 1.48 (d, *J* = 7.0 Hz, 6H, CH­(C*H*_3_)_2_); MS (ESI) *m*/*z*: 397 [(M + H)^+^, 32]; HRMS (ESI) *m*/*z*: C_21_H_18_ClNO_5_ ([M + H]^+^) calcd for 397.1156; found, 397.1159.

#### 2-(((2-(2,4-Dimethylbenzoyl)-3-isopropylbenzofuran-5-yl)­methyl)­amino)-2-oxoacetic Acid (**DM242**)

A solution of ethyl 2-(((2-(2,4-dimethylbenzoyl)-3-isopropylbenzofuran-5-yl)­methyl)­amino)-2-oxoacetate **7d** (144 mg, 0.34 mmol) and NaOH (27 mg, 0.68 mmol) in MeOH/THF (1:1, 3.4 mL) was stirred at rt for 20 min. The solution was then acidified with 1 M HCl_(aq)_ (3 mL). Water (10 mL) was added, and organics were extracted with CH_2_Cl_2_ (3 × 10 mL). The organic layers were combined, dried (MgSO_4_), and evaporated under reduced pressure to give product **DM242** as an off-white solid (132 mg, 99%); mp 78–80 °C; IR (ATR) 3005 (O–H str, N–H str), 2967, 2928, 2872, 1760 (C=O str), 1683 (C=O str), 1644 (C=O str), 1611, 1550, 1462, 1352, 1313, 1289, 1265, 1229, 1050, 974, 896, 776 cm^–1^; ^1^H NMR (300 MHz, CDCl_3_): δ 7.82–7.79 (m, 1H, Ar), 7.45 (d, *J* = 8.5 Hz, 1H, Ar), 7.40–7.33 (m, 2H, Ar), 7.15–7.06 (m, 2H, Ar), 4.63 (d, *J* = 6.0 Hz, 2H, C*H*_2_NH), 3.83 (hept, *J* = 7.0 Hz, 1H, C*H*(CH_3_)_2_), 2.39 (s, 3H, CC*H*_3_), 2.36 (s, 3H, CC*H*_3_), 1.44 (d, *J* = 7.0 Hz, 6H, CH­(C*H*_3_)_2_); ^13^C NMR (75.5 MHz, CDCl_3_): δ 189.4 (C), 160.4 (C), 157.6 (C), 154.5 (C), 148.1 (C), 141.6 (C), 137.5 (C), 135.8 (C), 135.3 (C), 132.1 (CH), 131.0 (C), 129.4 (CH), 127.9 (CH), 127.8 (C), 126.2 (CH), 123.1 (CH), 113.3 (C), 44.7 (CH_2_), 25.4 (CH), 22.2 (CH_3_), 21.6 (CH_3_), 19.9 (CH_3_); MS (ESI) *m*/*z*: 809 [(M + Na)^+^, 5] 394 [(M + H)^+^, 10], 147 [(BF + CH_2_NH_2_)^+^, 100]; HRMS (ESI) *m*/*z*: C_21_H_18_ClNO_5_ ([M + H]^+^) calcd for 394.1649; found, 394.1649.

#### 2-(((2-(2,4-Dimethoxybenzoyl)-3-isopropylbenzofuran-5-yl)­methyl)­amino)-2-oxoacetic Acid (**DM244**)

A solution of ethyl 2-(((2-(2,4-dimethoxybenzoyl)-3-isopropylbenzofuran-5- yl)­methyl)­amino)-2-oxoacetate **7e** (78 mg, 0.17 mmol) and NaOH (14 mg, 0.35 mmol) in MeOH/THF (1:1, 1.7 mL) was stirred at rt for 20 min. The solution was then acidified with 1 M HCl_(aq)_ (3 mL). Water (10 mL) was added, and organics were extracted with CH_2_Cl_2_ (3 × 10 mL). The organic layers were combined, dried (MgSO_4_), and evaporated under reduced pressure to give product **DM244** as an off-white solid (70 mg, 97%); mp 83–85 °C; IR (ATR) 3305 (O–H str, N–H str), 2965, 2934, 2873, 2840, 1689 (C=O str), 1640 (C=O str), 1602, 1574, 1504, 1462, 1363, 1310, 1272, 1231, 1210, 1161, 1125, 1026, 814, 775 cm^–1^; ^1^H NMR (300 MHz, CDCl_3_): δ 7.77 (d, *J* = 1.5 Hz, 1H, Ar), 7.50 (d, *J* = 8.5 Hz, 1H, Ar), 7.42 (d, *J* = 8.5 Hz, 1H, Ar), 7.32 (dd, *J* = 8.5, 1.5 Hz, 1H, Ar), 6.57 (dd, *J* = 8.5, 2.0 Hz, 1H, Ar), 6.51 (d, *J* = 2.0 Hz, 1H, Ar), 4.62 (d, *J* = 6.0 Hz, 2H, C*H*_2_NH), 3.90–3.77 (m, 4H, OC*H*_3_ + C*H*(CH_3_)_2_), 3.70 (s, 3H, OC*H*_3_), 1.43 (d, *J* = 7.0 Hz, 6H, CH­(C*H*_3_)_2_); MS (ESI) *m*/*z*: 448 [(M + Na)^+^, 8], 426 [(M + H)^+^, 12], 382 [(M–CO_2_ + H)^+^, 100]; HRMS (ESI) *m*/*z*: C_23_H_23_NO_7_ ([M + H]^+^) calcd for 426.1547; found, 426.1556.

#### 2-(((3-Isopropyl-2-(2-methoxybenzoyl)­benzofuran-5-yl)­methyl)­amino)-2-oxoacetic Acid (**DM243**)

A solution of ethyl 2-(((3-isopropyl-2-(2-methoxybenzoyl)­benzofuran-5-yl)­methyl)­amino)-2-oxoacetate **7f** (74 mg, 0.17 mmol) and NaOH (14 mg, 0.35 mmol) in MeOH/THF (1:1, 1.7 mL) was stirred at rt for 20 min. The solution was then acidified with 1 M HCl_(aq)_ (3 mL). Water (10 mL) was added, and organics were extracted with CH_2_Cl_2_ (3 × 10 mL). The organic layers were combined, dried (MgSO_4_), and evaporated under reduced pressure to give product **DM243** as an off-white solid (60 mg, 83%); mp 76–78 °C; IR (ATR) 3307 (O–H str, N–H str), 2966, 2930, 1688 (C=O str), 1645 (C=O str), 1599, 1555, 1462, 1293, 1248, 1044, 1020, 909, 754, 657 cm^–1^; ^1^H NMR (300 MHz, CDCl_3_): δ 7.81–7.77 (m, 1H, Ar), 7.54–7.40 (m, 3H, Ar), 7.34 (dd, *J* = 8.5, 2.0 Hz, 1H, Ar), 7.10–6.97 (m, 2H, Ar), 4.62 (d, *J* = 6.0 Hz, 2H, C*H*_2_NH), 3.86 (hept, *J* = 7.0 Hz, 1H, C*H*(CH_3_)_2_), 3.73 (s, 3H, OC*H*_3_), 1.44 (d, *J* = 7.0 Hz, 6H, CH­(C*H*_3_)_2_); MS (ESI) *m*/*z*: 396 [(M + H)^+^, 2], 147 [(BF + CH_2_NH_2_)^+^, 100]; HRMS (ESI) *m*/*z*: C_22_H_21_NO_6_ ([M + H]^+^) calcd for 396.1442; found, 396.1436.

#### 2-(((2-(2-Naphthoyl)-3-isopropylbenzofuran-5-yl)­methyl)­amino)-2-oxoacetic Acid (**DM245**)

A solution of ethyl 2-(((2-(2-naphthoyl)-3-isopropylbenzofuran-5-yl)­methyl)­amino)-2-oxoacetate **7g** (34 mg, 0.08 mmol) and NaOH (6 mg, 0.16 mmol) in MeOH/THF (1:1, 0.8 mL) was stirred at rt for 20 min. The solution was then acidified with 1 M HCl_(aq)_ (3 mL). Water (10 mL) was added, and organics were extracted with CH_2_Cl_2_ (3 × 10 mL). The organic layers were combined, dried (MgSO_4_), and evaporated under reduced pressure to give product **DM245** as an amorphous solid (26 mg, 79%); IR (ATR) 3306 (O–H str, N–H str), 3058, 2965, 2927, 2872, 1689 (C=O str), 1643 (C=O str), 1625, 1597, 1533, 1463, 1363, 1266, 1224, 1195, 1122, 1052, 780, 734 cm^–1^; ^1^H NMR (300 MHz, CDCl_3_): δ 8.56 (s, 1H, Ar), 8.09–8.03 (m, 1H, Ar), 8.00–7.81 (m, 4H, Ar), 7.66–7.50 (m, 3H, Ar), 7.44–7.37 (m, 1H, Ar), 4.65 (d, *J* = 5.0 Hz, 2H, C*H*_2_NH), 3.99 (hept, *J* = 7.0 Hz, 1H, C*H*(CH_3_)_2_), 1.50 (d, *J* = 7.0 Hz, 6H, CH­(C*H*_3_)_2_); MS (ESI) *m*/*z*: 394 [(M + H)^+^, 100]; HRMS (ESI) *m*/*z*: C_25_H_21_NO_5_ ([M + H]^+^) calcd for 394.1398; found, 394.1402.

#### 3-Bromo-4-hydroxybenzaldehyde (**9**)

4-Hydroxybenzaldehyde (10 g, 82 mmol) and iodine (533 mg, 2.1 mmol) were dissolved in CH_2_Cl_2_ (50 mL), and the resulting solution was cooled to −5 °C. Bromine (4.4 mL, 86 mmol) in CH_2_Cl_2_ (30 mL) was added slowly, while keeping the reaction solution temperature below 0 °C. The resulting solution was warmed to rt and stirred at rt for 16 h. Then, 0.5 M Na_2_S_2_O_3(aq)_ (100 mL) was added, and the resulting solution was stirred at rt for a further 30 min. The precipitate was filtered and washed with H_2_O (100 mL), before being air-dried over 16 h to give **9** (9.738 g, 59%) as a white solid, mp 126–128 °C; IR (ATR) 3091 (O–H str), 1678 (C=O str), 1650, 1589, 1557, 1498, 1421, 1381, 1334, 1312, 1274, 1196, 1154, 1138, 1037, 1018, 892, 880 cm^–1^; ^1^H NMR (300 MHz, DMSO-*d*_6_): δ 9.73 (s, 1H, C*H*O), 8.00 (d, *J* = 2.0 Hz, 1H, Ar), 7.74 (dd, *J* = 8.5, 2.0 Hz, 1H, Ar), 7.08 (d, *J* = 8.5 Hz, 1H, Ar); ^13^C NMR (75.5 MHz, DMSO-*d*_6_): δ 190.9 (CH), 160.1 (C), 135.3 (CH), 131.1 (CH), 130.0 (C), 116.9 (CH), 110.5 (C). Spectroscopic data consistent with that reported in literature.^27^

#### 4-(Benzyloxy)-3-bromobenzaldehyde (**10**)

3-Bromo-4-hydroxybenzaldehyde **9** (9.71g, 48.3 mmol), K_2_CO_3_ (13.36 g, 96.7 mmol), and KI (160 mg, 0.97 mmol) were dissolved in DMF (50 mL) under N_2,_ and then benzyl bromide (6.33 mL, 53.2 mmol) was added to the resulting solution at rt. The solution was then heated to 80 °C and stirred for 3 h. The resulting solution was allowed to cool to rt before the solids were removed by filtration. EtOAc (100 mL) and H_2_O (50 mL) were added to the filtrate, and a precipitate was formed which was filtered off and air-dried to give **10** (13.14 g, 93%) as a tan solid, mp 98–99 °C; IR (ATR) 3034, 2836, 1676 (C=O str), 1591, 1498, 1489, 1279, 1258, 1214, 1191, 1042, 995, 981, 911, 899, 811 cm^–1^; ^1^H NMR (300 MHz, DMSO-*d*_6_): δ 9.84 (s, 1H, C*H*O), 8.11 (d, *J* = 2.0 Hz, 1H, Ar), 7.91 (dd, *J* = 8.5, 2.0 Hz, 1H, Ar), 7.53–7.31 (m, 6H, Ar), 5.33 (s, 2H, OC*H*_2_); ^13^C NMR (75.5 MHz, DMSO-*d*_6_): δ 190.8 (CH), 159.3 (C), 136.0 (C), 134.2 (CH), 131.3 (CH), 130.9 (C), 128.8 (CH), 128.4 (CH), 127.7 (CH), 114.3 (CH), 112.1 (C), 70.7 (CH_2_). Spectroscopic data are consistent with those reported in the literature.^27^

#### 2-(Benzyloxy)-5-formylbenzonitrile (**11**)

4-(Benzyloxy)-3-bromobenzaldehyde **10** (13.06 g, 45 mmol) and CuCN (5.64 g, 63 mmol) were dissolved in DMF (90 mL) under N_2_, and the resulting solution was stirred and heated at 130 °C for 16 h. The solution was allowed to cool to rt, CH_2_Cl_2_ (100 mL) and aqueous ammonium hydroxide (30% w/w, 50 mL) were added, and the mixture was filtered. The layers were separated, and the organic layer was washed with H_2_O (5 × 50 mL) and brine (100 mL), dried (Na_2_SO_4_), and evaporated under reduced pressure to give **11** (8.39 g, 79%) as an off-white solid, mp 114–115 °C; IR (ATR) 3064, 2874, 2232 (C≡N str), 1680 (C=O str), 1600, 1500, 1451, 1381, 1312, 1292, 1276, 1217, 1161, 1107, 1019, 926, 906, 824 cm^–1^; ^1^H NMR (300 MHz, CDCl_3_): δ 9.89 (d, *J* = 0.5 Hz, 1H, C*H*O), 8.12 (dd, *J* = 2.0, 0.5 Hz, 1H, Ar), 8.03 (dd, *J* = 9.0, 2.0 Hz, 1H, Ar), 7.48–7.35 (m, 5H, Ar), 7.15 (d, *J* = 9.0 Hz, 1H, Ar), 5.32 (s, 2H, OCH_2_); ^13^C NMR (75.5 MHz, CDCl_3_): δ 188.9 (CH), 164.4 (C), 136.1 (CH), 135.6 (CH), 134.7 (C), 130.0 (C), 129.1 (CH), 128.8 (CH), 127.2 (CH), 115.2 (C), 113.3 (CH), 103.7 (C), 71.5 (CH_2_). Spectroscopic data are consistent with those reported in the literature.^27^

#### 5-Formyl-2-hydroxybenzonitrile (**12**)

2-(Benzyloxy)-5-formylbenzonitrile **11** (8.35 g, 35.2 mmol) and palladium on carbon (10% w/w, 430 mg) were stirred in THF (33 mL) at rt, and the vessel was backfilled with H_2_ (4 times) and stirred at rt for 16 h. The resulting suspension was filtered over Celite, and the filter cake was washed with EtOAc. The filtrate was evaporated under reduced pressure to give **12** (4.012 g, 77%) as an off-white solid, mp 214–215 °C (decomposition); IR (ATR) 3171 (O–H str), 2871, 2233 (C≡N), 1679 (C=O), 1584, 1505, 1440, 1367, 1296, 1254, 1105, 955, 841 cm^–1^; ^1^H NMR (300 MHz, DMSO-*d*_6_): δ 9.78 (s, 1H, C*H*O), 8.17 (d, *J* = 2.0 Hz, 1H, Ar), 7.98 (dd, *J* = 8.5, 2.0 Hz, 1H, Ar), 7.13 (d, *J* = 8.5 Hz, 1H, Ar); ^13^C NMR (75.5 MHz, DMSO-*d*_6_): δ 190.7 (CH), 165.6 (C), 137.3 (CH), 135.3 (CH), 128.7 (C), 117.3 (CH), 116.5 (C), 100.1 (C). Spectroscopic data are consistent with those reported in the literature.^27^

#### *tert*-Butyl (3-Cyano-4-hydroxybenzyl)­carbamate (**13**)

5-Formyl-2-hydroxybenzonitrile **12** (147 mg, 1 mmol) and *tert*-butyl carbonate (351 mg, 3 mmol) were dissolved in CH_2_Cl_2_ (2 mL) at rt before MeCN (6 mL), and then triethylsilane (0.479 mL, 3 mmol) and TFA (0.15 mL, 2 mmol) were added dropwise sequentially. The resulting solution was stirred at rt for 16 h. Saturated NaHCO_3(aq)_ (10 mL) was then added, the layers were separated, and the aqueous layer was extracted with CH_2_Cl_2_ (3 × 10 mL). The combined organic layers were washed with brine (10 mL), dried (Na_2_SO_4_), and evaporated under reduced pressure to give the crude product. Purification by flash column chromatography on silica with petroleum ether/EtOAc (60:40) as eluent gave **13** (196 mg, 79%) as an off-white solid, mp 148–150 °C; *R*_F_ (petroleum ether/EtOAc 60:40) 0.2; IR (ATR) 3369 (N–H str), 3244 (O–H str), 2981, 2944, 2236 (C≡N str), 1680 (C=O str), 1612, 1506, 1432, 1393, 1318, 1278, 1252, 1231, 1157, 1111, 895, 847 cm^–1^; ^1^H NMR (300 MHz, (acetone-*d*_6_): δ 9.72 (br s, 1H, O*H*), 7.48 (d, *J* = 2.0 Hz, 1H, Ar), 7.44 (dd, *J* = 8.5, 2.0 Hz, 1H, Ar), 7.02 (d, *J* = 8.5 Hz, 1H, Ar), 6.51 (br s, 1H, CH_2_N*H*), 4.20 (d, *J* = 6.5 Hz, 2H, C*H*_2_NH), 1.41 (s, 9H, C­(C*H*_3_)_3_); ^13^C NMR (75.5 MHz, acetone-*d*_6_): δ 159.5 (C), 156.8 (C), 134.6 (CH), 133.4 (C), 132.8 (CH), 117.2 (C), 117.1 (CH), 100.4 (C), 79.0 (C), 43.6 (CH_2_), 28.6 (CH_3_).

#### *tert*-Butyl (4-Hydroxy-3-isobutyrylbenzyl)­carbamate (**14**)

*tert*-Butyl (3-cyano-4-hydroxybenzyl)­carbamate **13** (124 mg, 0.5 mmol) was dissolved in THF (1 mL) under N_2,_ and the resulting solution was cooled to 0 °C. Then, *i*-PrMgBr (1.4 M, 1.1 mL, 1.52 mmol) was added before the solution was allowed to warm to rt and stirred at rt for 3 h. One M HCl_(aq)_ (1 mL) was then added, and the solution was stirred at rt for a further 30 min. Then, H_2_O (5 mL) was added, the layers were separated, and the organic layer was extracted with EtOAc (3 × 5 mL). The combined organic layers were washed with brine (20 mL) and dried (Na_2_SO_4_) and then evaporated under reduced pressure to give the crude product. Purification by flash column chromatography on silica with petroleum ether/EtOAc (80:20) as eluent gave **14** (100 mg, 68%) as a yellow oil; *R*_F_ (petroleum ether/EtOAc 80:20) 0.2; IR (ATR) 3349 (O–H str), 2975, 2933, 1694 (carbamate C=O str, ketone C=O str), 1638, 1511, 1486, 1386, 1364, 1270, 1247, 1193, 1152, 1099, 1087, 1028, 994 cm^–1^; ^1^H NMR (400 MHz, CDCl_3_): δ 12.46 (s, 1H, O*H*), 7.72 (s, 1H, Ar), 7.39 (dd, *J* = 8.5, 2.0 Hz, 1H, Ar), 6.96 (d, *J* = 8.5 Hz, 1H, Ar), 4.85 (s, 1H, CH_2_N*H*), 4.25 (d, *J* = 6.0 Hz, 2H, C*H*_2_NH), 3.61 (hept, *J* = 7.0 Hz, 1H, C*H*(CH_3_)_2_), 1.46 (s, 9H, C­(CH_3_)_3_), 1.24 (d, *J* = 7.0 Hz, 6H, CH­(C*H*_3_)_2_); ^13^C NMR (101 MHz, CDCl_3_): δ 210.8 (C), 162.6 (C), 156.0 (C), 135.8 (CH), 129.6 (C), 129.1 (CH), 119.2 (CH), 118.0 (C), 79.9 (C), 44.2 (CH_2_), 35.1 (CH), 28.5 (CH_3_), 19.5 (CH_3_).

#### *tert*-Butyl ((3-Isopropyl-2-(4-methylbenzoyl)­benzofuran-5-yl)­methyl)­carbamate (**15**)

*tert*-Butyl (4-hydroxy-3-isobutyrylbenzyl)­carbamate **14** (90 mg, 0.31 mmol), 2-bromo-1-(*p*-tolyl)­ethan-1-one (79 mg, 0.37 mmol), K_2_CO_3_ (85 mg, 0.61 mmol), and 4 Å molecular sieves were stirred in DMF (1 mL) at 60 °C under N_2_ for 16 h. The resulting solution was allowed to cool to rt, and then H_2_O (5 mL) was added. The solution was extracted with EtOAc (3 × 5 mL), and the combined organic layers were washed with H_2_O (5 mL) and brine (15 mL), dried (MgSO_4_), and evaporated under reduced pressure to give the crude product. Purification by flash column chromatography on silica with petroleum ether/EtOAc (90:10) as eluent gave **15** (86 mg, 68%) as a waxy solid; *R*_F_ (petroleum ether/EtOAc) 0.2; IR (ATR) 3349 (N–H str), 2972, 2929, 2872, 1699 (carbamate C=O, ketone C=O), 1641, 1606, 1553, 1505, 1462, 1365, 1286, 1265, 1166, 1054, 935, 912 cm^–1^; ^1^H NMR (400 MHz, CDCl_3_): δ 7.93 (d, *J* = 8.0 Hz, 2H, Ar), 7.79 (s, 1H, Ar), 7.47 (d, *J* = 8.5 Hz, 1H, Ar), 7.38 (d, *J* = 8.5 Hz, 1H, Ar), 7.30 (d, *J* = 8.0 Hz, 2H, Ar), 5.07 (s, 1H, CH_2_N*H*), 4.42 (d, *J* = 6.0 Hz, 2H, C*H*_2_NH), 3.97 (hept, *J* = 7.0 Hz, 1H, C*H*(CH_3_)_2_), 2.44 (s, 3H, CC*H*_3_), 1.49–1.45 (m, 15H, CH­(C*H*_3_)_2_ + C­(C*H*_3_)_3_); ^13^C NMR (101 MHz, CDCl_3_): δ 186.2 (C), 156.0 (C), 154.0 (C), 147.6 (C), 143.6 (C), 135.6 (C), 135.4 (C), 134.1 (C), 130.1 (CH), 129.1 (CH), 127.4 (C + CH), 122.0 (CH), 112.7 (CH), 79.7 (C), 44.8 (CH_2_), 28.5 (CH_3_), 25.5 (CH), 22.2 (CH_3_), 21.8 (CH_3_).

#### Ethyl 2-(((3-Iso-propyl-2-(4-methylbenzoyl)­benzofuran-5-yl)­methyl)­amino)-2-oxoacetate (**7h**)

F_3_CCO_2_H (1 mL) was added dropwise to a stirred solution of *tert*-Butyl ((3-isopropyl-2-(4-methylbenzoyl)­benzofuran-5-yl)­methyl)­carbamate **15** (80 mg, 0.2 mmol) in CH_2_Cl_2_ (1 mL) at rt under N_2,_ and the resulting solution was stirred at rt for 2 h. Then, the resulting solution was evaporated to dryness under reduced pressure, and CH_2_Cl_2_ (1 mL) and TEA (100 μL, 0.8 mmol) were added. The resulting solution was cooled to 0 °C, and ethyl oxalyl chloride (27 μL, 0.24 mmol) was added dropwise, and the resulting solution was stirred at 0 °C for 5 min before being allowed to warm to rt and stirred at rt for 2 h. CH_2_Cl_2_ (5 mL) was then added, and the resulting solution was washed with H_2_O (2 × 5 mL), dried (MgSO_4_), and evaporated under reduced pressure to give the crude product. Purification by flash column chromatography on silica with petroleum ether/EtOAc (80:20) as eluent gave product **7h** as a colorless oil (34 mg, 43%); *R*_F_ 0.2 (petroleum ether/EtOAc 80:20); IR (film) 3316 (N–H str), 2967, 2927, 2872, 1734 (C=O str), 1690 (C=O str), 1642 (C=O str), 1607, 1555, 1463, 1367, 1299, 1266, 1211, 1095, 1055, 1020, 974, 911, 876, 832 cm^–1^; ^1^H NMR (300 MHz, CDCl_3_): δ 7.92 (d, *J* = 8.0 Hz, 2H, Ar), 7.81 (d, *J* = 1.5 Hz, 1H), 7.52 (br s, 1H, N*H*), 7.50 (d, *J* = 8.5 Hz, 1H, Ar), 7.40 (dd, *J* = 8.5, 1.5 Hz, 1H, Ar), 7.31 (d, *J* = 8.0 Hz, 2H, Ar), 4.63 (d, *J* = 6.0 Hz, 2H, C*H*_2_NH), 4.35 (q, *J* = 7.0 Hz, 2H, OC*H*_2_CH_3_), 3.96 (sept, *J* = 7.0 Hz 1H, C*H*(CH_3_)_2_), 2.45 (s, 3H, CC*H*_3_), 1.47 (d, *J* = 7.0 Hz, 6H, CH­(C*H*_3_)_2_), 1.38 (t, *J* = 7.0 Hz, 3H, OCH_2_C*H*_3_); ^13^C NMR (75.5 MHz, CDCl_3_): δ 186.2 (C), 160.7 (C), 156.6 (C), 154.2 (C), 147.8 (C), 143.8 (C), 135.5 (C), 135.4 (C), 131.8 (C), 130.1 (CH), 129.1 (CH), 127.9 (CH), 127.6 (C), 123.0 (CH), 113.1 (CH), 63.5 (CH_2_), 44.2 (CH_2_), 25.5 (CH), 22.2 (CH_3_), 21.9 (CH_3_), 14.1 (CH_3_).

#### 2-(((3-Isopropyl-2-(4-methylbenzoyl)­benzofuran-5-yl)­methyl)­amino)-2-oxoacetic Acid (**SY009**)

A solution of ethyl 2-(((3-iso-propyl-2-(4-methylbenzoyl)­benzofuran-5-yl)­methyl)­amino)-2-oxoacetate **7h** (43 mg, 0.11 mmol) and NaOH (8 mg, 0.21 mmol) in MeOH/THF (1:1, 4 mL) was stirred at rt for 20 min. The solution was then acidified with 1 M HCl_(aq)_ (3 mL). Water (10 mL) was added, and organics were extracted with CH_2_Cl_2_ (3 × 10 mL). The organic layers were combined, dried (MgSO_4_), and evaporated under reduced pressure to give product **SY009** as an off-white solid (43 mg, 85%); mp 138–141 °C; IR (solid) 3283 (N–H str), 2966, 2871, 1760 (C=O str), 1686 (C=O str), 1636 (C=O str), 1607, 1549, 1464, 1409, 1355, 1312, 1286, 1268, 1237, 1186, 1174, 1094, 1054, 1009, 973, 908, 875, 834, 810 cm^–1^; ^1^H NMR (300 MHz, acetone-*d*_6_): δ 8.90 (br s, 1H, N*H*), 8.04 (s, 1H, Ar), 7.93 (d, *J* = 8.0 Hz, 2H, Ar), 7.56 (m, 2H, Ar), 7.38 (d, *J* = 8.0 Hz, 2H, Ar), 4.66 (d, *J* = 6.5 Hz, 2H, C*H*_2_NH), 3.96 (sept, *J* = 7.0 Hz, 1H, C*H*(CH_3_)_2_), 2.44 (s, 3H, CCH_3_), 1.47 (d, *J* = 7.0 Hz, 6H, CH­(C*H*_3_)_2_); ^13^C NMR (75.5 MHz, acetone-*d*_6_): δ 186.3 (C), 166.9 (C), 161.8 (C), 154.7 (C), 148.3 (C), 144.5 (C), 136.3 (C), 135.7 (C), 134.4 (C), 130.7 (CH), 129.8 (CH), 128.8 (CH), 127.9 (C), 123.4 (CH), 113.2 (CH), 44.2 (CH_2_), 26.1 (CH), 22.2 (CH_3_), 21.6 (CH_3_); MS (ESI) *m*/*z*: 424 [(M + 2Na)^+^, 100], 402 [(M + Na)^+^, 57], 380 [(M + H)^+^, 20], 142 [(BF + Na)^+^, 86]; HRMS (ESI) *m*/*z*: C_22_H_21_NO_5_ ([M + H]^+^) calcd for 380.1493; found, 380.1506.

#### *tert*-Butyl (4-Hydroxy-3-propionylbenzyl)­carbamate (**16**)

EtMgBr (0.55 M, 33 mL, 17.8 mmol) was added to *tert*-butyl (3-cyano-4- hydroxybenzyl)­carbamate **13** (1.45 g, 5.84 mmol) in THF (12 mL) at 0 °C under N_2_, and the resulting solution was warmed to rt and stirred at rt for 3 h. Then, 1 M HCl_(aq)_ (30 mL) was added, and the solution was stirred for a further 30 min. H_2_O (30 mL) was added, and the layers were separated. The aqueous layer was extracted with EtOAc (3 × 30 mL), and the combined organic layers were washed with brine (50 mL), dried (Na_2_SO_4_), and evaporated under reduced pressure to give the crude product. Purification by flash column chromatography on silica with petroleum ether/EtOAc (80:20) as eluent gave **16** (707 mg, 43%) as a white solid, mp 90–92 °C; *R*_F_ (petroleum ether/EtOAc) 0.2; IR (ATR) 3384 (N–H str), 2977, 2940, 1686 (carbamate C=O, ketone C=O), 1639, 1521, 1486, 1408, 1390, 1312, 1276, 1246, 1209, 1167, 940, 895, 859, 821 cm^–1^; ^1^H NMR (300 MHz, CDCl_3_): δ 12.28 (s, 1H, O*H*), 7.68 (d, *J* = 2.0 Hz, 1H, Ar), 7.38 (dd, *J* = 8.5, 2.0 Hz, 1H, Ar), 6.94 (d, *J* = 8.5 Hz, 1H, Ar), 4.85 (s, 1H, CH_2_N*H*), 4.24 (d, *J* = 6.0 Hz, 2H, C*H*_2_NH), 3.03 (q, *J* = 7.5 Hz, 2H, C*H*_2_CH_3_), 1.46 (s, 9H, C­(C*H*_3_)_3_), 1.23 (t, *J* = 7.5 Hz, 3H, CH_2_C*H*_3_); ^13^C NMR (75.5 MHz, CDCl_3_): δ 207.1 (C), 161.8 (C), 156.0 (C), 135.7 (CH), 129.6 (C), 129.0 (CH), 119.1 (C), 118.9 (CH), 79.9 (C), 44.2 (CH_2_), 31.7 (CH_2_), 28.5 (CH_3_), 8.3 (CH_3_).

#### *tert*-Butyl ((3-Ethyl-2-(4-methylbenzoyl)­benzofuran-5-yl)­methyl)­carbamate (**17**)

*tert*-Butyl (4-hydroxy-3-propionylbenzyl)­carbamate **16** (682 mg, 2.44 mmol), 2-bromo-1-(*p*-tolyl)­ethan-1-one (624 mg, 2.93 mmol), and K_2_CO_3_ (674 mg, 4.88 mmol) were suspended in DMF (25 mL) and stirred at 60 °C under N_2_ for 2 h. The resulting solution was allowed to cool to rt then H_2_O (25 mL) was added. The resulting solution was extracted with EtOAc (3 × 25 mL), and the combined organic layers were washed with H_2_O (25 mL) and brine (50 mL), dried (MgSO_4_), and evaporated under reduced pressure to give a residue, to which *p*-toluenesulfonic acid (46 mg, 0.24 mmol) was added, and the mixture dissolved in toluene (25 mL). The resulting solution was stirred and heated under reflux under a Dean–Stark trap for 16 h, before being allowed to cool to rt. Then, the solution was diluted with CH_2_Cl_2_ (30 mL), washed with saturated NaHCO_3(aq)_ (2 × 30 mL) and brine (30 mL), dried (MgSO_4_), and evaporated under reduced pressure to give the crude product. Purification by flash column chromatography on silica with petroleum ether/EtOAc (80:20) as eluent gave **17** (661 mg, 69%) as an off-white solid, mp 121–123 °C; *R*_F_ (petroleum ether/EtOAc 80:20) 0.2; IR (ATR) 3354 (N–H str), 2973, 2930, 1675 (carbamate C=O, ketone C=O), 1631, 1605, 1554, 1498, 1496, 1456, 1365, 1338, 1315, 1276, 1246, 1183, 1157, 1111, 1053, 986, 909, 879, 839, 810 cm^–1^; ^1^H NMR (300 MHz, CDCl3): δ 8.00 (d, *J* = 8.5 Hz, 2H, Ar), 7.63 (dd, *J* = 1.5, 0.5 Hz, 1H, Ar), 7.48 (dd, *J* = 8.5, 0.5 Hz, 1H, Ar), 7.40 (dd, *J* = 8.5, 1.5 Hz, 1H, Ar), 7.32 (d, *J* = 8.5 Hz, 2H, Ar), 4.98 (s, 1H, CH_2_N*H*), 4.43 (d, *J* = 6.0 Hz, 2H, C*H*_2_NH), 3.12 (q, *J* = 7.5 Hz, 2H, C*H*_2_CH_3_), 2.45 (s, 3H, CC*H*_3_), 1.48 (s, 9H, C­(C*H*_3_)_3_), 1.34 (t, *J* = 7.5 Hz, 3H, CH_2_C*H*_3_); ^13^C NMR (75.5 MHz, CDCl_3_): δ 185.5 (C), 156.0 (C), 153.8 (C), 148.6 (C), 143.6 (C), 135.3 (C), 134.4 (C), 132.5 (C), 130.1 (CH), 129.1 (CH), 128.7 (C), 127.9 (CH), 120.3 (CH), 112.5 (CH), 79.8 (C), 44.9 (CH_2_), 28.5 (CH_3_), 21.8 (CH_3_), 18.0 (CH_2_), 14.3 (CH_3_).

#### Diethyl-2,2′-(((3-ethyl-2-(4-methylbenzoyl)­benzofuran-5-yl)­methyl)­azanediyl)­bis­(2-oxoacetate) (**18**)

*tert*-Butyl ((3-ethyl-2-(4-methylbenzoyl)­benzofuran-5-yl)­methyl)­carbamate **17** (658 mg, 1.67 mmol) was dissolved in CH_2_Cl_2_ (8.4 mL) and TFA (8.3 mL, 109 mmol) and then dropwise added. The resulting solution was stirred at rt for 1 h and then evaporated under reduced pressure, and the residue was redissolved in CH_2_Cl_2_ (8.4 mL). Triethylamine (0.93 mL, 6.7 mmol) was added, and the resulting solution was cooled to 0 °C before ethyl oxalyl chloride (0.44 mL, 4 mmol) was added dropwise, and the solution was stirred for 5 min. Then, the resulting solution was allowed to warm to rt and stirred for a further 1 h. CH_2_Cl_2_ (10 mL) was added, and the resulting organic solution was washed with H_2_O (2 × 10 mL), dried (MgSO_4_), and evaporated under reduced pressure to give the crude product. Purification by flash column chromatography on silica with petroleum ether/EtOAc (70:30) as eluent gave **18** (235 mg, 86%) as a waxy solid; *R*_F_ (petroleum ether/EtOAc 70:30) 0.2; IR (ATR) 3349, 2978, 2937, 1738 (ester C=O), 1690 (carbamate C=O, ketone C=O), 1640, 1606, 1557, 1371, 1314, 1295, 1210, 1182, 1160, 1056, 1019, 907, 834 cm^–1^; ^1^H NMR (300 MHz, CDCl_3_): δ 7.98 (d, *J* = 8.5 Hz, 2H, Ar), 7.71 (s, 1H, Ar), 7.48 (s, 1H, Ar), 7.48 (s, 1H, Ar), 7.32 (d, *J* = 8.5 Hz, 2H, Ar), 5.14 (s, 2H, C*H*_2_N), 4.31 (q, *J* = 7.0 Hz, 4H, OC*H*_2_CH_3_), 3.11 (q, *J* = 7.5 Hz, 2H, C*H*_2_CH_3_), 2.45 (s, 3H CC*H*_3_), 1.38–1.30 (m, 9H, OCH_2_C*H*_3_ + CH_2_C*H*_3_); ^13^C NMR (75.5 MHz, CDCl_3_): δ 185.5 (C), 161.7 (C), 160.3 (C), 154.1 (C), 148.7 (C), 143.7 (C), 135.3 (C), 132.4 (C), 130.6 (C), 130.1 (CH), 129.2 (CH), 128.8 (C), 128.4 (CH), 121.7 (CH), 112.8 (CH), 63.9 (CH_2_), 48.3 (CH_2_), 21.9 (CH_3_), 18.0 (CH_2_), 14.3 (CH_3_), 13.9 (CH_3_).

#### 2-(((3-Ethyl-2-(4-methylbenzoyl)­benzofuran-5-yl)­methyl)­amino)-2-oxoacetic Acid (**SY007**)

A solution of LiOH (62 mg, 2.58 mmol) in H_2_O (5 mL) was added to a stirred solution of diethyl-2,2′-(((3-ethyl-2-(4-methylbenzoyl)­benzofuran-5-yl)­methyl)­azanediyl)­bis­(2-oxoacetate) **18** (255 mg, 0.52 mmol) in THF (5 mL) at rt, and the resulting solution was stirred at rt for 1 h. Then, the resulting solution was acidified with 1 M HCl_(aq)_ (15 mL), and EtOAc (10 mL) and H_2_ (10 mL) were added. The layers were separated, and the aqueous layer was extracted with EtOAc (2 × 20 mL); the combined organic layers were dried (MgSO_4_) and evaporated under reduced pressure to give product **SY007** as an off-white solid (41 mg, 98%); mp 194–196 °C; IR (solid) 3275 (N–H str), 2981, 2454, 1763 (C=O str), 1682 (C=O str), 1634 (C=O str), 1608, 1591, 1554, 1464, 1410, 1362, 1345, 1318, 1291, 1277, 1244, 1231, 1186, 1161, 1113, 1082, 1057, 1002, 972, 954, 902, 876, 839, 801 cm^–1^; ^1^H NMR (300 MHz, acetone-*d*_6_): δ 8.85 (br s, 1H, N*H*), 8.01 (d, *J* = 8.0 Hz, 2H, Ar), 7.88 (s, 1H, Ar), 7.58 (m, 2H, Ar), 7.39 (d, *J* = 8.0 Hz, 2H, Ar), 4.67 (d, *J* = 6.5 Hz, 2H, C*H*_2_NH), 3.13 (q, *J* = 7.5 Hz, 2H, CC*H*_2_CH_3_), 2.45 (s, 3H, CC*H*_3_), 1.31 (t, *J* = 7.5 Hz, 3H, CCH_2_C*H*_3_); ^13^C NMR (101 MHz, acetone-*d*_6_): δ 185.6 (C), 164.1 (C), 161.8 (C), 154.5 (C), 149.2 (C), 144.4 (C), 136.2 (C), 134.8 (C), 132.7 (C), 130.6 (CH), 129.9 (CH), 129.3 (CH), 129.1 (C), 121.7 (CH), 113.1 (CH), 44.0 (CH_2_), 21.6 (CH_3_), 18.3 (CH_2_), 14.6 (CH_3_); MS (ESI) *m*/*z*: 753 [(MM + Na)^+^, 50], 388 [(M + Na)^+^, 100], 366 [(M + H)^+^, 60], 288 [(M–CH2NHCOCOOH + Na)^+^, 60]; HRMS (ESI) *m*/*z*: C_21_H_20_NO_5_ ([M + H]^+^) calcd for 366.1336; found, 366.1335.

#### 1-(3-((*tert*-Butyldimethylsilyl)­oxy)­phenyl)­ethan-1-one (**20**)

3-Hydroxyacetophenone **19** (681 mg, 5 mmol) and imidazole (851 mg, 12.5 mmol) were dissolved in DMF (5.5 mL), and the resulting solution was cooled to 0 °C. *tert*-Butyldimethylsilyl chloride (904 mg, 6 mmol) was added, and the solution was stirred at 0 °C for 15 min before warming to rt and stirring for a further 1 h. The solution was then diluted with EtOAc (100 mL), and the layers were separated. The organic layer was washed with 1 M NaOH_(aq)_ (50 mL), H_2_O (3 × 50 mL), and brine (50 mL), dried (MgSO_4_), and evaporated under reduced pressure to give the crude product. Purification by flash column chromatography on silica with petroleum ether/EtOAc (90:10) as eluent gave product **20** as a colorless oil (1.220 g, 97%); *R*_F_ 0.3 (petroleum ether/EtOAc 90:10); IR (ATR) 3168, 2955, 2857, 1661 (C=O str), 1575, 1489, 1428, 1363, 1296, 1254, 1215, 1081, 996, 960, 911, 865, 791, 707, 680, 610 cm^–1^; ^1^H NMR (300 MHz, CDCl_3_): δ 7.54 (ddd, *J* = 7.5, 1.5, 1.0 Hz, 1H, Ar), 7.41 (dd, *J* = 2.5, 1.5 Hz, 1H, Ar), 7.32 (t, *J* = 8.0 Hz, 1H, Ar), 7.04 (ddd, *J* = 8.0, 2.5, 1.0 Hz, 1H, Ar), 2.58 (s, 3H, COC*H*_3_), 1.00 (s, 9H, (CC*H*_3_)_3_), 0.22 (s, 6H, Si­(C*H*_3_)_2_); ^13^C NMR (75.5 MHz, CDCl_3_): δ 198.0 (C), 156.1 (C), 138.8 (C), 129.7 (CH), 125.1 (CH), 121.7 (CH), 119.6 (CH), 26.9 (CH_3_), 25.8 (CH_3_), 18.4 (C), −4.3 (CH_3_). Spectroscopic data are consistent with those reported in the literature.^41^

#### 2-Bromo-1-(3-((*tert*-butyldimethylsilyl)­oxy)­phenyl)­ethan-1-one (**21**)

1-(3-((*tert*-Butyldimethylsilyl)­oxy)­phenyl)­ethan-1-one **20** (1.21 g, 4.84 mmol) was dissolved in CH_2_Cl_2_ (10 mL), and the resulting solution was cooled to 0 °C. DIPEA (1.05 mL, 6.06 mmol) and TMSOTf (1.05 mL, 5.81 mmol) were added, and the mixture was stirred at 0 °C for 30 min. Then, NBS (1.034 g, 5.81 mmol) was added, and the solution was allowed to warm to rt and stirred for a further 4 h. The solution was evaporated under reduced pressure to give the crude product. Purification by flash column chromatography on silica with petroleum ether/EtOAc (95:5) as eluent gave product **21** as a yellow oil (1.498 g, 94%); *R*_F_ 0.2 (petroleum ether/EtOAc 95:5); IR (ATR) 3340, 2927, 2856, 1676 (C=O str), 1580, 1514, 1443, 1397, 1318, 1257, 1211, 1164, 1101, 834, 771, 618 cm^–1^; ^1^H NMR (300 MHz, CDCl_3_): δ 7.57 (ddd, *J* = 7.5, 1.5, 1.0 Hz, 1H, Ar), 7.44 (ddd, *J* = 2.5, 1.5, 0.5 Hz, 1H, Ar), 7.35 (td, *J* = 8.0, 0.5 Hz, 1H, Ar), 7.08 (ddd, *J* = 8.0, 2.5, 1.0 Hz, 1H, Ar), 4.42 (s, 2H, COC*H*_2_Br), 1.00 (s, 9H, CC*H*_3_), 0.23 (s, 6H, Si­(C*H*_3_)_2_); ^13^C NMR (75.5 MHz, CDCl_3_): δ 191.2 (C), 156.3 (C), 135.5 (C), 130.0 (CH), 126.1 (CH), 122.1 (CH), 120.3 (CH), 31.2 (CH_2_), 25.8 (CH_3_), 18.4 (C), −4.3 (CH_3_).

#### *tert*-Butyl ((2-(3-Hydroxybenzoyl)-3-isopropylbenzofuran-5-yl)­methyl)­carbamate (**22**)

*tert*-Butyl­(4-hydroxy-3-isobutyrylbenzyl)­carbamate **14** (293 mg, 1 mmol), 2-bromo-1-(3-((*tert*-butyldimethylsilyl)­oxy)­phenyl)­ethan-1-one **21** (395 mg, 1.2 mmol), K_2_CO_3_ (276 mg, 2 mmol), and 4 Å molecular sieves were stirred in DMF (3 mL) at 60 °C under N_2_ for 16 h. The resulting solution was allowed to cool to rt, and then H_2_O (5 mL) was added. The solution was then extracted with EtOAc (3 × 5 mL), and the combined organic layers were washed with H_2_O (5 mL) and brine (15 mL), dried (MgSO_4_), and evaporated under reduced pressure to give the crude product. Purification by flash column chromatography on silica with petroleumether/EtOAc (80:20) as eluent gave **22** as a yellow oil (209 mg, 51%); *R*_F_ 0.2 (petroleum ether/EtOAc 80:20); IR (ATR) 3338 (O–H str), 2973, 2932, 1680 (C=O str), 1642 (C=O str), 1551, 1515, 1447, 1366, 1289, 1270, 1160, 1054, 857, 753, 736 cm^–1^; ^1^H NMR (300 MHz, CDCl_3_): δ 7.67 (dd, *J* = 1.5, 1.0 Hz, 1H, Ar), 7.47–7.40 (m, 2H, Ar), 7.33 (d, *J* = 8.5 Hz, 1H, Ar), 7.24 (dd, *J* = 8.5, 1.5 Hz, 2H, Ar), 7.24 (t, *J* = 8.0 Hz, 1H, Ar), 7.01 (ddd, *J* = 8.0, 2.5, 1.0 Hz, 1H, Ar), 6.97 (s, 1H, O*H*), 4.99 (s, 1H, CH_2_N*H*), 4.32 (d, *J* = 6.0 Hz, 2H, C*H*_2_NH), 3.84 (hept, *J* = 7.0 Hz, 1H, C*H*(CH_3_)_2_), 1.39 (s, 9H, C­(C*H*_3_)_3_), 1.36 (d, *J* = 7.0 Hz, 6H, CH­(C*H*_3_)_2_); ^13^C NMR (75.5 MHz, CDCl_3_): δ 186.6 (C), 156.4 (C), 156.4 (C), 154.2 (C), 147.3 (C), 139.3 (C), 136.4 (C), 133.8 (C), 129.6 (CH), 127.6 (CH), 127.4 (C), 122.1 (CH), 122.0 (CH), 120.3 (CH), 116.6 (CH), 112.8 (CH), 80.2 (C), 44.9 (CH_2_), 28.5 (CH_3_), 25.6 (CH), 22.2 (CH_3_).

#### Ethyl 2-(((2-(3-Hydroxybenzoyl)-3-isopropylbenzofuran-5-yl)­methyl)­amino)-2- oxoacetate (**23**)

*tert*-Butyl­((2-(3-hydroxybenzoyl)-3-isopropylbenzofuran-5-yl)­methyl)­carbamate **22** (200 mg, 0.49 mmol) was dissolved in CH_2_Cl_2_ (2.5 mL), and TFA (2.4 mL, 31.4 mmol) was added dropwise. The resulting solution was stirred at rt for 1 h and then evaporated under reduced pressure, and the residue was redissolved in CH_2_Cl_2_ (2.5 mL). Triethylamine (0.27 mL, 1.96 mmol) was added, and the resulting solution was cooled to 0 °C before ethyl oxalyl chloride (66 μL, 0.59 mmol) was added dropwise and the solution was stirred for 5 min. Then, the resulting solution was allowed to warm to rt and stirred for a further 1 h. CH_2_Cl_2_ (10 mL) was added, and the resulting organic solution was washed with H_2_O (2 × 10 mL), dried (MgSO_4_), and evaporated under reduced pressure to give the crude product. Purification by flash column chromatography on silica with petroleum ether/EtOAc (60:40) as eluent gave **23** as a yellow solid (145 mg, 73%); *R*_F_ 0.2 (petroleum ether/EtOAc 60:40); mp 80–82 °C; IR (ATR) 3307 (O–H str), 2968, 1738 (C=O str), 1681 (C=O str), 1640 (C=O str), 1582, 1545, 1445, 1368, 1288, 1208, 1094, 1055, 1018, 995, 856, 805, 770, 752, 685 cm^–1^; ^1^H NMR (300 MHz, CDCl_3_): δ 7.79 (d, *J* = 1.0 Hz, 1H, Ar), 7.59 (t, *J* = 6.0 Hz, 1H, N*H*CH_2_), 7.55–7.50 (m, 2H, Ar), 7.45 (d, *J* = 8.5 Hz, 1H, Ar), 7.39–7.29 (m, 2H, Ar), 7.13 (ddd, *J* = 8.0, 2.5, 1.0 Hz, 1H, Ar), 6.96 (s, 1H, O*H*), 4.60 (d, *J* = 6.0 Hz, 2H, NHC*H*_2_), 4.34 (q, *J* = 7.0 Hz, 2H, CH_3_C*H*_2_), 3.90 (hept, *J* = 7.0 Hz, 1H, C*H*(CH_3_)_2_), 1.44 (d, *J* = 7.0 Hz, 6H, CH­(C*H*_3_)_2_), 1.35 (t, *J* = 7.0 Hz, 3H, C*H*_3_CH_2_); ^13^C NMR (75.5 MHz, CDCl_3_): δ 186.5 (C), 160.6 (C), 156.9 (C), 156.4 (C), 154.3 (C), 147.5 (C), 139.2 (C), 136.2 (C), 131.6 (C), 129.6 (CH), 128.1 (CH), 127.5 (C), 123.0 (CH), 122.1 (CH), 120.4 (CH), 116.6 (CH), 113.2 (CH), 63.6 (CH_2_), 44.3 (CH_2_), 25.6 (CH), 22.2 (CH_3_), 14.0 (CH_3_).

#### 2-(((2-(3-Hydroxybenzoyl)-3-isopropylbenzofuran-5-yl)­methyl)­amino)-2-oxoacetic Acid (**DM357**)

A solution of ethyl 2-(((2-(3-hydroxybenzoyl)-3-isopropylbenzofuran-5-yl)­methyl)­amino)-2-oxoacetate **23** (124 mg, 0.3 mmol), and NaOH (36 mg, 0.9 mmol) in MeOH/THF (1:1, 6 mL) was stirred at rt for 20 min. The solution was then acidified with 1 M HCl_(aq)_, and water (10 mL) was added. The resulting solution was extracted with EtOAc (3 × 10 mL), and the combined organic layers were dried (Na_2_SO_4_) and evaporated under reduced pressure to give product **DM357** as an off-white solid (110 mg, 96%); mp 173 °C (decomposition); IR (ATR) 3308 (O–H str), 2977, 2957, 2931, 1763 (C=O str), 1660 (C=O str), 1630 (C=O str), 1592, 1543, 1464, 1444, 1355, 1311, 1289, 1270, 1219, 855, 804, 747, 682 cm^–1^; ^1^H NMR (300 MHz, acetone-*d*_6_): δ 8.77 (s, 1H, O*H*), 7.91 (s, 1H, Ar), 7.46–7.32 (m, 4H, Ar), 7.26 (t, *J* = 8.0 Hz, 1H, Ar) 7.01 (ddd, *J* = 8.0, 2.5, 1.0 Hz, 1H, Ar), 4.52 (d, *J* = 6.0 Hz, 2H, NHC*H*_2_), 3.80 (hept, *J* = 7.0 Hz, 1H, C*H*(CH_3_)_2_), 1.33 (d, *J* = 7.0 Hz, 6H, CH­(C*H*_3_)_2_); ^13^C NMR (101 MHz, acetone-*d*_6_): δ 186.6 (C), 158.2 (C), 154.7 (C), 148.2 (C), 140.2 (C), 135.9 (C), 134.5 (C), 130.3 (CH), 128.9 (CH), 127.8 (C), 123.5 (CH), 121.9 (CH), 120.8 (CH), 116.8 (CH), 113.2 (CH), 44.3 (CH2), 26.1 (CH), 22.2 (CH_3_); MS (ESI) *m*/*z*: 404 [(M + Na)^+^, 0.5], 382 [(M + H)^+^, 3]; HRMS (ESI) *m*/*z*: C_23_H_23_NO_7_ ([M + H]^+^) calcd for 382.1285; found, 382.1280.

#### (*E*)-4-(5-Bromo-2-hydroxyphenyl)­but-3-en-2-one (**25**)

5-Bromosalicaldehyde **24** (2.01 g, 10 mmol) was dissolved in acetone (15 mL, 200 mmol) and 1.2 M NaOH_(aq)_ (166 mL, 200 mmol), and the resulting solution was stirred at rt for 1 h. Then the solution was acidified with 6 M HCl_(aq)_ to pH 1. The resulting precipitate was filtered off, washed with cold H_2_O (100 mL), and then air-dried overnight to give **25** as a yellow solid (2.24 g, 93%); mp 155 °C (decomposition); IR (ATR) 3054 (O–H str), 1627 (C=O str), 1586, 1572, 1494, 1399, 1360, 1283, 1253, 1214, 1187, 1134, 983, 822 cm^–1^; ^1^H NMR (300 MHz, CDCl_3_): δ 7.73 (d, *J* = 16.5 Hz, 1H, C*H*CHCO), 7.58 (d, *J* = 2.5 Hz, 1H, Ar), 7.51 (s, 1H, O*H*), 7.34 (dd, *J* = 8.5, 2.5 Hz, 1H, Ar), 7.05 (d, *J* = 16.5 Hz, 1H, CHC*H*CO), 6.83 (d, *J* = 8.5 Hz, 1H, Ar), 2.43 (s, 3H, COC*H*3); ^13^C NMR (75.5 MHz, CDCl_3_): δ 200.7 (C), 154.9 (C), 138.9 (CH), 134.4 (CH), 132.1 (CH), 128.7 (CH), 123.7 (C), 118.4 (CH), 112.9 (C), 27.4 (CH3).

#### (*E*)-4-(5-Bromo-2-(2-oxo-2-(*p*-tolyl)­ethoxy)­phenyl)­but-3-en-2-one (**26**)

(*E*)-4-(5-Bromo-2-hydroxyphenyl)­but-3-en-2-one **25** (2.23 g, 9.25 mmol), 2-bromo-4′-methylacetophenone (2.96 g, 13.9 mmol), and K_2_CO_3_ (2.55 g, 18.5 mmol) were dissolved in acetone (83 mL) and then stirred at rt for 1 h. The resulting solution was then evaporated under reduced pressure, and the residue was dissolved in CH_2_Cl_2_ (50 mL). H_2_O (50 mL) was added, the layers were separated, and the aqueous layer was extracted with CH_2_Cl_2_ (3 × 50 mL). The combined organic layers were washed with brine (100 mL), dried (MgSO_4_), and evaporated under reduced pressure to give the crude product. Purification by flash column chromatography on silica with petroleum ether/EtOAc (80:20 → 0:100) as eluent gave product **26** as a colorless oil (3.45 g, 99%); *R*_F_ 0.2 (petroleum ether/EtOAc 80:20); IR (ATR) 2921, 1698 (C=O str), 1607, 1485, 1408, 1360, 1284, 1229, 1182, 1131, 972, 810 cm^–1^; ^1^H NMR (300 MHz, CDCl_3_): δ 7.91–7.82 (m, 3H, Ar + C*H*CHCO), 7.67 (d, *J* = 2.5 Hz, 1H, Ar), 7.39 (dd, *J* = 9.0, 2.5 Hz, 1H, Ar), 7.31 (d, *J* = 8.0 Hz, 2H, Ar), 6.79 (d, *J* = 16.5 Hz, 1H, CHC*H*CO), 6.68 (d, *J* = 9.0 Hz, 1H, Ar), 5.35 (s, 2H, OC*H*_2_), 2.44 (s, 3H, COCH_3_), 2.36 (s, 3H, CC*H*_3_); ^13^C NMR (75.5 MHz, CDCl_3_): δ 198.8 (C), 193.0 (C), 155.9 (C), 145.4 (C), 136.9 (CH), 134.0 (CH), 131.9 (C), 131.3 (CH), 129.8 (CH), 129.2 (CH), 128.2 (CH), 126.2 (C), 114.3 (CH), 114.3 (C), 71.2 (CH_2_), 27.5 (CH_3_), 21.9 (CH_3_).

#### 6-Bromo-3-methyl-1-(*p*-tolyl)­benzofuro­[2,3-*c*]­pyridine (**27**)

(*E*)-4-(5-Bromo-2-(2-oxo-2-(*p*-tolyl)­ethoxy)­phenyl)­but-3-en-2-one **26** (3.42 g, 9.16 mmol) and NH_4_OAc (7.06 g, 92 mmol) were dissolved in THF (92 mL), and the resulting solution was stirred at 60 °C for 24 h. The solution was allowed to cool to rt and evaporated under reduced pressure. H_2_O (30 mL) was then added to the residue, and the resulting aqueous solution was extracted with CH_2_Cl_2_ (3 × 30 mL). The combined organic layers were dried (Na_2_SO_4_) and evaporated under reduced pressure to give the crude product. Purification by flash column chromatography on silica with petroleum ether/EtOAc (90:10) as eluent gave product **27** as a white solid (1.711 g, 53%); *R*_F_ 0.2 (petroleum ether/EtOAc 90:10); mp 136–138 °C; IR (ATR) 3087, 2918, 1624, 1579, 1516, 1456, 1407, 1380, 1266, 1190, 1054, 1042, 1024, 852, 823, 805, 774, 692, 652, 625 cm^–1^; ^1^H NMR (400 MHz, CDCl_3_): δ 8.30 (d, *J* = 8.0 Hz, 2H, Ar), 8.10 (d, *J* = 2.0 Hz, 1H, Ar), 7.67 (dd, *J* = 9.0, 2.0 Hz, 1H, Ar), 7.61–7.51 (m, 2H, Ar), 7.37 (d, *J* = 7.5 Hz, 2H, Ar), 2.76 (s, 3H, CC*H*_3_), 2.46 (s, 3H, CC*H*_3_); ^13^C NMR (101 MHz, CDCl_3_): δ 155.6 (C), 151.8 (C), 149.3 (C), 141.9 (C), 139.5 (C), 133.4 (C), 132.5 (CH), 131.6 (C), 129.5 (CH), 128.7 (CH), 124.8 (CH), 124.6 (C), 116.1 (C), 114.1 (CH), 112.9 (CH), 24.7 (CH_3_), 21.6 (CH_3_).

#### 3-Methyl-1-(*p*-tolyl)­benzofuro­[2,3-*c*]­pyridine-6-carbonitrile (**28**)

A solution of 6-bromo-3-methyl-1-(*p*-tolyl)­benzofuro­[2,3-*c*]­pyridine **27** (1.46 g, 4.15 mmol), Zn­(CN)_2_ (584 mg, 4.98 mmol), Pd_2_dba_3_ (266 mg, 0.29 mmol), and dppf (322 mg, 0.58 mmol) in DMF (28 mL) was stirred and heated at 120 °C for 16 h. Then, the solution was allowed to cool to rt before being filtered and washed with EtOAc (30 mL). Dilute NH_4_OH_(aq)_ (10% v/v, 20 mL) was added to the filtrate, and the layers were separated. The organic layer was washed with dilute NH_4_OH_(aq)_ (10% v/v, 20 mL) and brine (20 mL), dried (MgSO_4_), and evaporated under reduced pressure to give the crude product. Purification by flash column chromatography on silica eluting with petroleum ether/EtOAc (80:20) gave product **28** as an off-white solid (1.162 g, 94%); *R*_F_ 0.2 (petroleum ether/EtOAc 80:20); mp 210 °C (decomposition); IR (ATR) 3079, 3036, 2917, 2857, 2224 (C≡N str), 1626, 1579, 1515, 1470, 1427, 1408, 1379, 1333, 1277, 1251, 1185, 1125, 1041, 1024, 907, 852, 827, 815, 772, 745, 711, 660, 638 cm^–1^; ^1^H NMR (300 MHz, CDCl_3_): δ 8.34–8.26 (m, 3H, Ar), 7.86 (dd, *J* = 8.5, 1.5 Hz, 1H, Ar), 7.75 (dd, *J* = 8.5, 0.5 Hz, 1H, Ar), 7.64 (d, *J* = 0.5 Hz, 1H, Ar), 7.38 (d, *J* = 8.0 Hz, 2H, Ar), 2.78 (d, *J* = 0.5 Hz, 3H, CC*H*_3_), 2.46 (s, 3H, CC*H*_3_); ^13^C NMR (75.5 MHz, CDCl_3_): δ 158.6 (C), 152.7 (C), 149.4 (C), 142.3 (C), 139.9 (C), 133.09 (CH), 133.06 (C), 131.1 (C), 129.6 (CH), 128.7 (CH), 126.9 (CH), 123.7 (C), 118.9 (C), 113.9 (CH), 113.0 (CH), 107.4 (C), 24.7 (CH_3_), 21.6 (CH_3_).

#### Ethyl 2-(((3-Methyl-1-(*p*-tolyl)­benzofuro­[2,3-*c*]­pyridin-6-yl)­methyl)­amino)-2-oxoacetate (**29**)

A solution of 3-methyl-1-(*p*-tolyl)­benzofuro­[2,3-*c*]­pyridine-6-carbonitrile **28** (1.16 mg, 3.89 mmol) in THF (15 mL) was added dropwise to a stirred suspension of LiAlH_4_ (888 mg, 23.4 mmol) in THF (15 mL) at 0 °C. The resulting solution was allowed to warm to rt before stirring and heating at reflux for 16 h. The solution was then cooled to 0 °C and quenched sequentially with water (0.88 mL), 1 M NaOH_(aq)_ (1.76 mL), and water (0.88 mL), dried (Na_2_SO_4_), filtered through Celite, washed with CH_2_Cl_2_/MeOH (95:5), and evaporated under reduced pressure to give the crude product. Purification by flash column chromatography on silica with CH_2_Cl_2_ → CH_2_Cl_2_/MeOH (98:2) → CH_2_Cl_2_/MeOH/NH_4_OH_(aq)_ (96:3:1) as eluent gave the impure amine (183 mg) that was used without further purification.

Ethyl oxalyl chloride (82 μL, 0.73 mmol) was added dropwise to a stirred solution of crude amine (183 mg, 0.6 mmol) and TEA (167 μL, 1.2 mmol) in CH_2_Cl_2_ (6 mL) at 0 °C. The resulting solution was allowed to warm to rt and stirred for a further 2 h. Then, CH_2_Cl_2_ (10 mL) and water (10 mL) were added, and the layers were separated. The organic layer was dried (MgSO_4_) and evaporated under reduced pressure to give the crude product. Purification by flash column chromatography on silica with petroleum ether/EtOAc (60:40) as eluent gave product **29** as an off-white solid (72 mg, 4.6%); *R*_F_ 0.2 (petroleum ether/EtOAc 60:40); mp 203–204 °C (decomposed); IR (ATR) 3243 (N–H str), 3040, 2986, 2925, 1750 (C=O str), 1685 (C=O str), 1634, 1580, 1538, 1514, 1431, 1338, 1297, 1184, 1050, 1015, 830, 817, 678, 659 cm^–1^; ^1^H NMR (300 MHz, CDCl_3_): δ 8.32 (d, *J* = 8.0 Hz, 2H, Ar), 7.94 (dd, *J* = 2.0, 0.5 Hz, 1H, Ar), 7.66–7.62 (m, 2H, Ar), 7.53 (dd, *J* = 8.5, 2.0 Hz, 1H, Ar), 7.37 (d, *J* = 8.0 Hz, 2H, Ar), 4.69 (d, *J* = 6.0 Hz, 2H, C*H*_2_NH), 4.38 (q, *J* = 7.0 Hz, 2H, OC*H*_2_CH_3_), 2.77 (d, *J* = 0.5 Hz, 3H, CC*H*_3_), 2.45 (s, 3H, CC*H*_3_), 1.41 (t, *J* = 7.0 Hz, 3H, OCH_2_C*H*_3_); ^13^C NMR (75.5 MHz, CDCl_3_): δ 160.8 (C), 156.7 (C), 156.6 (C), 151.7 (C), 149.4 (C), 141.8 (C), 139.4 (C), 133.6 (C), 132.3 (C), 132.1 (C), 129.8 (CH), 129.5 (CH), 128.7 (CH), 123.1 (C), 121.6 (CH), 113.0 (CH), 112.9 (CH), 63.6 (CH_2_), 44.0 (CH_2_), 24.7 (CH_3_), 21.6 (CH_3_), 14.1 (CH_3_).

#### 2-(((3-Methyl-1-(*p*-tolyl)­benzofuro­[2,3-*c*]­pyridin-6-yl)­methyl)­amino)-2-oxoacetic Acid (**DM312**)

A solution of ethyl 2-(((3-methyl-1-(*p*-tolyl)­benzofuro­[2,3-*c*]­pyridin-6-yl)­methyl)­amino)-2-oxoacetate **29** (66 mg, 0.164 mmol) and NaOH (7 mg, 0.32 mmol) in MeOH/THF (1:1, 4 mL) was stirred at rt for 20 min. The solution was then acidified with 1 M HCl_(aq)_ (3 mL). Water (10 mL) was added, and the resulting solution was extracted with CH_2_Cl_2_ (3 × 10 mL). The combined organic layers were dried (MgSO_4_) and evaporated under reduced pressure to give product **DM312** as an amorphous solid (45 mg, 74%); IR (ATR) 3305 (O–H str, N–H str), 2941, 2831, 1634 (C=O str), 1517, 1515, 1431, 1380, 1246, 1192, 1117, 1019, 894, 867, 826 cm^–1^; ^1^H NMR (300 MHz, methanol-*d*_4_): δ 8.10–8.01 (m, 3H, Ar), 7.81 (d, *J* = 0.5 Hz, 1H, Ar), 7.63 (dd, *J* = 8.5, 2.0 Hz, 1H, Ar), 7.58 (dd, *J* = 8.5, 0.5 Hz, 1H, Ar), 7.38 (d, *J* = 8.0 Hz, 2H, Ar), 4.59 (s, 2H, C*H*_2_NH), 2.70 (d, *J* = 0.5 Hz, 3H, CCH_3_), 2.45 (s, 3H, CCH_3_); ^13^C NMR (75.5 MHz, methanol-*d*_4_): δ 163.8 (C), 161.9 (C), 158.4 (C), 151.7 (C), 150.0 (C), 141.8 (C), 141.7­(C), 135.9 (C), 135.8 (C), 132.1 (CH), 131.9 (C), 130.4 (CH), 130.0 (CH), 123.0 (C), 122.7 (CH), 115.4 (CH), 113.4 (CH), 44.0 (CH_2_), 22.8 (CH_3_), 21.5 (CH_3_); MS (ESI) *m*/*z*: 375 [(M + H)^+^, 15], 331 [(M–CO_2_ + H)^+^, 100]; HRMS (ESI) *m*/*z*: C_22_H_19_N_2_O_4_ ([M + H]^+^) calcd for 375.1339; found, 375.1330.

### SDS-PAGE and Western Blotting

Protein samples for sodium dodecyl sulfate polyacrylamide gel electrophoresis (SDS-PAGE) and Western blotting were prepared by mixing with SDS Sample Loading Buffer and denatured at 95 °C. The percentage of polyacrylamide gel used varied in accordance with the molecular weight of the proteins, with electrophoresis performed initially at 80 V and subsequently at 130 V. During Western blotting, proteins separated by SDS-PAGE were transferred onto nitrocellulose membranes, which were then blocked using either nonfat dry milk or BSA. This step was followed by an overnight incubation at 4 °C with primary antibodies including total VASP (vasodilator-stimulated phosphoprotein) and phospho-VASP (Ser157) rabbit (to measure PKA activation mAb, Rap1A/Rap1B (26B4) rabbit mAb (for Rap1 activation assays, phospho-STAT3 (Tyr705), and total STAT3 protein (to measure STA3 activation), all from Cell Signaling Technology, Danvers, USA. Subsequently, the membranes were incubated with horseradish peroxidase-conjugated secondary antibodies, specifically anti-Mouse IgG (catalogue number A5278) and anti-Rabbit IgG (catalogue number A6154), from Sigma-Aldrich, St. Louis, USA. After incubation, the membranes were washed and treated with SuperSignal West Pico PLUS Chemiluminescent Substrate for signal detection, captured using a Fusion FX7 camera. Signal intensities were quantified densitometrically using ImageJ software and normalized against a housekeeping protein signal. This methodology not only effectively separates proteins by molecular weight but also enables selective detection and quantification of relative expression levels using specific antibodies.

### Rap1 Activation Assays

In the Rap1 activation assay, U2OS cells expressing EPAC1 or EPAC2 were cultured to 80% confluence, starved for 16 h in medium with reduced FBS, and then treated with various compounds, including the EPAC activators D-007 (EPAC1 activator) and S-220 (EPAC2 activator). Post-treatment, cells were lysed, and Rap1.GTP was isolated using a pull-down technique with GST-RalGDS-RBD immobilized on Glutathione Sepharose 4B beads. The lysates were incubated with GST-RalGDS-RBD to capture active Rap1, followed by washing and separation of the glutathione resin. The recovered Rap1.GTP and input control samples were then denatured, subjected to SDS-PAGE, and analyzed by Western blotting using an anti-Rap1 antibody. This assay effectively quantifies the active GTP-bound form of Rap1, a downstream effector of EPAC1, providing insights into the activation status of EPAC in response to various treatments.

### Fibroblast-to-Myofibroblast Transition Assay

Normal lung fibroblasts (Lonza, CC512) were used for the assay. Each well of a 384-well plate was coated with 20 μL of rat collagen-I (Sigma), incubated for 1 h at room temperature with agitation, and washed thrice with 1× PBS. The plates were left to air-dry. Cells were seeded at a density of 3000 cells per well in 50 μL of FGM-2 medium (Lonza) and incubated in a humidity and temperature-controlled incubator at 37 °C and 5% CO2. 24 h postseeding, the medium was replaced with 50 μL of serum-free FBM medium (Lonza), and cells were incubated overnight under the same incubation conditions. Cells were treated with compounds dissolved in FBM medium supplemented with 0.1% BSA and 200 μM ascorbic acid. The medium was changed to 30 μL of the treatment medium. Compound concentration–response curves (CRCs) were prepared through serial dilution in 100% DMSO, using the automated liquid handler Biomek (Beckman Coulter). An intermediate compound plate was created by adding 500 nL of each CRC point using Echo 650 (LabCyte). To this, 50 μL of treatment medium containing 0.01% Pluronic F127 (Sigma) was added using Multidrop (Thermo). 10 μL from the intermediate plate was then transferred to the assay plate using Viaflo (Integra) and incubated for 1 h. Subsequently, TGFβ was added to reach a final volume of 50 μL per well, and cells were incubated for 72 h. Cells were fixed with −20 °C cold 100% Methanol on ice for 15 min, followed by three washes in 1× PBS. Cells underwent permeabilization in 0.2% Triton-X-100 (Sigma) for 5 min at room temperature. After blocking in PBS 1× + 3% BSA, primary antibodies to αSMA and Collagen I in 1× PBS containing 0.5% BSA were added and incubated overnight at 4 °C. Following three washes, secondary antibodies and cell mask dye in 1× PBS containing 0.5% BSA were applied for 1 h at room temperature. Cells were then stained with Hoechst 33342 for 10 min and washed thrice. Imaging was conducted using the InCell Analyzer 2200 (GE Healthcare) with a 10× objective. The Columbus software (PerkinElmer) performed image segmentation to identify nuclei and individual cells, calculating mean cell fluorescence intensity and cell viability. Viability was expressed as a percentage relative to the control condition. Compound potency was represented as pIC_50_, calculated as −Log IC_50_ M. The assay incorporated QC criteria, including a plate *Z*′ > 0.4. IC50 values were determined using a four-parameter logistic model via Screener software (Genedata).

### Comparative Cytotoxicity Assay

Cytotoxicity in adherent fibroblasts was assessed by quantifying nuclear integrity via Hoechst 33342 staining and automated image analysis. Briefly, following a 72 h compound treatment at 37 °C and 5% CO_2_, the culture medium was aspirated, and cells were fixed in prechilled (−20 °C) 100% methanol for 15 min on ice. Wells were washed three times with 1× PBS using an automated plate washer and then permeabilized in PBS containing 0.2% Triton X-100 for 5 min at room temperature. After a further PBS wash, nuclei were stained with Hoechst 33342 (1:1000 in PBS) for 10 min at room temperature, followed by three additional PBS washes to remove excess dye. Plates were imaged on an InCell Analyzer 2200 (10× objective), capturing the Hoechst channel for nuclear visualization. Image segmentation was performed in Columbus software to identify and count nuclei, yielding absolute cell numbers per well. Cytotoxicity was then expressed as the percentage reduction in nuclear count relative to vehicle-treated controls (0.2% DMSO), with wells showing >50% nuclear loss flagged and excluded from further analysis. This high-content nuclear count assay enabled sensitive detection of compound-induced cytotoxicity alongside phenotypic readouts.

### Cyclic AMP Competition Binding Assays

The 8-NBD-cAMP competition binding assay, integral for assessing the affinity of EPAC binders, utilizes changes in fluorescence intensity to measure the displacement of the fluorescent probe, 8-NBD-cAMP, from EPAC proteins’ cyclic AMP binding sites. Initially, the assay’s reproducibility and quality were verified using control and background samples in Binding Assay Buffer on 96-well plates. Fluorescence intensity was measured post 4 h incubation using a FLUOstar Omega microplate reader at specific wavelengths. The assay’s precision was confirmed by calculating statistical parameters like % CV, S/B, S/N, and *Z*′-factor from repeated experiments. Following this, DM compounds were screened under similar conditions. Dose–response experiments with these compounds were performed against various recombinant EPAC proteins, and IC50 values were determined using nonlinear regression in GraphPad Prism 8, enabling comparison of inhibitory efficacies between compounds and proteins. The assay involved careful preparation of samples with specified concentrations of EPAC proteins, compounds, and 8-NBD-cAMP, ensuring accurate assessment of binding affinities.

### Statistical Analysis

All statistical analyses were performed using GraphPad Prism 8 software. When two means were compared, an unpaired, two-tailed *t*-test was used. When comparing three or more means, one-way ANOVA with Tukey’s post hoc test was performed, preceded by the Brown–Forsythe test for equality of variances. *P*-value thresholds were marked as follows: * represents *p* ≤ 0.05; ** represents *p* ≤ 0.01; *** represents *p* ≤ 0.001. Nonsignificant changes were indicated, ns.

## Supplementary Material







## Notes

Representative HPLC traces of **DM243** and **DM245** and ^1^H and ^13^C NMR spectra of all isolated compounds (PDF)
PDB file of the EPAC1 homology model (PDB)
CSV file containing SMILES and binding and cellular data for final target compounds (CSV)

## List of Abbreviations

Ac: acetyl
aq: aqueous
Boc: *tert*-butoxycarbonyl
cAMP: cyclic adenosine monophosphate
CNDB: cyclic nucleotide binding domain
DAPI: 4′,6-diamidino-2-phenylindole
dba: dibenzylideneacetone
DIPEA: di*iso*propylethylamine
DMF: *N*,*N*-dimethylformamide
dppf: 1,1′-*bis*(diphenylphosphino)­ferrocene
DS: Dean–Stark
EPAC: exchange protein activated by cAMP
FMT: fibroblast-to-myofibroblast transition
GEF: guanine nucleotide exchange factor
GTP: guanosine triphosphate
h: hours
HM: homology model
HUVECs: human vascular endothelial cells
IL-6: interleukin 6
JAK: Janus kinase
MHz: mega Hertz
min: minutes
MS: molecular sieves
NBS: *N*-bromosuccinimide
NMR: nuclear magnetic resonance
PKA: protein kinase A
Rap: Ras-related protein
rt: room temperature
αSMA: α-smooth muscle actin
SOCS3: suppressor of cytokine signaling 3
STAT3: signal transducer and activator of transcription protein 3
TBDMS: *tert*-butyldimethylsilyl
TFA: trifluoroacetic acid
TGF: transforming growth factor
THF: tetrahydrofuran
TLC: thin layer chromatography
Ts: *p*-tolylsulfonyl
UV: ultraviolet
