# Supplementary material for: Development and Evaluation of Benzofuran Oxoacetic Acid Compounds as EPAC1 Activators
Source: J Med Chem. 2026 Mar 14;69(6):6706–35. doi: 10.1021/acs.jmedchem.5c02974 (PMC13036782; doi:10.1021/acs.jmedchem.5c02974)
Supplement: Supplementary file 1 [file jm5c02974_si_001.pdf]

Supporting Information

Development and Evaluation of Benzofuran Oxoacetic Acid Compounds as EPAC1 Activators

David Morgan[1], Jolanta Wiejak[2], Frederick G. Powell[1], Chiara Fitzpatrick[1], Stephen J. Yarwood[2], Graeme Barker[1]**

[1]Institute of Chemical Sciences, Heriot-Watt University, Riccarton, Edinburgh, UK, EH14 4AS

[2]Institute of Biochemistry, Biophysics and Bioengineering, Heriot-Watt University, Riccarton, Edinburgh, UK, EH14 4AS

* **Corresponding Authors:** Dr Stephen Yarwood, email: S.Yarwood@hw.ac.uk

Dr Graeme Barker, email: graeme.barker@h.ac.uk

Table of Contents

HPLC Traces for **DM243** and **DM245** pS3

Copies of ^{1}H and ^{13}C NMR Spectra pS4-S70

HPLC trace for **DM243**:

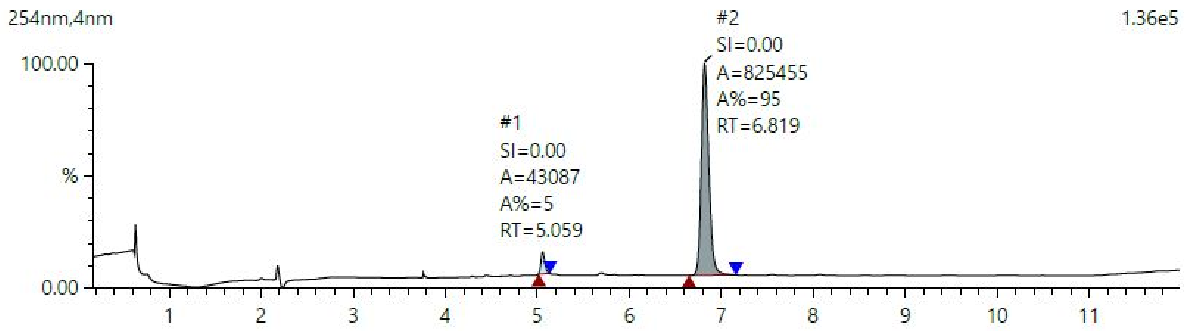

Pk#	RT	Area	Area %	Height	Height %
1	5.059	43,087	4.961	14,933	9.203
2	6.819	825,455	95.039	147,332	90.797
**Totals:**		**868,541**	**100.000**	**162,265**	**100.000**

HPLC trace for **DM245**:

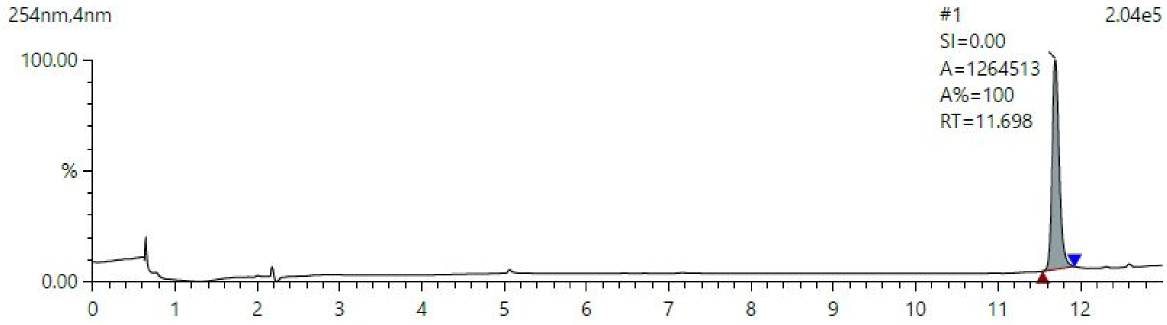

Pk#	RT	Area	Area %	Height	Height %
1	11.698	1,264,513	100.000	210,822	100.000
**Totals:**		**1,264,513**	**100.000**	**210,822**	**100.000**

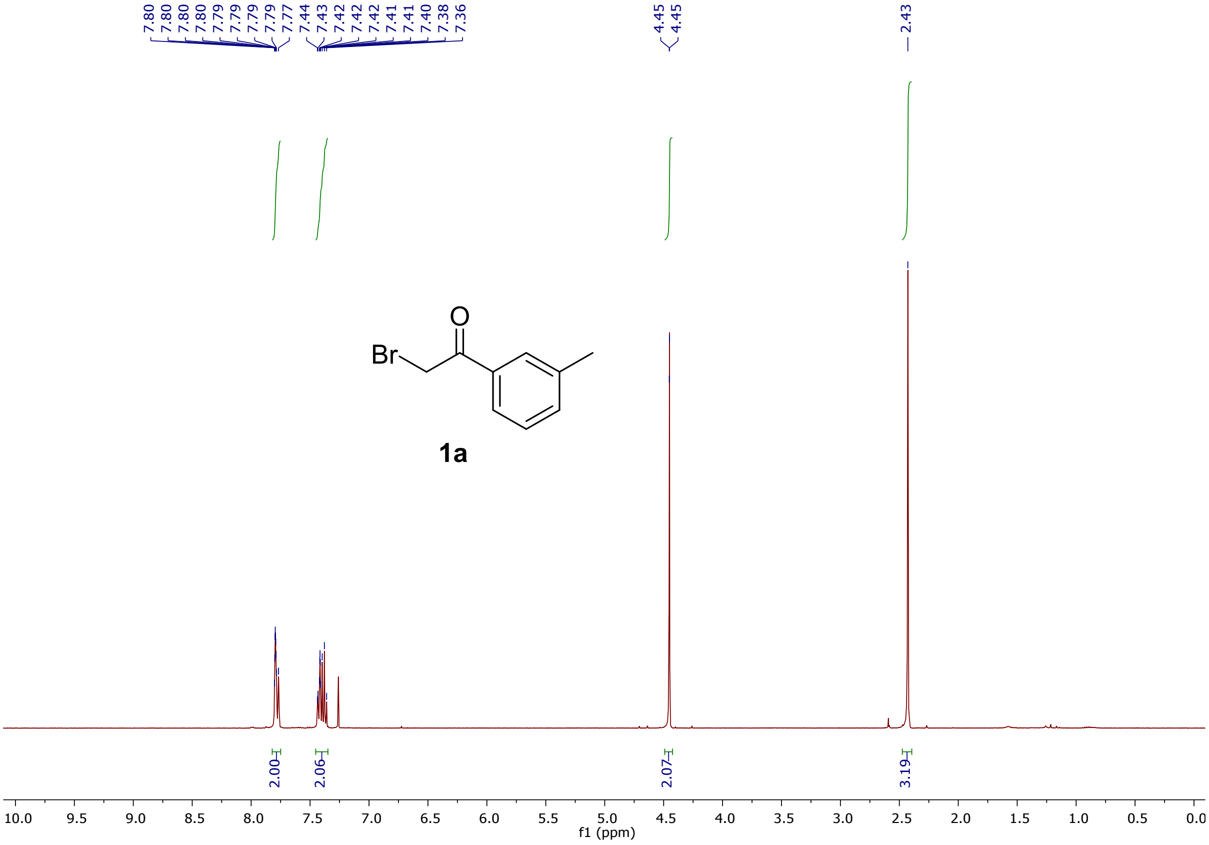
7.80 7.80 7.80 7.80 7.79 7.79 7.79 7.79 7.77 7.44 7.43 7.42 7.42 7.42 7.41 7.41 7.40 7.38 7.36
4.45 4.45
2.43
Br
O
1a
2.00
2.06
2.07
3.19
10.0 9.5 9.0 8.5 8.0 7.5 7.0 6.5 6.0 5.5 5.0 4.5 4.0 3.5 3.0 2.5 2.0 1.5 1.0 0.5 0.0
f1 (ppm)

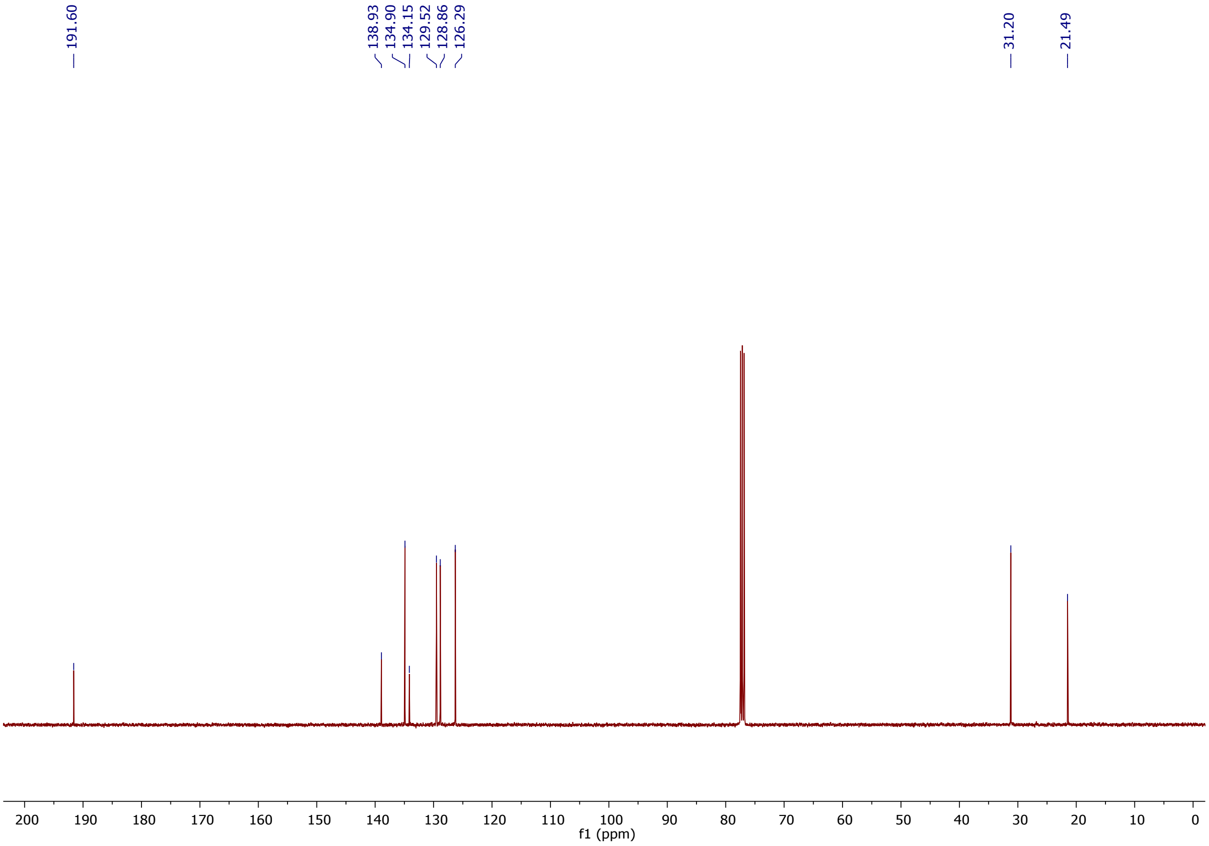
191.60
138.93 134.90 134.15 129.52 128.86 126.29
31.20
21.49
200 190 180 170 160 150 140 130 120 110 100 90 80 70 60 50 40 30 20 10 0
f1 (ppm)

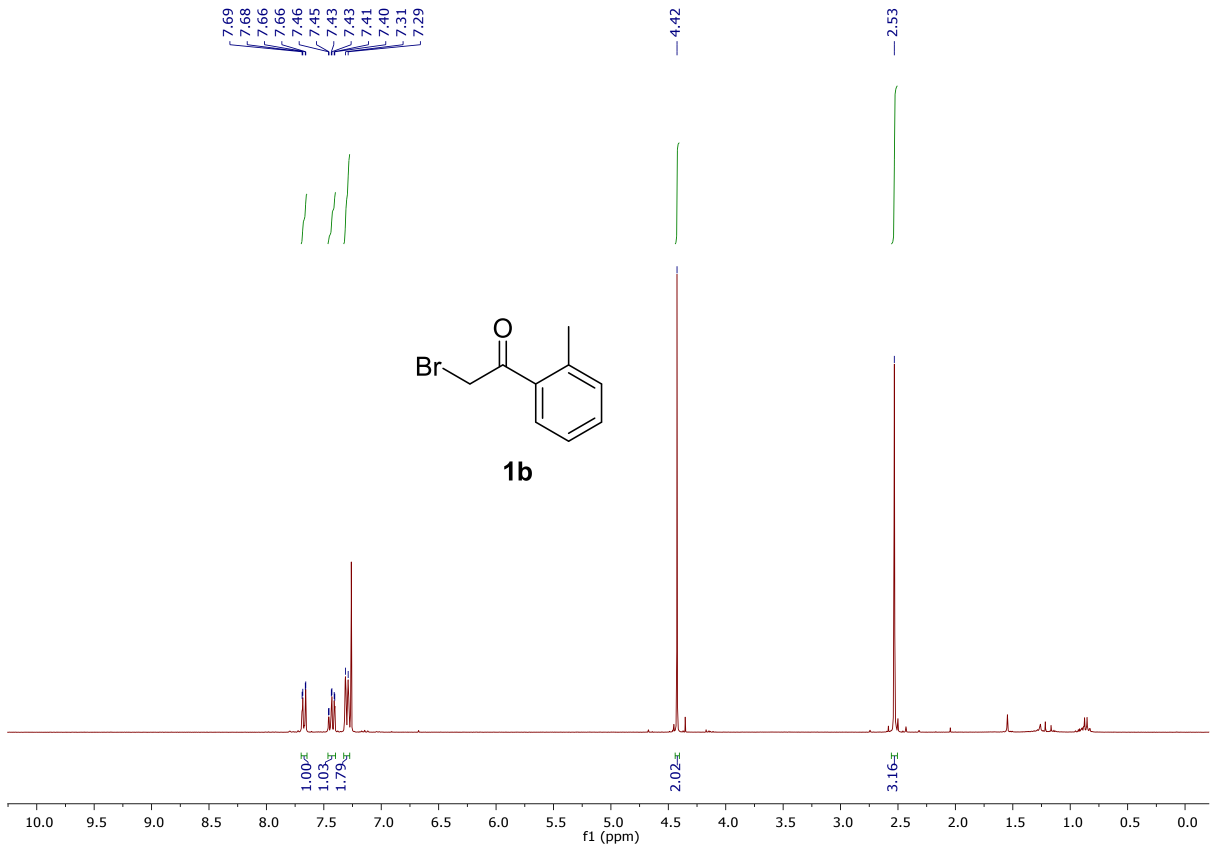
7.69
7.68
7.66
7.66
7.46
7.45
7.43
7.43
7.41
7.40
7.31
7.29
4.42
2.53
Br
O
1b
1.00
1.03
1.79
2.02
3.16
10.0 9.5 9.0 8.5 8.0 7.5 7.0 6.5 6.0 5.5 5.0 4.5 4.0 3.5 3.0 2.5 2.0 1.5 1.0 0.5 0.0
f1 (ppm)

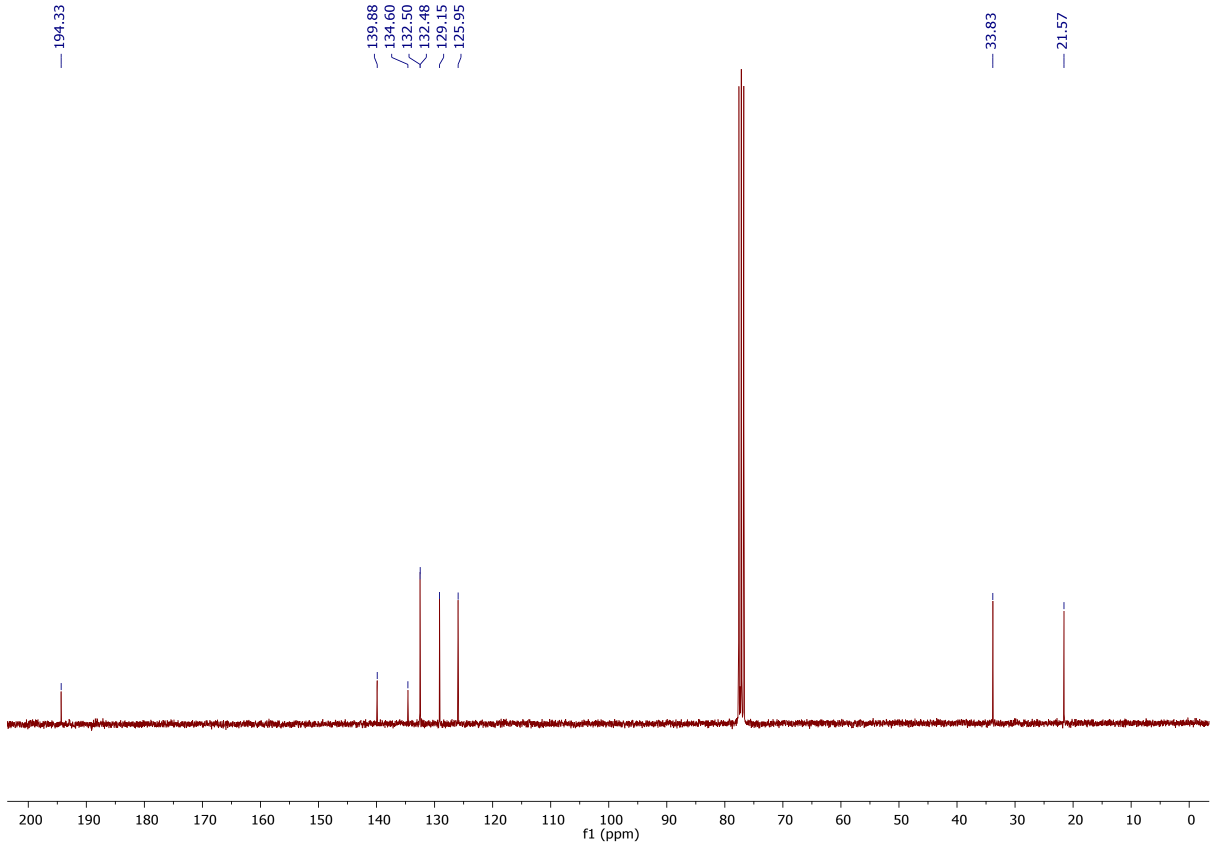
194.33
139.88
134.60
132.50
132.48
129.15
125.95
33.83
21.57
200 190 180 170 160 150 140 130 120 110 100 90 80 70 60 50 40 30 20 10 0
f1 (ppm)

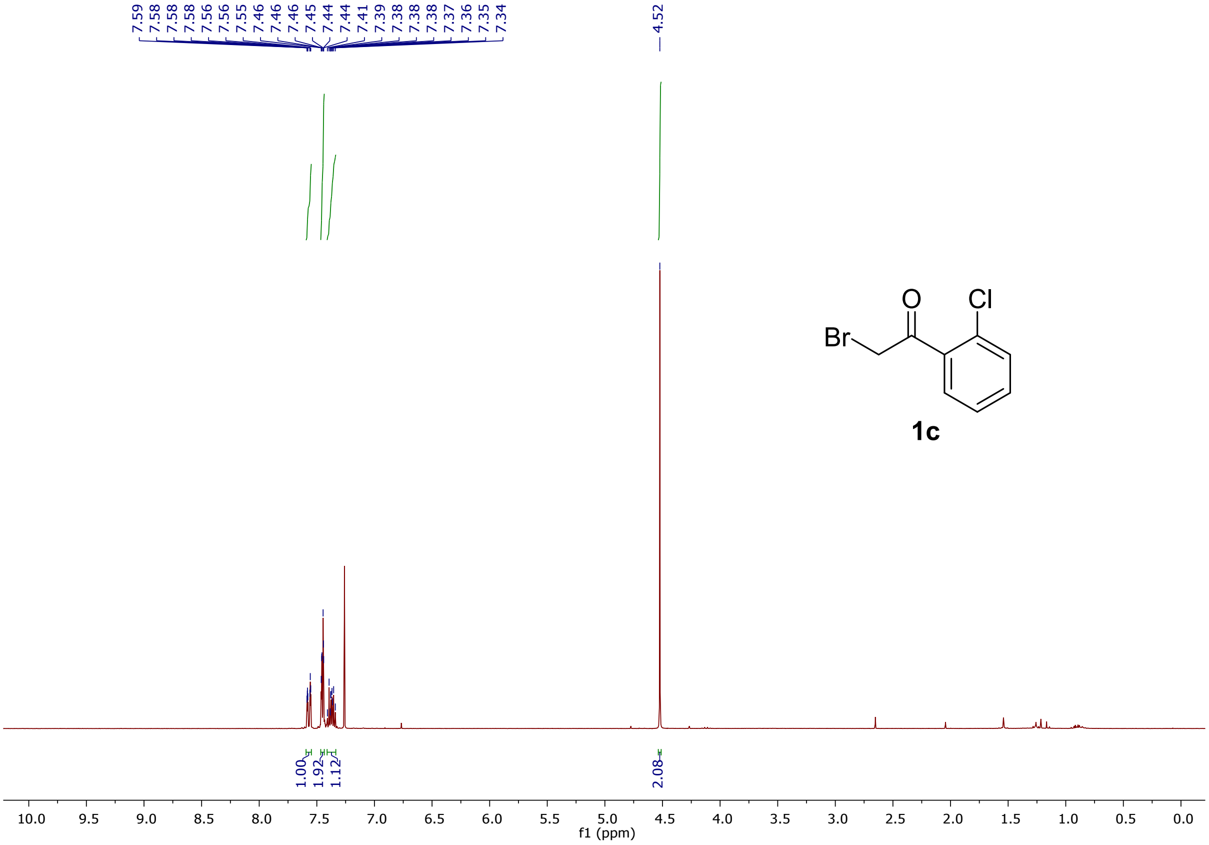
7.59 7.58 7.58 7.58 7.56 7.56 7.55 7.46 7.46 7.45 7.44 7.44 7.41 7.39 7.38 7.38 7.38 7.37 7.36 7.35 7.34
4.52
1.00
1.92
1.12
2.08
10.0 9.5 9.0 8.5 8.0 7.5 7.0 6.5 6.0 5.5 5.0 4.5 4.0 3.5 3.0 2.5 2.0 1.5 1.0 0.5 0.0
f1 (ppm)
Br
O
Cl

1c

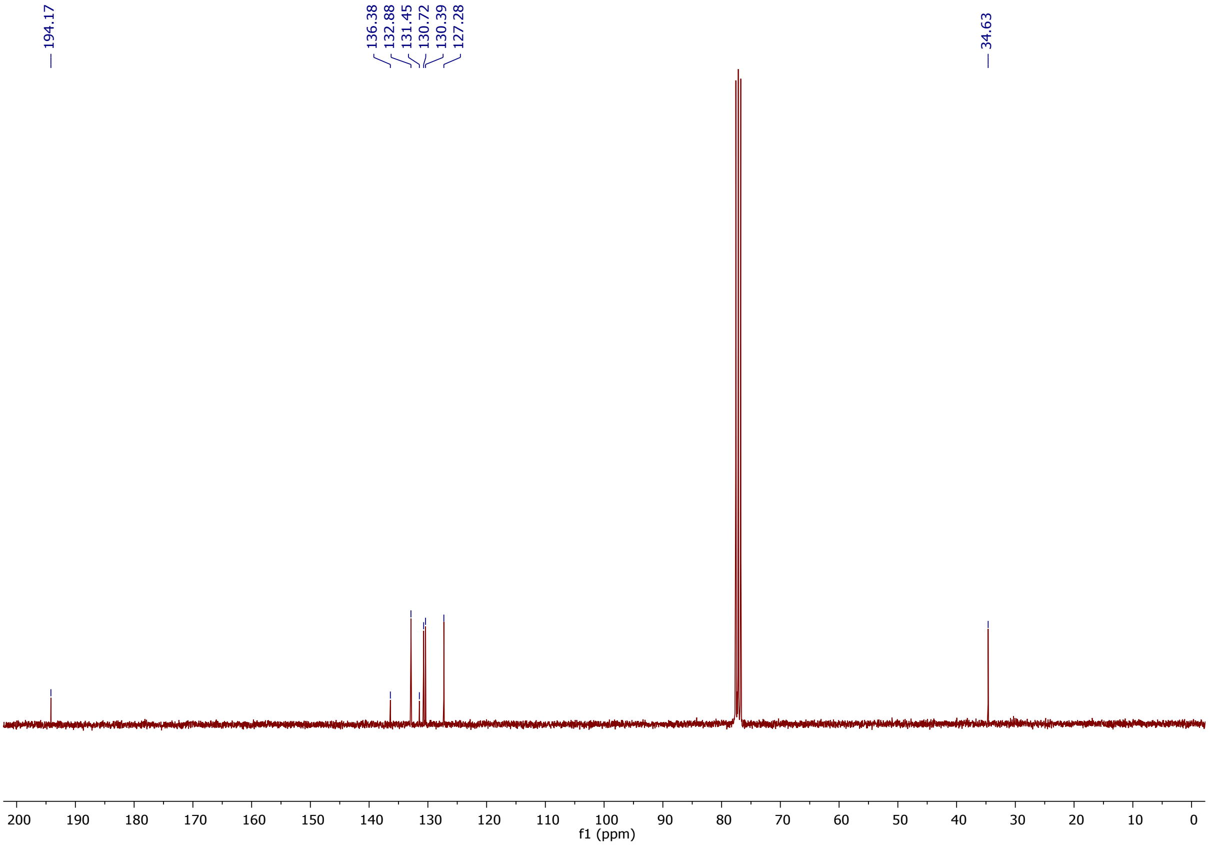
194.17
136.38 132.88 131.45 130.72 130.39 127.28
34.63
200 190 180 170 160 150 140 130 120 110 100 90 80 70 60 50 40 30 20 10 0
f1 (ppm)

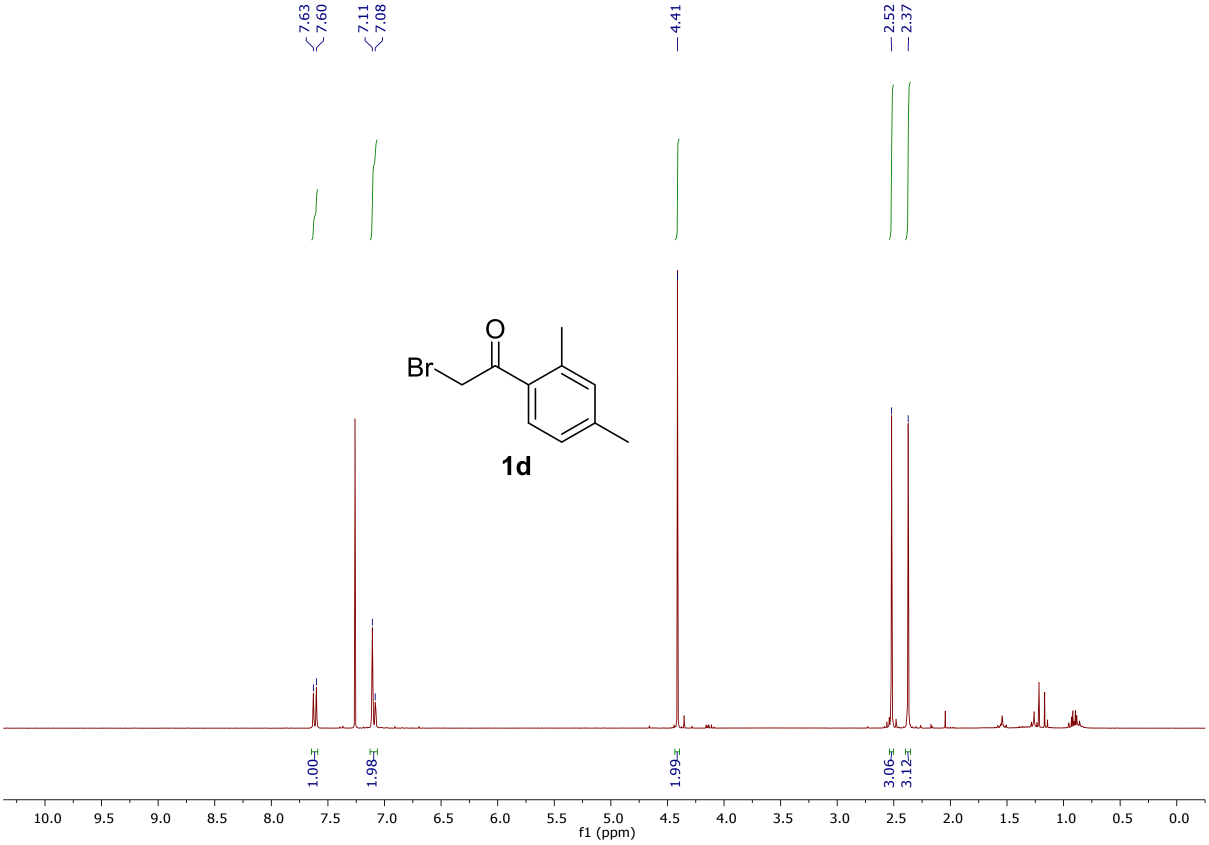
7.63
7.60
7.11
7.08
4.41
2.52
2.37
O
Br
1d
1.00
1.98
1.99
3.06
3.12
10.0 9.5 9.0 8.5 8.0 7.5 7.0 6.5 6.0 5.5 5.0 4.5 4.0 3.5 3.0 2.5 2.0 1.5 1.0 0.5 0.0
f1 (ppm)

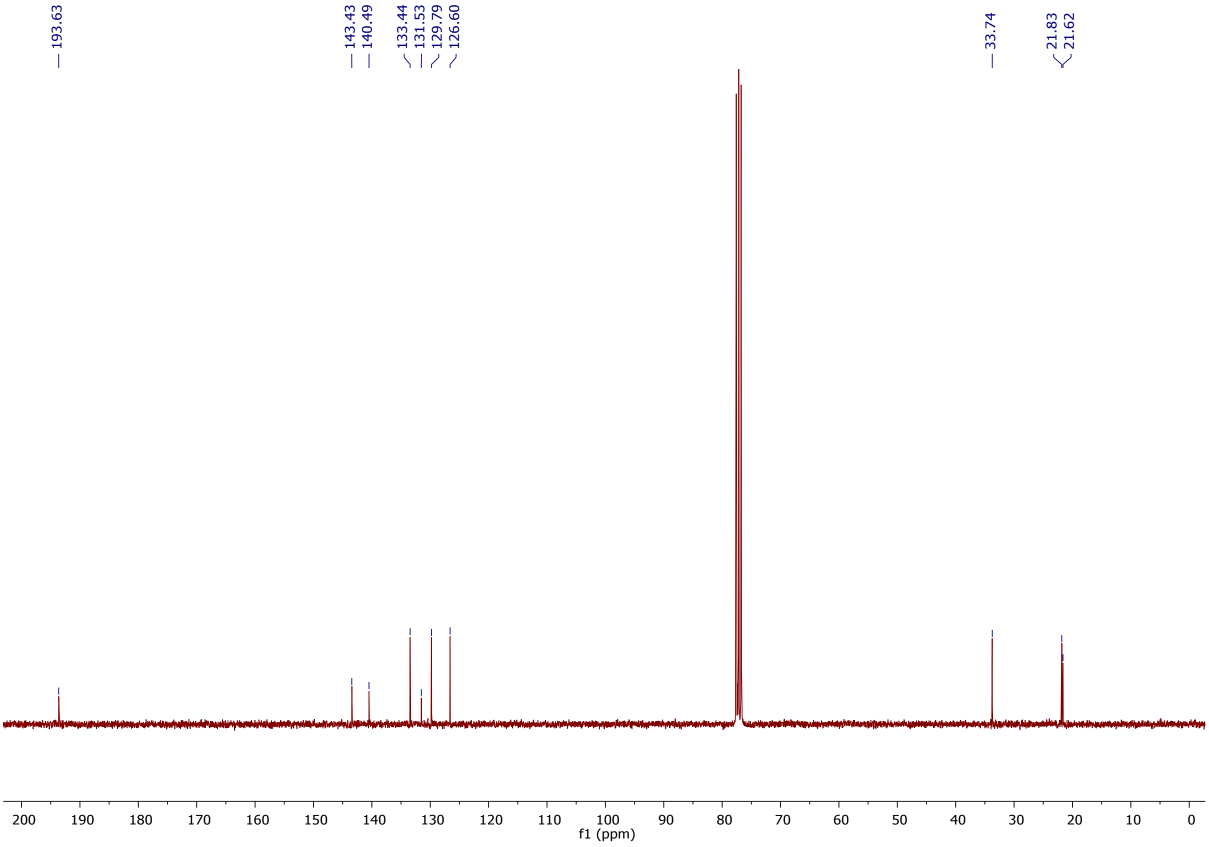
193.63
143.43
140.49
133.44
131.53
129.79
126.60
33.74
21.83
21.62
200 190 180 170 160 150 140 130 120 110 100 90 80 70 60 50 40 30 20 10 0
f1 (ppm)

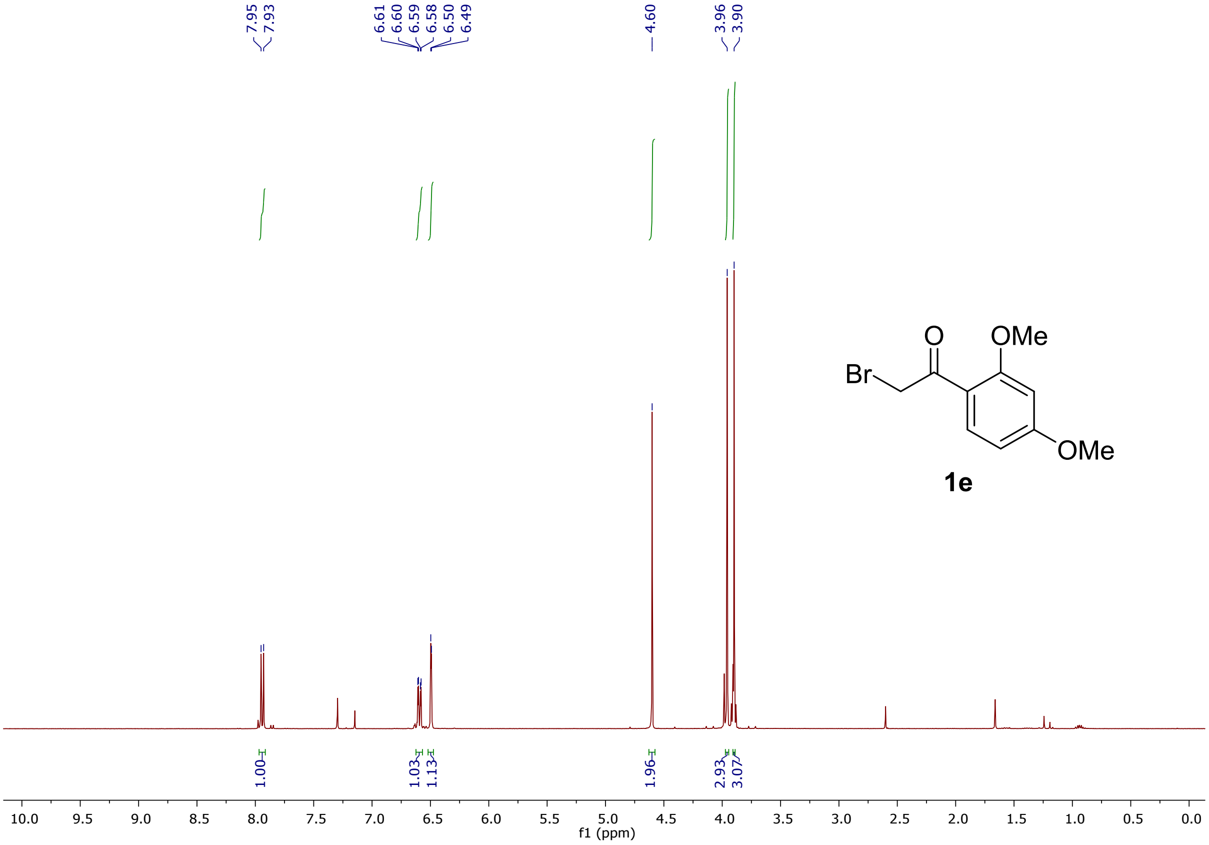
7.95
7.93
6.61
6.60
6.59
6.58
6.50
6.49
4.60
3.96
3.90
Br
O
OMe
OMe
1e
1.00
1.03
1.13
1.96
2.93
3.07
f1 (ppm)

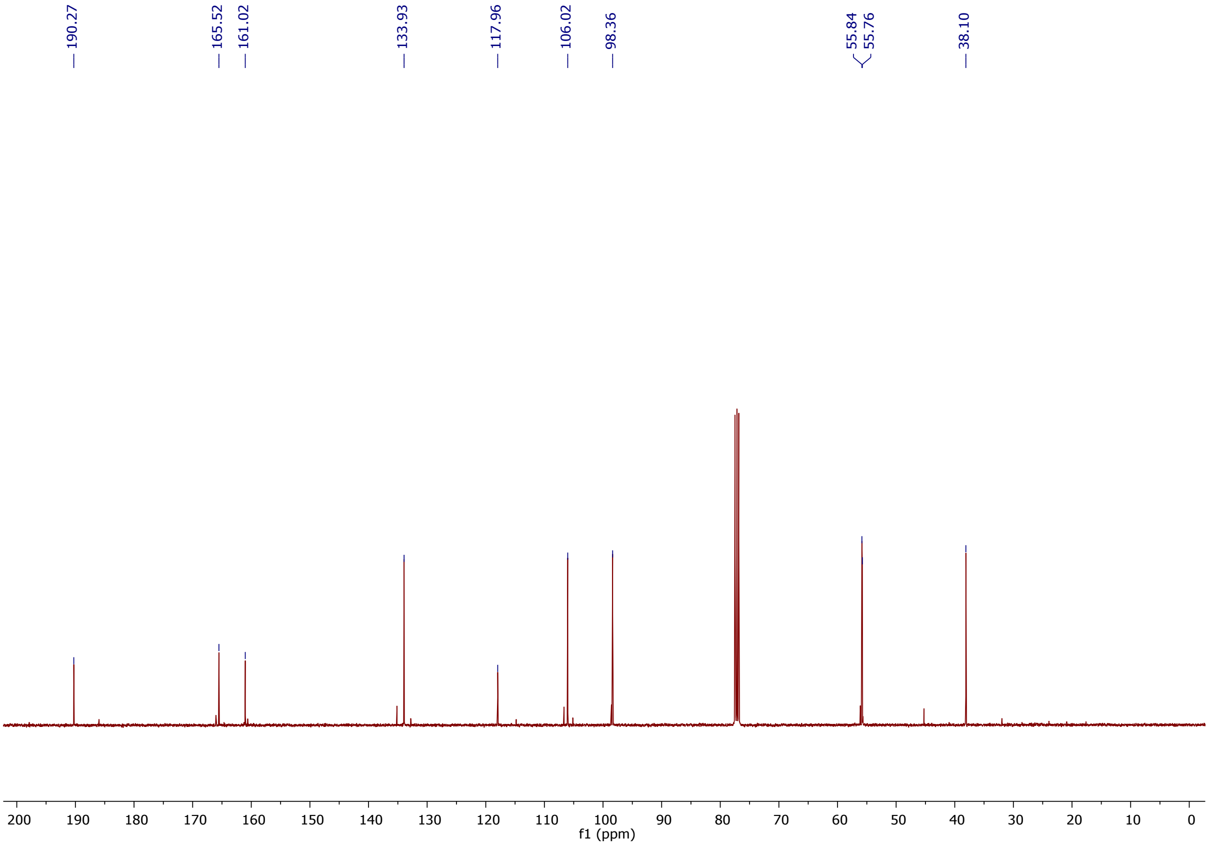
190.27
165.52
161.02
133.93
117.96
106.02
98.36
55.84
55.76
38.10
f1 (ppm)

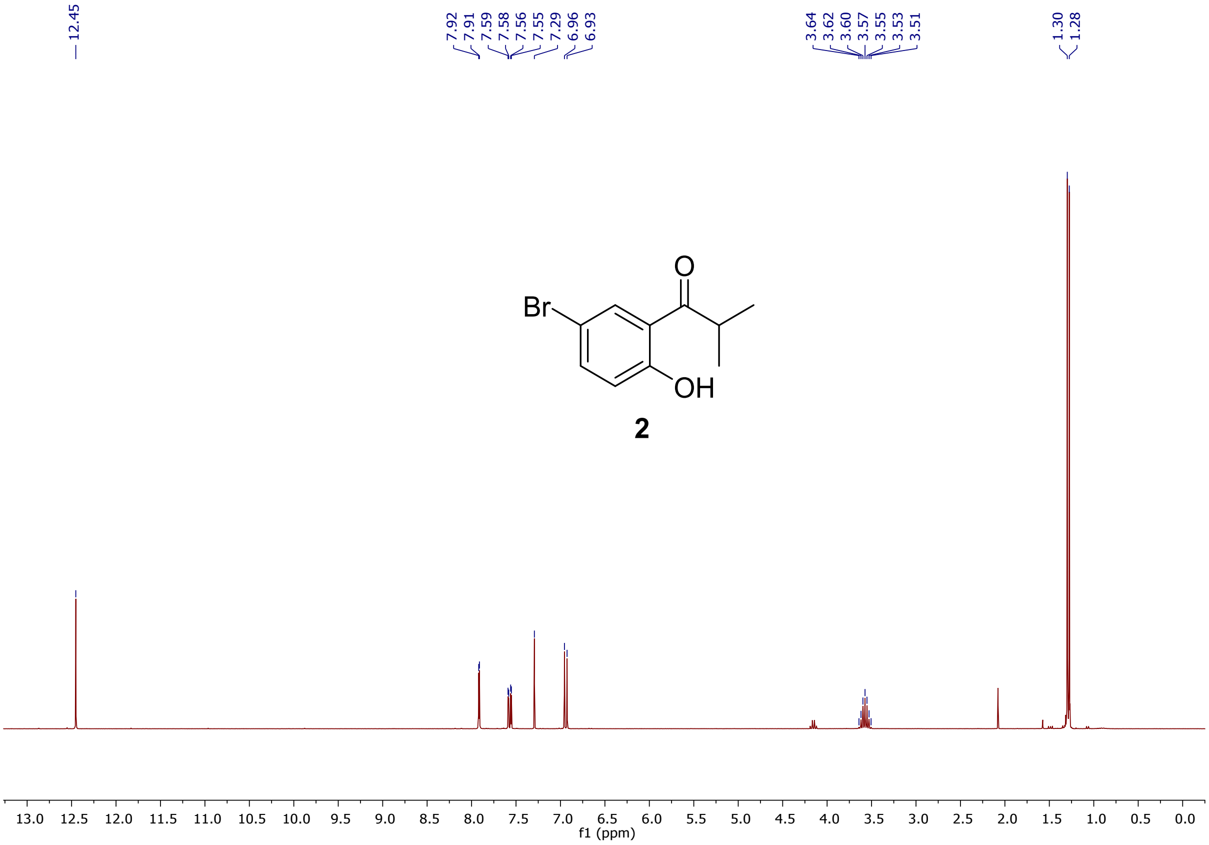
12.45
7.92
7.91
7.59
7.58
7.56
7.55
7.29
6.96
6.93
3.64
3.62
3.60
3.57
3.55
3.53
3.51
1.30
1.28
Br
O
OH
2
13.0 12.5 12.0 11.5 11.0 10.5 10.0 9.5 9.0 8.5 8.0 7.5 7.0 6.5 6.0 5.5 5.0 4.5 4.0 3.5 3.0 2.5 2.0 1.5 1.0 0.5 0.0
f1 (ppm)

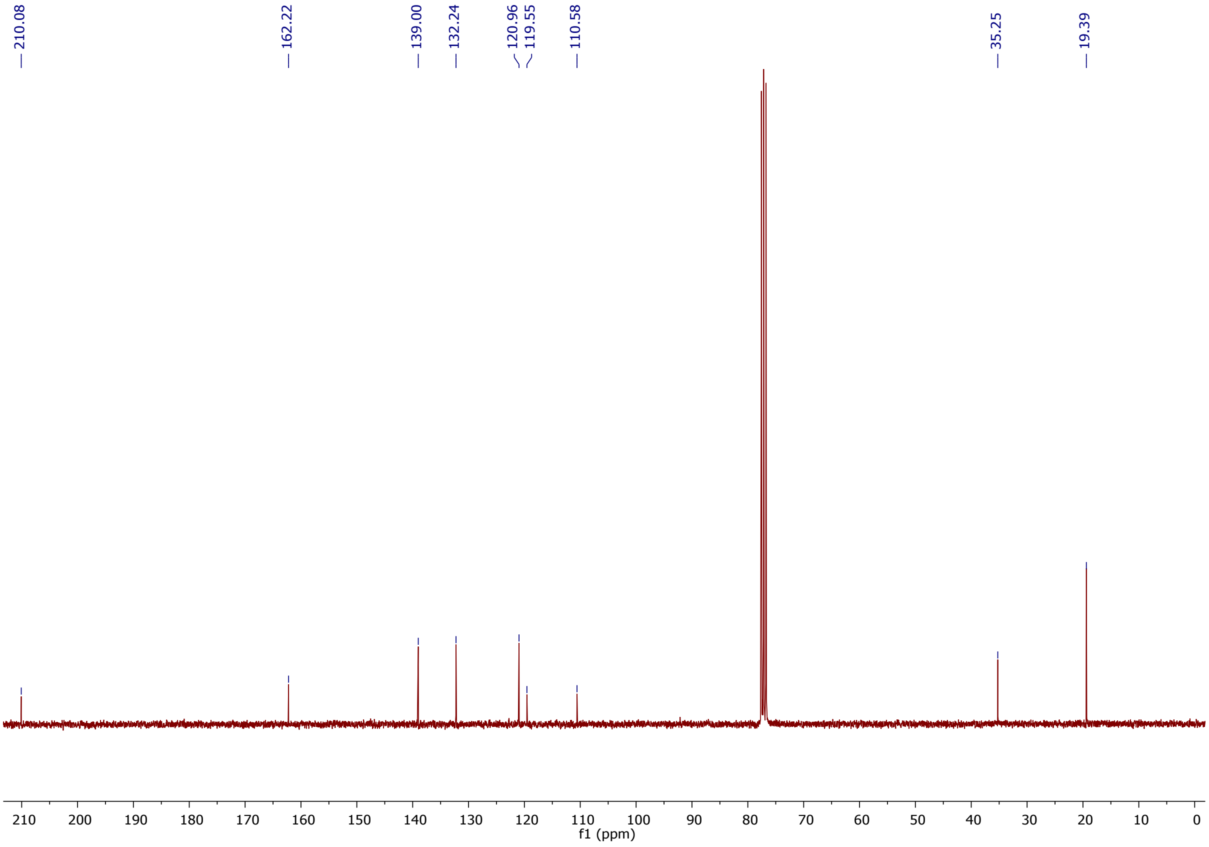
210.08
162.22
139.00
132.24
120.96
119.55
110.58
35.25
19.39
210 200 190 180 170 160 150 140 130 120 110 100 90 80 70 60 50 40 30 20 10 0
f1 (ppm)

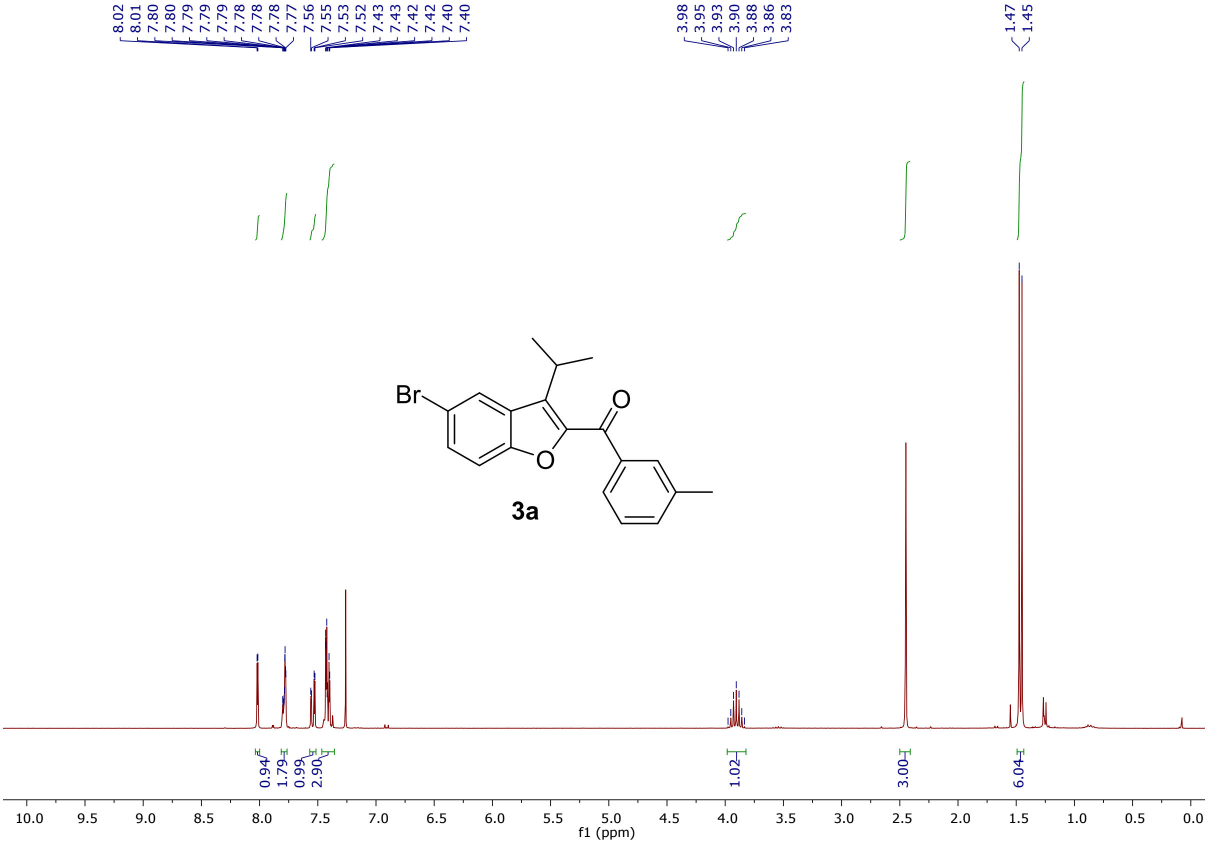
8.02
8.01
7.80
7.80
7.79
7.79
7.79
7.78
7.78
7.77
7.56
7.55
7.53
7.52
7.43
7.43
7.42
7.40
7.40
3.98
3.95
3.93
3.90
3.88
3.86
3.83
1.47
1.45
Br
O
O
3a
0.94
1.79
0.99
2.90
1.02
3.00
6.04
10.0 9.5 9.0 8.5 8.0 7.5 7.0 6.5 6.0 5.5 5.0 4.5 4.0 3.5 3.0 2.5 2.0 1.5 1.0 0.5 0.0
f1 (ppm)

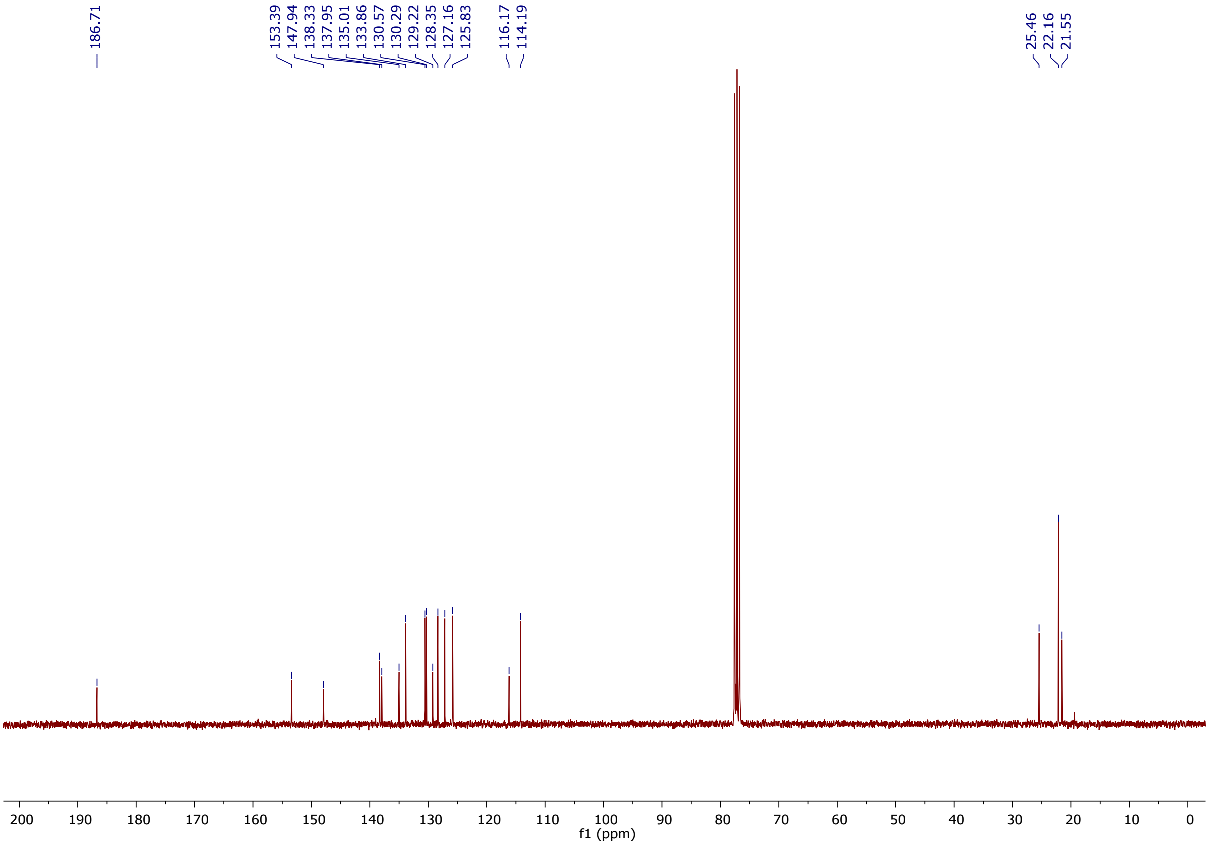
186.71
153.39
147.94
138.33
137.95
135.01
133.86
130.57
130.29
129.22
128.35
127.16
125.83
116.17
114.19
25.46
22.16
21.55
200 190 180 170 160 150 140 130 120 110 100 90 80 70 60 50 40 30 20 10 0
f1 (ppm)

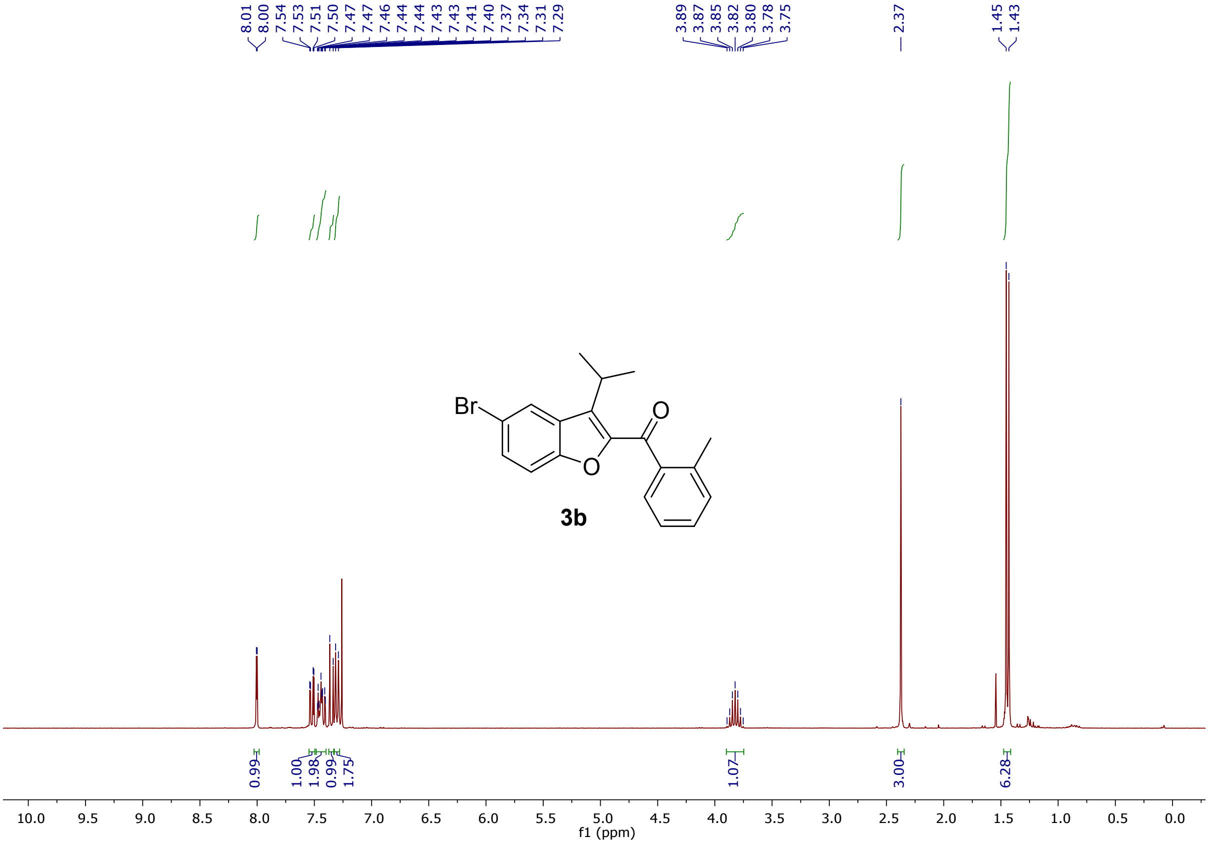
8.01
8.00
7.54
7.53
7.51
7.50
7.47
7.47
7.46
7.44
7.44
7.43
7.43
7.41
7.40
7.37
7.34
7.31
7.29
3.89
3.87
3.85
3.82
3.80
3.78
3.75
2.37
1.45
1.43
Br
O
O
3b
0.99
1.00
1.98
0.99
1.75
1.07
3.00
6.28
10.0 9.5 9.0 8.5 8.0 7.5 7.0 6.5 6.0 5.5 5.0 4.5 4.0 3.5 3.0 2.5 2.0 1.5 1.0 0.5 0.0
f1 (ppm)

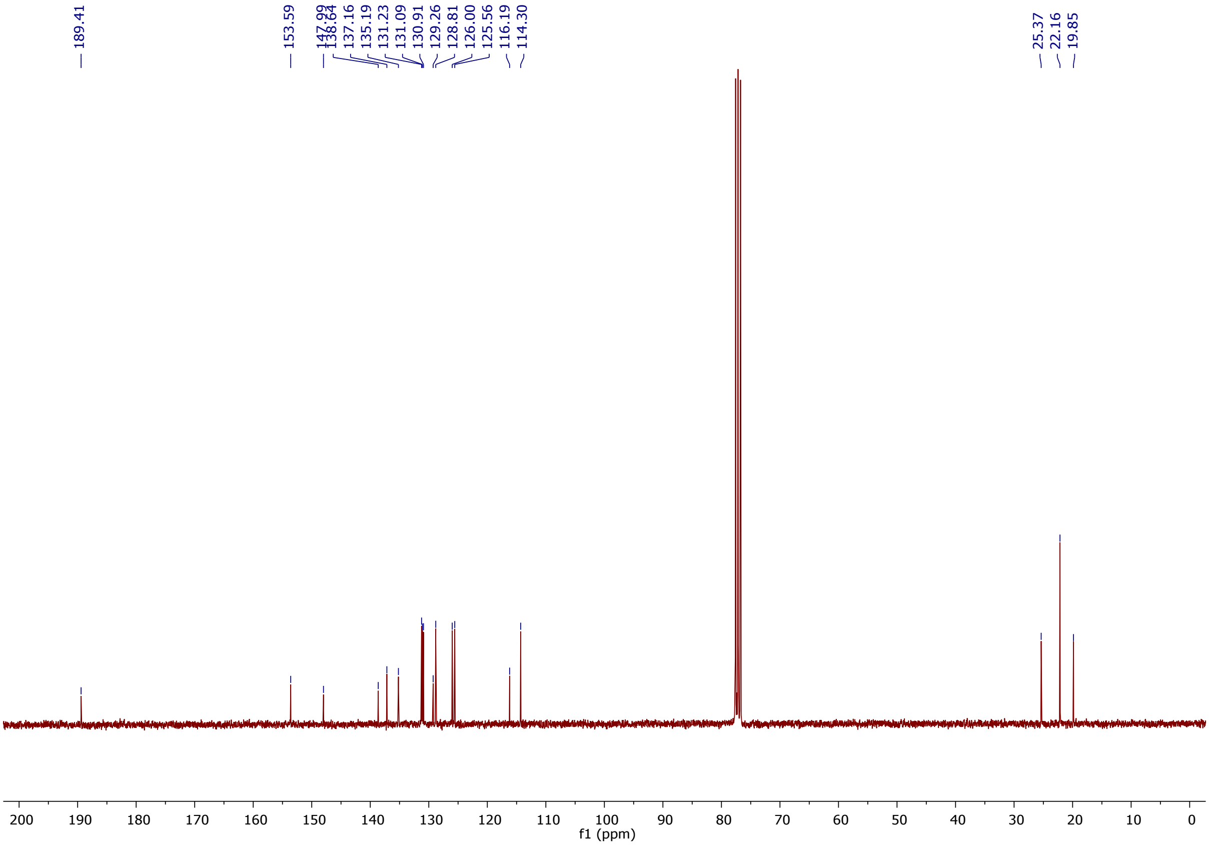
189.41
153.59
147.99
138.64
137.16
135.19
131.23
131.09
130.91
129.26
128.81
126.00
125.56
116.19
114.30
25.37
22.16
19.85
200 190 180 170 160 150 140 130 120 110 100 90 80 70 60 50 40 30 20 10 0
f1 (ppm)

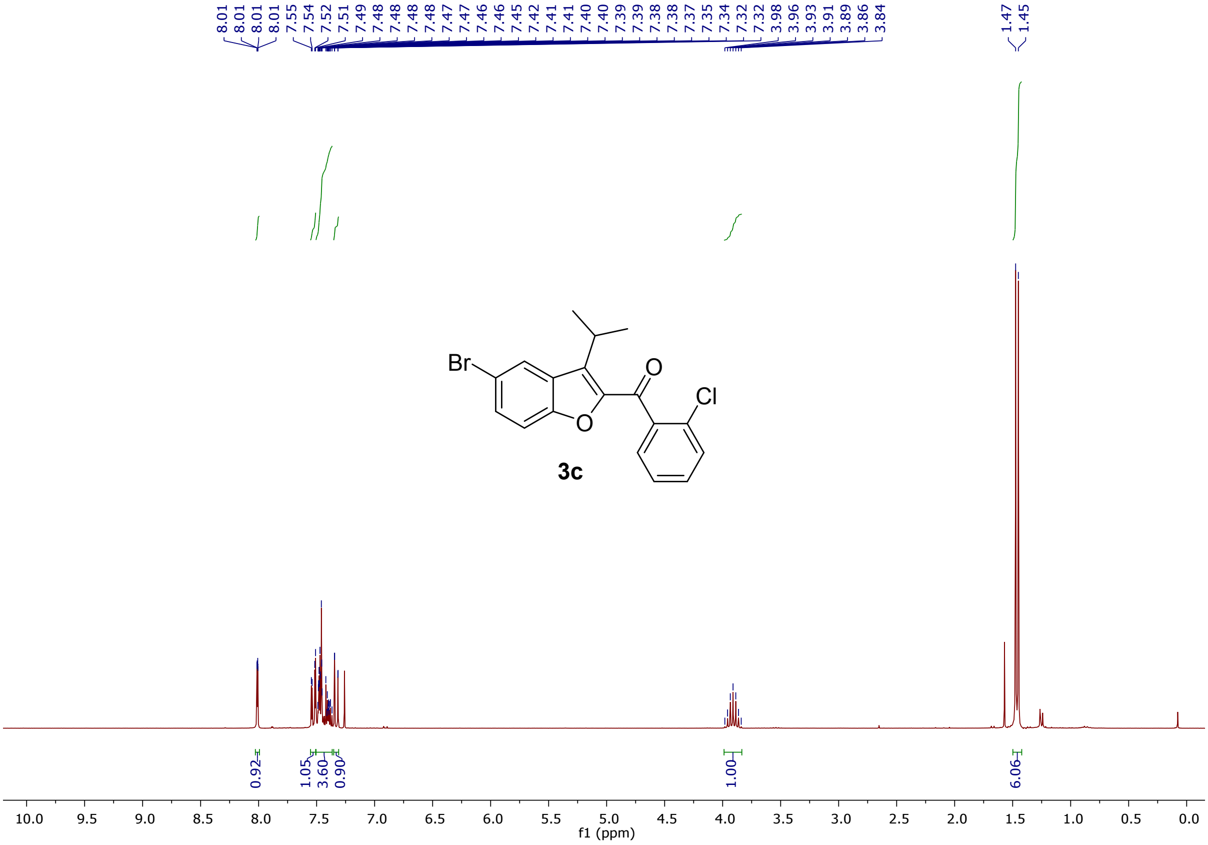
8.01 8.01 8.01 8.01 7.55 7.54 7.52 7.51 7.49 7.48 7.48 7.48 7.47 7.47 7.46 7.46 7.45 7.42 7.41 7.41 7.40 7.40 7.39 7.39 7.38 7.38 7.37 7.35 7.34 7.32 7.32 3.98 3.96 3.93 3.91 3.89 3.86 3.84
1.47 1.45
Br
O
O
Cl
3c
0.92
1.05
3.60
0.90
1.00
6.06
f1 (ppm)

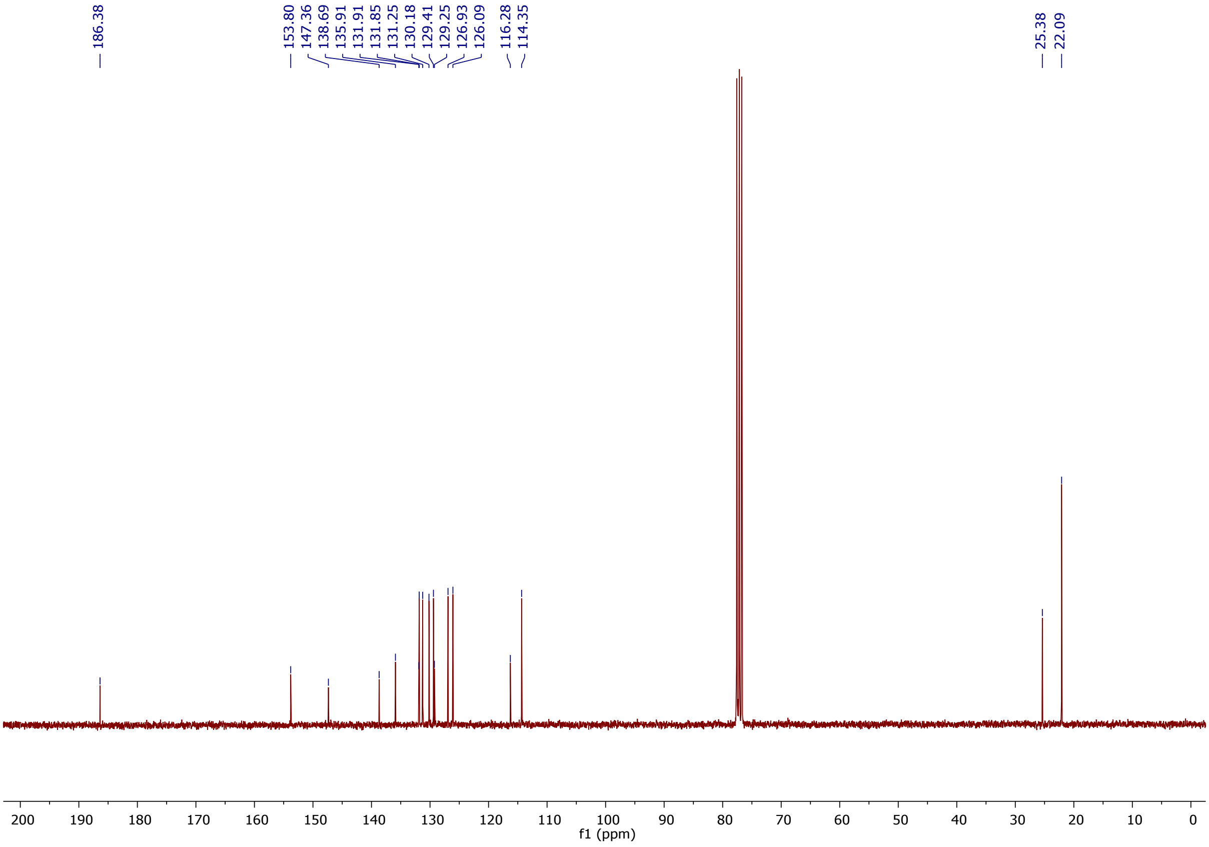
186.38
153.80 147.36 138.69 135.91 131.91 131.85 131.25 130.18 129.41 129.25 126.93 126.09 116.28 114.35
25.38 22.09
f1 (ppm)

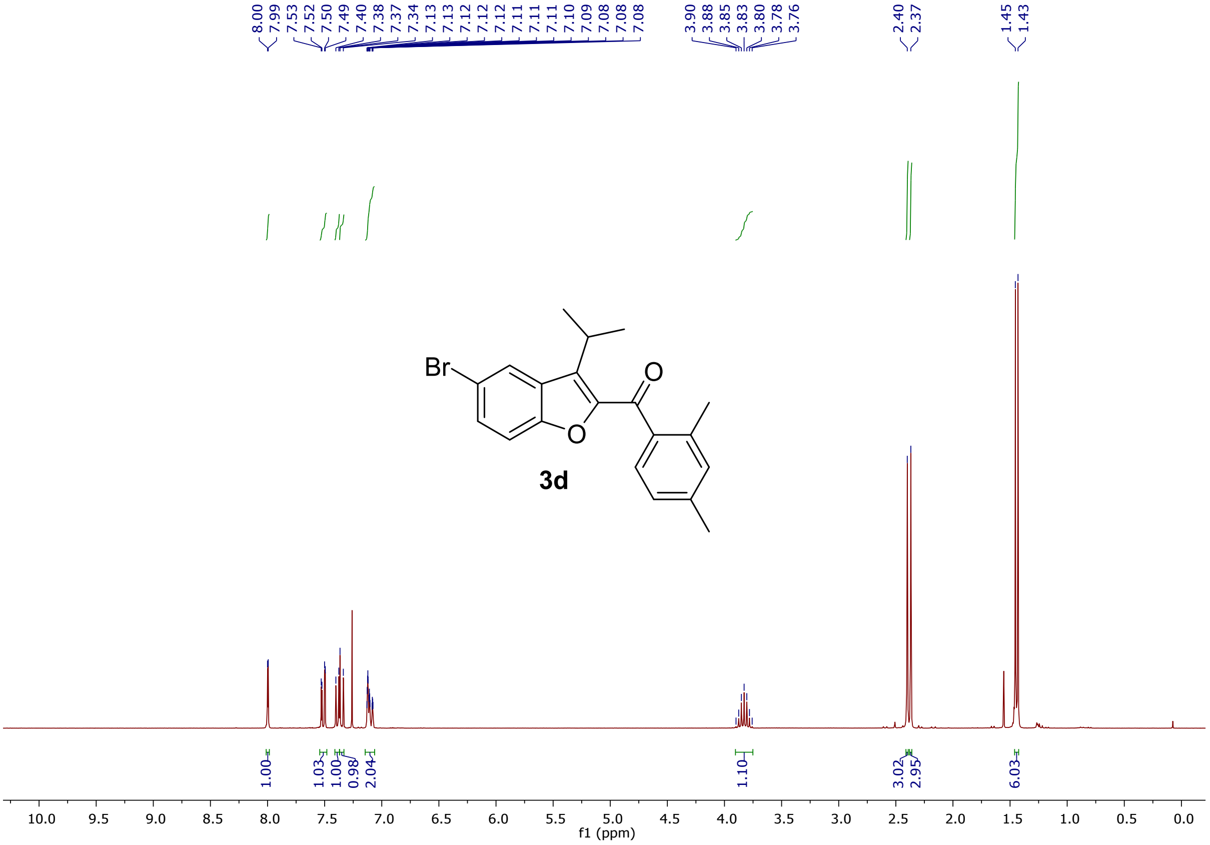
8.00
7.99
7.53
7.52
7.50
7.49
7.40
7.38
7.37
7.34
7.13
7.13
7.12
7.12
7.12
7.11
7.11
7.11
7.10
7.09
7.08
7.08
7.08
3.90
3.88
3.85
3.83
3.80
3.78
3.76
2.40
2.37
1.45
1.43
Br
O
O
3d
1.00
1.03
1.00
0.98
2.04
1.10
3.02
2.95
6.03
f1 (ppm)

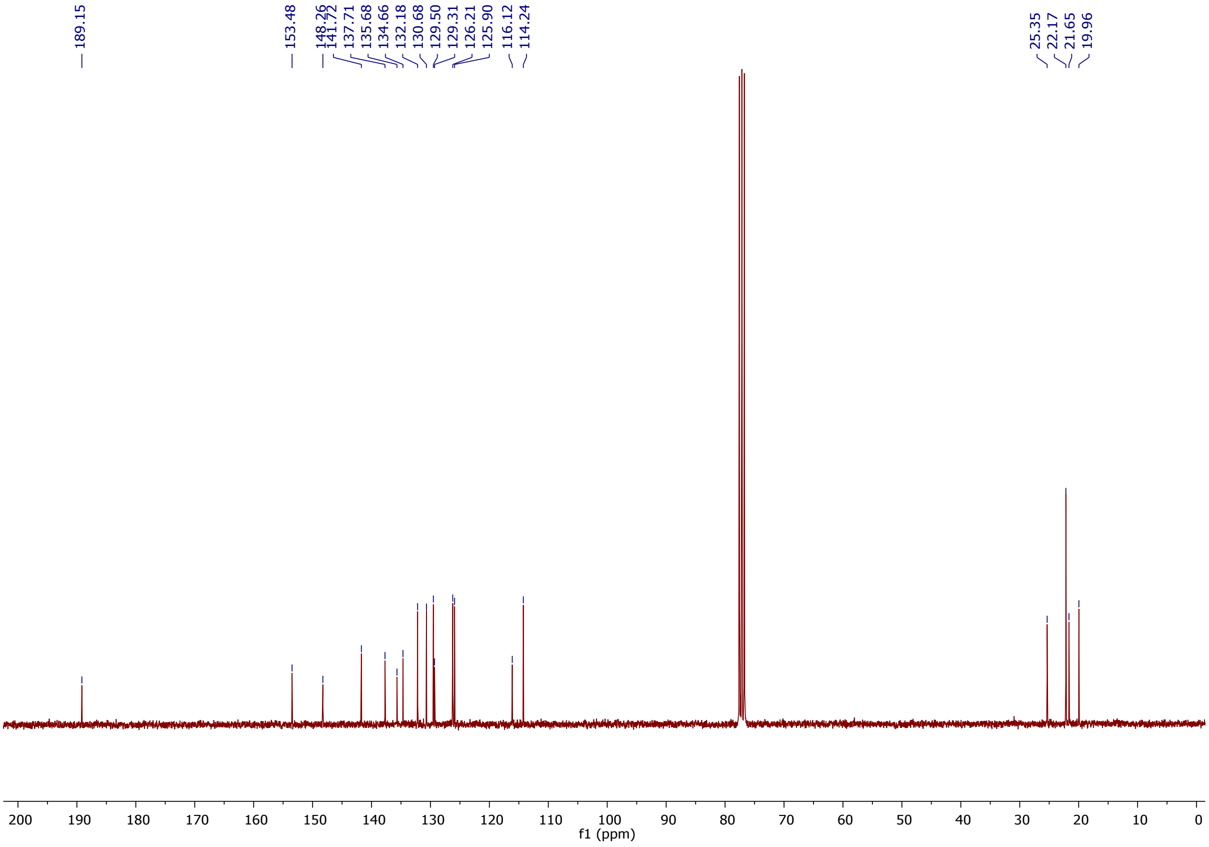
189.15
153.48
148.26
141.72
137.71
135.68
134.66
132.18
130.68
129.50
129.31
126.21
125.90
116.12
114.24
25.35
22.17
21.65
19.96
f1 (ppm)

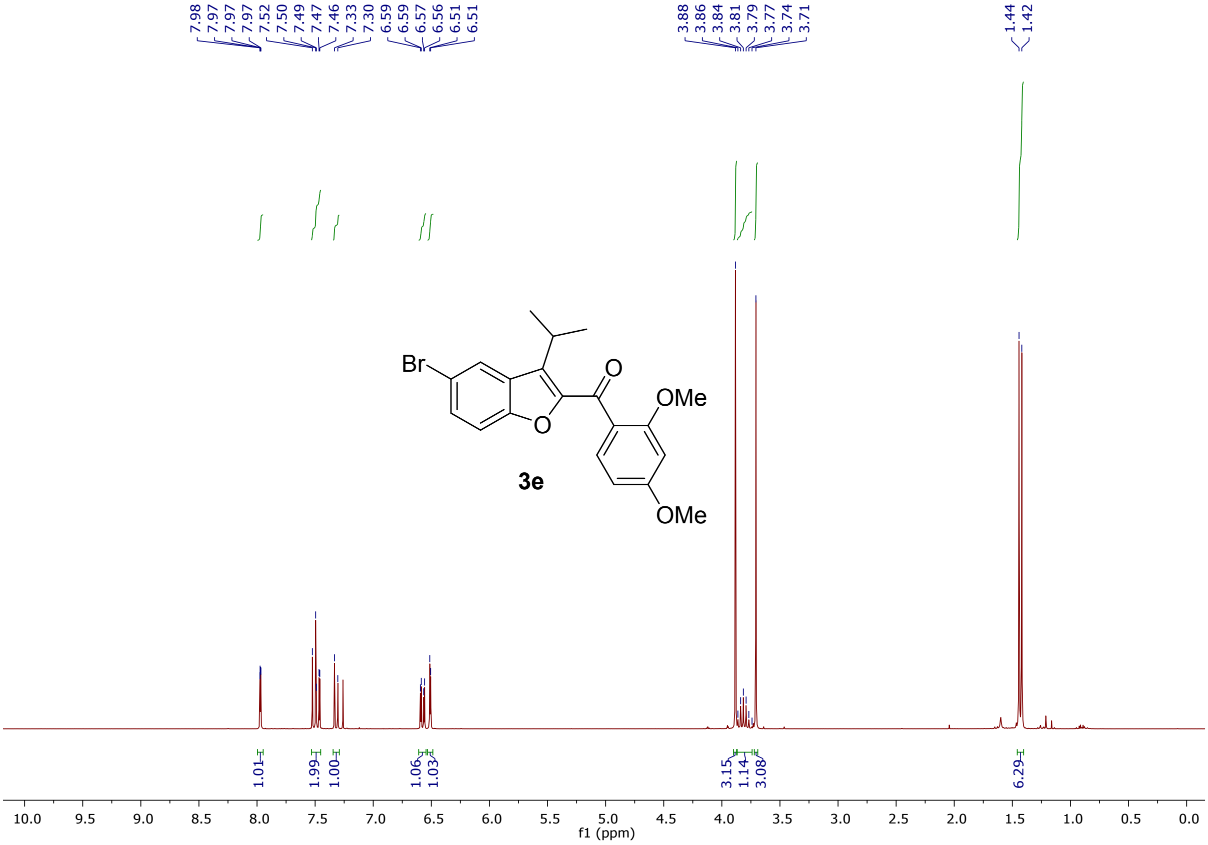
7.98 7.97 7.97 7.97 7.52 7.50 7.49 7.47 7.46 7.33 7.30 6.59 6.59 6.57 6.56 6.51 6.51
3.88 3.86 3.84 3.81 3.79 3.77 3.74 3.71
1.44 1.42
Br
O
OMe
OMe
O
3e
1.01
1.99
1.00
1.06
1.03
3.15
1.14
3.08
6.29
10.0 9.5 9.0 8.5 8.0 7.5 7.0 6.5 6.0 5.5 5.0 4.5 4.0 3.5 3.0 2.5 2.0 1.5 1.0 0.5 0.0
f1 (ppm)

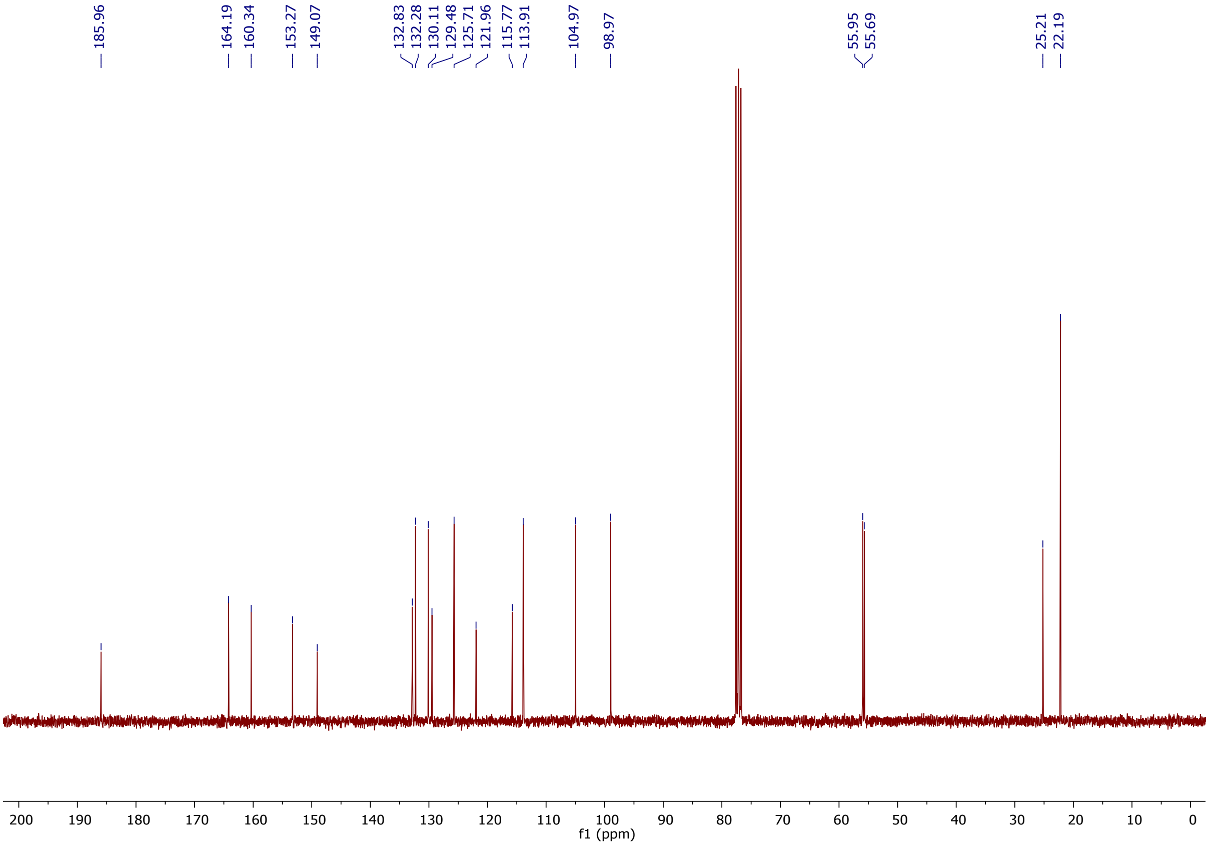
185.96
164.19 160.34
153.27 149.07
132.83 132.28 130.11 129.48 125.71 121.96 115.77 113.91
104.97 98.97
55.95 55.69
25.21 22.19
200 190 180 170 160 150 140 130 120 110 100 90 80 70 60 50 40 30 20 10 0
f1 (ppm)

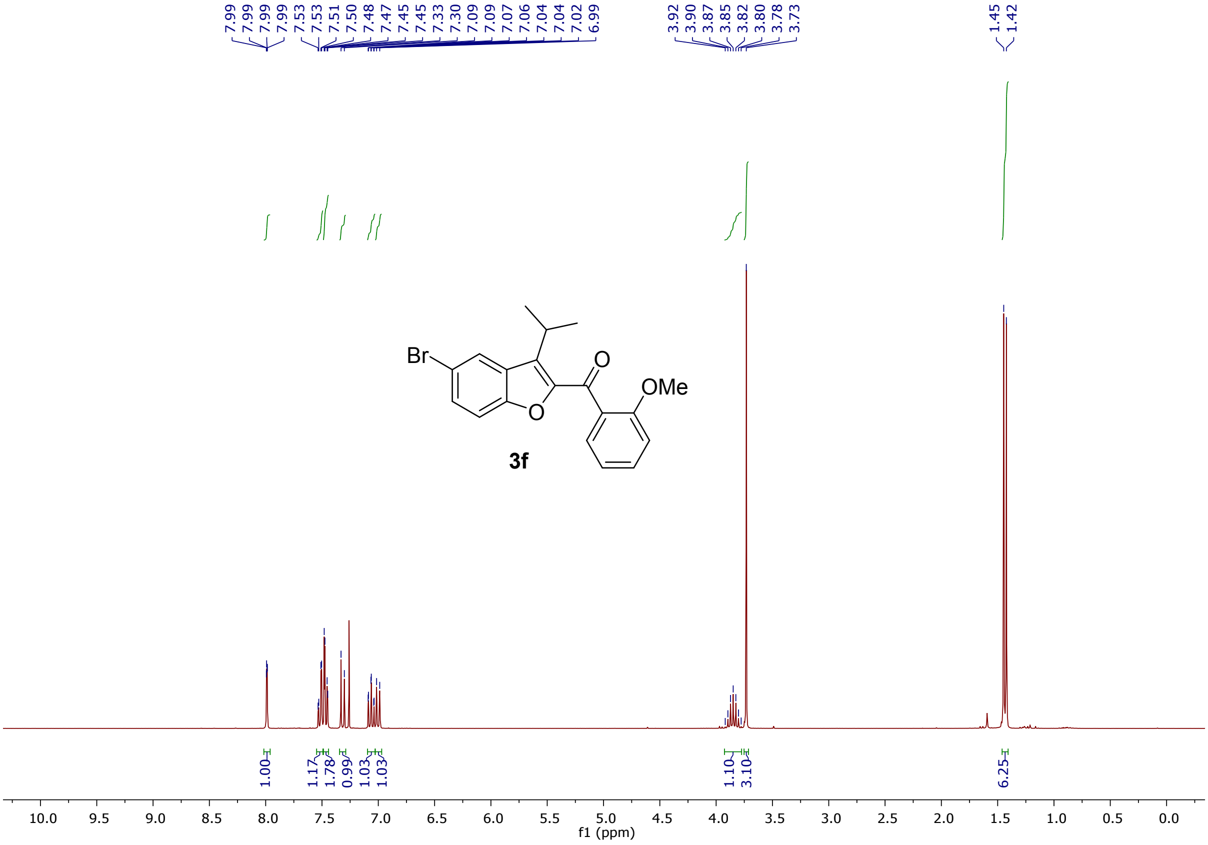
7.99 7.99 7.99 7.99 7.53 7.53 7.51 7.50 7.48 7.47 7.45 7.45 7.33 7.30 7.09 7.09 7.07 7.06 7.04 7.04 7.02 6.99
3.92 3.90 3.87 3.85 3.82 3.80 3.78 3.73
1.45 1.42
Br
O
OMe
O
3f
1.00
1.17
1.78
0.99
1.03
1.03
1.10
3.10
6.25
10.0 9.5 9.0 8.5 8.0 7.5 7.0 6.5 6.0 5.5 5.0 4.5 4.0 3.5 3.0 2.5 2.0 1.5 1.0 0.5 0.0
f1 (ppm)

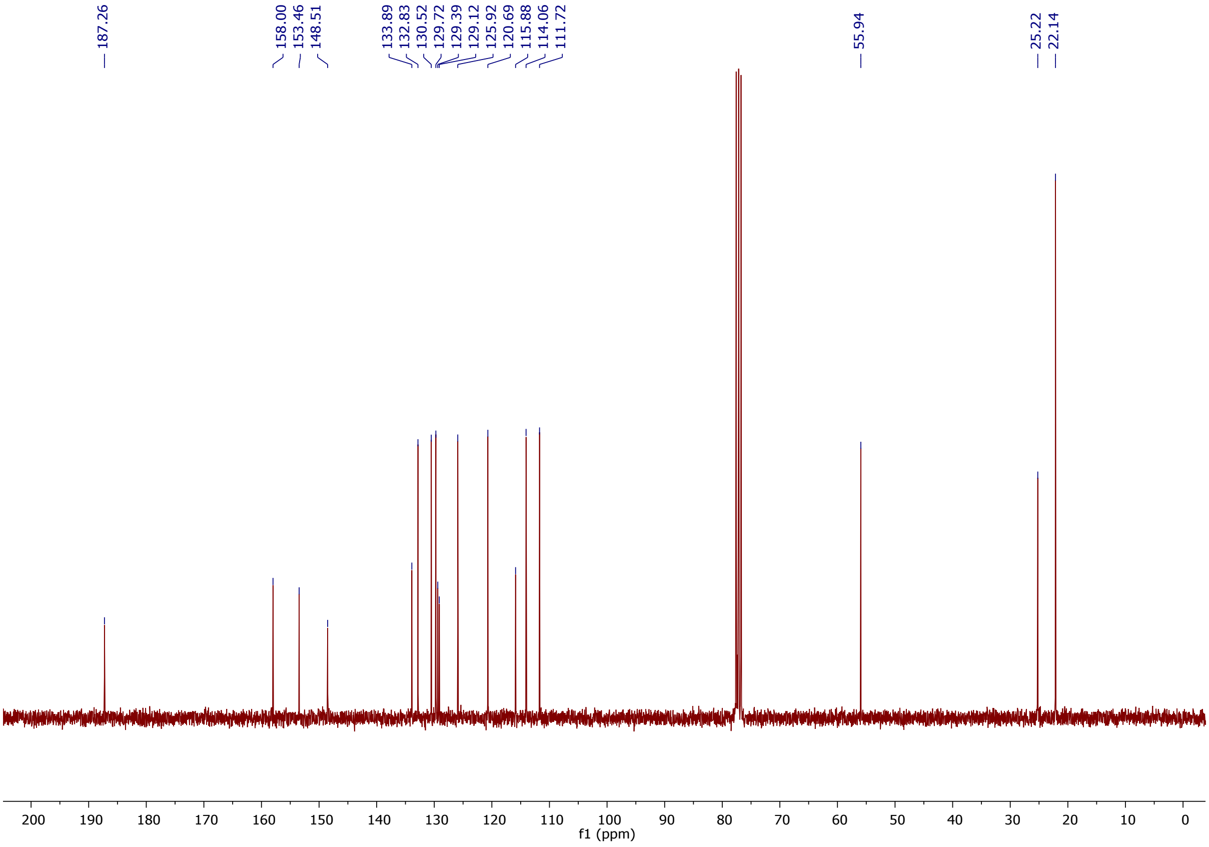
187.26
158.00 153.46 148.51
133.89 132.83 130.52 129.72 129.39 129.12 125.92 120.69 115.88 114.06 111.72
55.94
25.22 22.14
200 190 180 170 160 150 140 130 120 110 100 90 80 70 60 50 40 30 20 10 0
f1 (ppm)

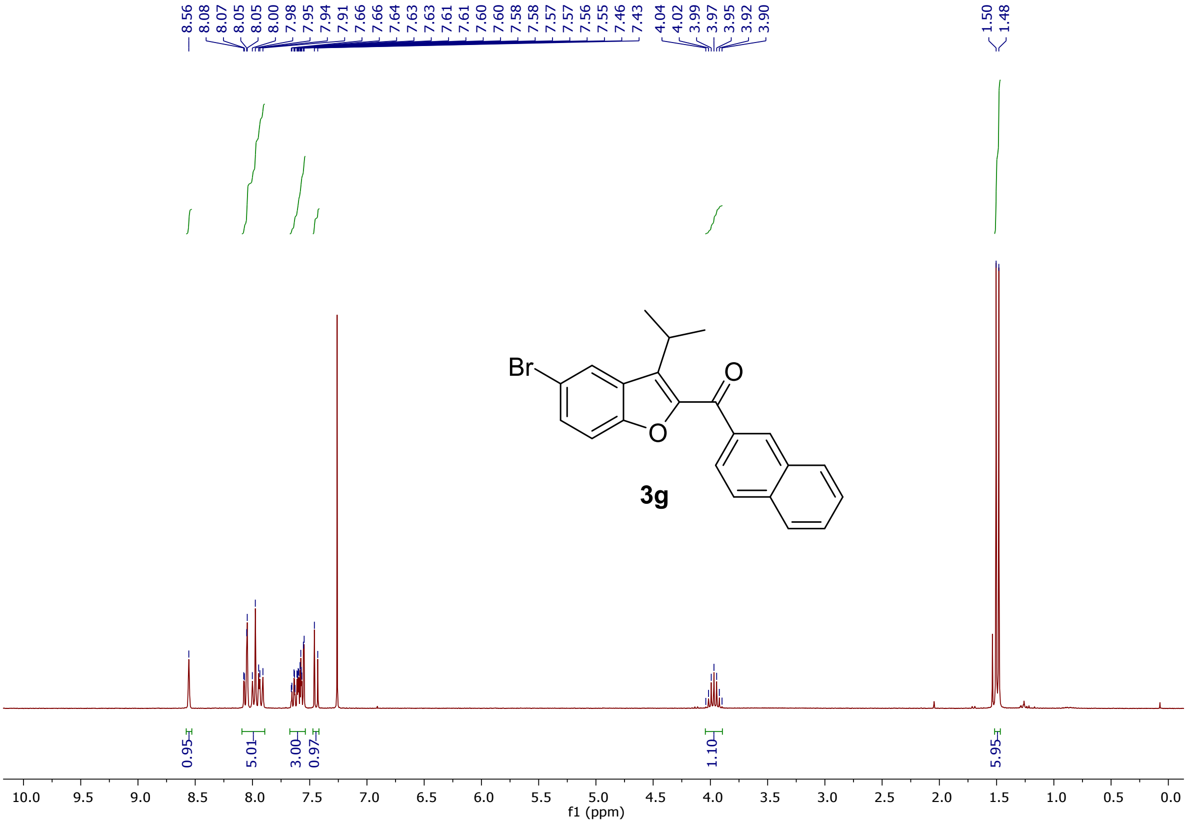
8.56
8.08
8.07
8.05
8.05
8.00
7.98
7.95
7.94
7.91
7.66
7.66
7.64
7.63
7.63
7.61
7.61
7.60
7.58
7.58
7.57
7.57
7.56
7.55
7.46
7.43
4.04
4.02
3.99
3.97
3.95
3.92
3.90
1.50
1.48
Br
O
O
3g
0.95
5.01
3.00
0.97
1.10
5.95
10.0 9.5 9.0 8.5 8.0 7.5 7.0 6.5 6.0 5.5 5.0 4.5 4.0 3.5 3.0 2.5 2.0 1.5 1.0 0.5 0.0
f1 (ppm)

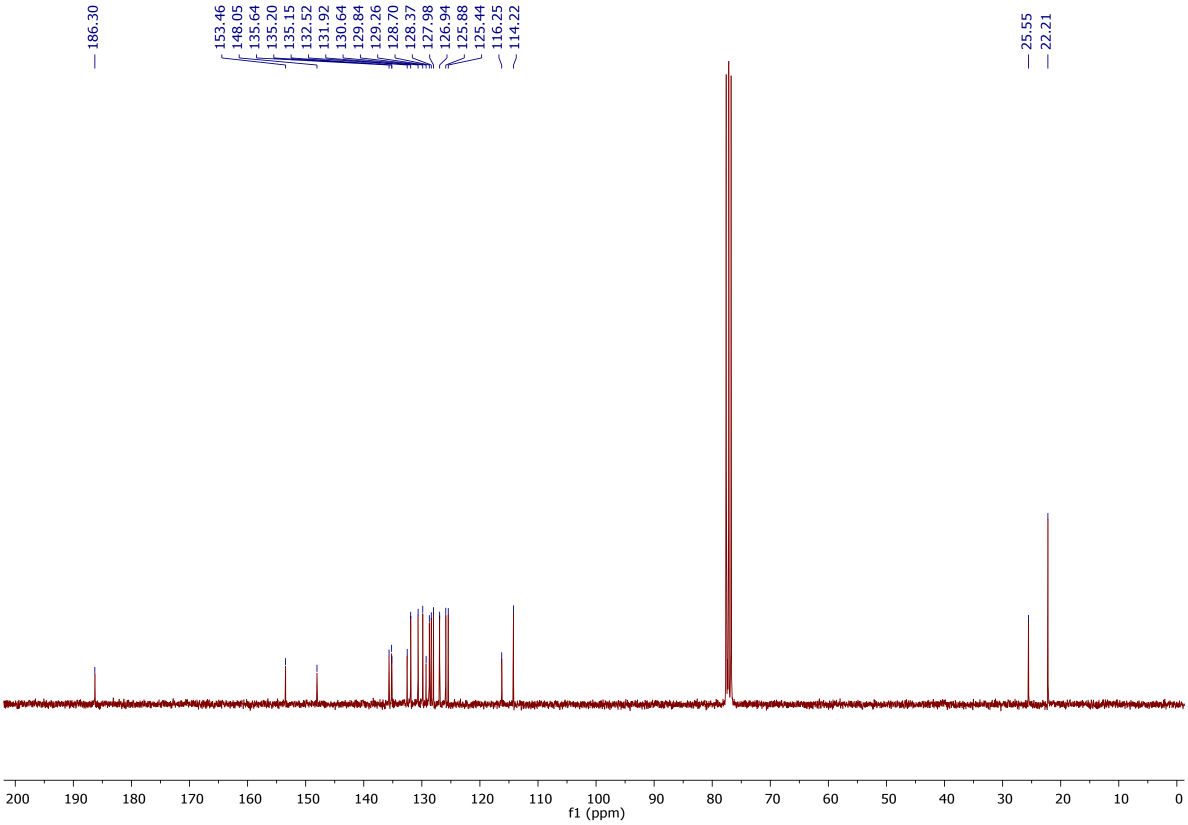
186.30
153.46
148.05
135.64
135.20
135.15
132.52
131.92
130.64
129.84
129.26
128.70
128.37
127.98
126.94
125.88
125.44
116.25
114.22
25.55
22.21
200 190 180 170 160 150 140 130 120 110 100 90 80 70 60 50 40 30 20 10 0
f1 (ppm)

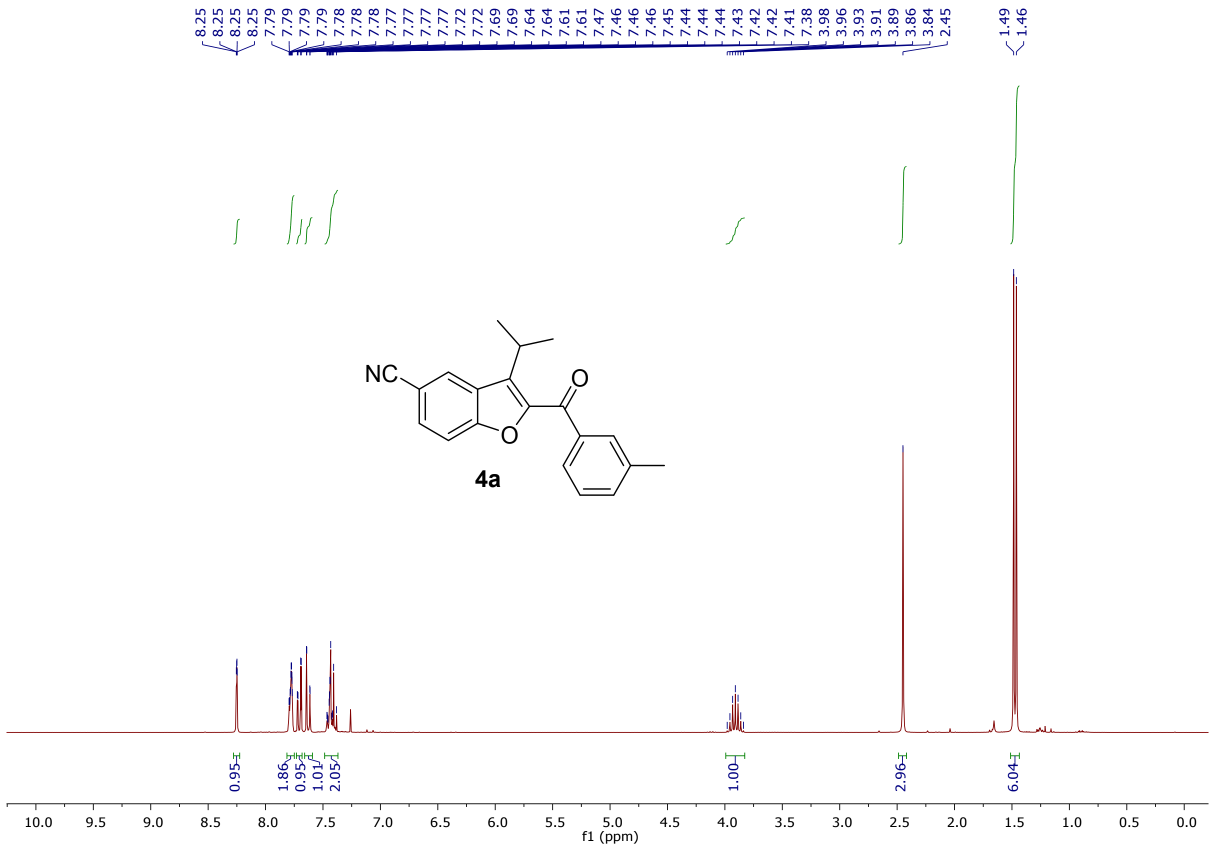
8.25 8.25 8.25 8.25 7.79 7.79 7.79 7.79 7.78 7.78 7.77 7.77 7.77 7.77 7.72 7.72 7.69 7.69 7.64 7.64 7.61 7.61 7.47 7.46 7.46 7.45 7.44 7.44 7.44 7.43 7.42 7.42 7.41 7.38 3.98 3.96 3.93 3.91 3.89 3.86 3.84 2.45
1.49 1.46
NC
O
O
4a
0.95 1.86 0.95 1.01 2.05 1.00 2.96 6.04
f1 (ppm)

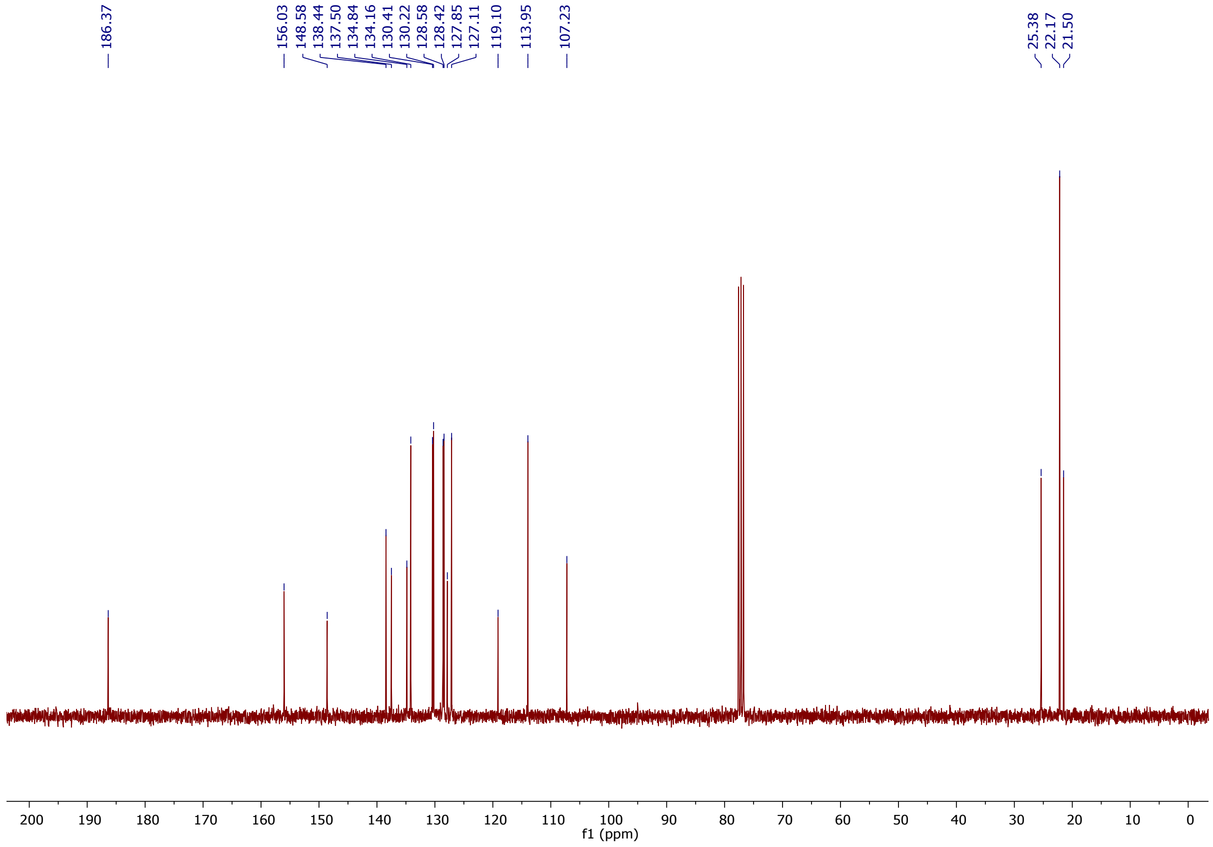
186.37
156.03 148.58 138.44 137.50 134.84 134.16 130.41 130.22 128.58 128.42 127.85 127.11 119.10 113.95 107.23
25.38 22.17 21.50
f1 (ppm)

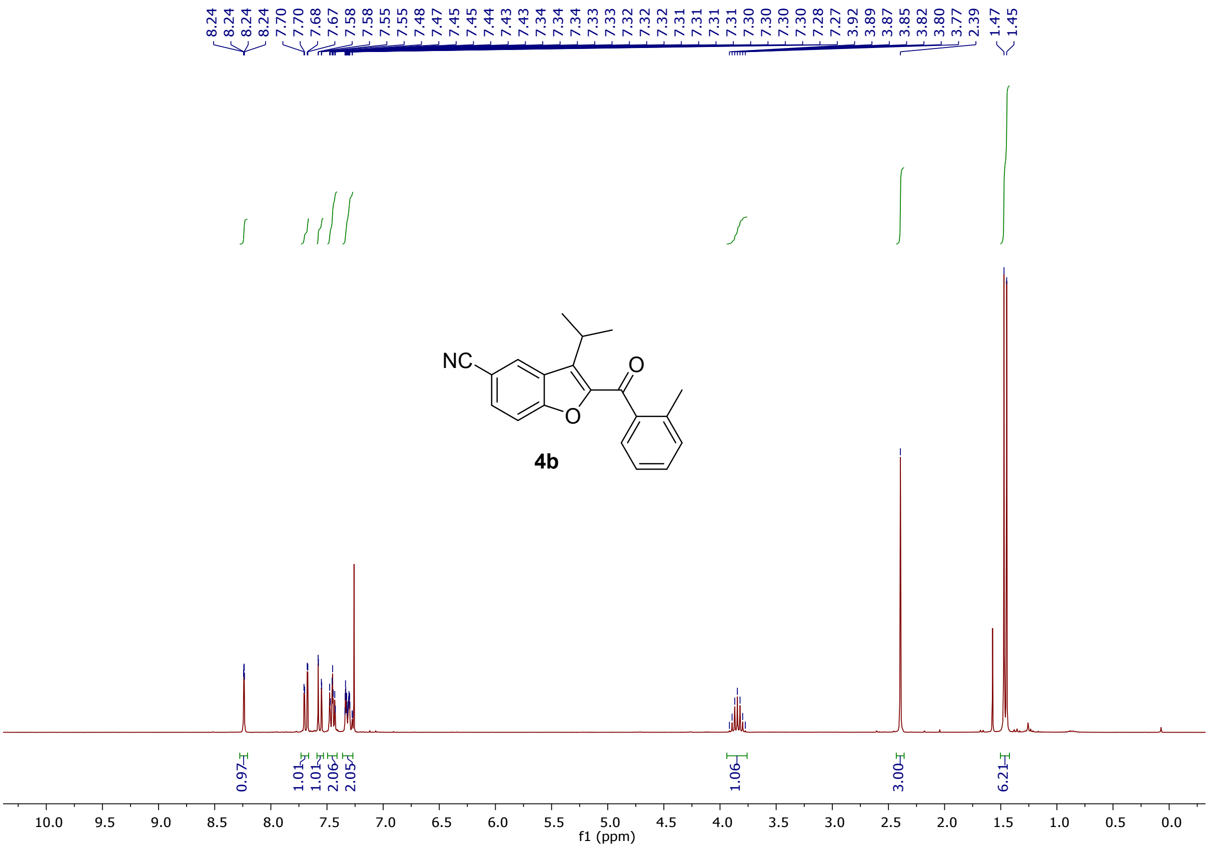
8.24
8.24
8.24
8.24
7.70
7.70
7.68
7.67
7.58
7.58
7.55
7.55
7.48
7.47
7.45
7.45
7.44
7.43
7.43
7.34
7.34
7.33
7.33
7.32
7.32
7.32
7.31
7.31
7.31
7.31
7.30
7.30
7.30
7.30
7.28
7.27
3.92
3.89
3.87
3.85
3.82
3.80
3.77
2.39
1.47
1.45
NC
O
O
4b
0.97
1.01
1.01
2.06
2.05
1.06
3.00
6.21
10.0
9.5
9.0
8.5
8.0
7.5
7.0
6.5
6.0
5.5
5.0
4.5
4.0
3.5
3.0
2.5
2.0
1.5
1.0
0.5
0.0
f1 (ppm)

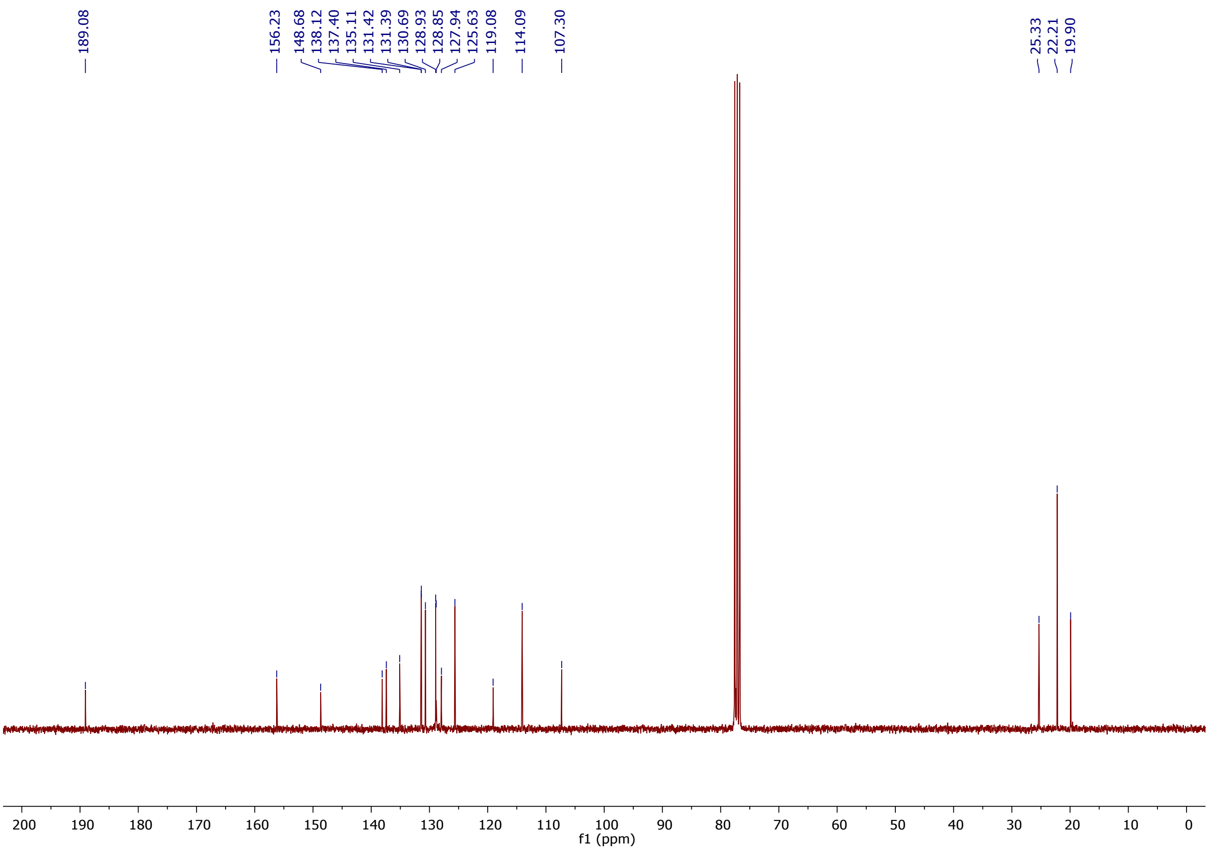
189.08
156.23
148.68
138.12
137.40
135.11
131.42
131.39
130.69
128.93
128.85
127.94
125.63
119.08
114.09
107.30
25.33
22.21
19.90
200
190
180
170
160
150
140
130
120
110
100
90
80
70
60
50
40
30
20
10
0
f1 (ppm)

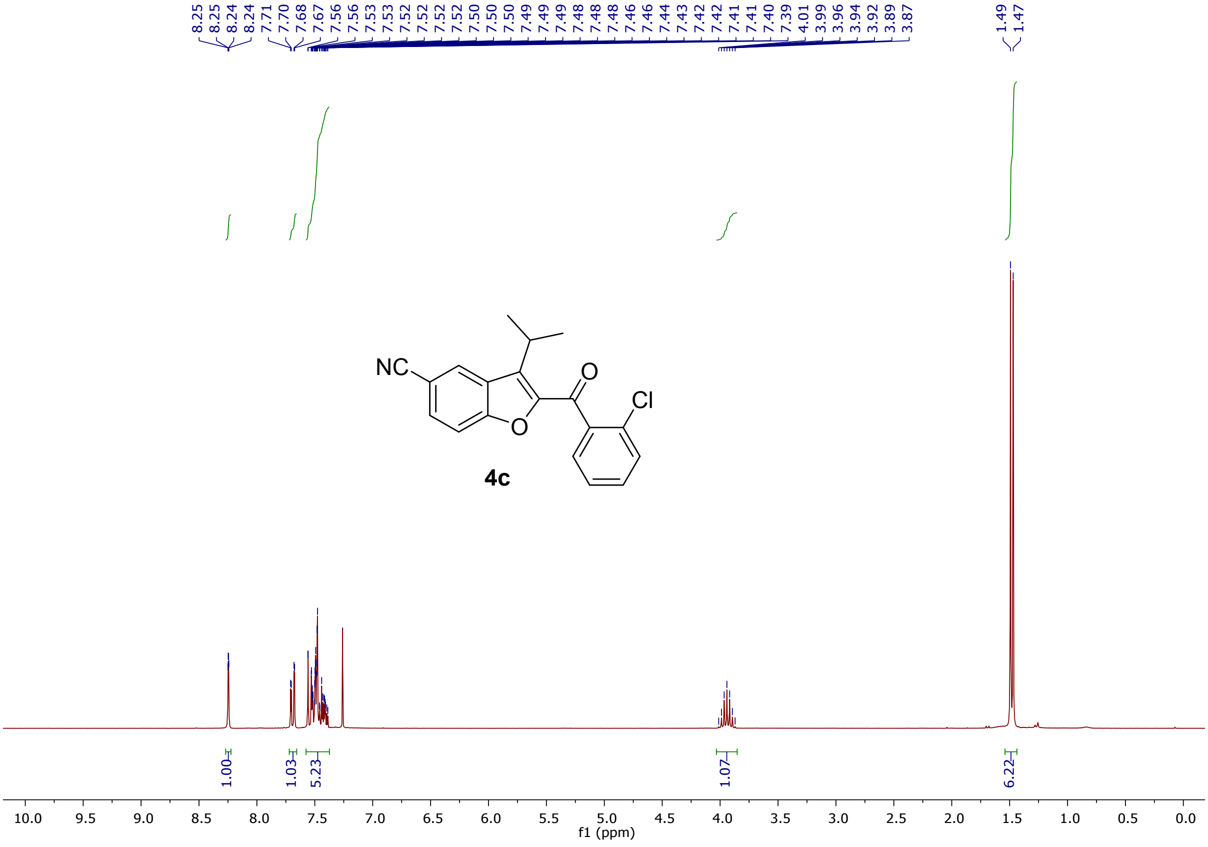
8.25 8.25 8.24 8.24 7.71 7.70 7.68 7.67 7.56 7.56 7.53 7.53 7.52 7.52 7.52 7.50 7.50 7.50 7.49 7.49 7.49 7.48 7.48 7.48 7.46 7.44 7.43 7.42 7.42 7.41 7.41 7.40 7.39 4.01 3.99 3.96 3.94 3.92 3.89 3.87
1.49 1.47
NC
O
Cl
4c
1.00
1.03
5.23
1.07
6.22
10.0 9.5 9.0 8.5 8.0 7.5 7.0 6.5 6.0 5.5 5.0 4.5 4.0 3.5 3.0 2.5 2.0 1.5 1.0 0.5 0.0
f1 (ppm)

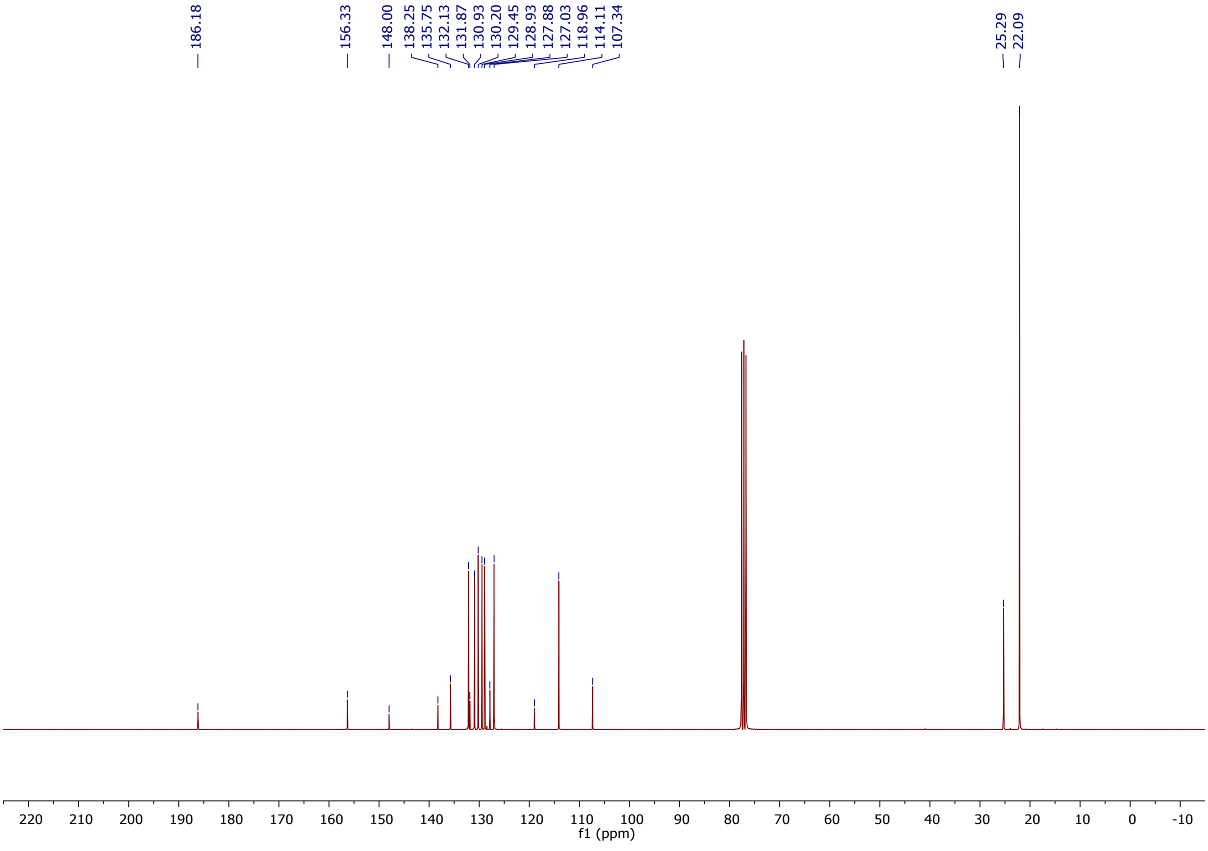
186.18
156.33
148.00
138.25 135.75 132.13 131.87 130.93 130.20 129.45 128.93 127.88 127.03 118.96 114.11 107.34
25.29 22.09
220 210 200 190 180 170 160 150 140 130 120 110 100 90 80 70 60 50 40 30 20 10 0 -10
f1 (ppm)

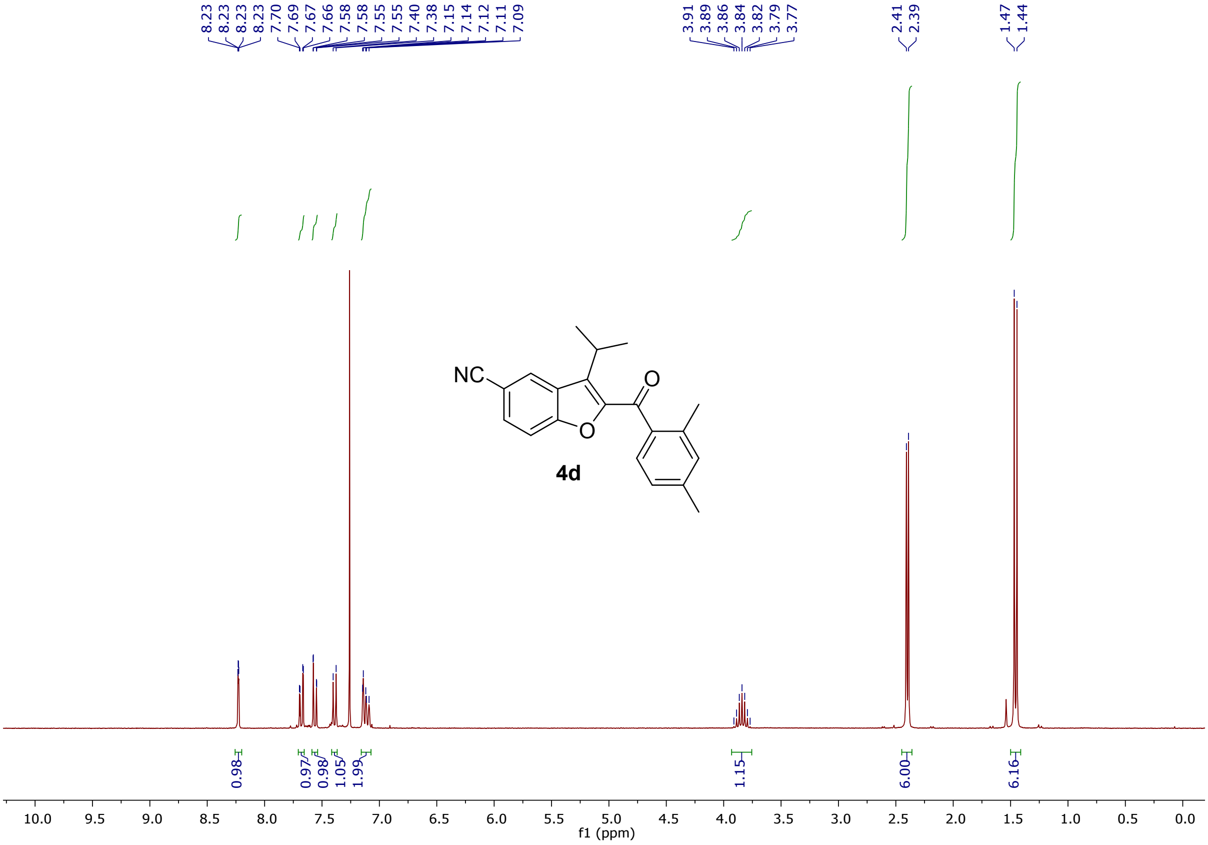
8.23 8.23 8.23 8.23 7.70 7.69 7.67 7.66 7.58 7.58 7.55 7.55 7.40 7.38 7.15 7.14 7.12 7.11 7.09
3.91 3.89 3.86 3.84 3.82 3.79 3.77
2.41 2.39
1.47 1.44
NC
O
O
4d
0.98
0.97
0.98
1.05
1.99
1.15
6.00
6.16
10.0 9.5 9.0 8.5 8.0 7.5 7.0 6.5 6.0 5.5 5.0 4.5 4.0 3.5 3.0 2.5 2.0 1.5 1.0 0.5 0.0
f1 (ppm)

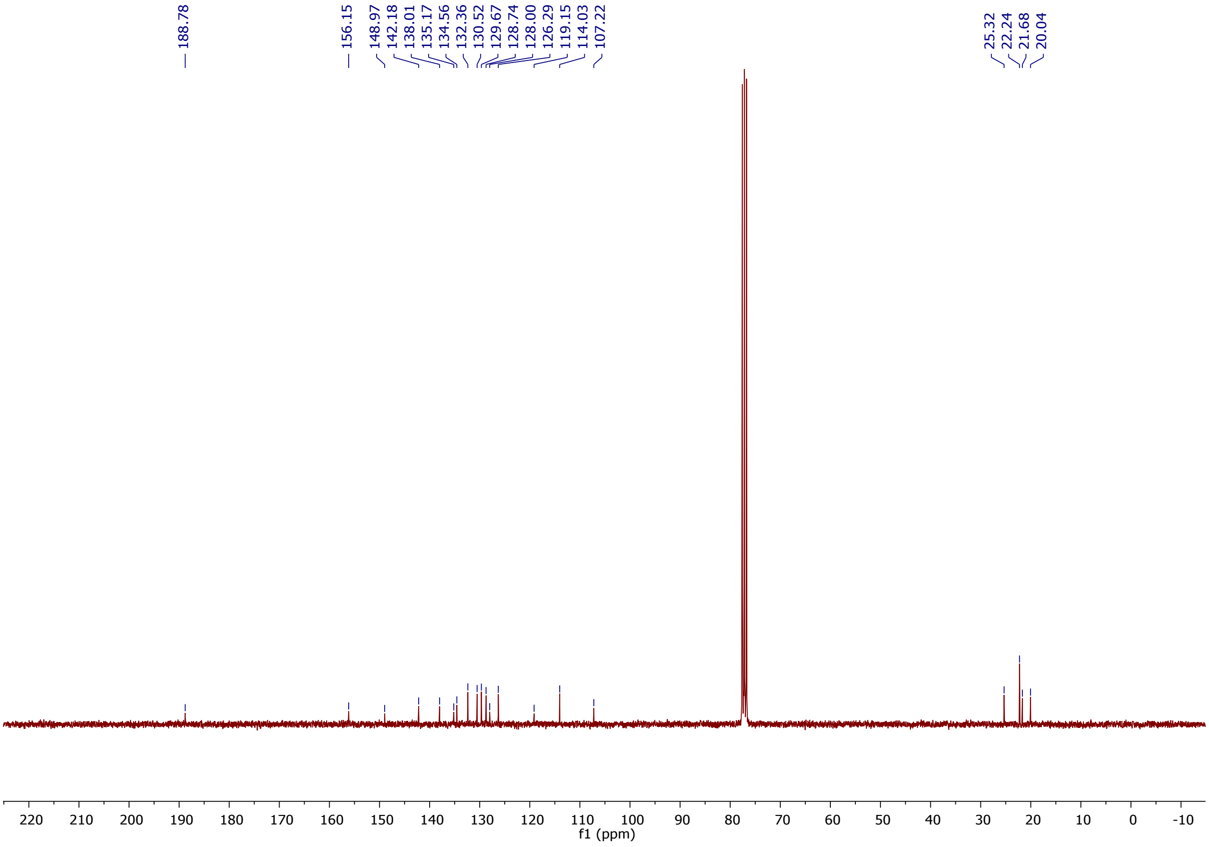
188.78
156.15 148.97 142.18 138.01 135.17 134.56 132.36 130.52 129.67 128.74 128.00 126.29 119.15 114.03 107.22
25.32 22.24 21.68 20.04
220 210 200 190 180 170 160 150 140 130 120 110 100 90 80 70 60 50 40 30 20 10 0 -10
f1 (ppm)

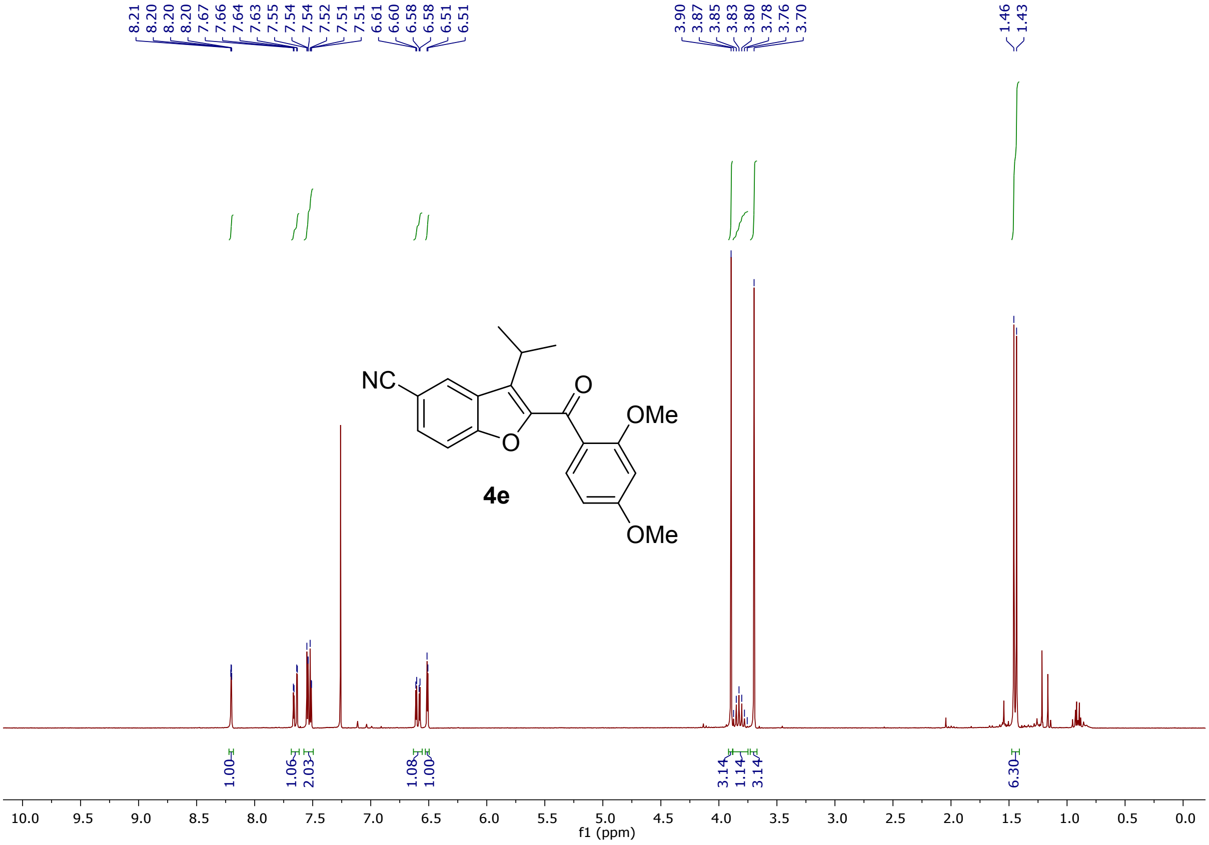
8.21 8.20 8.20 8.20 7.67 7.66 7.64 7.63 7.55 7.54 7.54 7.52 7.51 7.51 6.61 6.60 6.58 6.58 6.51 6.51
3.90 3.87 3.85 3.83 3.80 3.78 3.76 3.70
1.46 1.43
NC
O
OMe
O
OMe
4e
1.00 1.06 2.03 1.08 1.00 3.14 1.14 3.14 6.30
10.0 9.5 9.0 8.5 8.0 7.5 7.0 6.5 6.0 5.5 5.0 4.5 4.0 3.5 3.0 2.5 2.0 1.5 1.0 0.5 0.0
f1 (ppm)

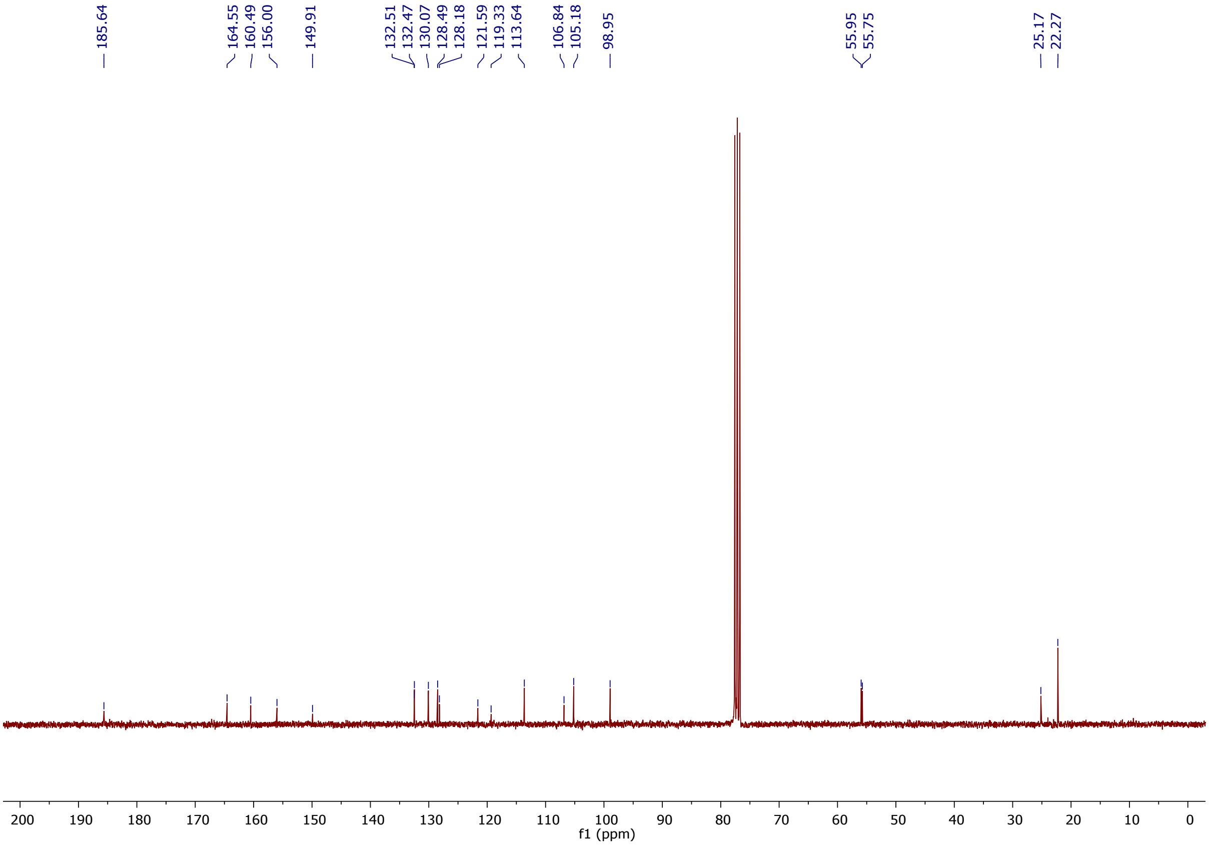
185.64
164.55 160.49 156.00
149.91
132.51 132.47 130.07 128.49 128.18 121.59 119.33 113.64
106.84 105.18
98.95
55.95 55.75
25.17 22.27
200 190 180 170 160 150 140 130 120 110 100 90 80 70 60 50 40 30 20 10 0
f1 (ppm)

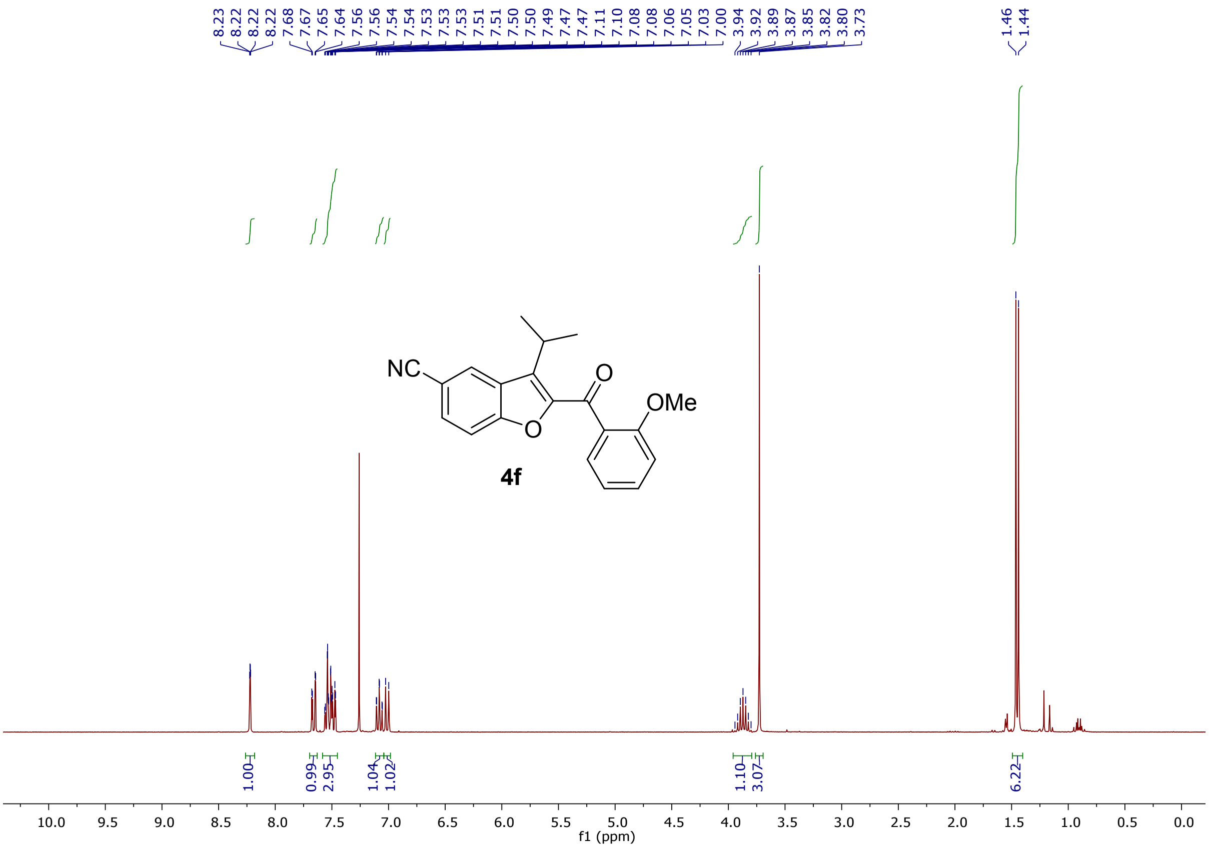
8.23
8.22
8.22
8.22
7.68
7.67
7.65
7.64
7.56
7.54
7.54
7.53
7.53
7.53
7.51
7.51
7.50
7.50
7.49
7.47
7.47
7.11
7.10
7.08
7.08
7.06
7.05
7.03
7.00
3.94
3.92
3.89
3.87
3.85
3.82
3.80
3.73
1.46
1.44
NC
O
OMe
O
4f
1.00
0.99
2.95
1.04
1.02
1.10
3.07
6.22
10.0 9.5 9.0 8.5 8.0 7.5 7.0 6.5 6.0 5.5 5.0 4.5 4.0 3.5 3.0 2.5 2.0 1.5 1.0 0.5 0.0
f1 (ppm)

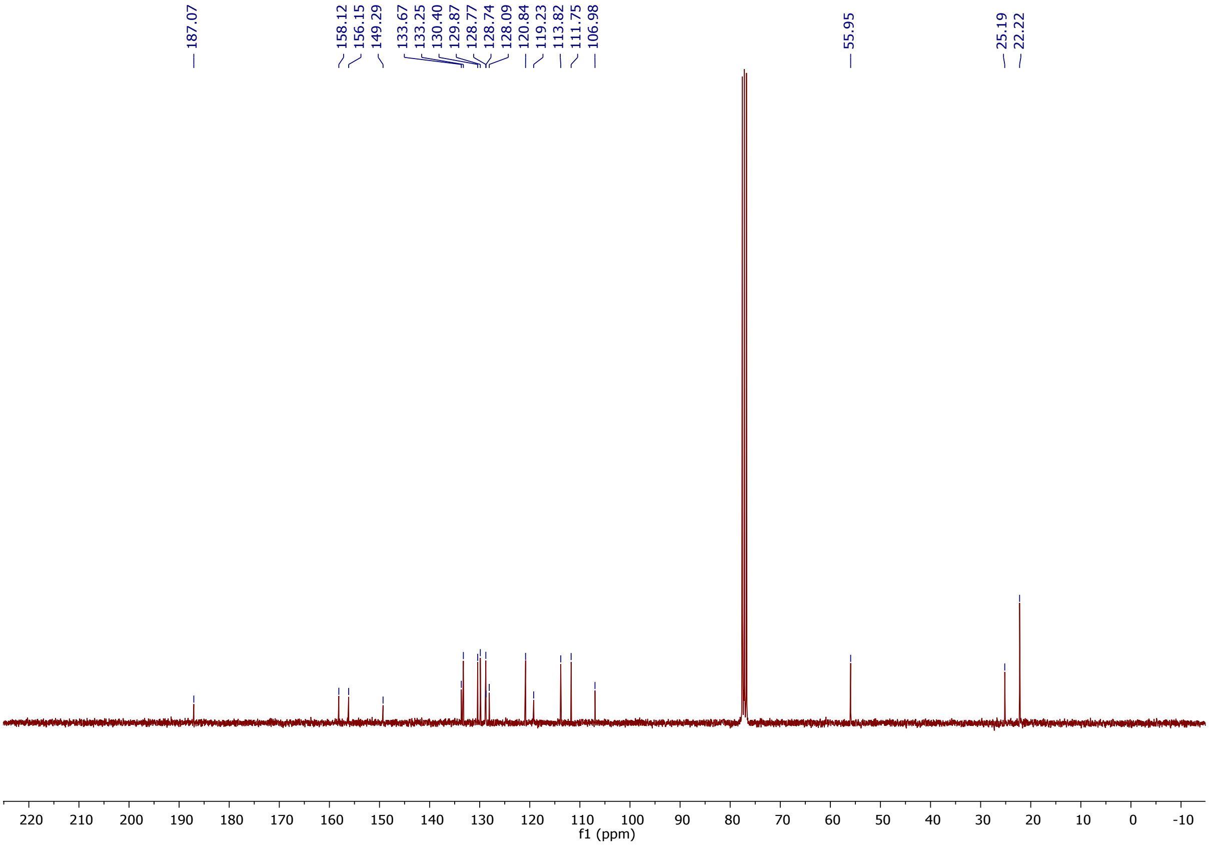
187.07
158.12
156.15
149.29
133.67
133.25
130.40
129.87
128.77
128.74
128.09
120.84
119.23
113.82
111.75
106.98
55.95
25.19
22.22
220 210 200 190 180 170 160 150 140 130 120 110 100 90 80 70 60 50 40 30 20 10 0 -10
f1 (ppm)

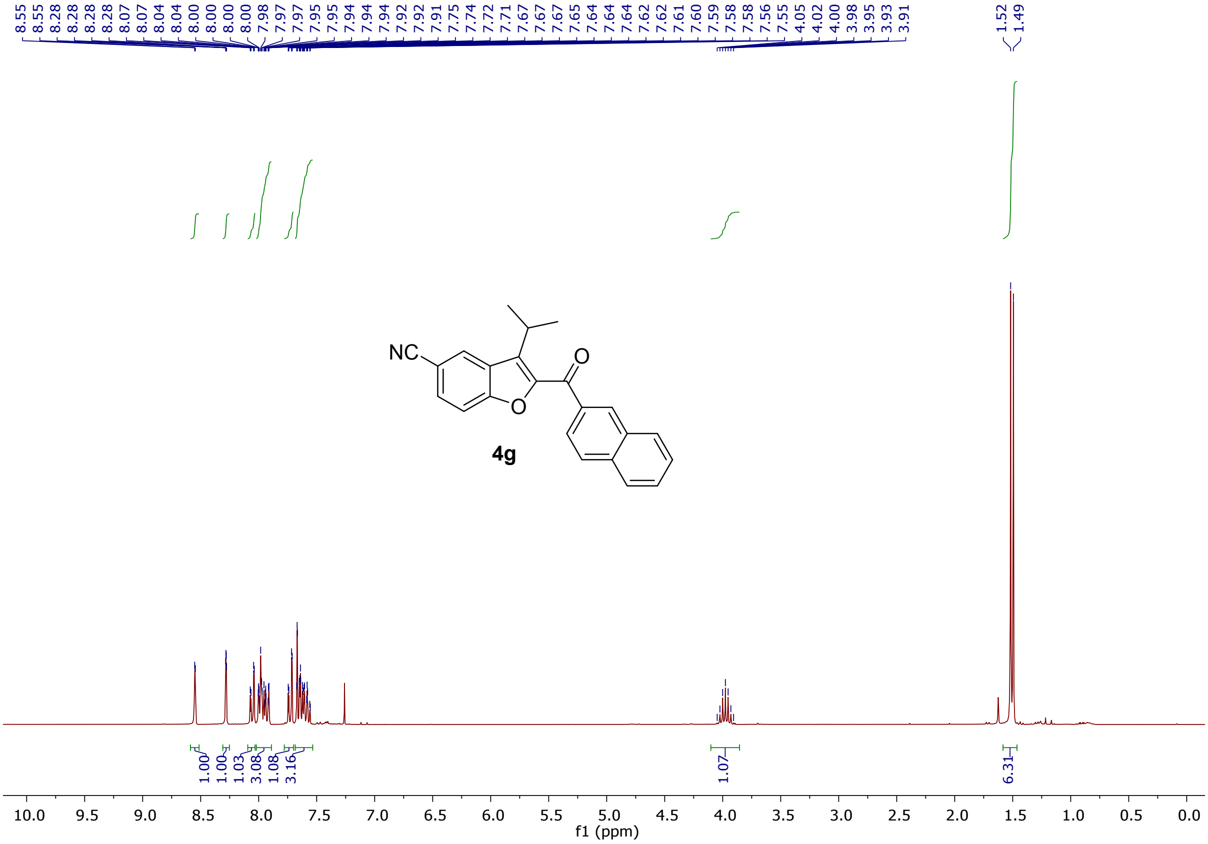
8.55 8.55 8.28 8.28 8.28 8.07 8.07 8.04 8.00 8.00 8.00 7.98 7.97 7.97 7.95 7.94 7.94 7.92 7.92 7.91 7.75 7.74 7.72 7.71 7.67 7.67 7.65 7.64 7.64 7.62 7.62 7.61 7.60 7.59 7.58 7.58 7.56 7.55 4.05 4.02 4.00 3.98 3.95 3.93 3.91
1.52 1.49
NC
O
O
4g
1.00 1.00 1.03 3.08 1.08 3.16
1.07
6.31
10.0 9.5 9.0 8.5 8.0 7.5 7.0 6.5 6.0 5.5 5.0 4.5 4.0 3.5 3.0 2.5 2.0 1.5 1.0 0.5 0.0
f1 (ppm)

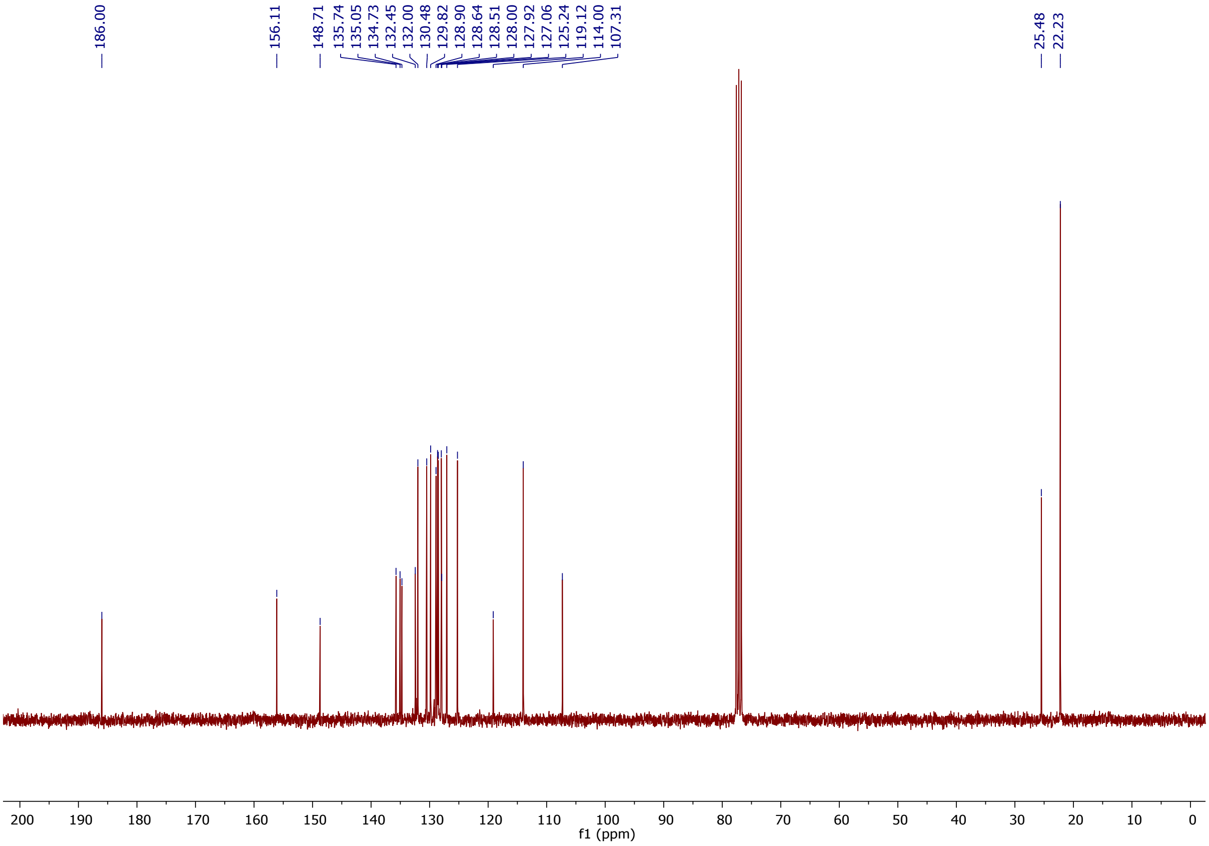
186.00
156.11
148.71
135.74 135.05 134.73 132.45 132.00 130.48 129.82 128.90 128.64 128.51 128.00 127.92 127.06 125.24 119.12 114.00 107.31
25.48 22.23
200 190 180 170 160 150 140 130 120 110 100 90 80 70 60 50 40 30 20 10 0
f1 (ppm)

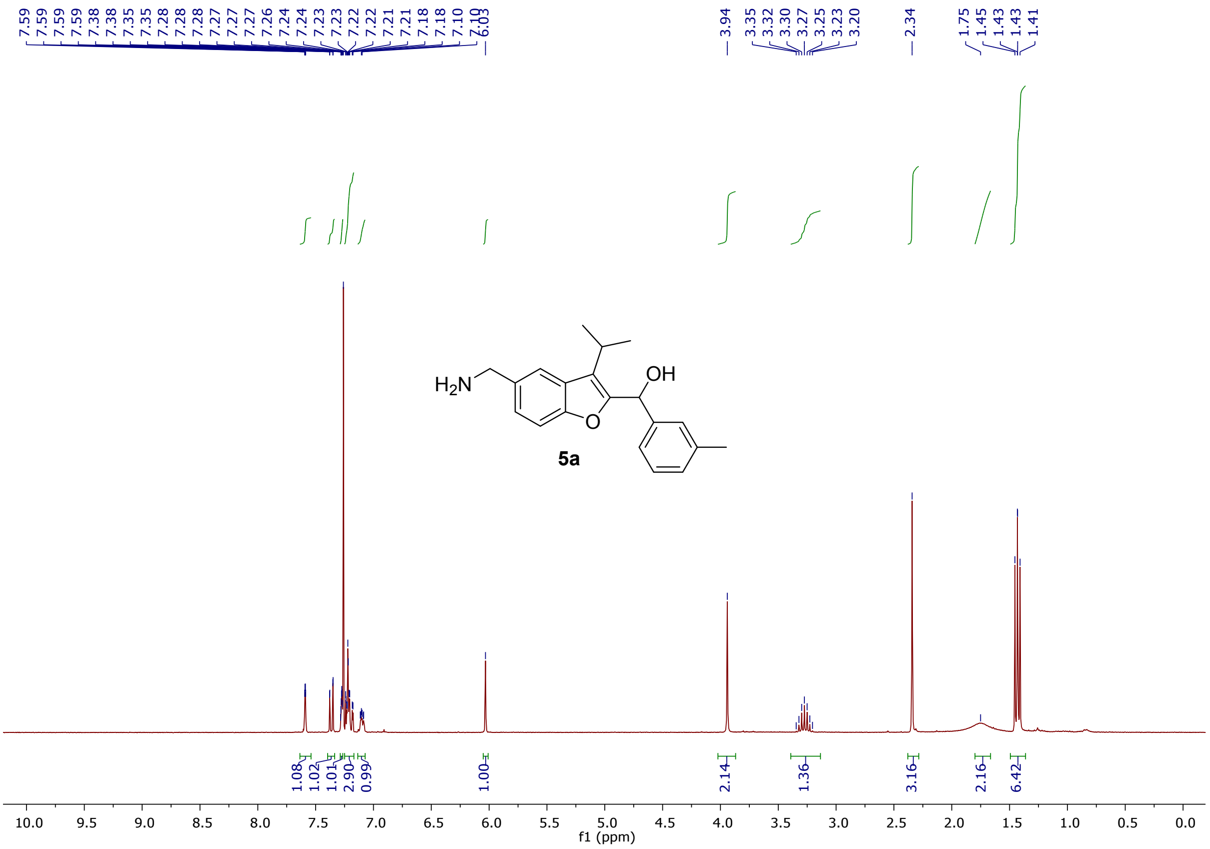
7.59 7.59 7.59 7.59 7.38 7.38 7.35 7.35 7.28 7.28 7.28 7.27 7.27 7.27 7.26 7.24 7.24 7.23 7.23 7.22 7.22 7.21 7.21 7.18 7.10 7.10 6.03
3.94 3.35 3.32 3.30 3.27 3.25 3.23 3.20
2.34
1.75 1.45 1.43 1.43 1.41
H2N
OH
O
5a
1.08 1.02 1.01 2.90 0.99 1.00 2.14 1.36 3.16 2.16 6.42
10.0 9.5 9.0 8.5 8.0 7.5 7.0 6.5 6.0 5.5 5.0 4.5 4.0 3.5 3.0 2.5 2.0 1.5 1.0 0.5 0.0
f1 (ppm)

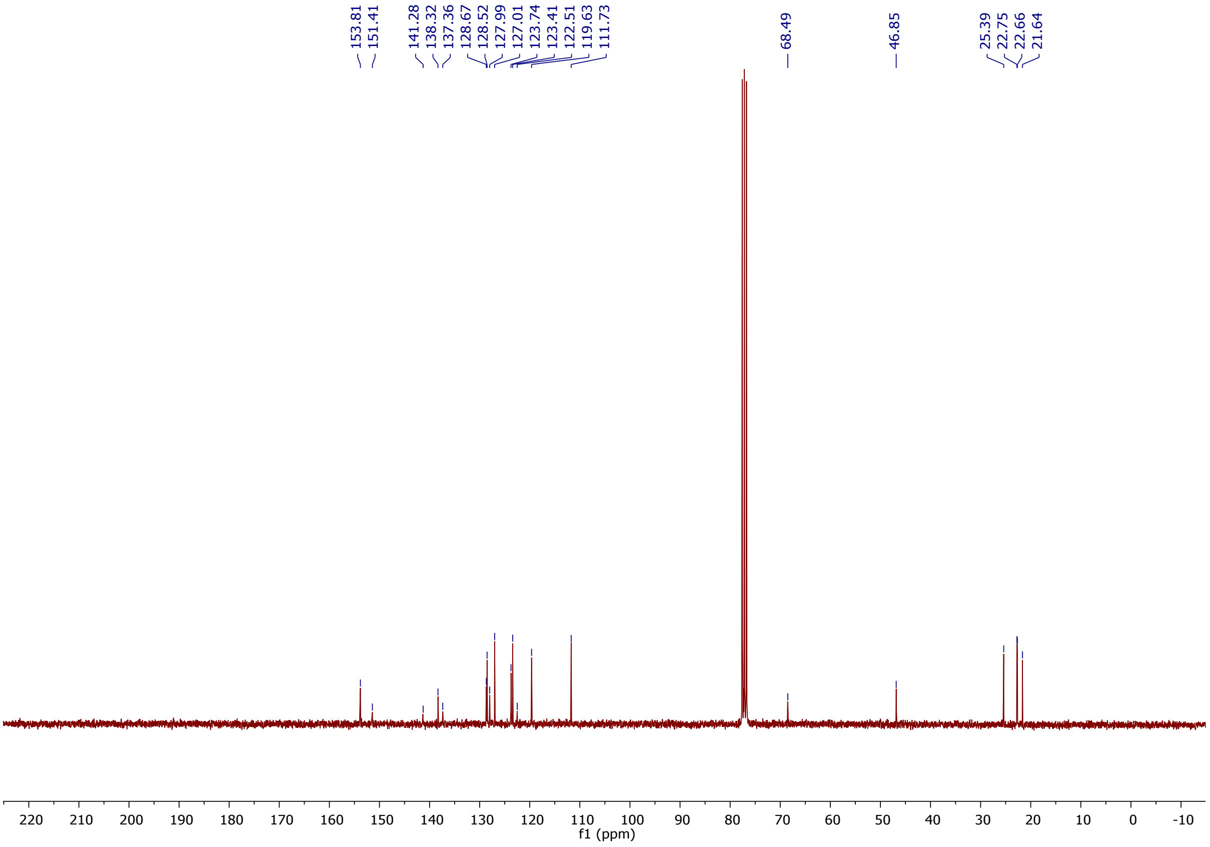
153.81 151.41
141.28 138.32 137.36 128.67 128.52 127.99 127.01 123.74 123.41 122.51 119.63 111.73
68.49
46.85
25.39 22.75 22.66 21.64
220 210 200 190 180 170 160 150 140 130 120 110 100 90 80 70 60 50 40 30 20 10 0 -10
f1 (ppm)

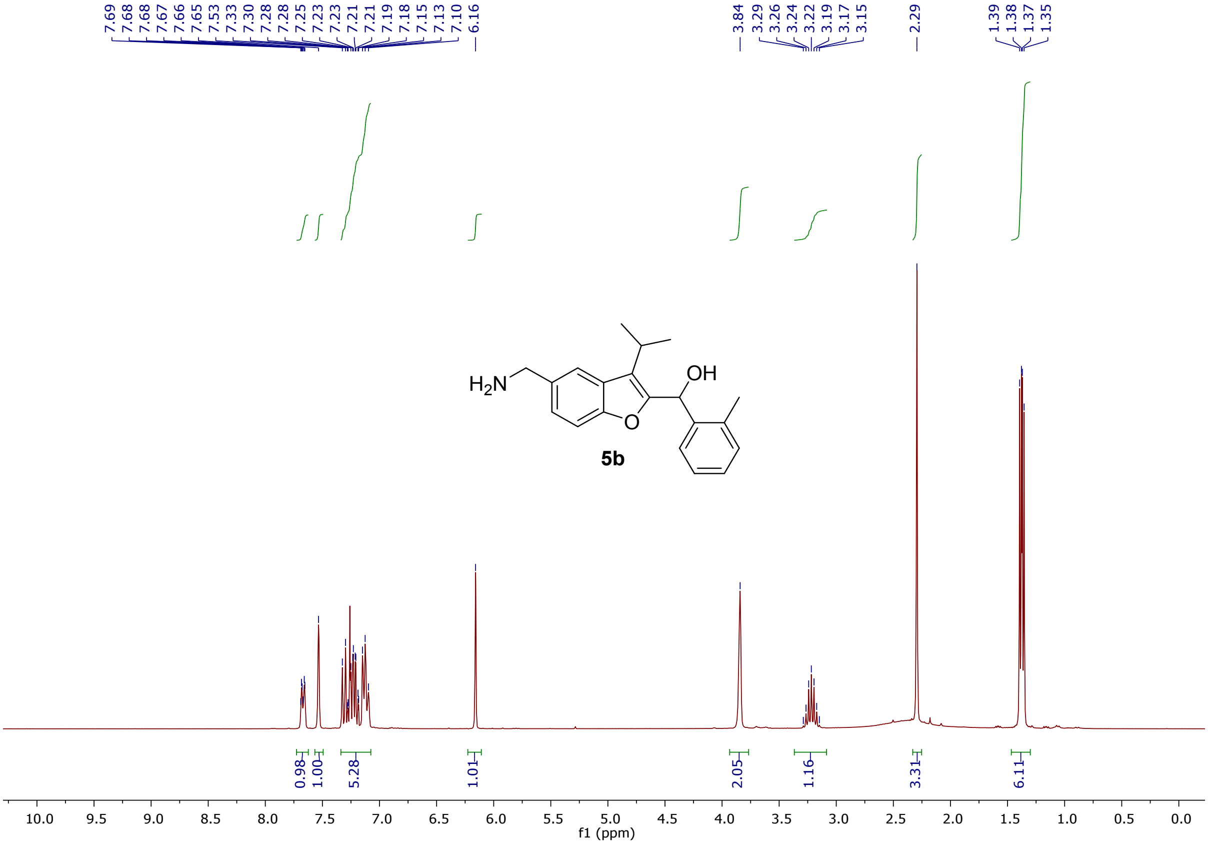
7.69 7.68 7.68 7.67 7.66 7.65 7.53 7.33 7.30 7.28 7.28 7.25 7.23 7.23 7.21 7.21 7.19 7.18 7.15 7.13 7.10 6.16
3.84 3.29 3.26 3.24 3.22 3.19 3.17 3.15
2.29
1.39 1.38 1.37 1.35
H_2N
OH
O
5b
0.98 1.00 5.28 1.01 2.05 1.16 3.31 6.11
10.0 9.5 9.0 8.5 8.0 7.5 7.0 6.5 6.0 5.5 5.0 4.5 4.0 3.5 3.0 2.5 2.0 1.5 1.0 0.5 0.0
f1 (ppm)

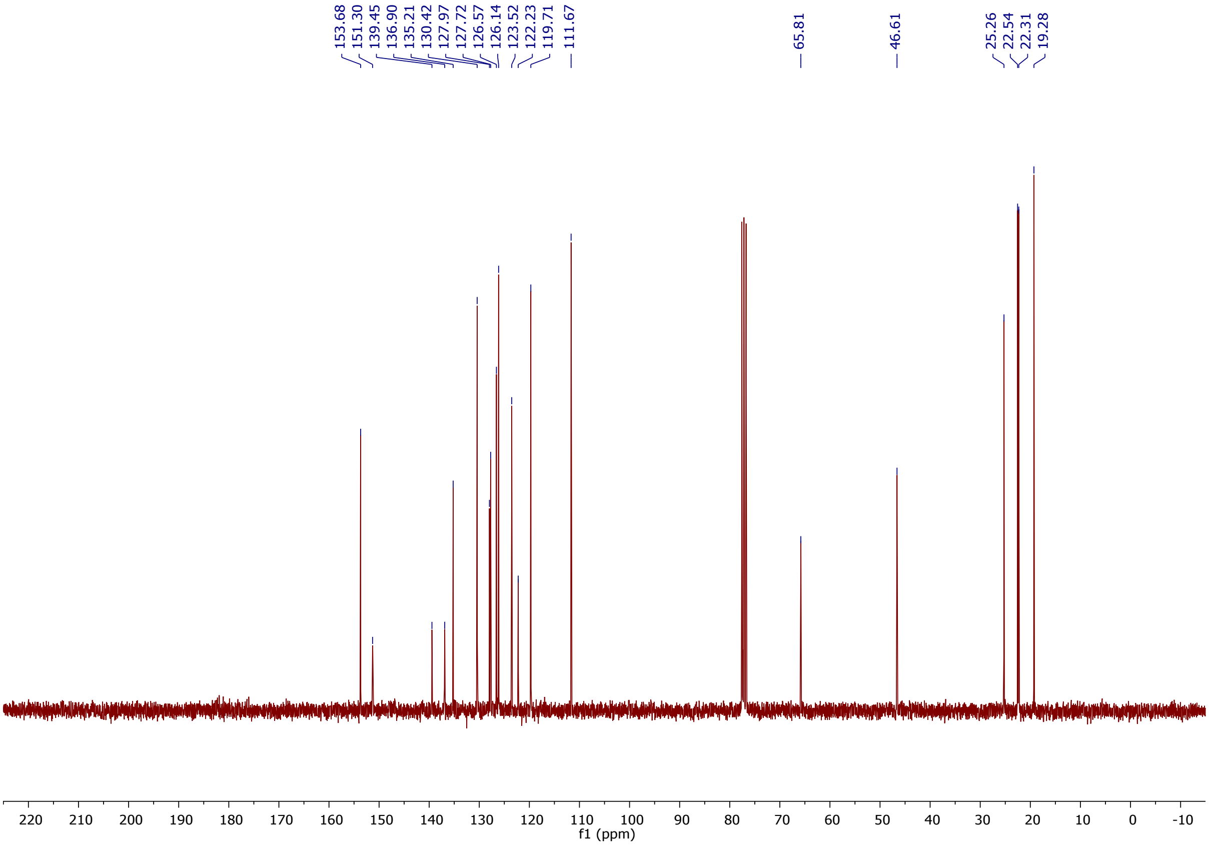
153.68 151.30 139.45 136.90 135.21 130.42 127.97 127.72 126.57 126.14 123.52 122.23 119.71 111.67
65.81
46.61
25.26 22.54 22.31 19.28
220 210 200 190 180 170 160 150 140 130 120 110 100 90 80 70 60 50 40 30 20 10 0 -10
f1 (ppm)

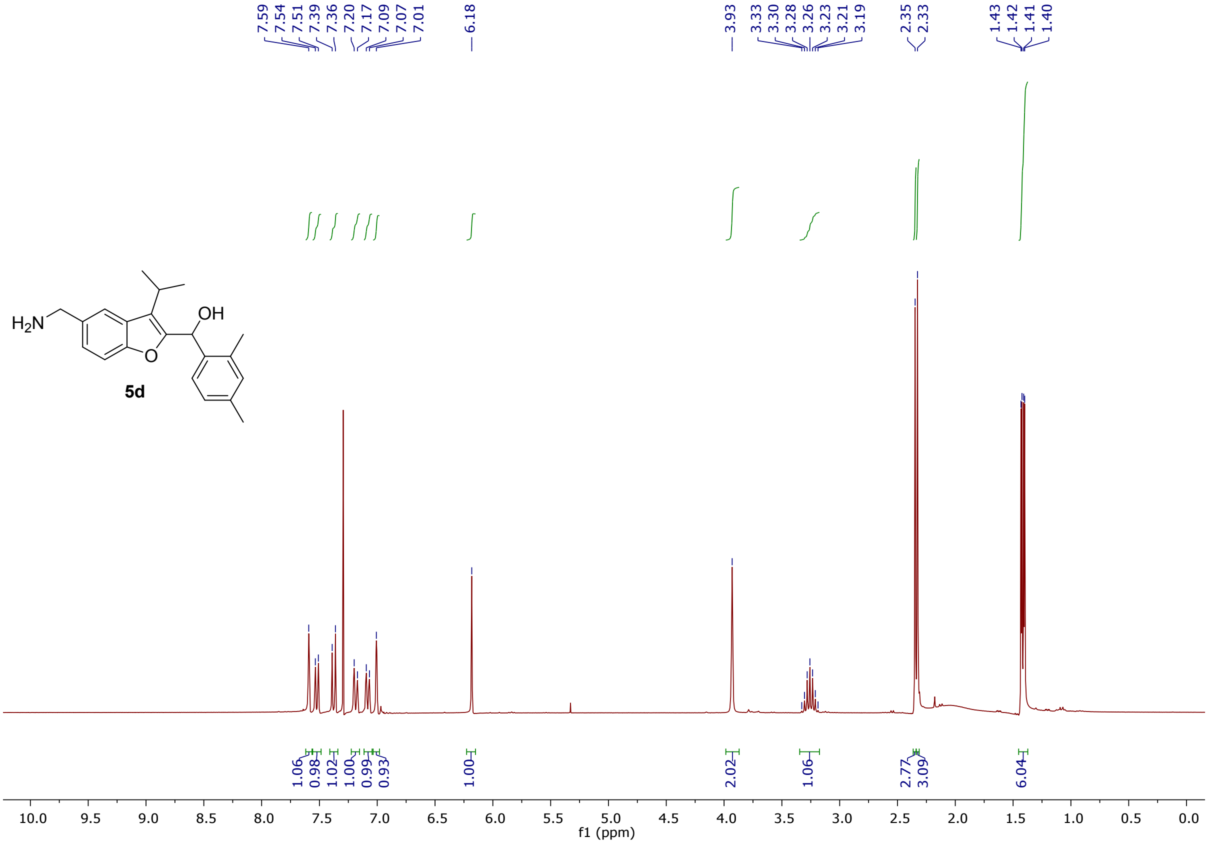
7.59
7.54
7.51
7.39
7.36
7.20
7.17
7.09
7.07
7.01
6.18
3.93
3.33
3.30
3.28
3.26
3.23
3.21
3.19
2.35
2.33
1.43
1.42
1.41
1.40
1.06
0.98
1.02
1.00
0.99
0.93
1.00
2.02
1.06
2.77
3.09
6.04
10.0 9.5 9.0 8.5 8.0 7.5 7.0 6.5 6.0 5.5 5.0 4.5 4.0 3.5 3.0 2.5 2.0 1.5 1.0 0.5 0.0
f1 (ppm)
H2N
OH
O

5d

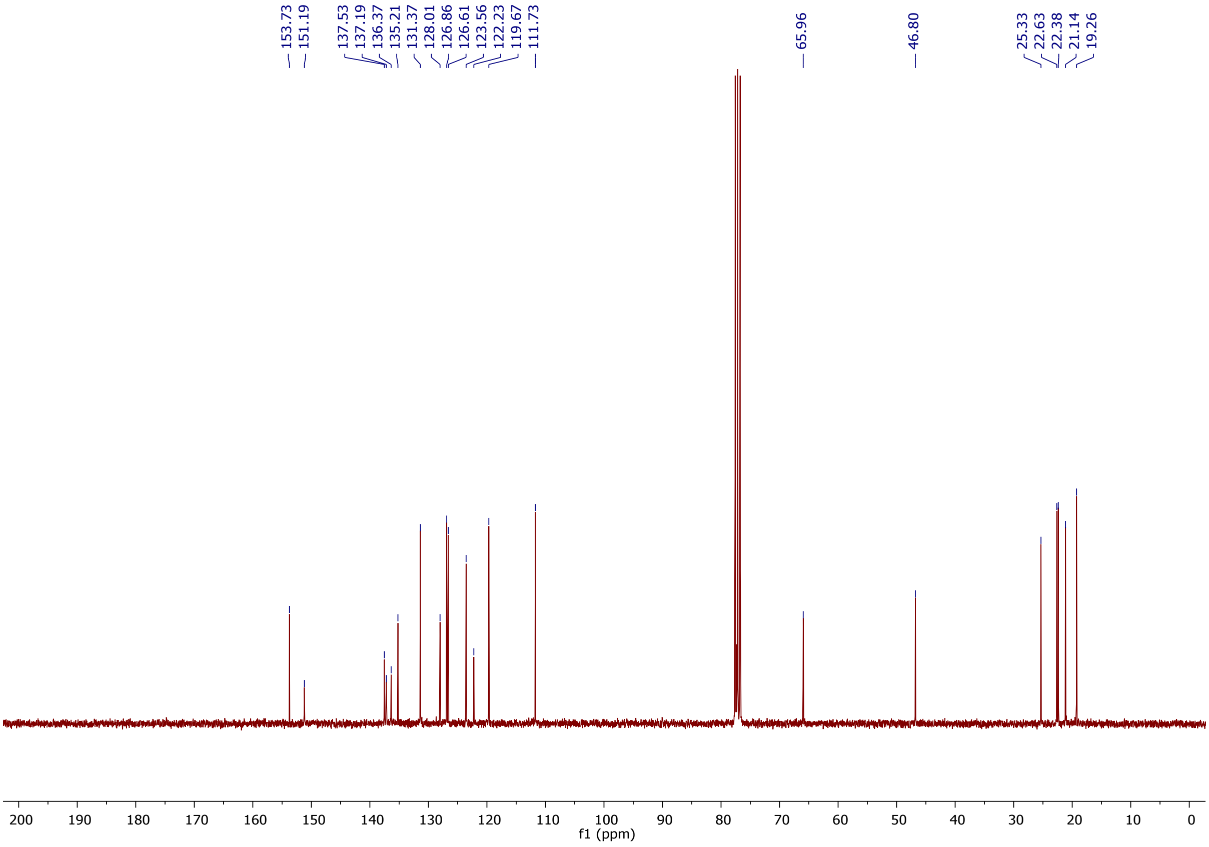
153.73
151.19
137.53
137.19
136.37
135.21
131.37
128.01
126.86
126.61
123.56
122.23
119.67
111.73
65.96
46.80
25.33
22.63
22.38
21.14
19.26
200 190 180 170 160 150 140 130 120 110 100 90 80 70 60 50 40 30 20 10 0
f1 (ppm)

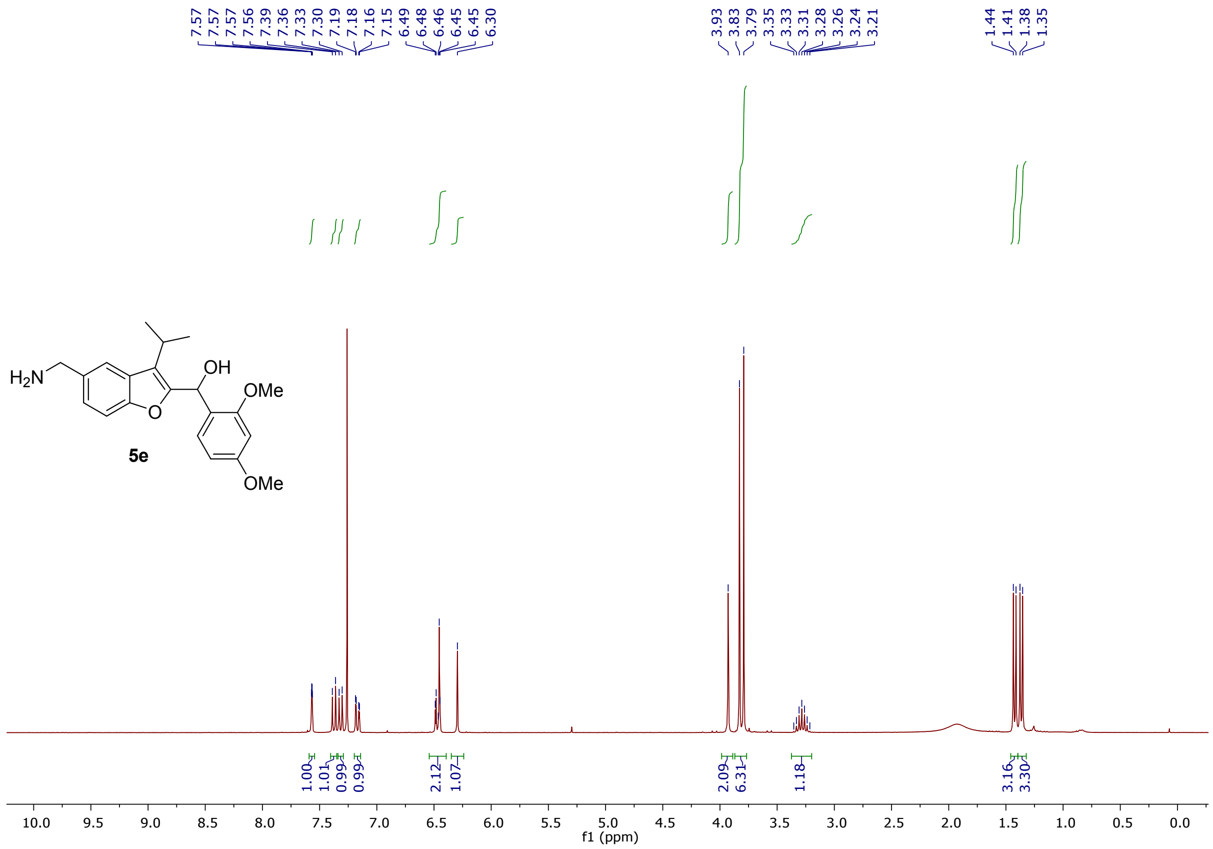
7.57 7.57 7.57 7.56 7.39 7.36 7.33 7.30 7.19 7.18 7.16 7.15 6.49 6.48 6.46 6.45 6.45 6.30
3.93 3.83 3.79 3.35 3.33 3.31 3.28 3.26 3.24 3.21
1.44 1.41 1.38 1.35
H2N
OH
OMe
O
OMe
5e
1.00 1.01 0.99 0.99 2.12 1.07 2.09 6.31 1.18 3.16 3.30
10.0 9.5 9.0 8.5 8.0 7.5 7.0 6.5 6.0 5.5 5.0 4.5 4.0 3.5 3.0 2.5 2.0 1.5 1.0 0.5 0.0
f1 (ppm)

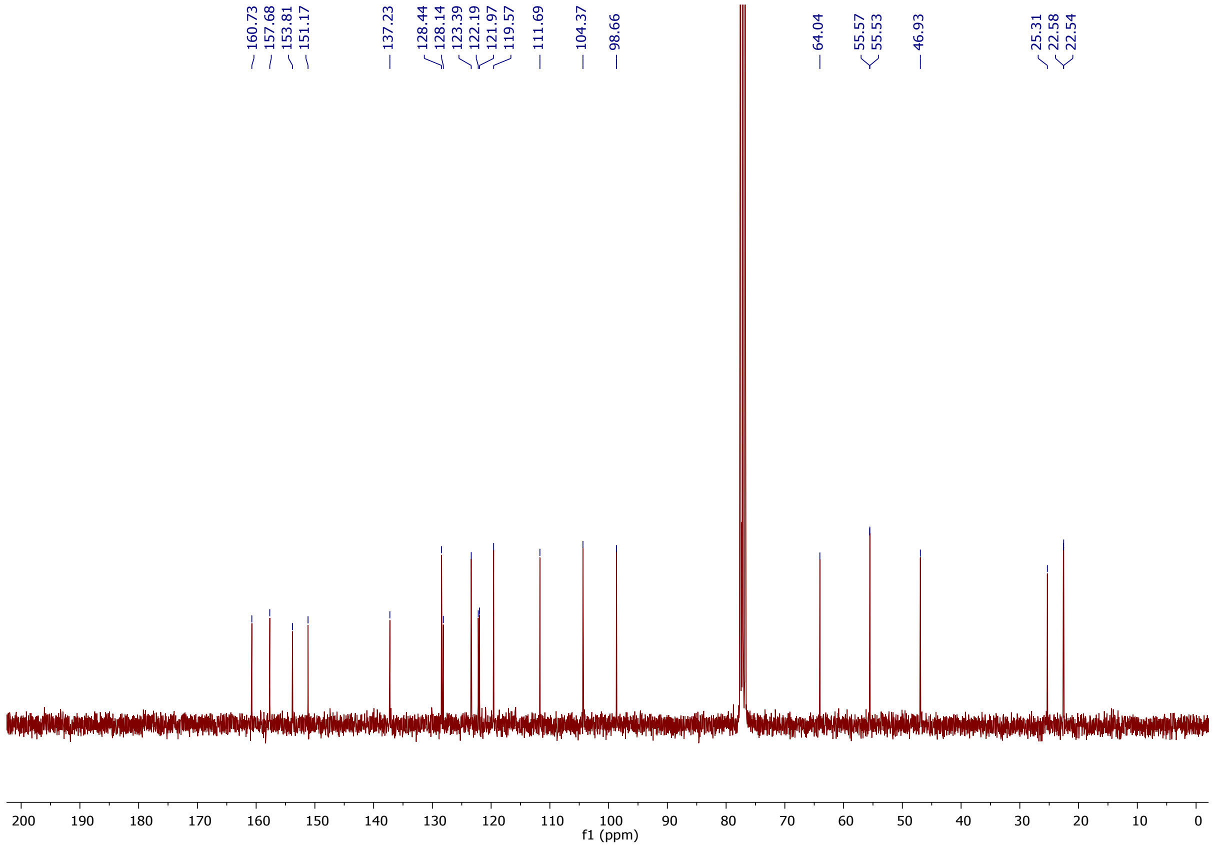
160.73 157.68 153.81 151.17 137.23 128.44 128.14 123.39 122.19 121.97 119.57 111.69 104.37 98.66
64.04 55.57 55.53 46.93 25.31 22.58 22.54
200 190 180 170 160 150 140 130 120 110 100 90 80 70 60 50 40 30 20 10 0
f1 (ppm)

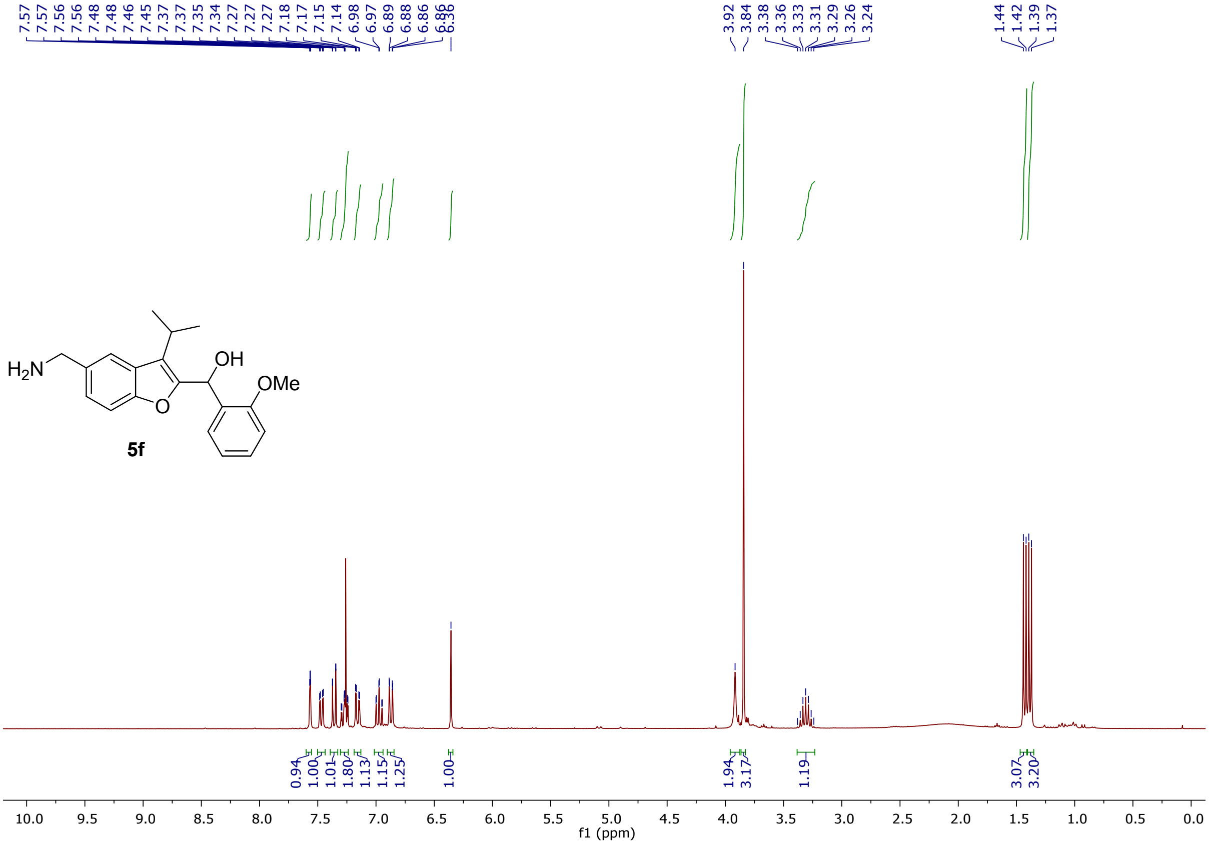
7.57 7.57 7.56 7.56 7.48 7.48 7.46 7.45 7.37 7.37 7.35 7.34 7.27 7.27 7.27 7.27 7.18 7.17 7.15 7.14 6.98 6.97 6.89 6.88 6.86 6.86 6.36
3.92 3.84 3.38 3.36 3.33 3.31 3.29 3.26 3.24
1.44 1.42 1.39 1.37
H2N
OH
OMe
O
5f
0.94 1.00 1.01 1.80 1.13 1.15 1.25 1.00 1.94 3.17 1.19 3.07 3.20
10.0 9.5 9.0 8.5 8.0 7.5 7.0 6.5 6.0 5.5 5.0 4.5 4.0 3.5 3.0 2.5 2.0 1.5 1.0 0.5 0.0
f1 (ppm)

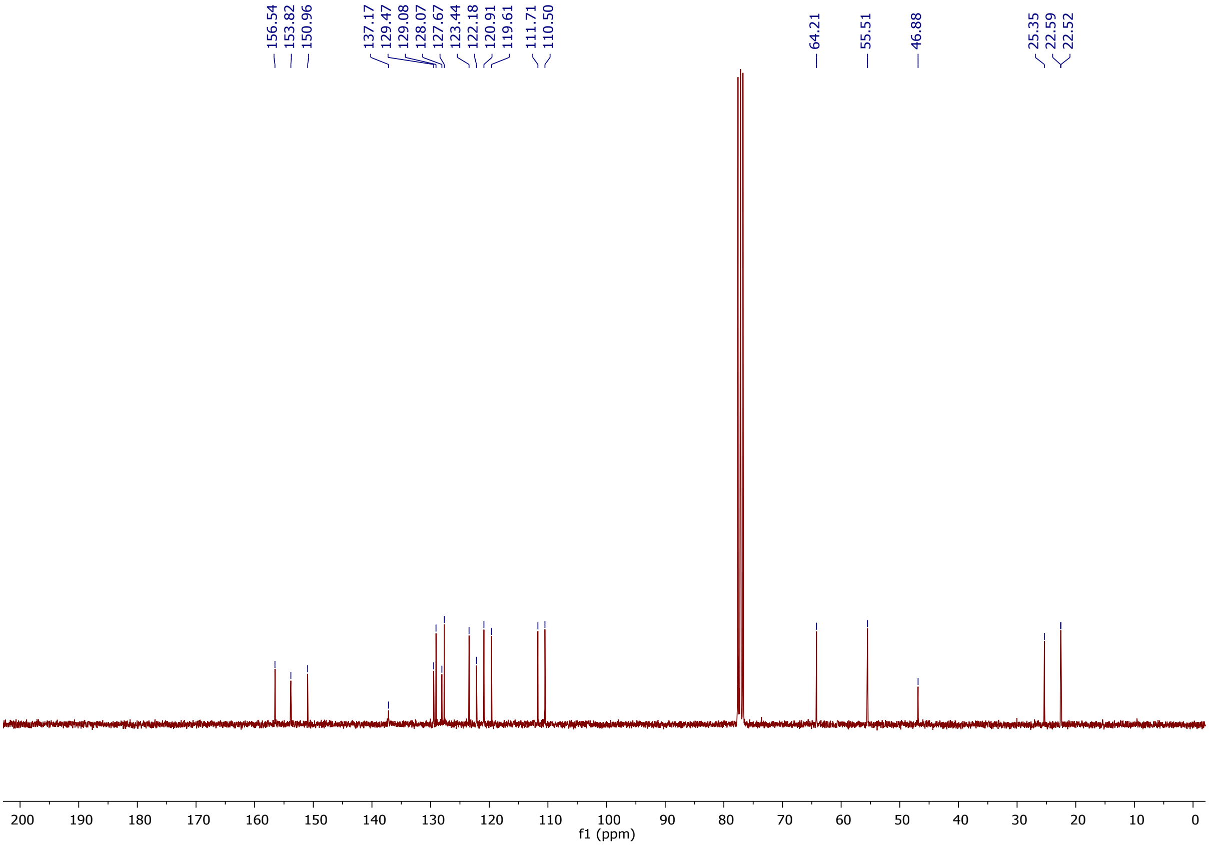
156.54 153.82 150.96
137.17 129.47 129.08 128.07 127.67 123.44 122.18 120.91 119.61 111.71 110.50
64.21
55.51
46.88
25.35 22.59 22.52
200 190 180 170 160 150 140 130 120 110 100 90 80 70 60 50 40 30 20 10 0
f1 (ppm)

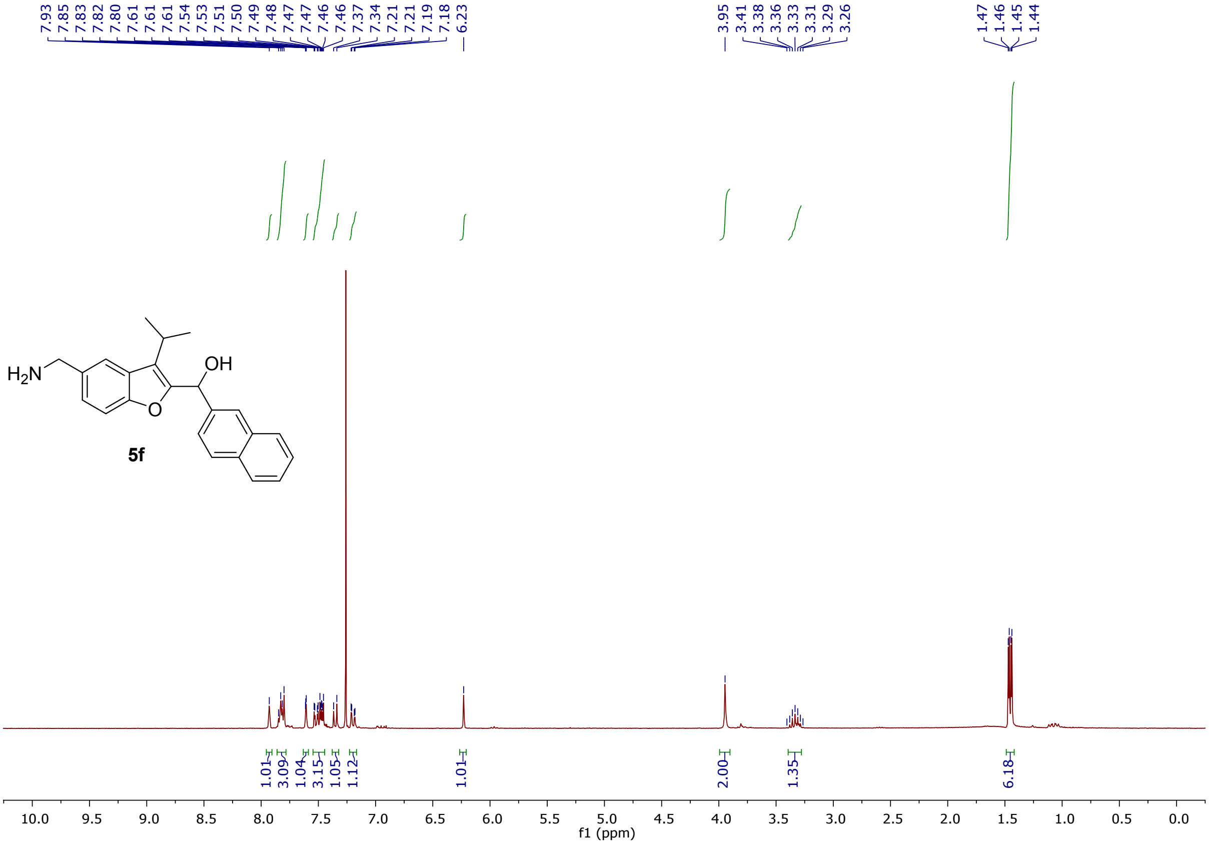
7.93 7.85 7.83 7.82 7.80 7.61 7.61 7.61 7.54 7.53 7.51 7.50 7.49 7.48 7.47 7.47 7.46 7.46 7.37 7.34 7.21 7.21 7.19 7.18 6.23
3.95 3.41 3.38 3.36 3.33 3.31 3.29 3.26
1.47 1.46 1.45 1.44
H2N
OH
O
5f
1.01 3.09 1.04 3.15 1.05 1.12 1.01 2.00 1.35 6.18
10.0 9.5 9.0 8.5 8.0 7.5 7.0 6.5 6.0 5.5 5.0 4.5 4.0 3.5 3.0 2.5 2.0 1.5 1.0 0.5 0.0
f1 (ppm)

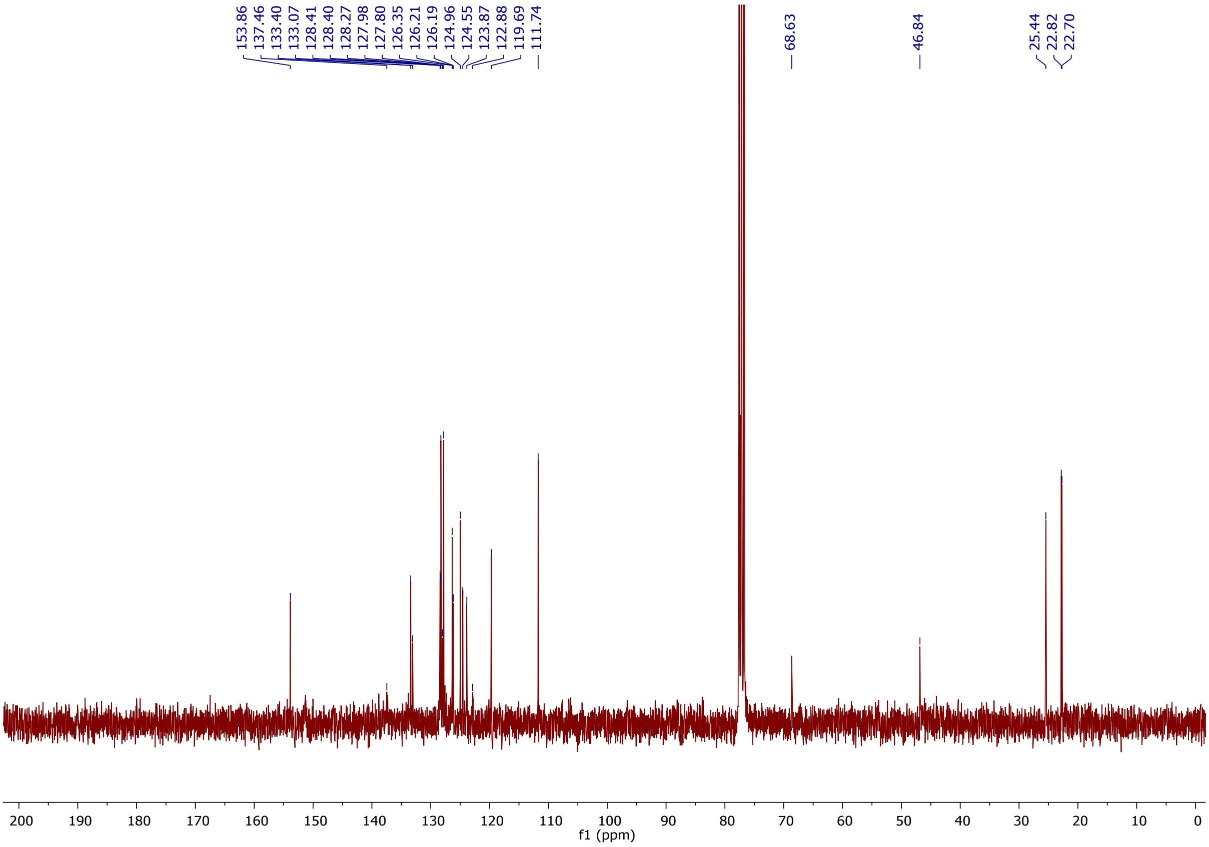
153.86 137.46 133.40 133.07 128.41 128.40 128.27 127.98 127.80 126.35 126.21 126.19 124.96 124.55 123.87 122.88 119.69 111.74
68.63
46.84
25.44 22.82 22.70
200 190 180 170 160 150 140 130 120 110 100 90 80 70 60 50 40 30 20 10 0
f1 (ppm)

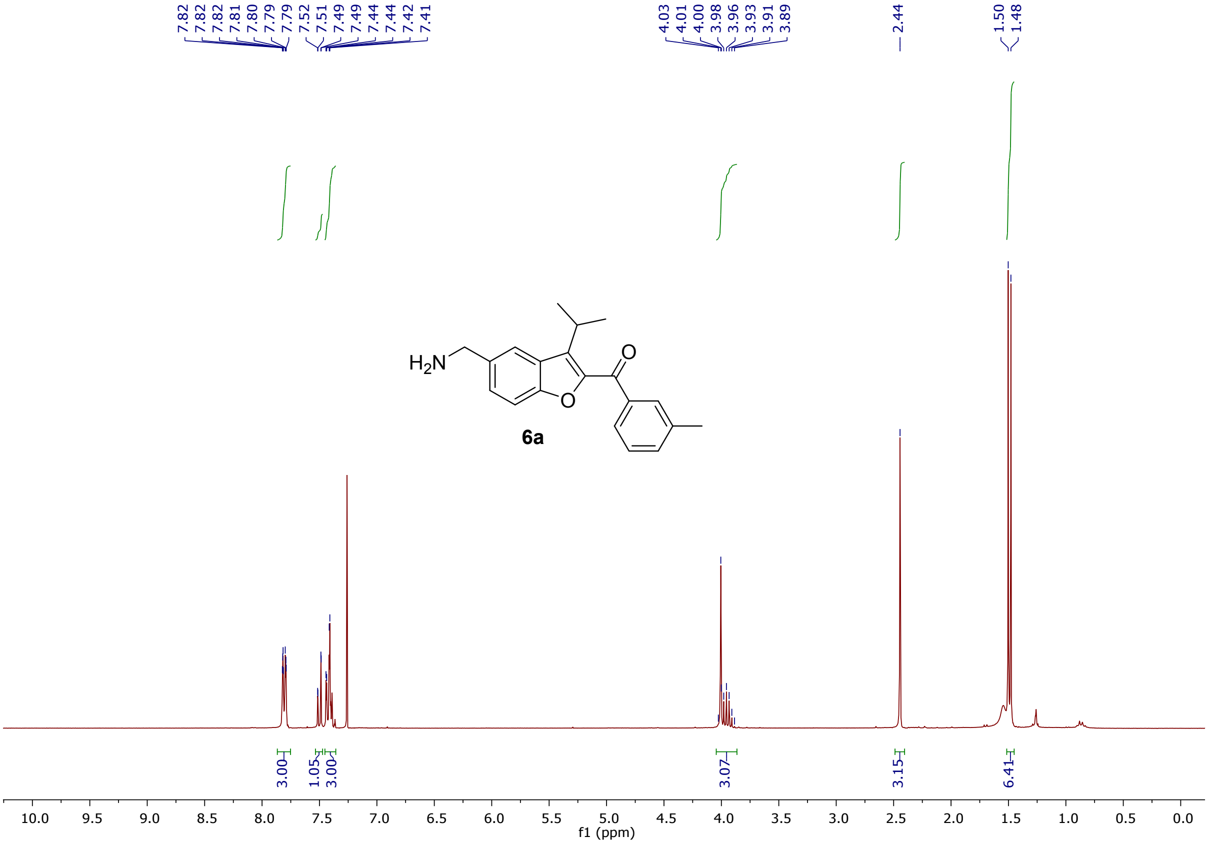
7.82
7.82
7.82
7.81
7.80
7.79
7.79
7.52
7.51
7.49
7.49
7.44
7.44
7.42
7.41
4.03
4.01
4.00
3.98
3.96
3.93
3.91
3.89
2.44
1.50
1.48
H_2N
O
O
6a
3.00
1.05
3.00
3.07
3.15
6.41
10.0 9.5 9.0 8.5 8.0 7.5 7.0 6.5 6.0 5.5 5.0 4.5 4.0 3.5 3.0 2.5 2.0 1.5 1.0 0.5 0.0
f1 (ppm)

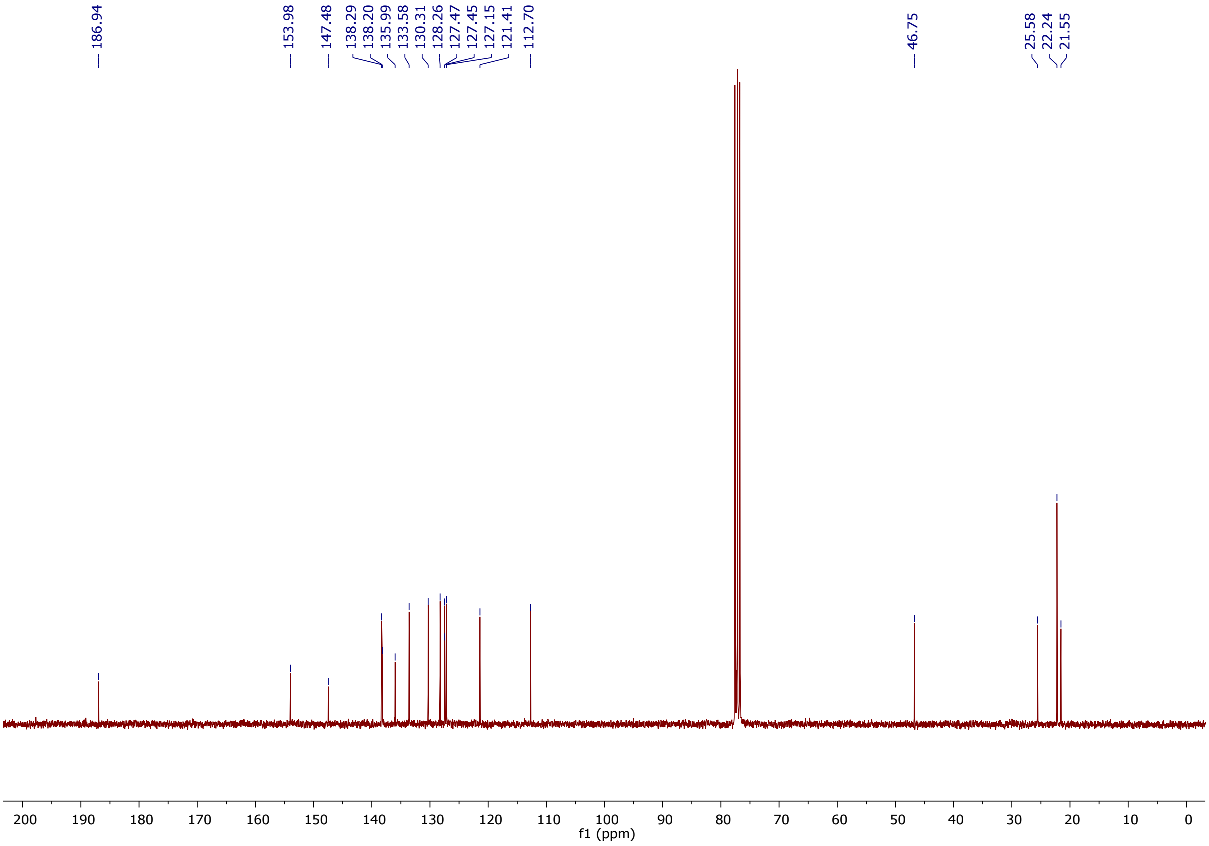
186.94
153.98
147.48
138.29
138.20
135.99
133.58
130.31
128.26
127.47
127.45
127.15
121.41
112.70
46.75
25.58
22.24
21.55
200 190 180 170 160 150 140 130 120 110 100 90 80 70 60 50 40 30 20 10 0
f1 (ppm)

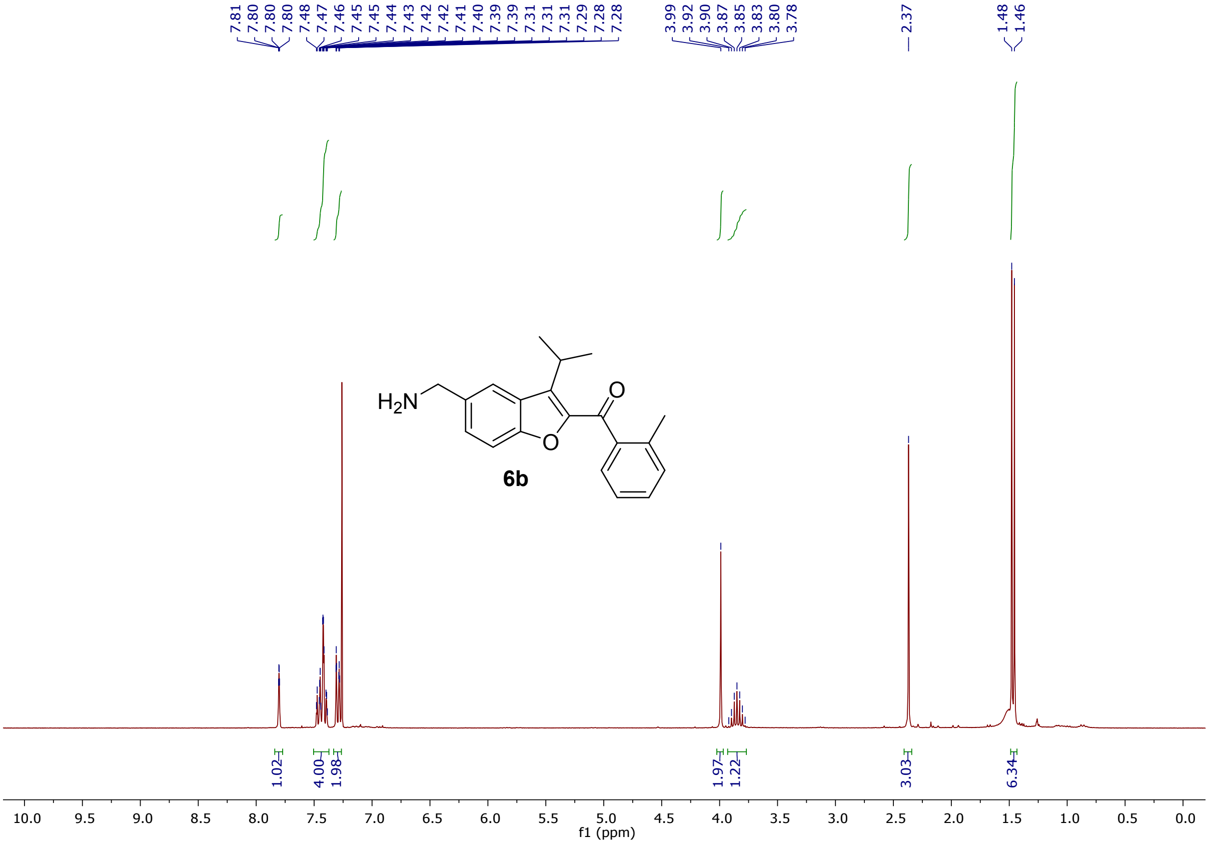
7.81 7.80 7.80 7.80 7.48 7.47 7.46 7.45 7.45 7.44 7.43 7.42 7.42 7.41 7.40 7.39 7.39 7.31 7.31 7.31 7.29 7.28 7.28
3.99 3.92 3.90 3.87 3.85 3.83 3.80 3.78
2.37
1.48 1.46
H_2N
O
O
6b
1.02 4.00 1.98 1.97 1.22 3.03 6.34
10.0 9.5 9.0 8.5 8.0 7.5 7.0 6.5 6.0 5.5 5.0 4.5 4.0 3.5 3.0 2.5 2.0 1.5 1.0 0.5 0.0
f1 (ppm)

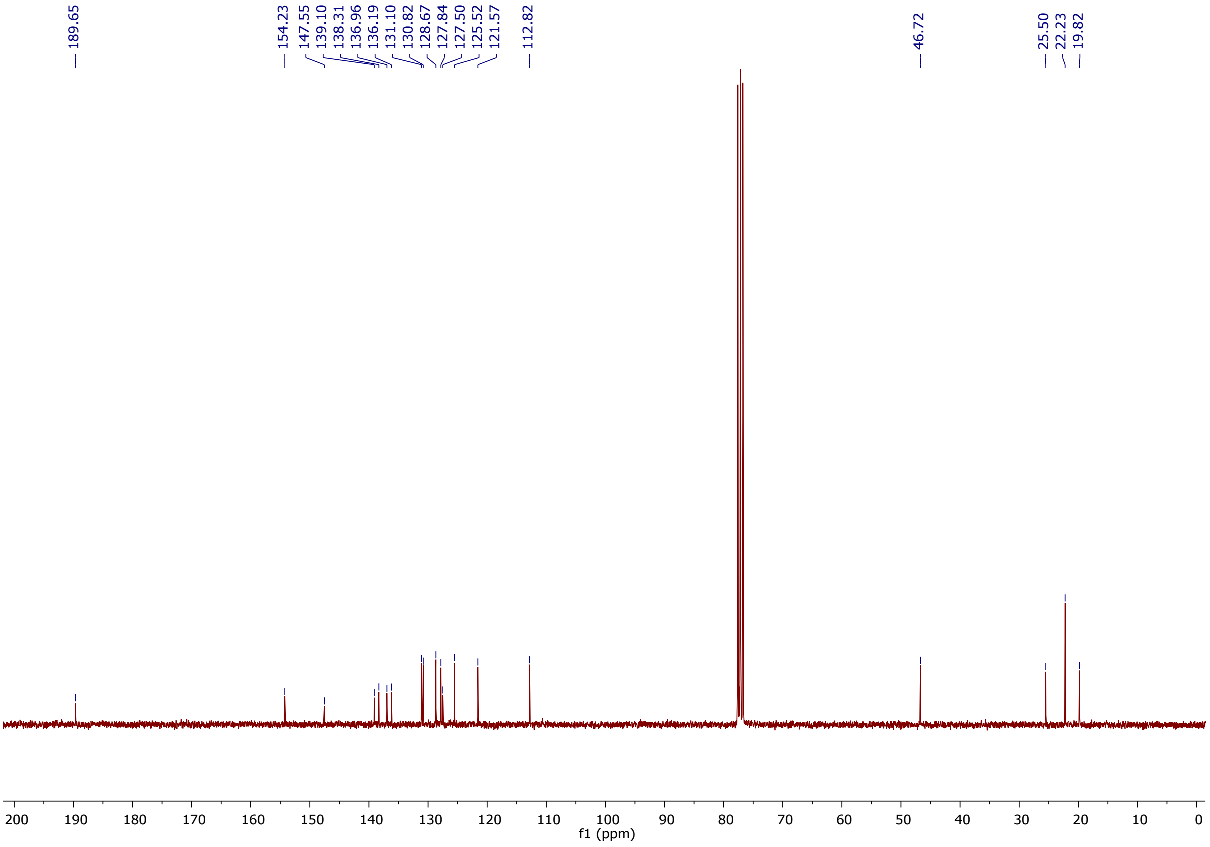
189.65
154.23 147.55 139.10 138.31 136.96 136.19 131.10 130.82 128.67 127.84 127.50 125.52 121.57
112.82
46.72
25.50 22.23 19.82
200 190 180 170 160 150 140 130 120 110 100 90 80 70 60 50 40 30 20 10 0
f1 (ppm)

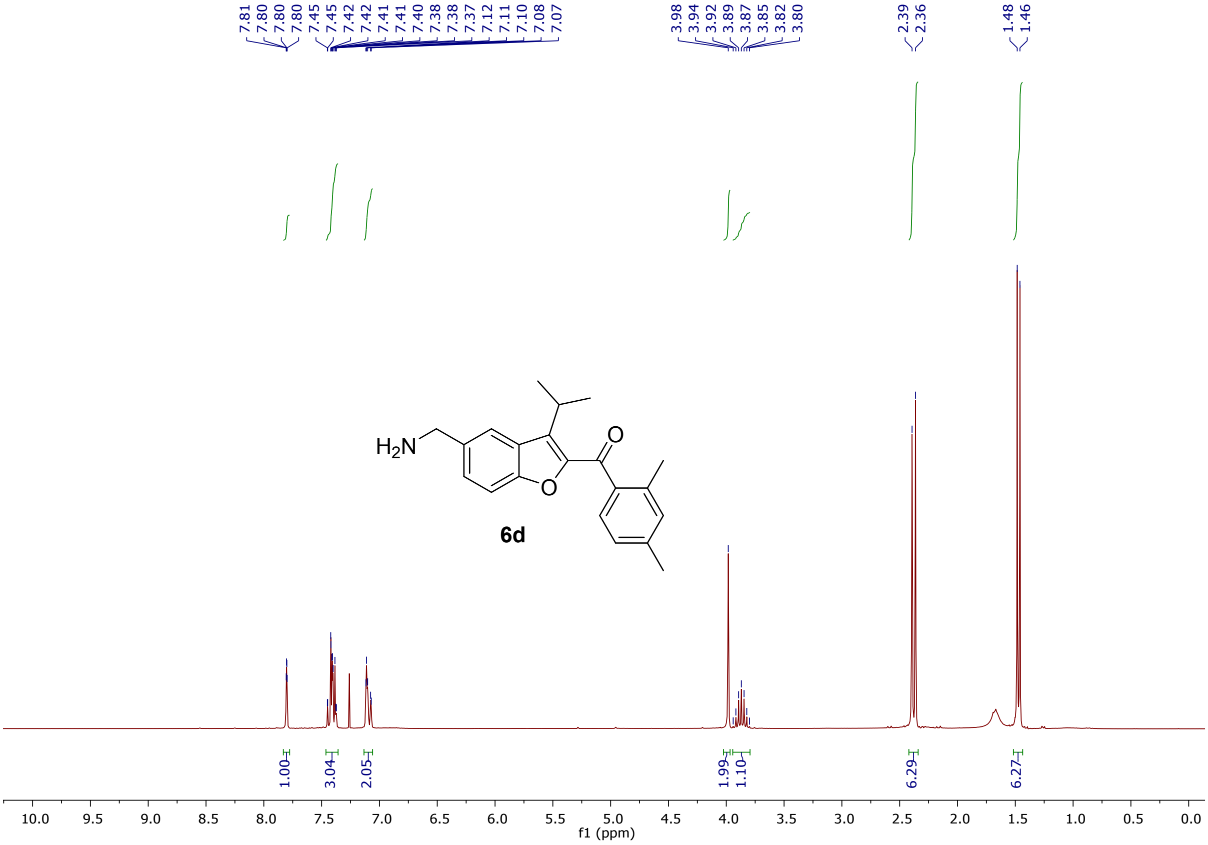
7.81 7.80 7.80 7.80 7.45 7.45 7.42 7.42 7.41 7.41 7.40 7.38 7.38 7.37 7.12 7.11 7.10 7.08 7.07
3.98 3.94 3.92 3.89 3.87 3.85 3.82 3.80
2.39 2.36
1.48 1.46
H2N
O
O
6d
1.00 3.04 2.05 1.99 1.10 6.29 6.27
10.0 9.5 9.0 8.5 8.0 7.5 7.0 6.5 6.0 5.5 5.0 4.5 4.0 3.5 3.0 2.5 2.0 1.5 1.0 0.5 0.0
f1 (ppm)

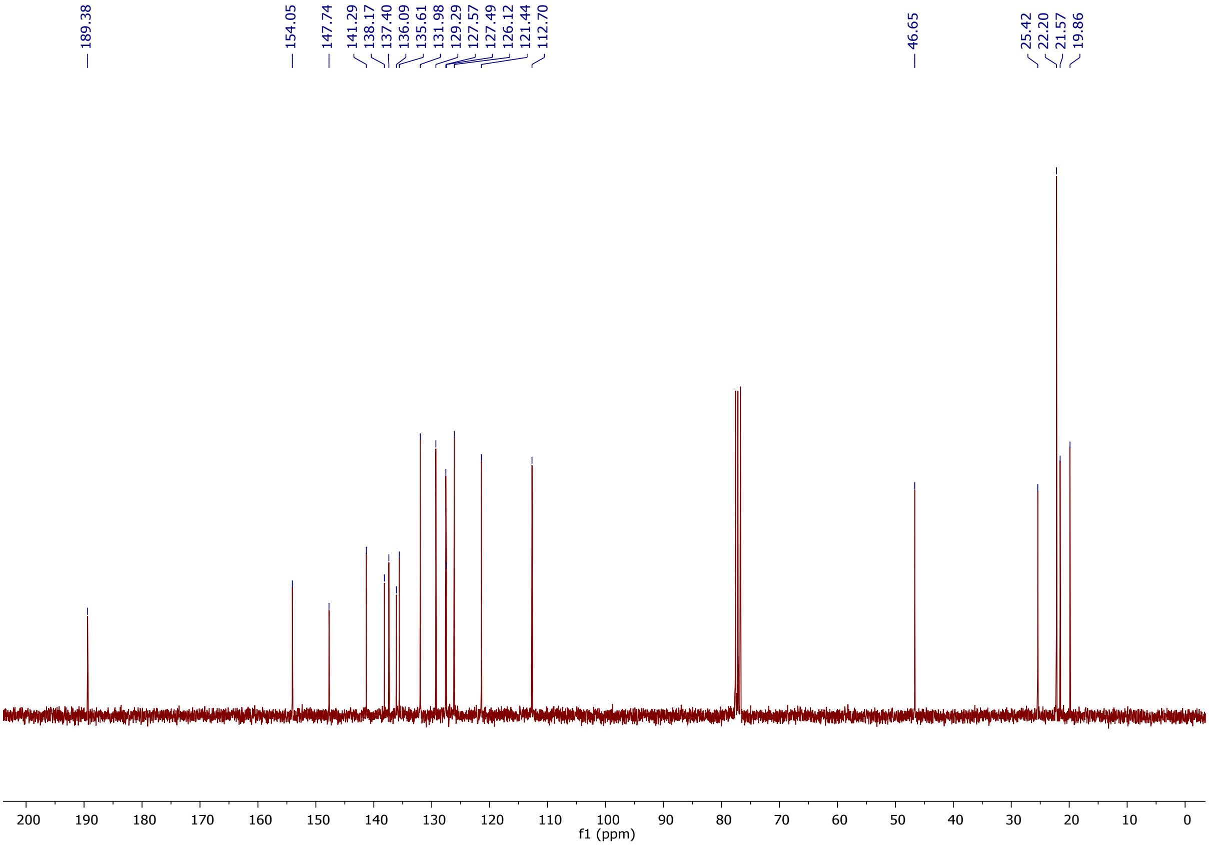
189.38
154.05
147.74
141.29 138.17 137.40 136.09 135.61 131.98 129.29 127.57 127.49 126.12 121.44 112.70
46.65
25.42 22.20 21.57 19.86
200 190 180 170 160 150 140 130 120 110 100 90 80 70 60 50 40 30 20 10 0
f1 (ppm)

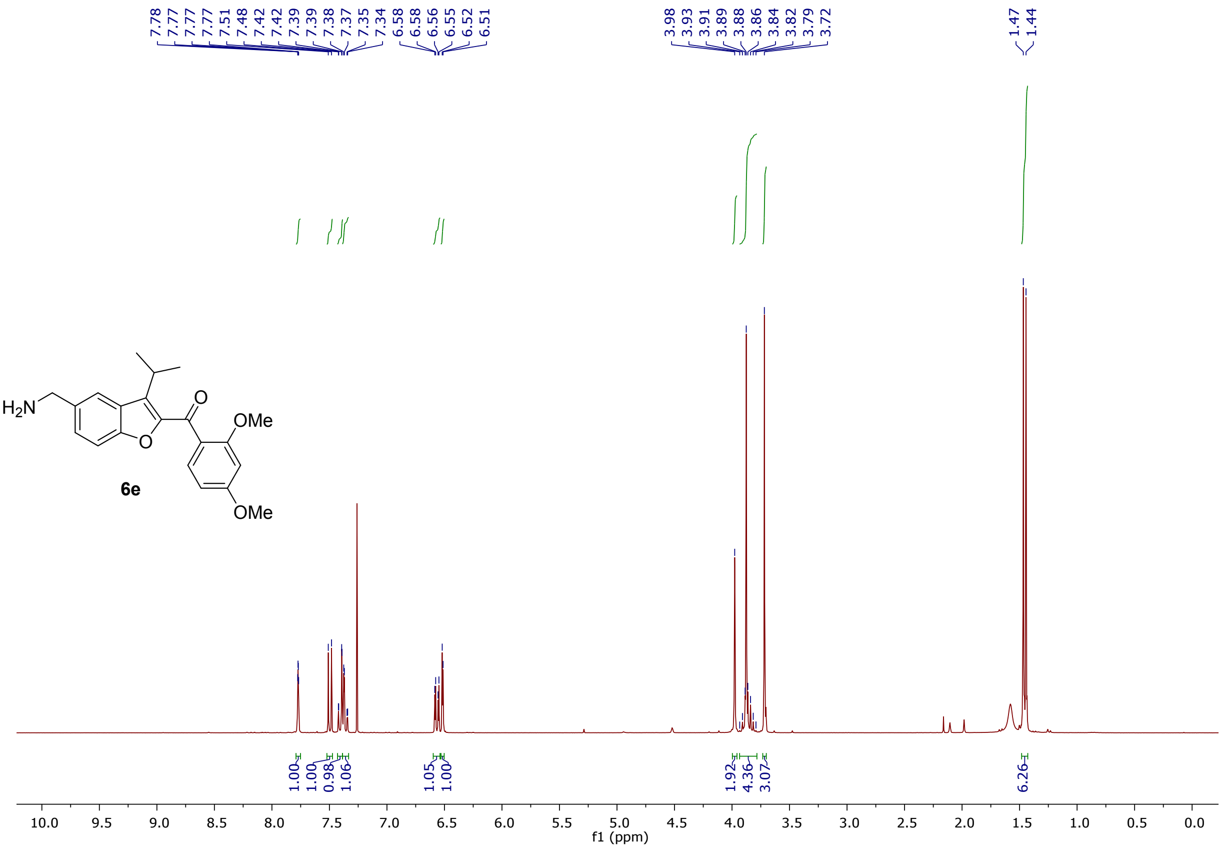
7.78
7.77
7.77
7.77
7.51
7.48
7.42
7.42
7.39
7.39
7.38
7.37
7.35
7.34
6.58
6.58
6.56
6.55
6.52
6.51
3.98
3.93
3.91
3.89
3.88
3.86
3.84
3.82
3.79
3.72
1.47
1.44
H2N
O
OMe
O
OMe
6e
1.00
1.00
0.98
1.06
1.05
1.00
1.92
4.36
3.07
6.26
10.0
9.5
9.0
8.5
8.0
7.5
7.0
6.5
6.0
5.5
5.0
4.5
4.0
3.5
3.0
2.5
2.0
1.5
1.0
0.5
0.0
f1 (ppm)

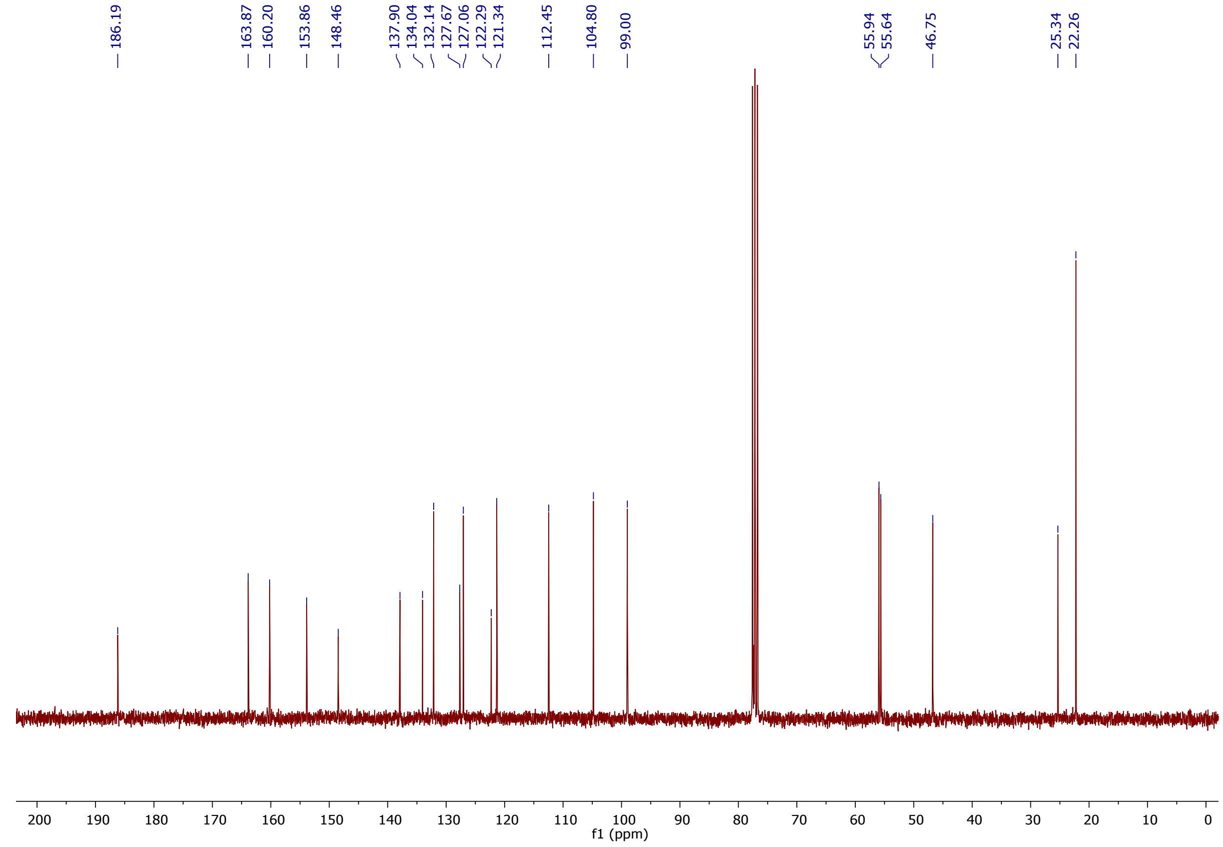
186.19
163.87
160.20
153.86
148.46
137.90
134.04
132.14
127.67
127.06
122.29
121.34
112.45
104.80
99.00
55.94
55.64
46.75
25.34
22.26
200
190
180
170
160
150
140
130
120
110
100
90
80
70
60
50
40
30
20
10
0
f1 (ppm)

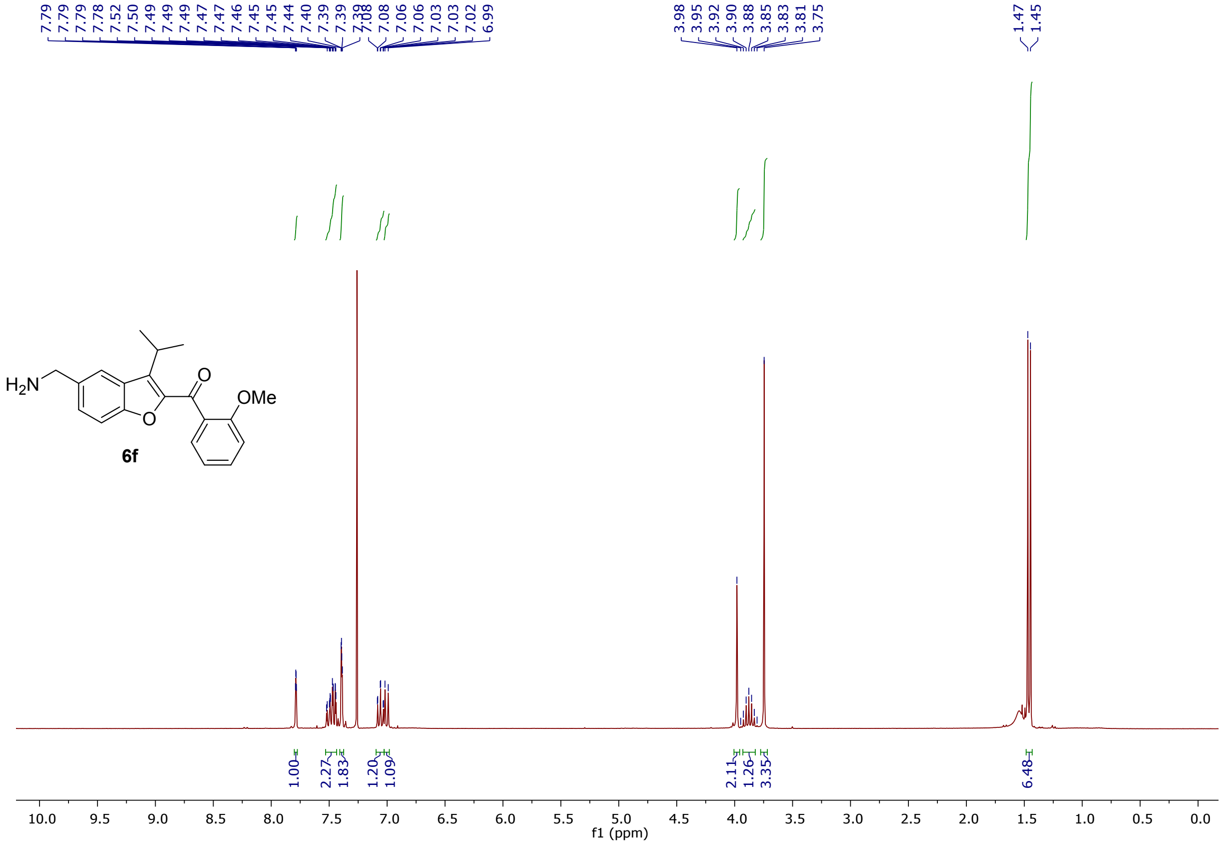
7.79 7.79 7.79 7.78 7.52 7.50 7.49 7.49 7.49 7.47 7.47 7.46 7.45 7.45 7.44 7.40 7.39 7.39 7.39 7.08 7.06 7.06 7.03 7.03 7.02 6.99
3.98 3.95 3.92 3.90 3.88 3.85 3.83 3.81 3.75
1.47 1.45
H_2N
O
OMe
O
6f
1.00 2.27 1.83 1.20 1.09
2.11 1.26 3.35
6.48
10.0 9.5 9.0 8.5 8.0 7.5 7.0 6.5 6.0 5.5 5.0 4.5 4.0 3.5 3.0 2.5 2.0 1.5 1.0 0.5 0.0
f1 (ppm)

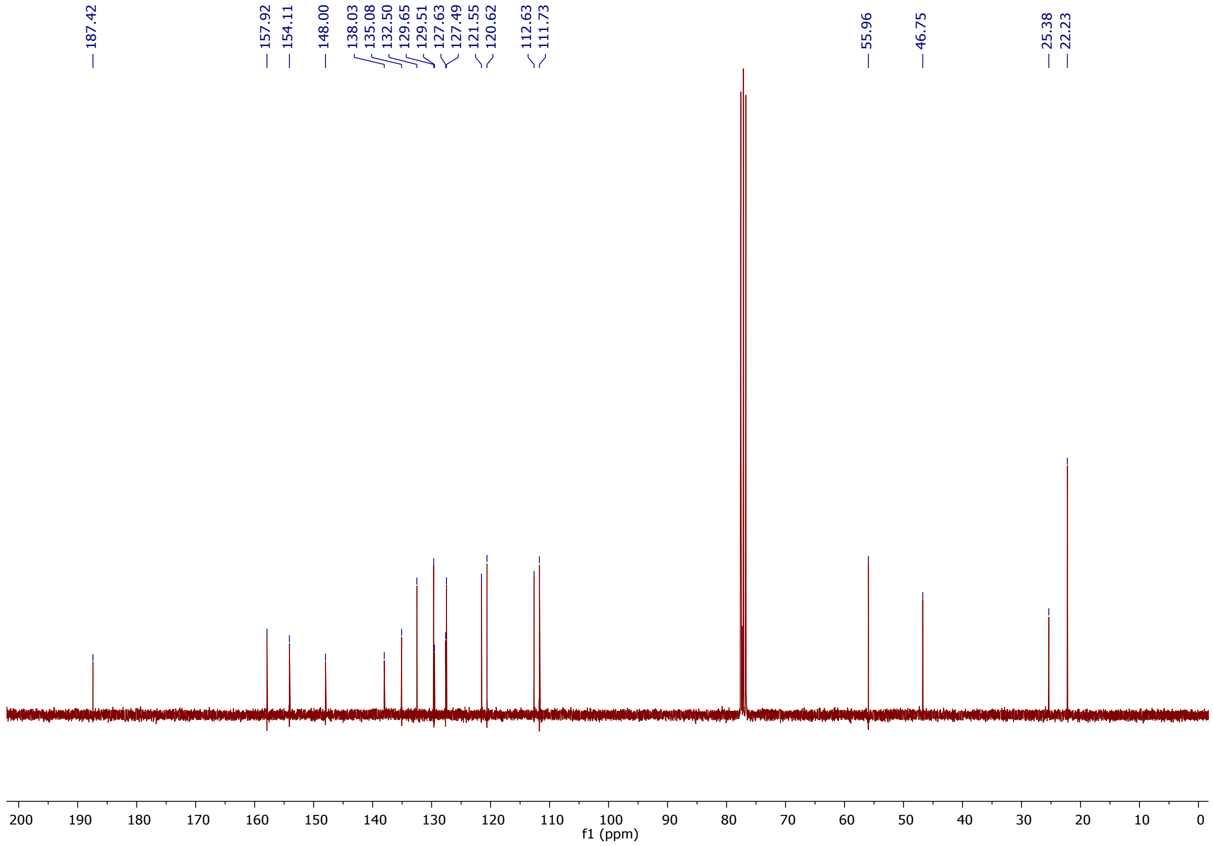
187.42
157.92 154.11 148.00 138.03 135.08 132.50 129.65 129.51 127.63 127.49 121.55 120.62 112.63 111.73
55.96 46.75 25.38 22.23
200 190 180 170 160 150 140 130 120 110 100 90 80 70 60 50 40 30 20 10 0
f1 (ppm)

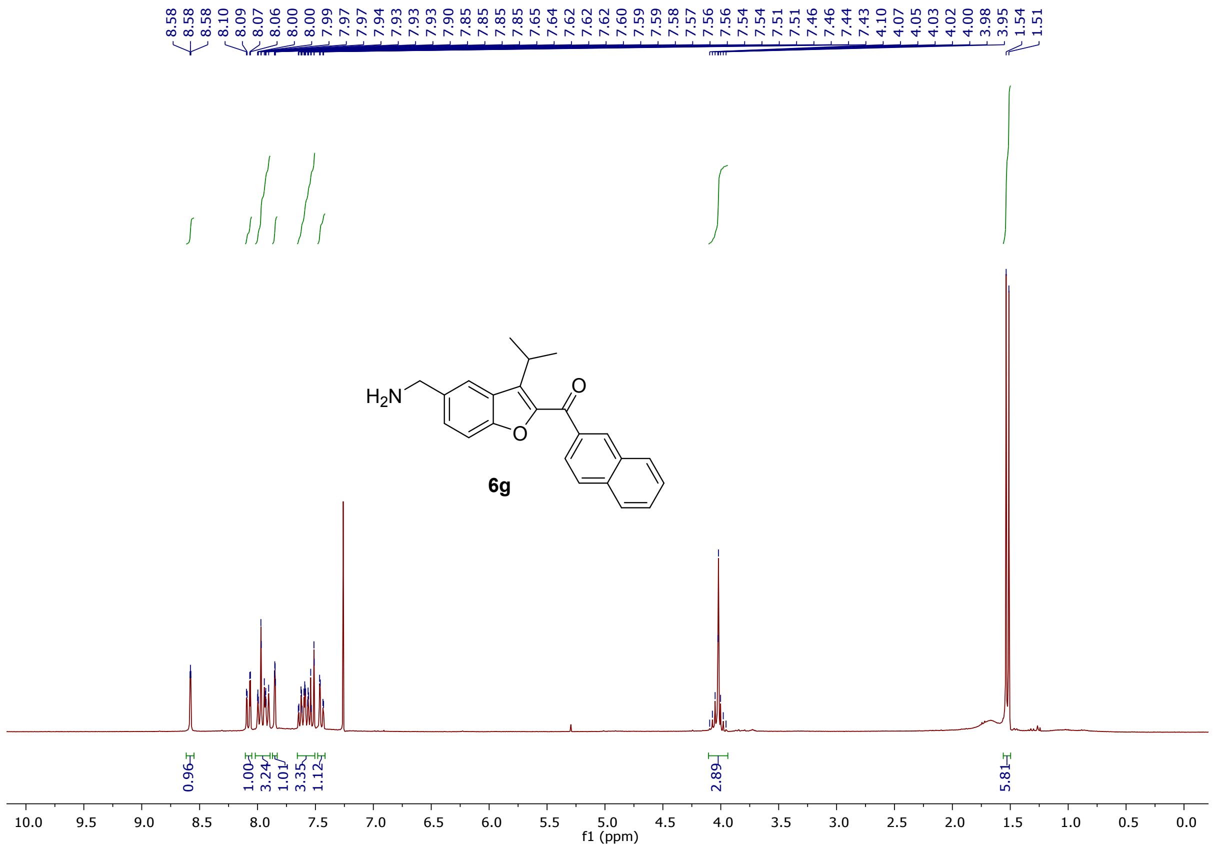
8.58 8.58 8.58 8.10 8.09 8.07 8.06 8.00 8.00 7.99 7.97 7.94 7.93 7.93 7.93 7.90 7.85 7.85 7.85 7.65 7.64 7.62 7.62 7.60 7.59 7.59 7.58 7.57 7.56 7.56 7.54 7.54 7.51 7.51 7.46 7.46 7.44 7.43 4.10 4.07 4.05 4.03 4.02 4.00 3.98 3.95 1.54 1.51
H2N
O
O
6g
0.96 1.00 3.24 1.01 3.35 1.12 2.89 5.81
10.0 9.5 9.0 8.5 8.0 7.5 7.0 6.5 6.0 5.5 5.0 4.5 4.0 3.5 3.0 2.5 2.0 1.5 1.0 0.5 0.0
f1 (ppm)

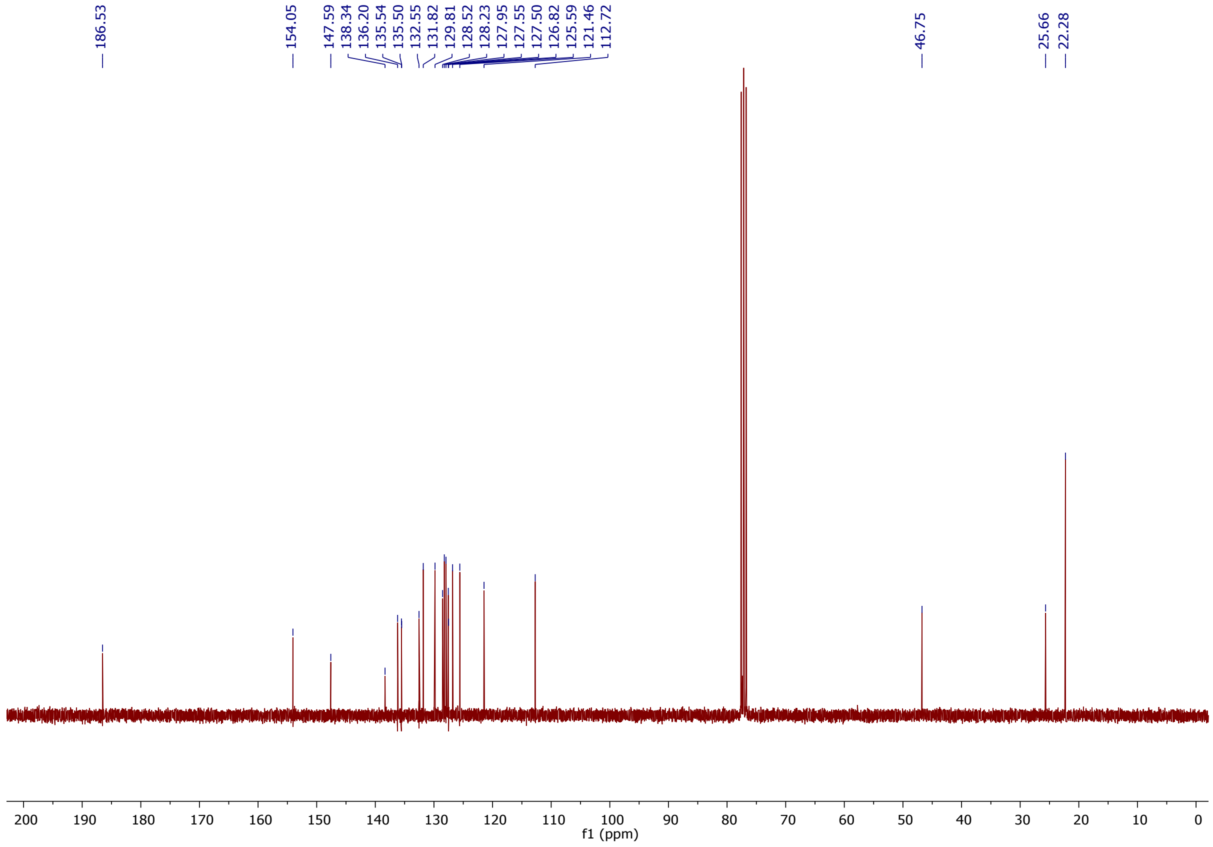
186.53
154.05
147.59 138.34 136.20 135.54 135.50 132.55 131.82 129.81 128.52 128.23 127.95 127.55 127.50 126.82 125.59 121.46 112.72
46.75
25.66 22.28
200 190 180 170 160 150 140 130 120 110 100 90 80 70 60 50 40 30 20 10 0
f1 (ppm)

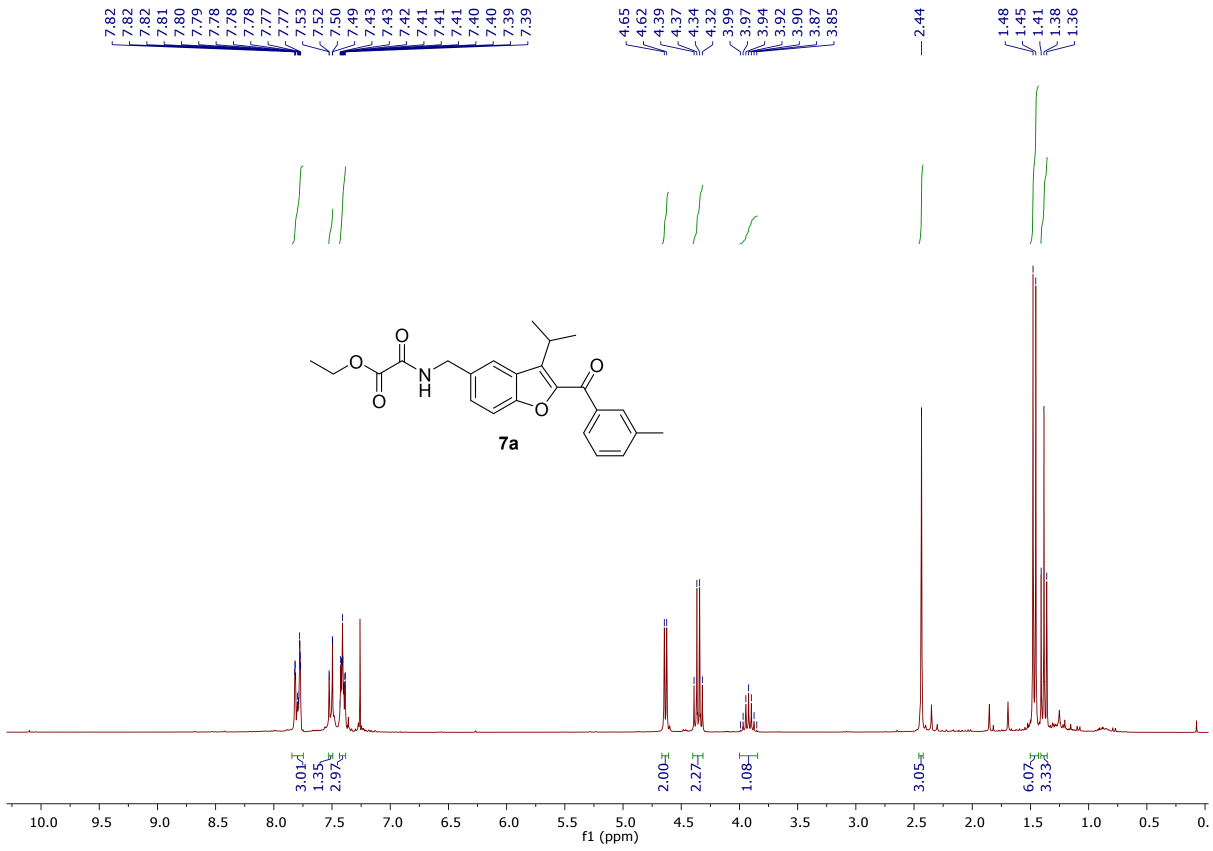
7.82 7.82 7.82 7.81 7.80 7.79 7.78 7.78 7.78 7.77 7.77 7.53 7.52 7.50 7.49 7.43 7.43 7.42 7.41 7.41 7.41 7.40 7.39 7.39
4.65 4.62 4.39 4.37 4.34 4.32 3.99 3.97 3.94 3.92 3.90 3.87 3.85
2.44
1.48 1.45 1.41 1.38 1.36
7a
3.01 1.35 2.97 2.00 2.27 1.08 3.05 6.07 3.33
10.0 9.5 9.0 8.5 8.0 7.5 7.0 6.5 6.0 5.5 5.0 4.5 4.0 3.5 3.0 2.5 2.0 1.5 1.0 0.5 0.
f1 (ppm)

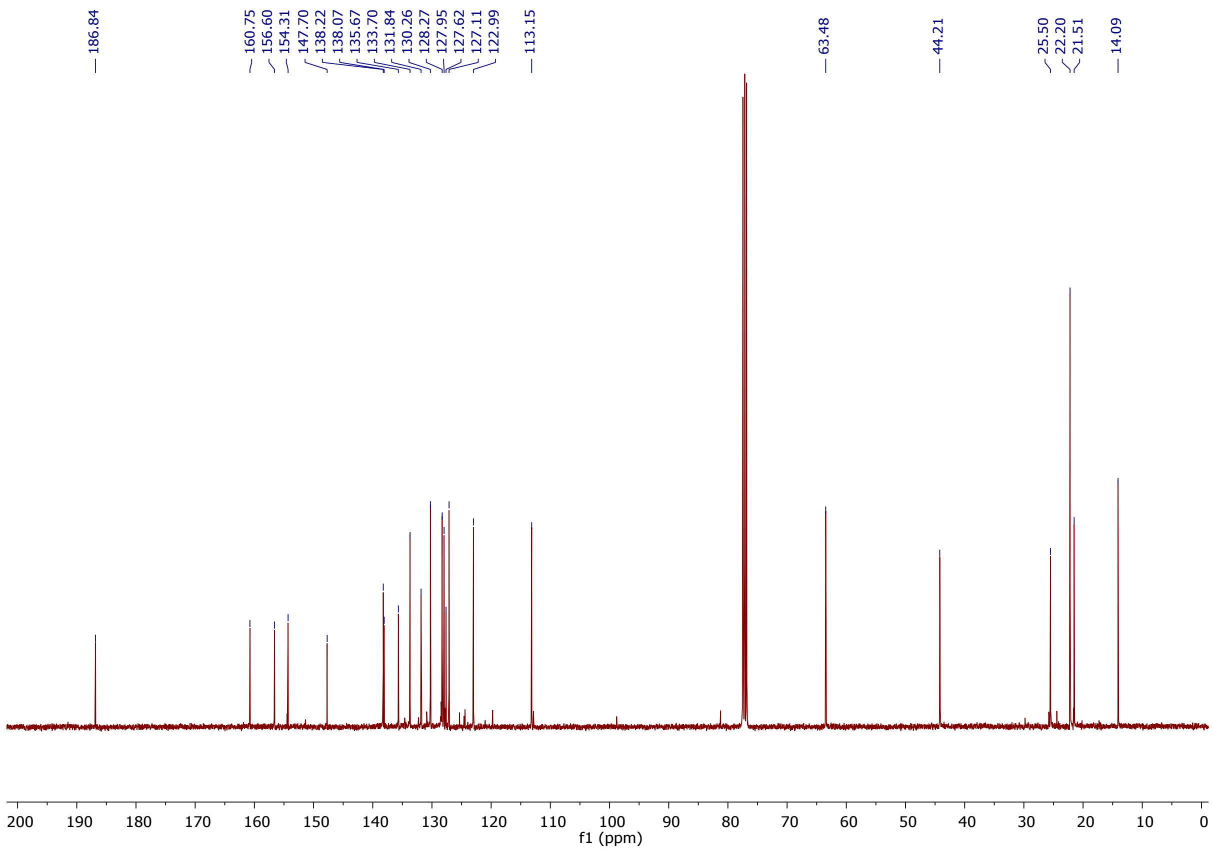
186.84
160.75 156.60 154.31 147.70 138.22 138.07 135.67 133.70 131.84 130.26 128.27 127.95 127.62 127.11 122.99
113.15
63.48
44.21
25.50 22.20 21.51
14.09
200 190 180 170 160 150 140 130 120 110 100 90 80 70 60 50 40 30 20 10 0
f1 (ppm)

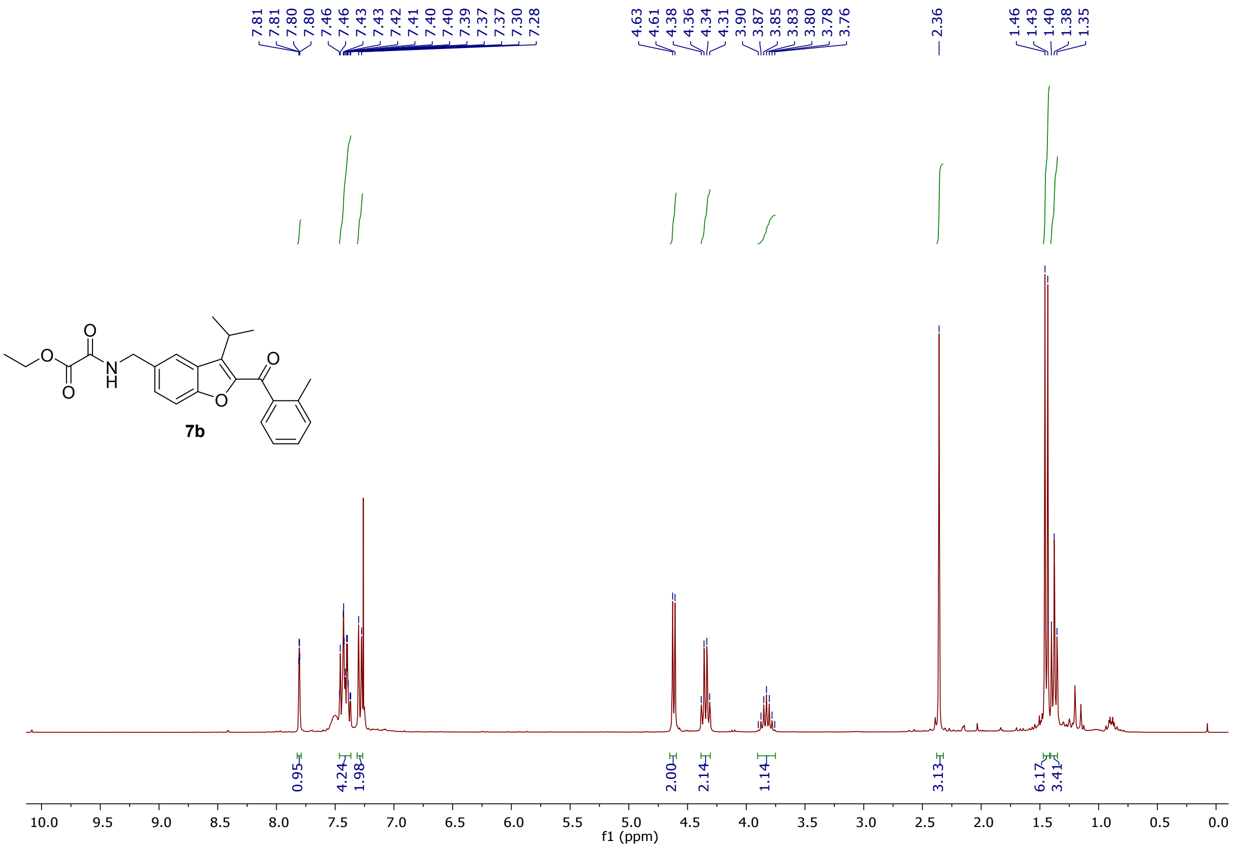

7b
7.81 7.81 7.80 7.80 7.46 7.46 7.43 7.43 7.42 7.41 7.40 7.40 7.39 7.37 7.37 7.30 7.28
4.63 4.61 4.38 4.36 4.34 4.31 3.90 3.87 3.85 3.83 3.80 3.78 3.76
2.36
1.46 1.43 1.40 1.38 1.35
0.95 4.24 1.98 2.00 2.14 1.14 3.13 6.17 3.41
10.0 9.5 9.0 8.5 8.0 7.5 7.0 6.5 6.0 5.5 5.0 4.5 4.0 3.5 3.0 2.5 2.0 1.5 1.0 0.5 0.0
f1 (ppm)

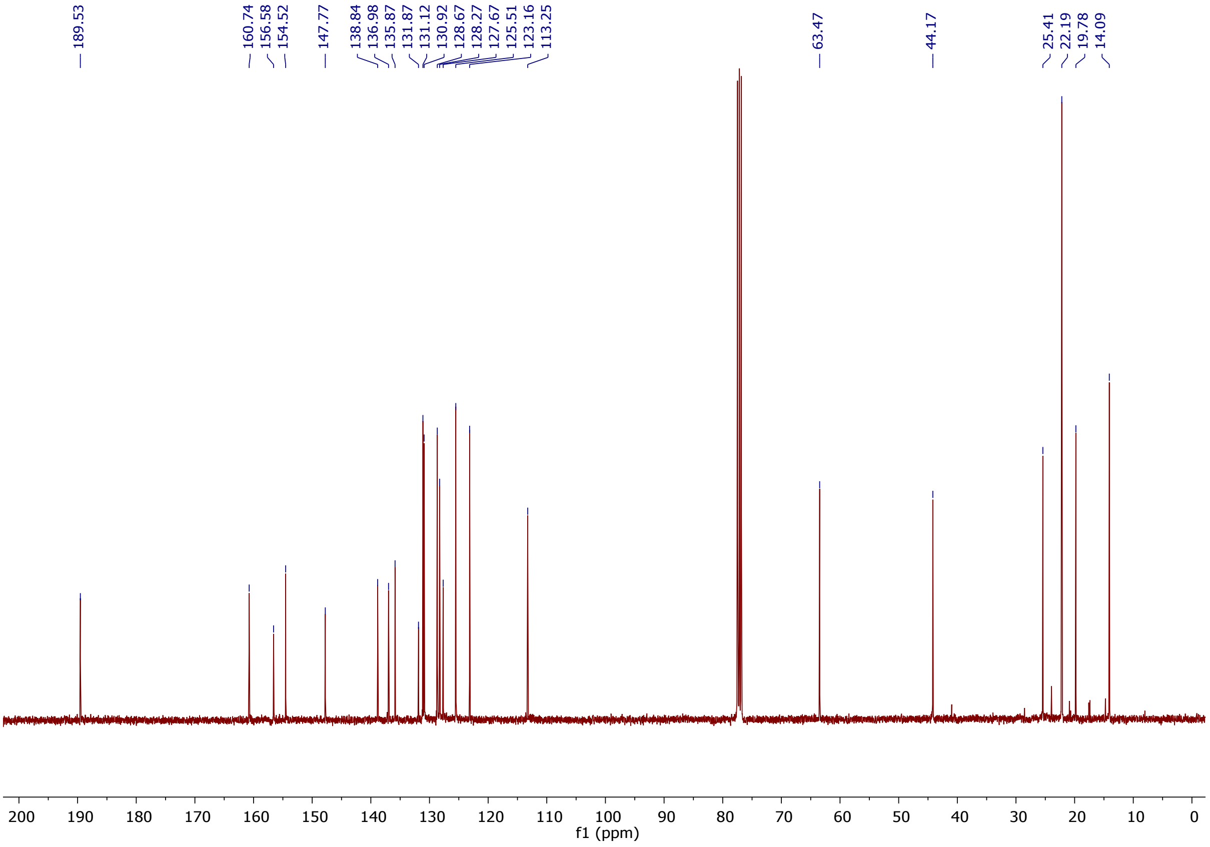
189.53
160.74 156.58 154.52
147.77
138.84 136.98 135.87 131.87 131.12 130.92 128.67 128.27 127.67 125.51 123.16 113.25
63.47
44.17
25.41 22.19 19.78 14.09
200 190 180 170 160 150 140 130 120 110 100 90 80 70 60 50 40 30 20 10 0
f1 (ppm)

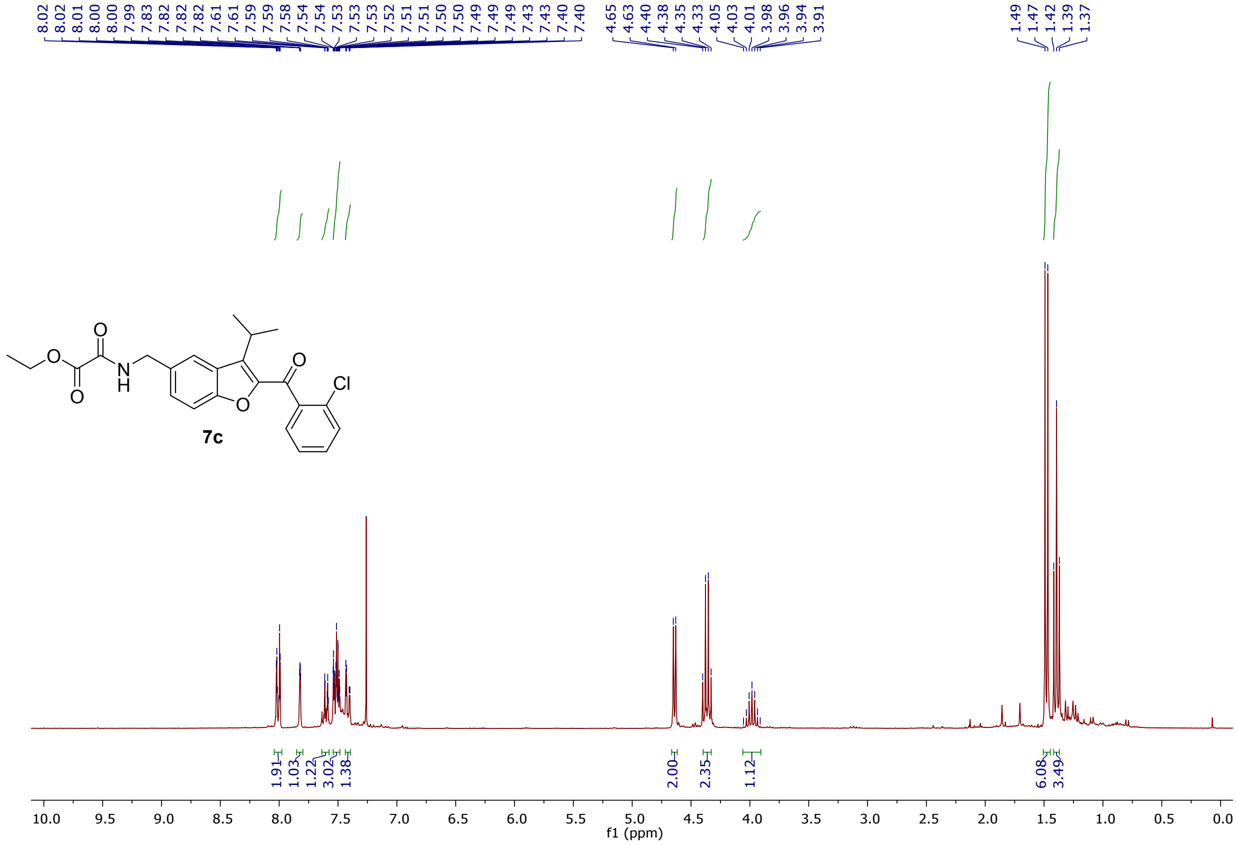

7c

8.02 8.02 8.01 8.00 8.00 7.99 7.83 7.82 7.82 7.82 7.61 7.59 7.59 7.58 7.54 7.54 7.53 7.53 7.53 7.52 7.51 7.51 7.50 7.50 7.49 7.49 7.49 7.43 7.40 7.40
4.65 4.63 4.40 4.38 4.35 4.33 4.05 4.03 4.01 3.98 3.96 3.94 3.91
1.49 1.47 1.42 1.39 1.37
1.91 1.03 1.22 3.02 1.38 2.00 2.35 1.12 6.08 3.49
10.0 9.5 9.0 8.5 8.0 7.5 7.0 6.5 6.0 5.5 5.0 4.5 4.0 3.5 3.0 2.5 2.0 1.5 1.0 0.5 0.0
f1 (ppm)

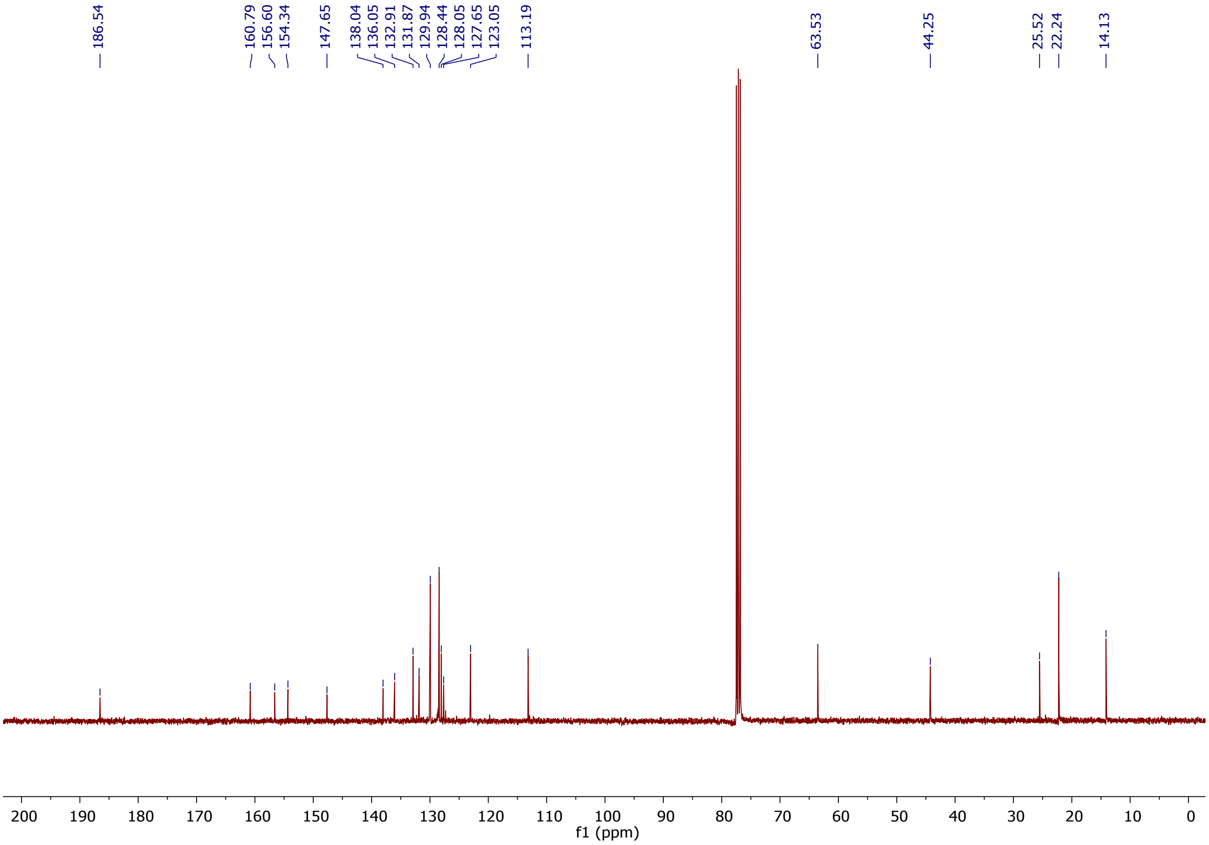
186.54
160.79 156.60 154.34
147.65
138.04 136.05 132.91 131.87 129.94 128.44 128.05 127.65 123.05
113.19
63.53
44.25
25.52 22.24
14.13
200 190 180 170 160 150 140 130 120 110 100 90 80 70 60 50 40 30 20 10 0
f1 (ppm)

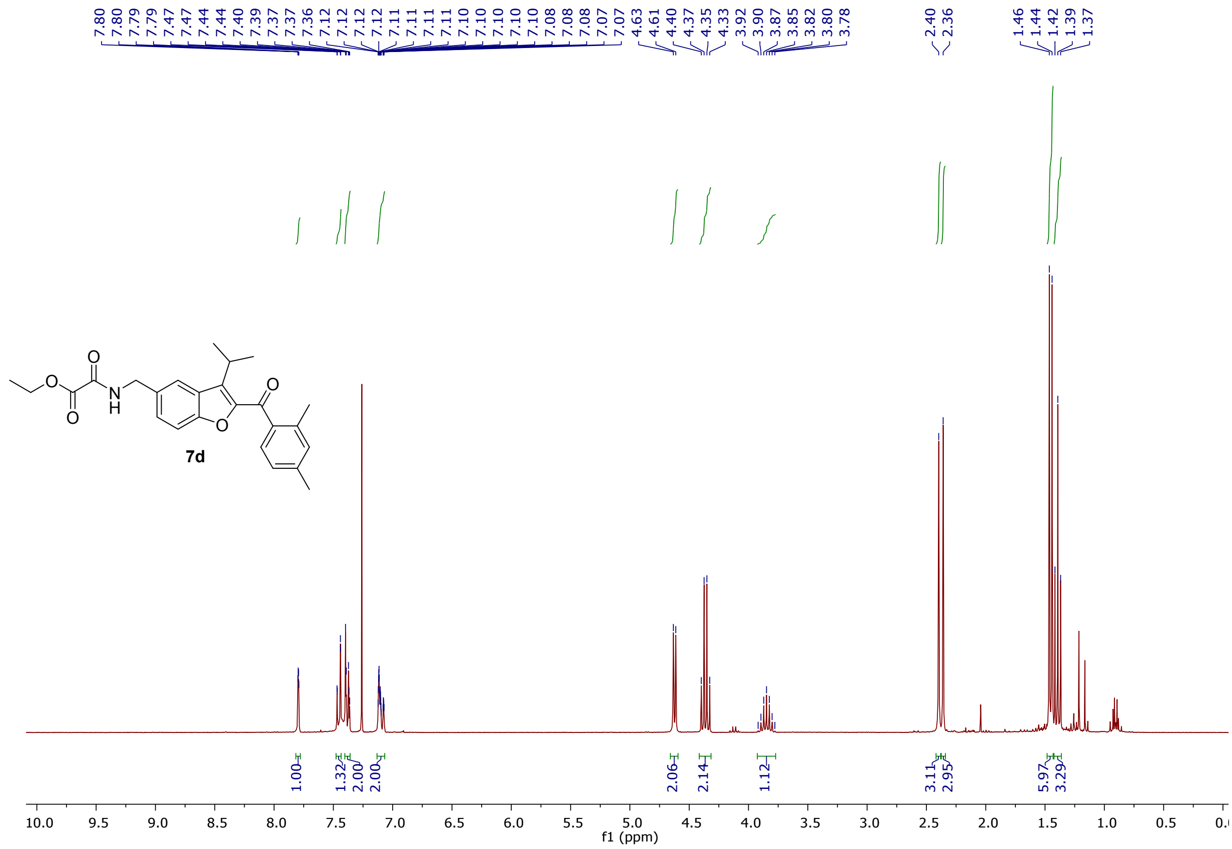
7.80 7.80 7.79 7.79 7.47 7.47 7.44 7.44 7.40 7.39 7.37 7.37 7.36 7.12 7.12 7.12 7.12 7.11 7.11 7.11 7.11 7.10 7.10 7.10 7.10 7.10 7.08 7.08 7.07 7.07 4.63 4.61 4.40 4.37 4.35 4.33 3.92 3.90 3.87 3.85 3.82 3.80 3.78
2.40 2.36
1.46 1.44 1.42 1.39 1.37
1.00 1.32 2.00 2.00 2.06 2.14 1.12 3.11 2.95 5.97 3.29
f1 (ppm)

7d

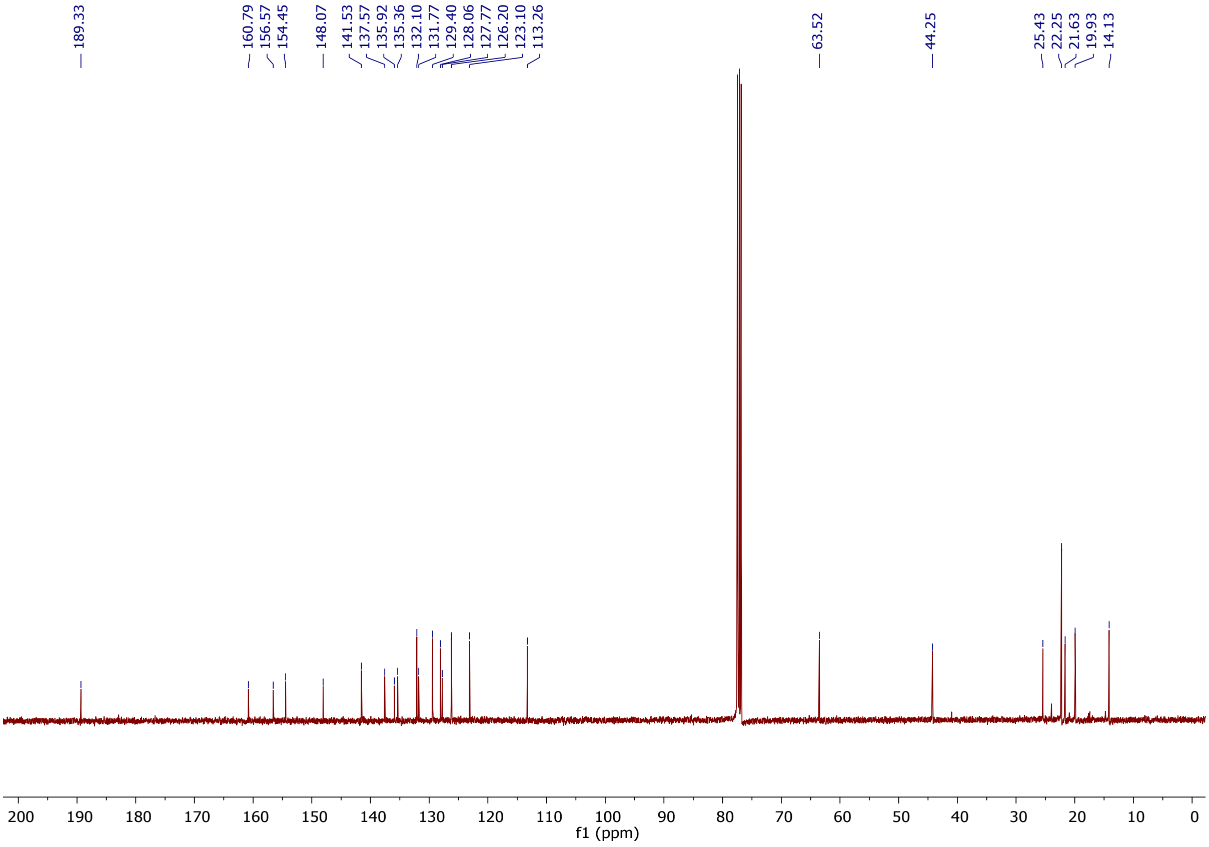
189.33
160.79 156.57 154.45
148.07
141.53 137.57 135.92 135.36 132.10 131.77 129.40 128.06 127.77 126.20 123.10 113.26
63.52
44.25
25.43 22.25 21.63 19.93 14.13
f1 (ppm)

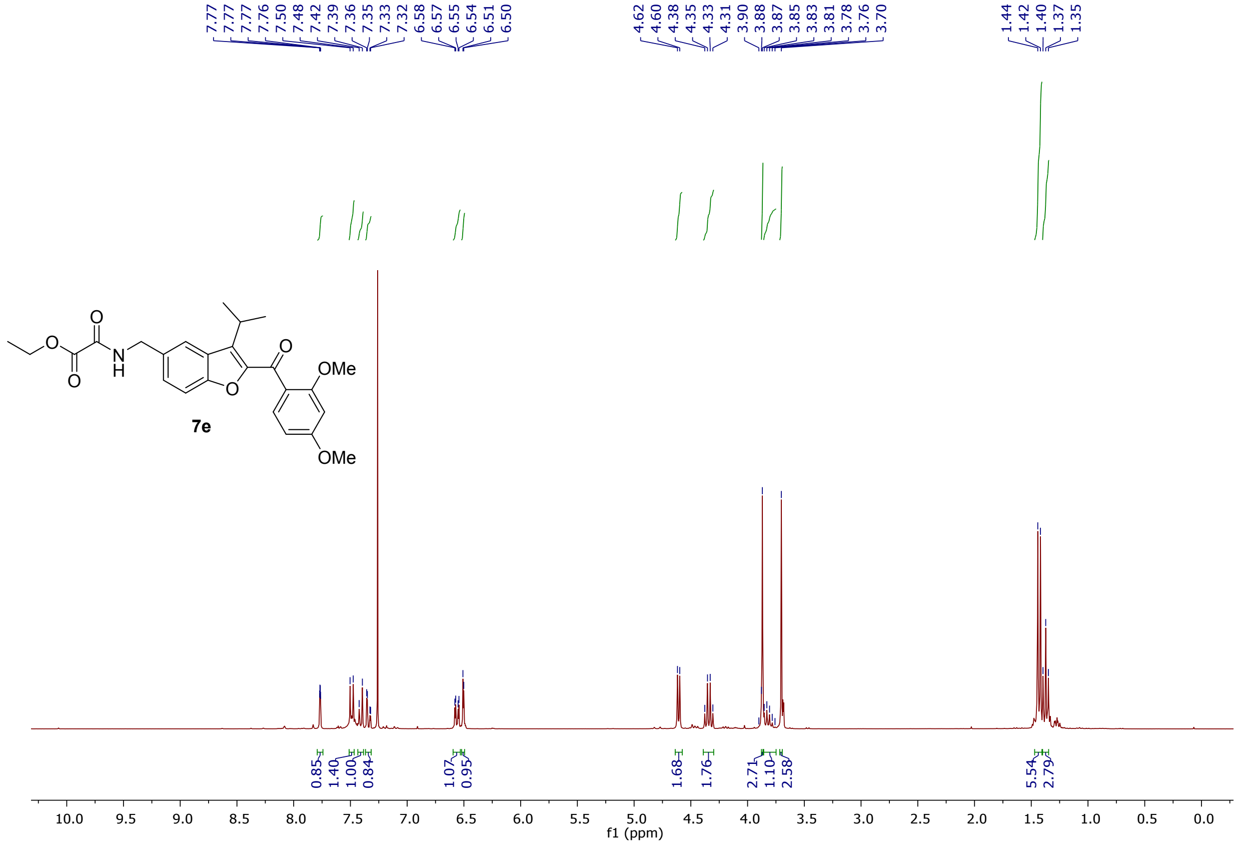
7.77
7.77
7.77
7.76
7.50
7.48
7.42
7.39
7.36
7.35
7.33
7.32
6.58
6.57
6.55
6.54
6.51
6.50
4.62
4.60
4.38
4.35
4.33
4.31
3.90
3.88
3.87
3.85
3.83
3.81
3.78
3.76
3.70
1.44
1.42
1.40
1.37
1.35
O
O
O
N
H
O
O
OMe
O
OMe
7e
0.85
1.40
1.00
0.84
1.07
0.95
1.68
1.76
2.71
1.10
2.58
5.54
2.79
10.0 9.5 9.0 8.5 8.0 7.5 7.0 6.5 6.0 5.5 5.0 4.5 4.0 3.5 3.0 2.5 2.0 1.5 1.0 0.5 0.0
f1 (ppm)

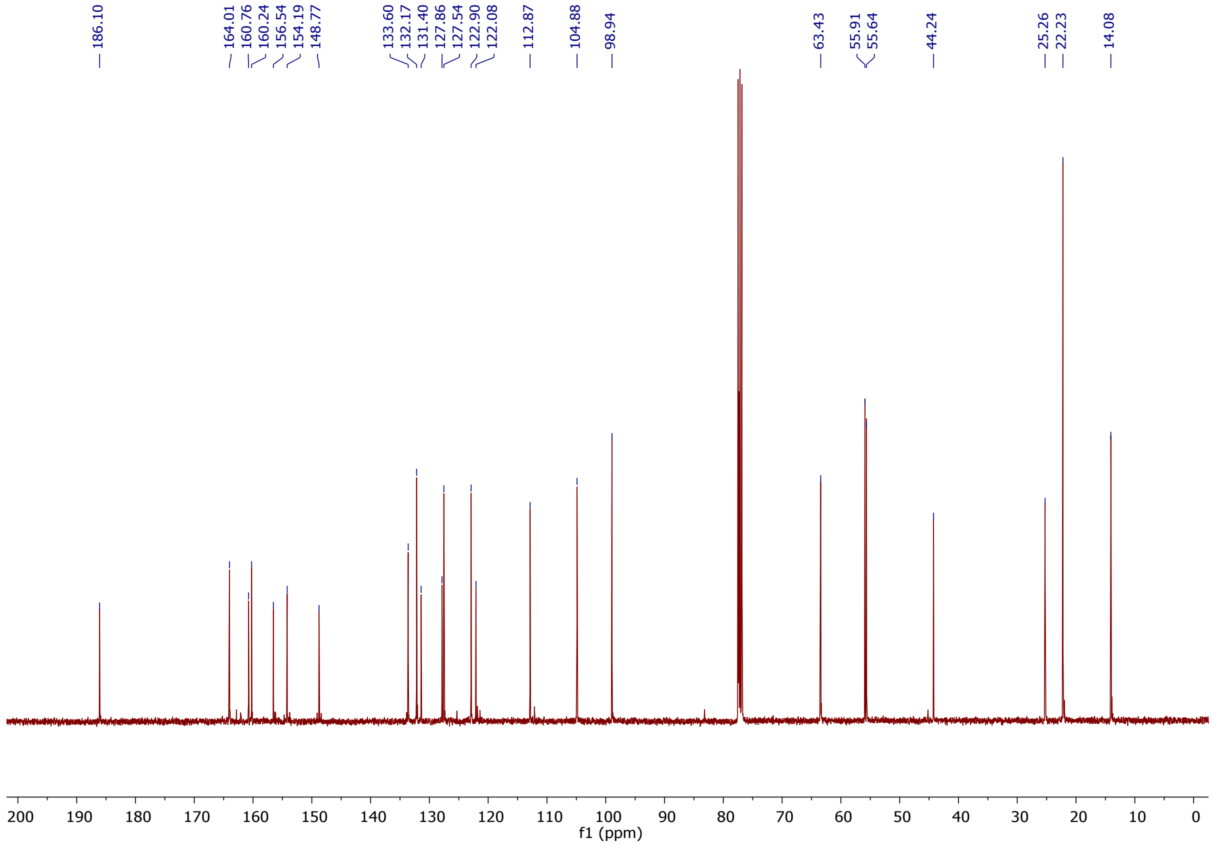
186.10
164.01
160.76
160.24
156.54
154.19
148.77
133.60
132.17
131.40
127.86
127.54
122.90
122.08
112.87
104.88
98.94
63.43
55.91
55.64
44.24
25.26
22.23
14.08
200 190 180 170 160 150 140 130 120 110 100 90 80 70 60 50 40 30 20 10 0
f1 (ppm)

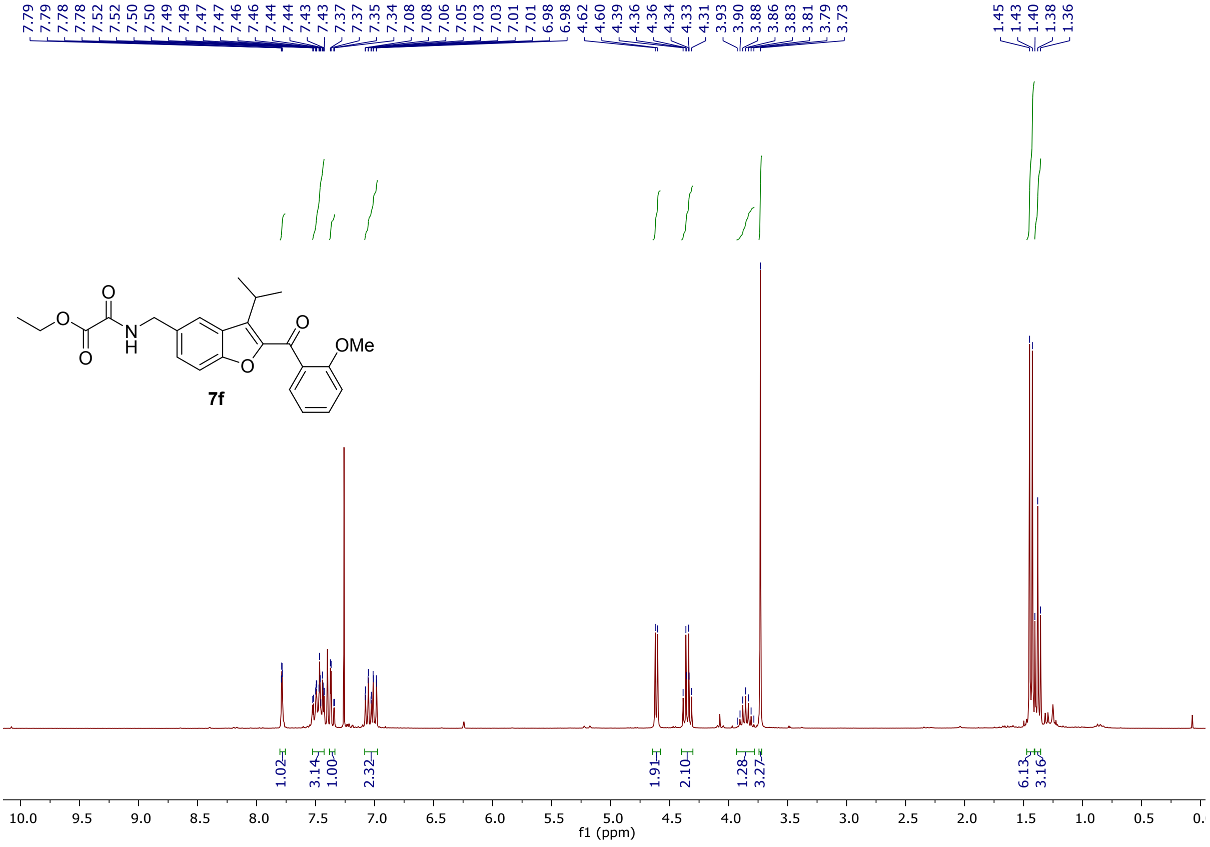
7.79 7.79 7.78 7.78 7.52 7.52 7.50 7.50 7.49 7.49 7.47 7.47 7.46 7.46 7.44 7.44 7.43 7.43 7.37 7.37 7.35 7.34 7.08 7.08 7.06 7.05 7.03 7.03 7.01 7.01 6.98 4.62 4.60 4.39 4.36 4.36 4.34 4.33 4.31 3.93 3.90 3.88 3.86 3.83 3.81 3.79 3.73
1.45 1.43 1.40 1.38 1.36
OMe
7f
1.02 3.14 1.00 2.32 1.91 2.10 1.28 3.27 6.13 3.16
f1 (ppm)

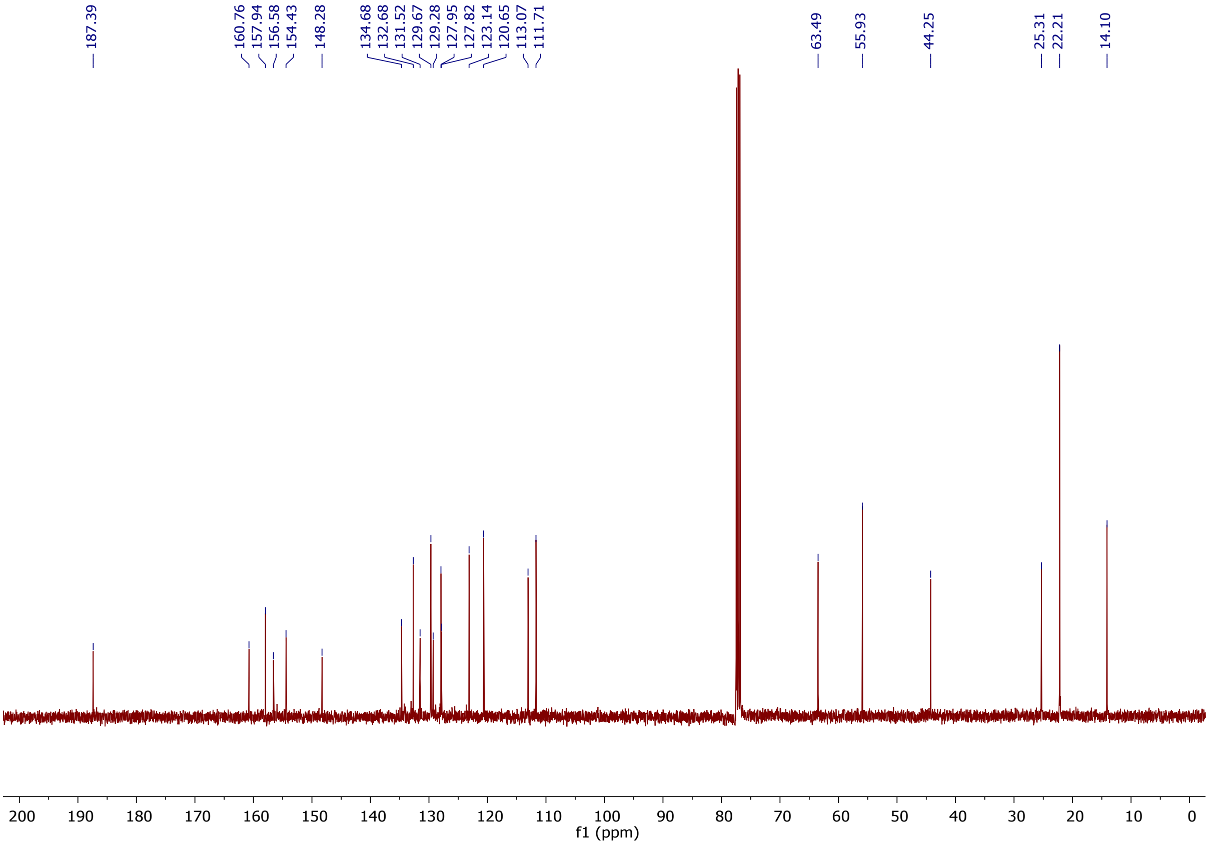
187.39
160.76 157.94 156.58 154.43 148.28
134.68 132.68 131.52 129.67 129.28 127.95 127.82 123.14 120.65 113.07 111.71
63.49 55.93 44.25 25.31 22.21 14.10
f1 (ppm)

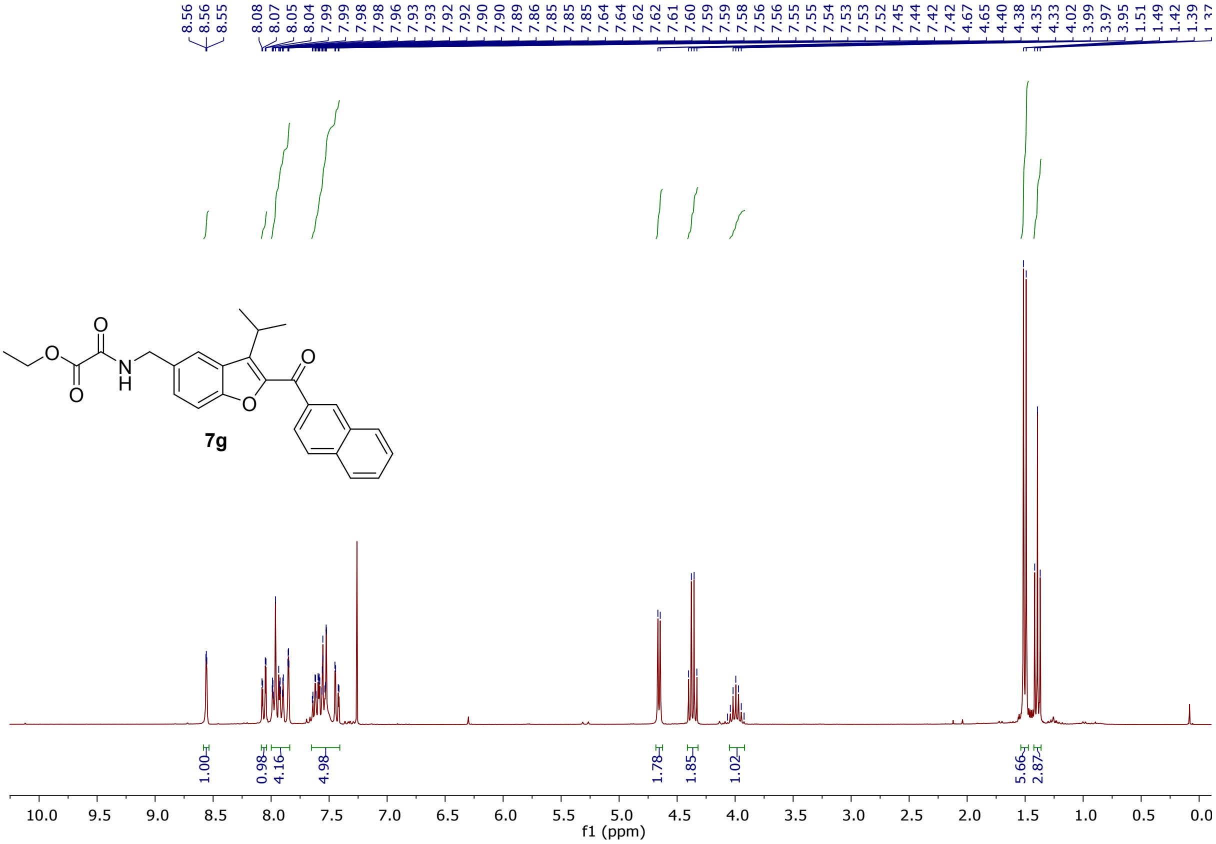

8.56 8.56 8.55 8.08 8.07 8.05 8.04 7.99 7.98 7.98 7.96 7.93 7.93 7.92 7.92 7.90 7.90 7.89 7.86 7.85 7.85 7.85 7.64 7.64 7.62 7.62 7.61 7.60 7.59 7.59 7.58 7.56 7.56 7.55 7.55 7.54 7.53 7.53 7.52 7.45 7.44 7.42 7.42 4.67 4.65 4.40 4.38 4.35 4.33 4.02 3.99 3.97 3.95 1.51 1.49 1.42 1.39 [illegible]
1.00
0.98
4.16
4.98
1.78
1.85
1.02
5.66
2.87
f1 (ppm)

7g

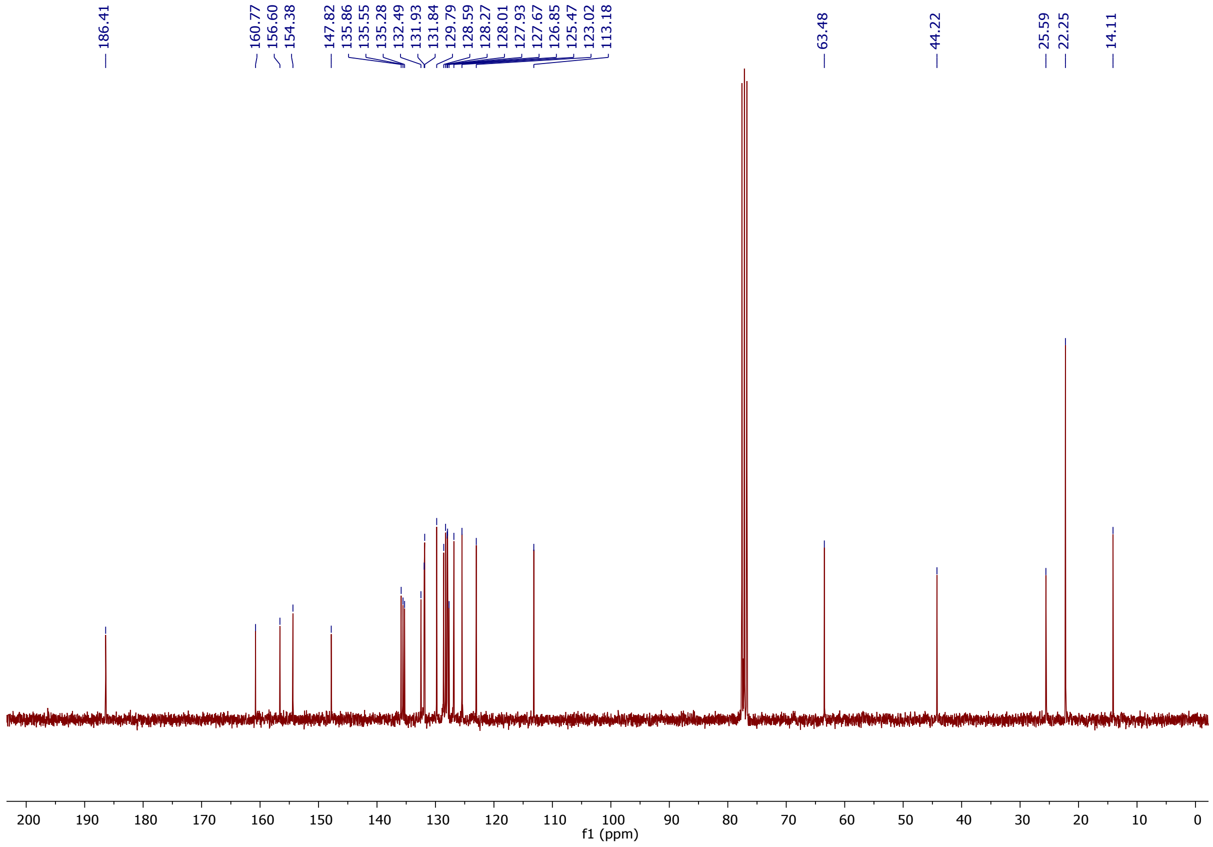

186.41
160.77 156.60 154.38
147.82 135.86 135.55 135.28 132.49 131.93 131.84 129.79 128.59 128.27 128.01 127.93 127.67 126.85 125.47 123.02 113.18
63.48
44.22
25.59 22.25
14.11
f1 (ppm)

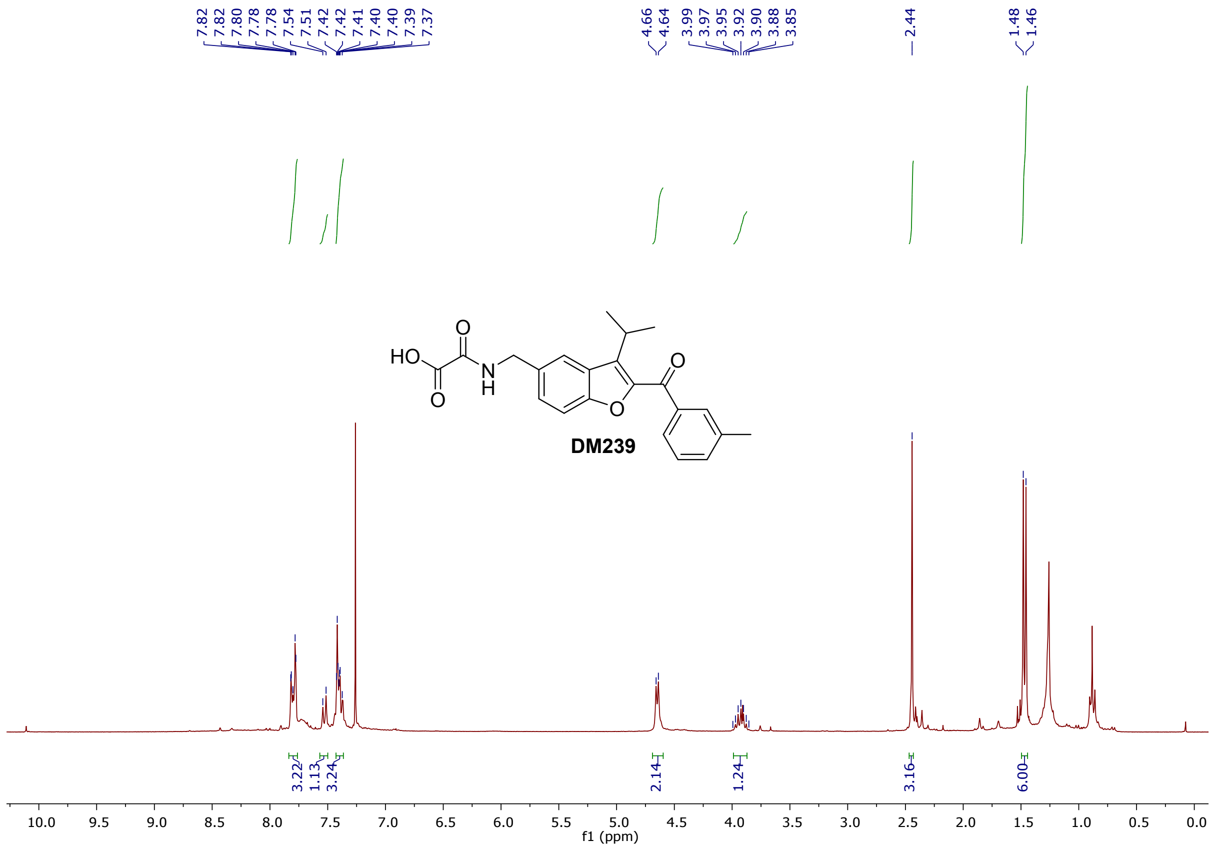
7.82
7.82
7.80
7.78
7.78
7.54
7.51
7.42
7.42
7.41
7.40
7.40
7.39
7.37
4.66
4.64
3.99
3.97
3.95
3.92
3.90
3.88
3.85
2.44
1.48
1.46
HO
O
O
N
H
O
O
DM239
3.22
1.13
3.24
2.14
1.24
3.16
6.00
10.0 9.5 9.0 8.5 8.0 7.5 7.0 6.5 6.0 5.5 5.0 4.5 4.0 3.5 3.0 2.5 2.0 1.5 1.0 0.5 0.0
f1 (ppm)

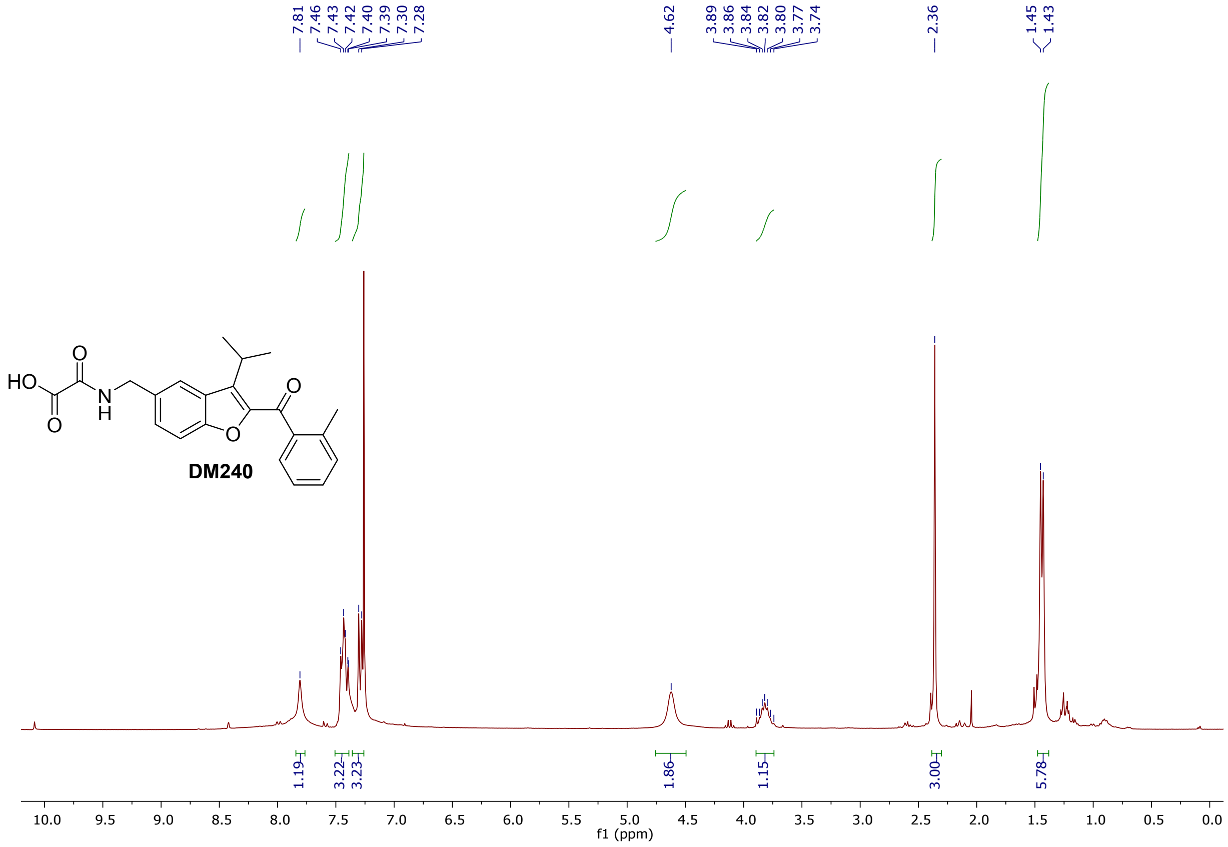
7.81
7.46
7.43
7.42
7.40
7.39
7.30
7.28
4.62
3.89
3.86
3.84
3.82
3.80
3.77
3.74
2.36
1.45
1.43
HO
O
O
N
H
O
O
DM240
1.19
3.22
3.23
1.86
1.15
3.00
5.78
10.0 9.5 9.0 8.5 8.0 7.5 7.0 6.5 6.0 5.5 5.0 4.5 4.0 3.5 3.0 2.5 2.0 1.5 1.0 0.5 0.0
f1 (ppm)

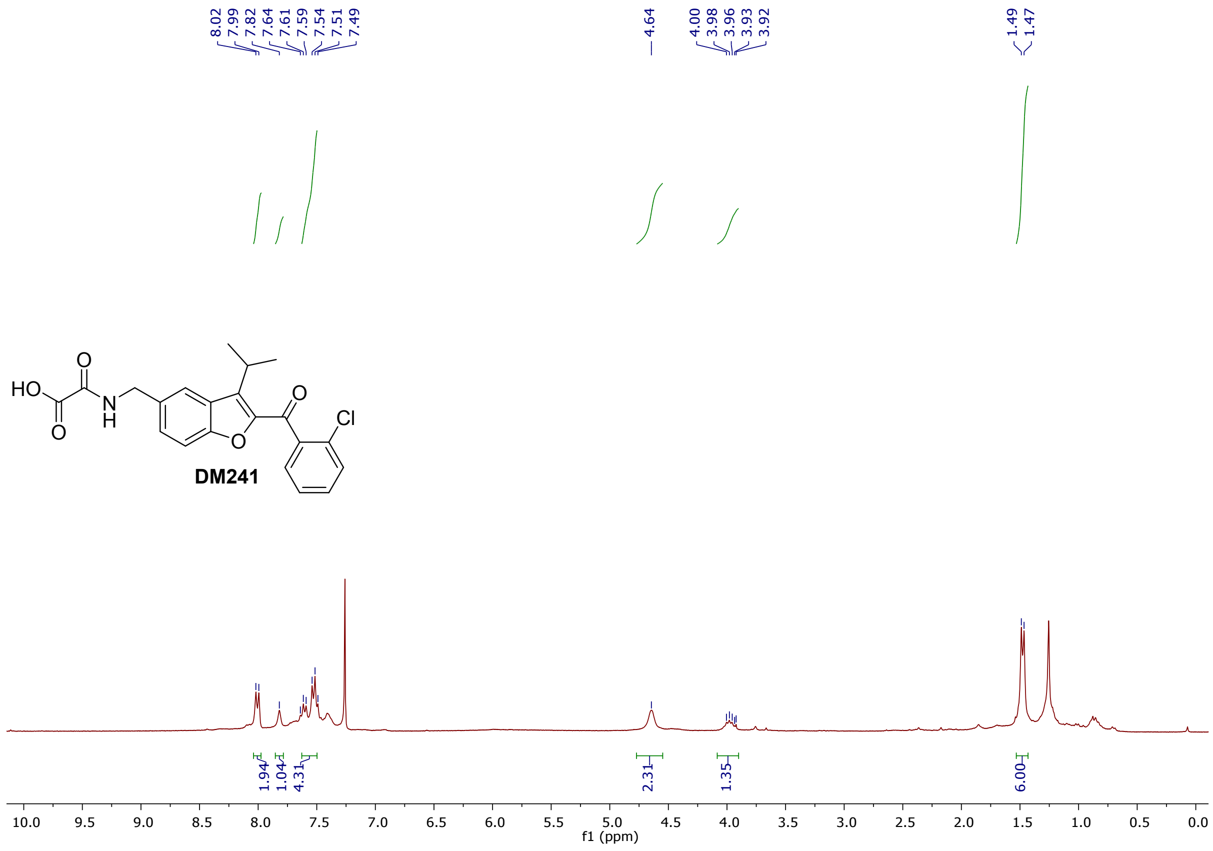
8.02
7.99
7.82
7.64
7.61
7.59
7.54
7.51
7.49
4.64
4.00
3.98
3.96
3.93
3.92
1.49
1.47
HO
O
O
N
H
O
O
Cl
DM241
1.94
1.04
4.31
2.31
1.35
6.00
10.0 9.5 9.0 8.5 8.0 7.5 7.0 6.5 6.0 5.5 5.0 4.5 4.0 3.5 3.0 2.5 2.0 1.5 1.0 0.5 0.0
f1 (ppm)

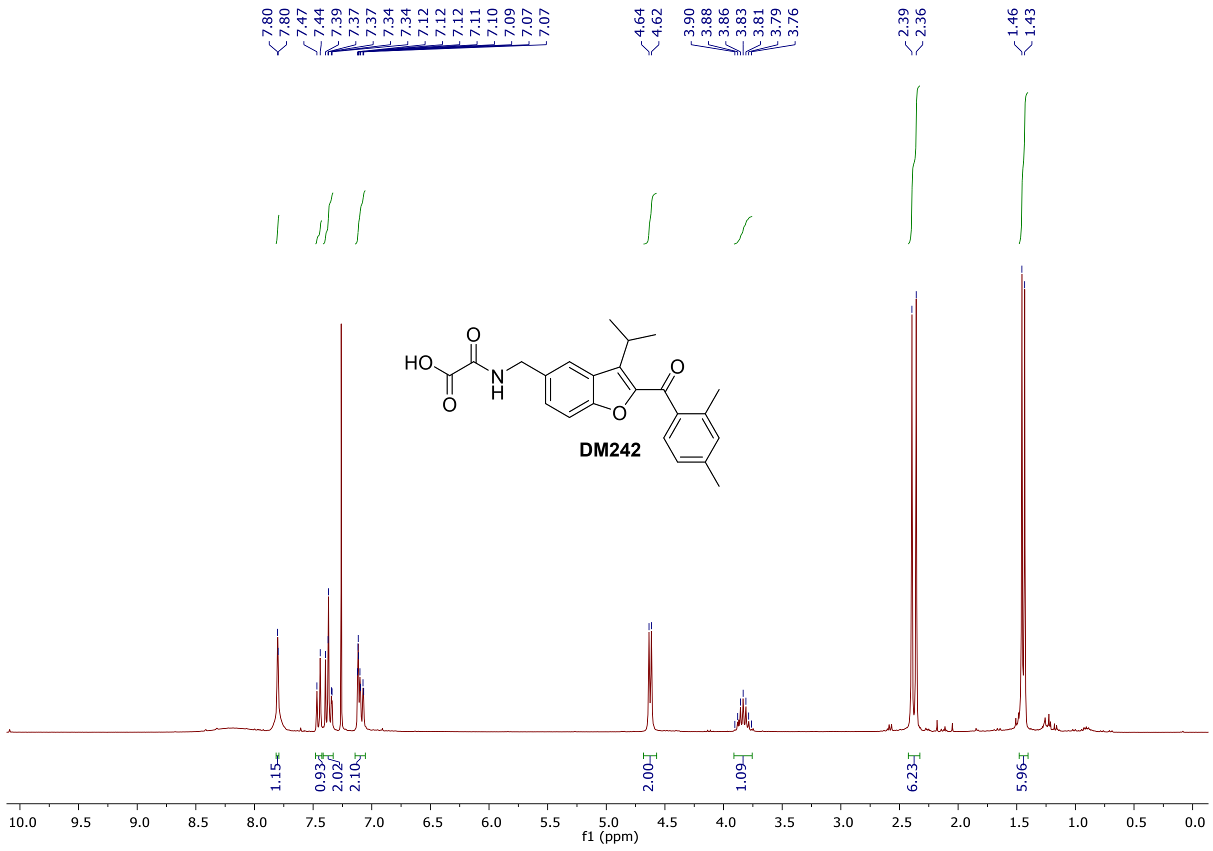
7.80
7.80
7.47
7.44
7.39
7.37
7.37
7.34
7.34
7.12
7.12
7.12
7.11
7.10
7.09
7.07
7.07
4.64
4.62
3.90
3.88
3.86
3.83
3.81
3.79
3.76
2.39
2.36
1.46
1.43
HO
O
O
N
H
O
O
DM242
1.15
0.93
2.02
2.10
2.00
1.09
6.23
5.96
f1 (ppm)

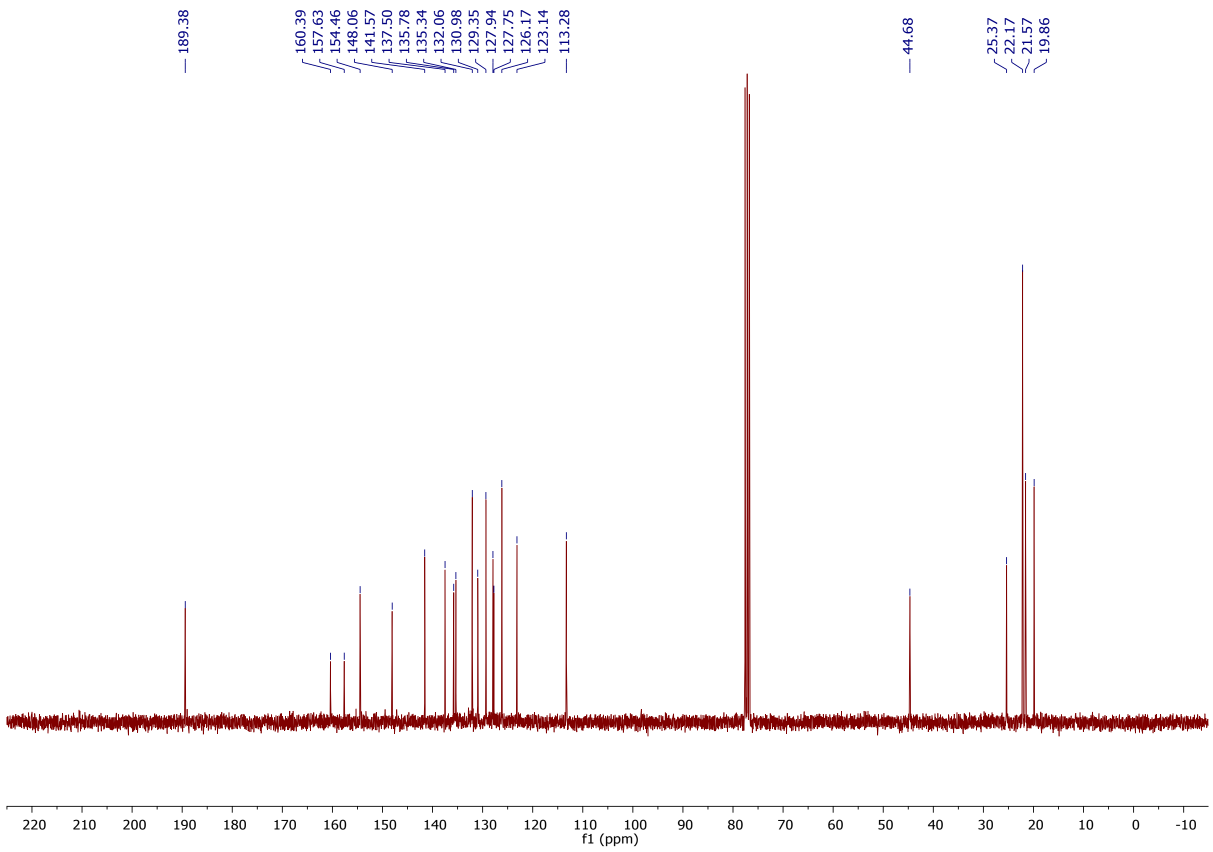
189.38
160.39
157.63
154.46
148.06
141.57
137.50
135.78
135.34
132.06
130.98
129.35
127.94
127.75
126.17
123.14
113.28
44.68
25.37
22.17
21.57
19.86
f1 (ppm)

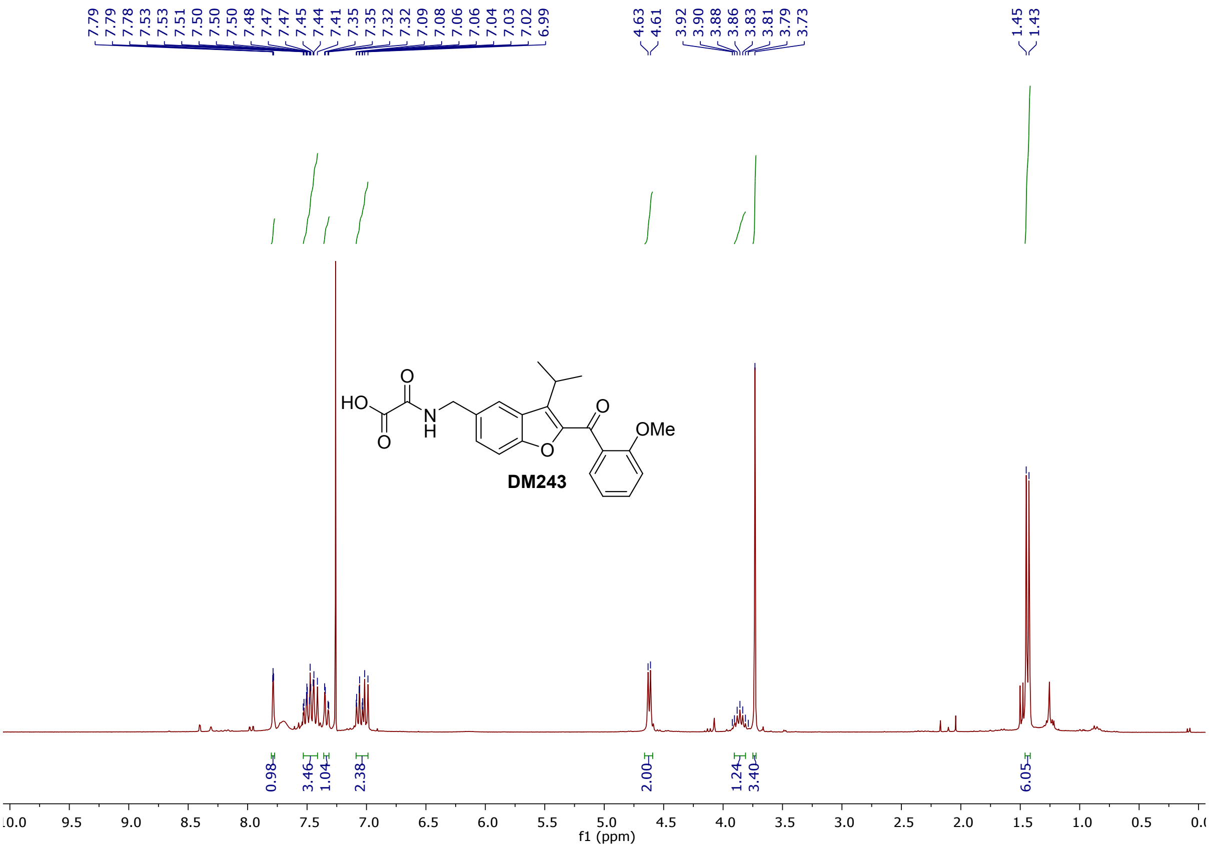

DM243
HO
O
O
N
H
O
O
OMe
f1 (ppm)

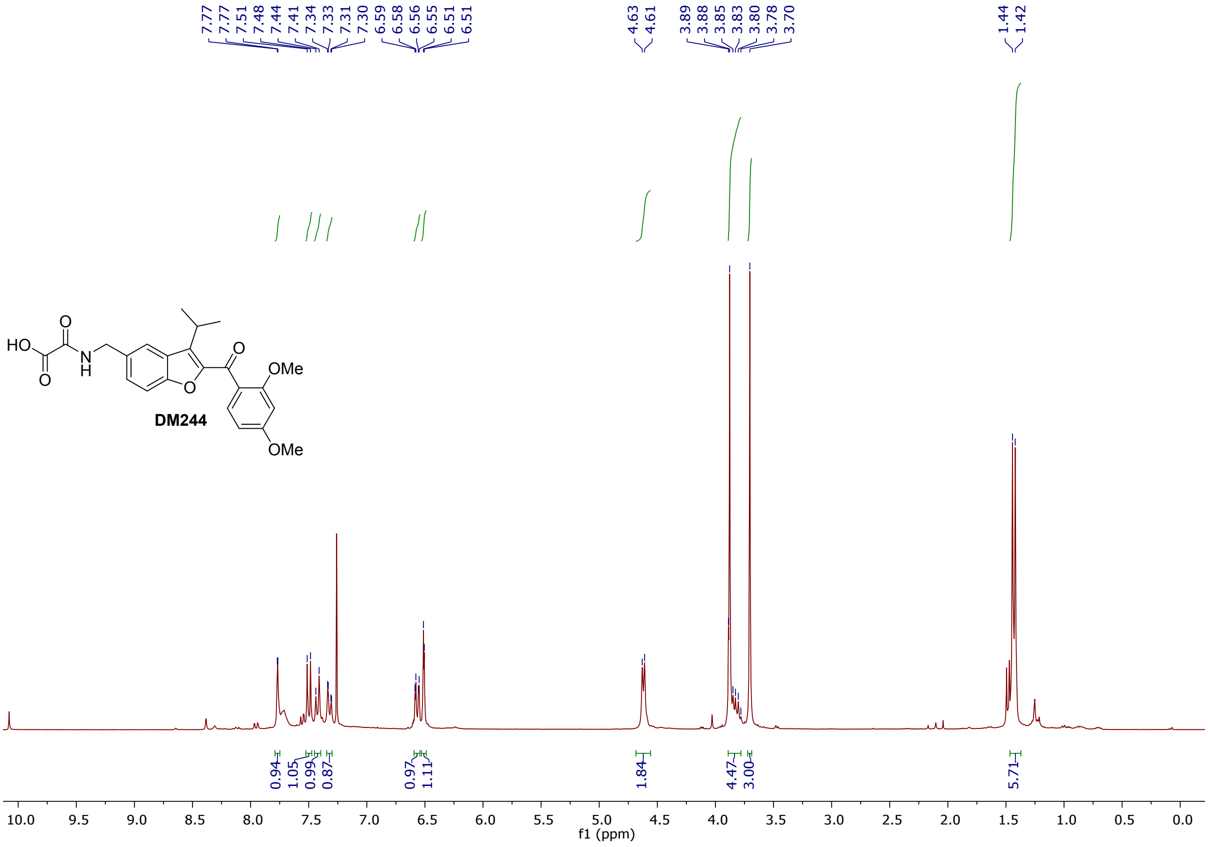

DM244
HO
O
O
N
H
O
O
OMe
OMe
f1 (ppm)

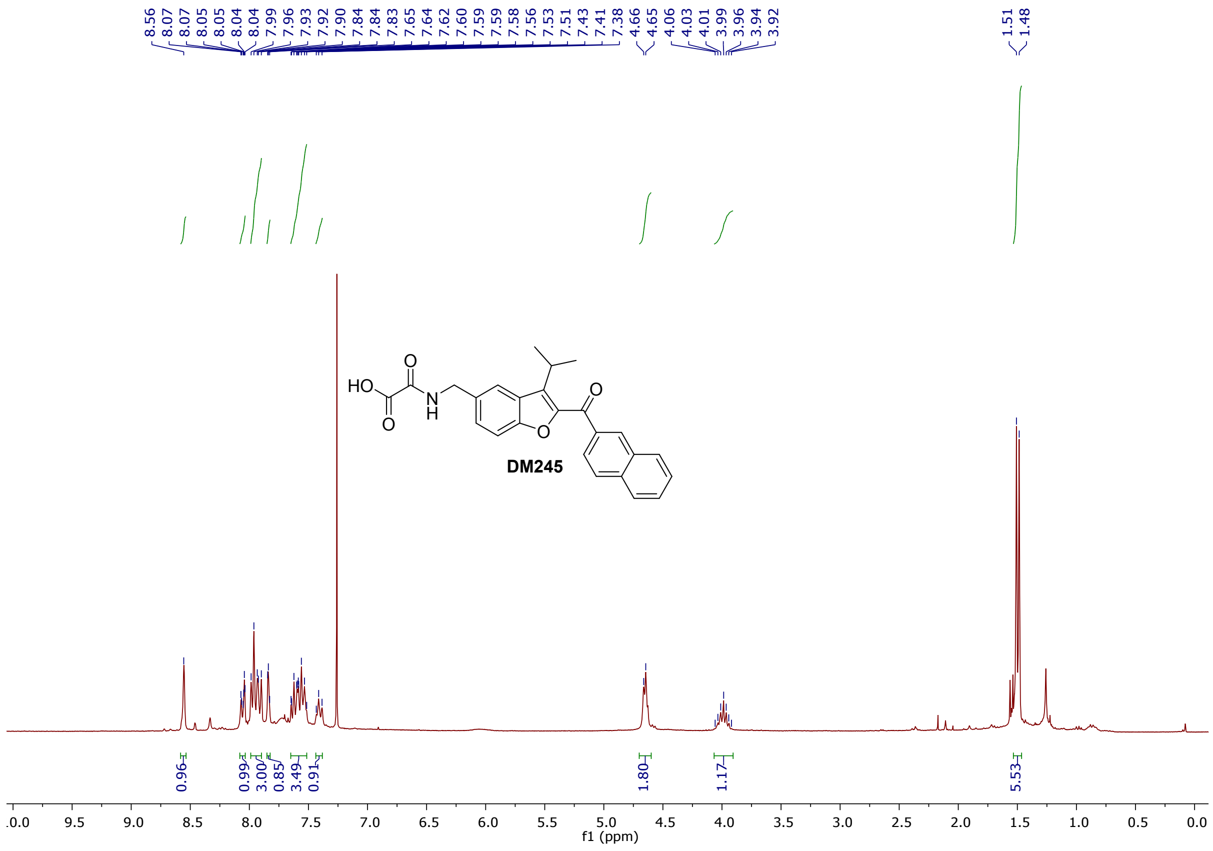
8.56
8.07
8.07
8.05
8.04
8.04
7.99
7.96
7.93
7.92
7.90
7.84
7.83
7.65
7.64
7.62
7.60
7.59
7.59
7.58
7.56
7.53
7.51
7.43
7.41
7.38
4.66
4.65
4.06
4.03
4.01
3.99
3.96
3.94
3.92
1.51
1.48
HO
O
O
N
H
O
O
DM245
0.96
0.99
3.00
0.85
3.49
0.91
1.80
1.17
5.53
f1 (ppm)

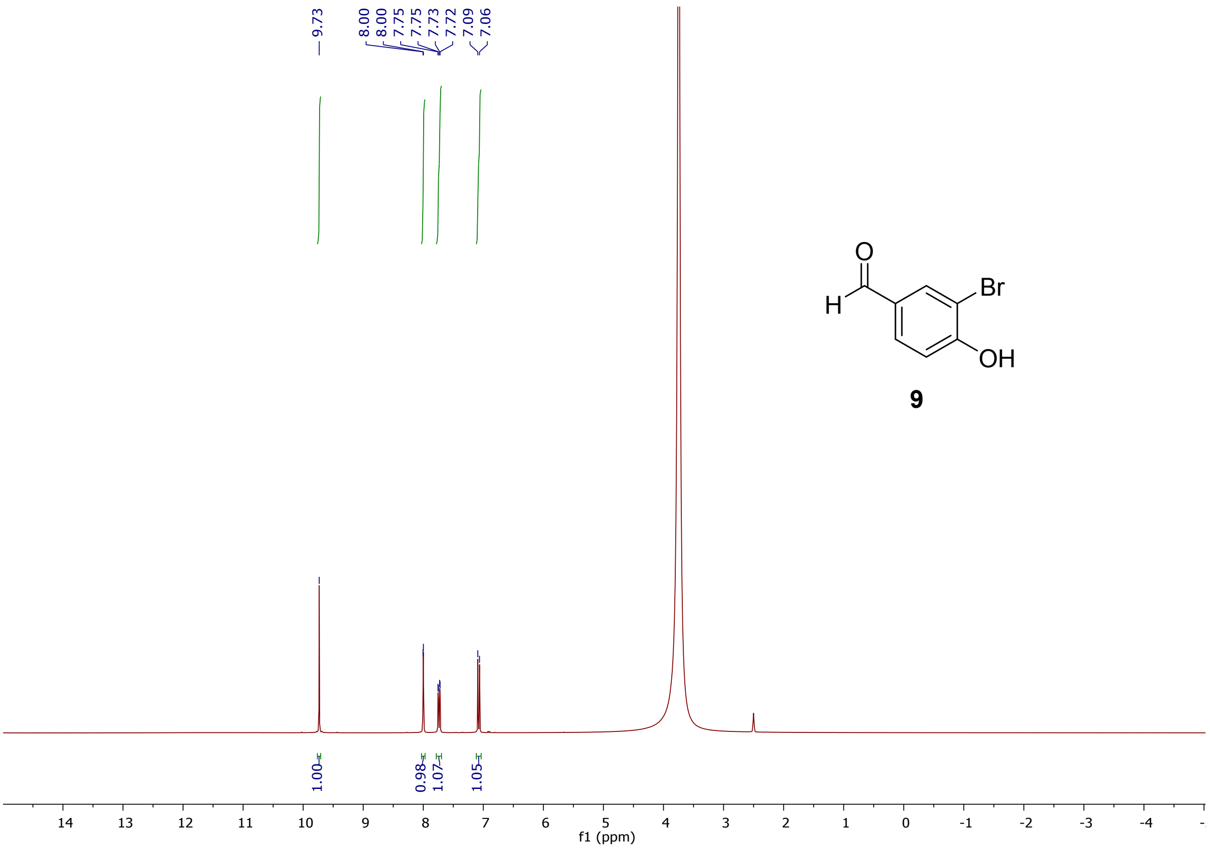
9.73
8.00
8.00
7.75
7.75
7.73
7.72
7.09
7.06
1.00
0.98
1.07
1.05
14 13 12 11 10 9 8 7 6 5 4 3 2 1 0 -1 -2 -3 -4
f1 (ppm)
O
H
Br
OH

9

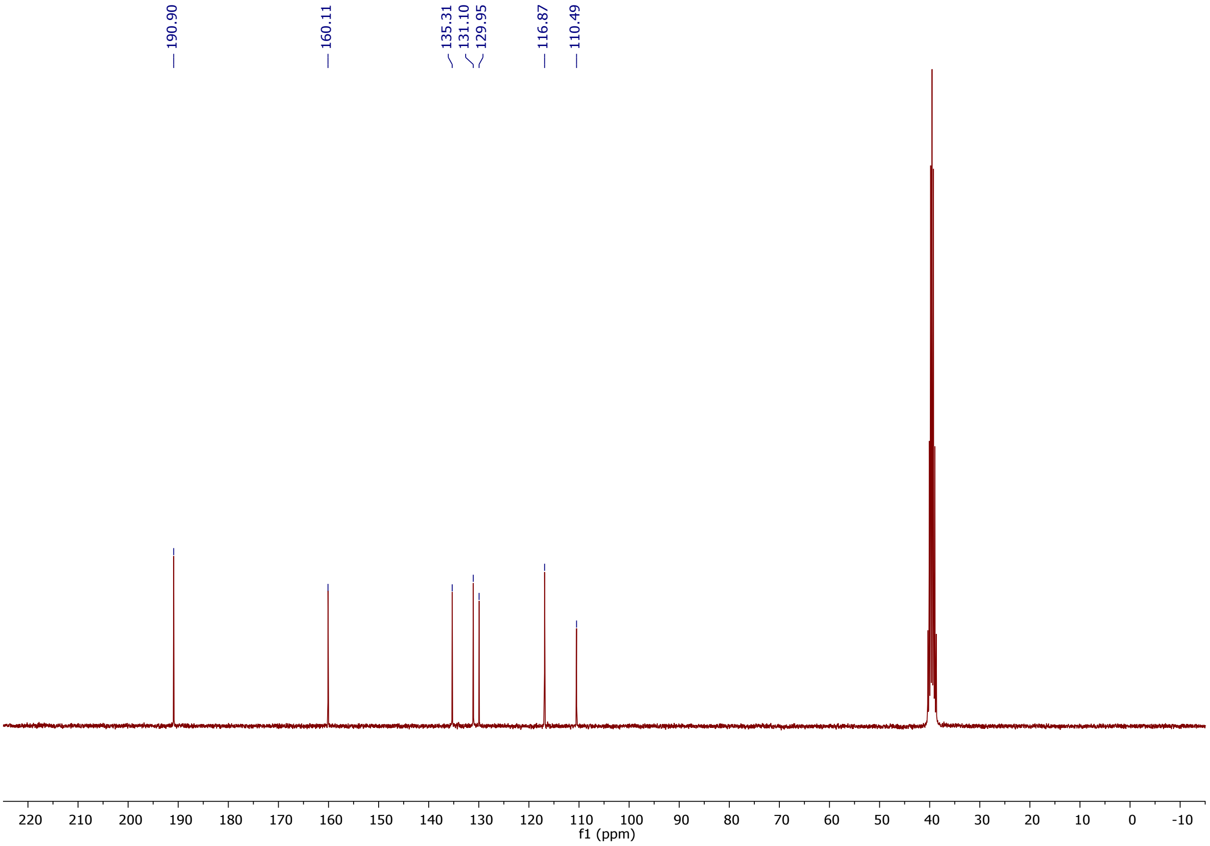
190.90
160.11
135.31
131.10
129.95
116.87
110.49
220 210 200 190 180 170 160 150 140 130 120 110 100 90 80 70 60 50 40 30 20 10 0 -10
f1 (ppm)

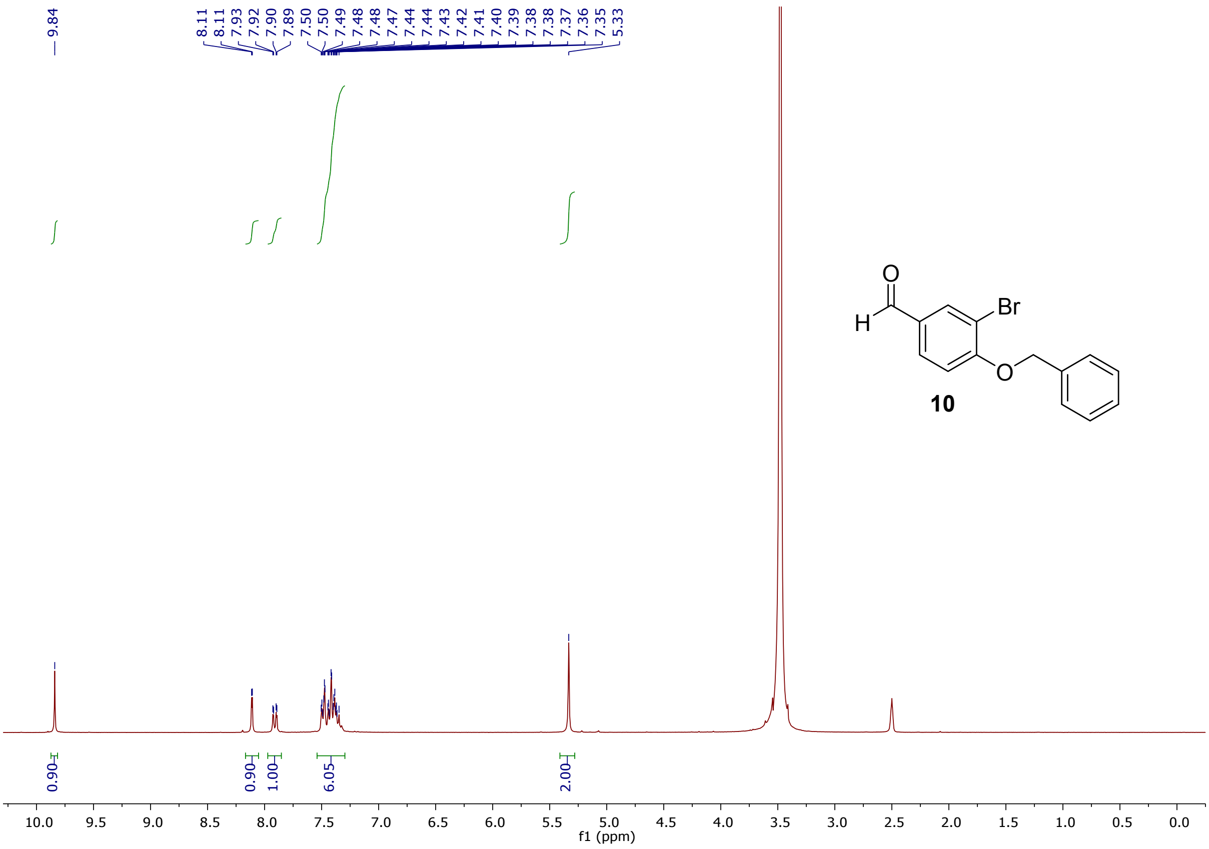
9.84
8.11
8.11
7.93
7.92
7.90
7.89
7.50
7.50
7.49
7.48
7.48
7.47
7.44
7.44
7.43
7.42
7.41
7.40
7.39
7.38
7.38
7.37
7.36
7.35
5.33
O
H
Br
O
10
0.90
0.90
1.00
6.05
2.00
10.0 9.5 9.0 8.5 8.0 7.5 7.0 6.5 6.0 5.5 5.0 4.5 4.0 3.5 3.0 2.5 2.0 1.5 1.0 0.5 0.0
f1 (ppm)

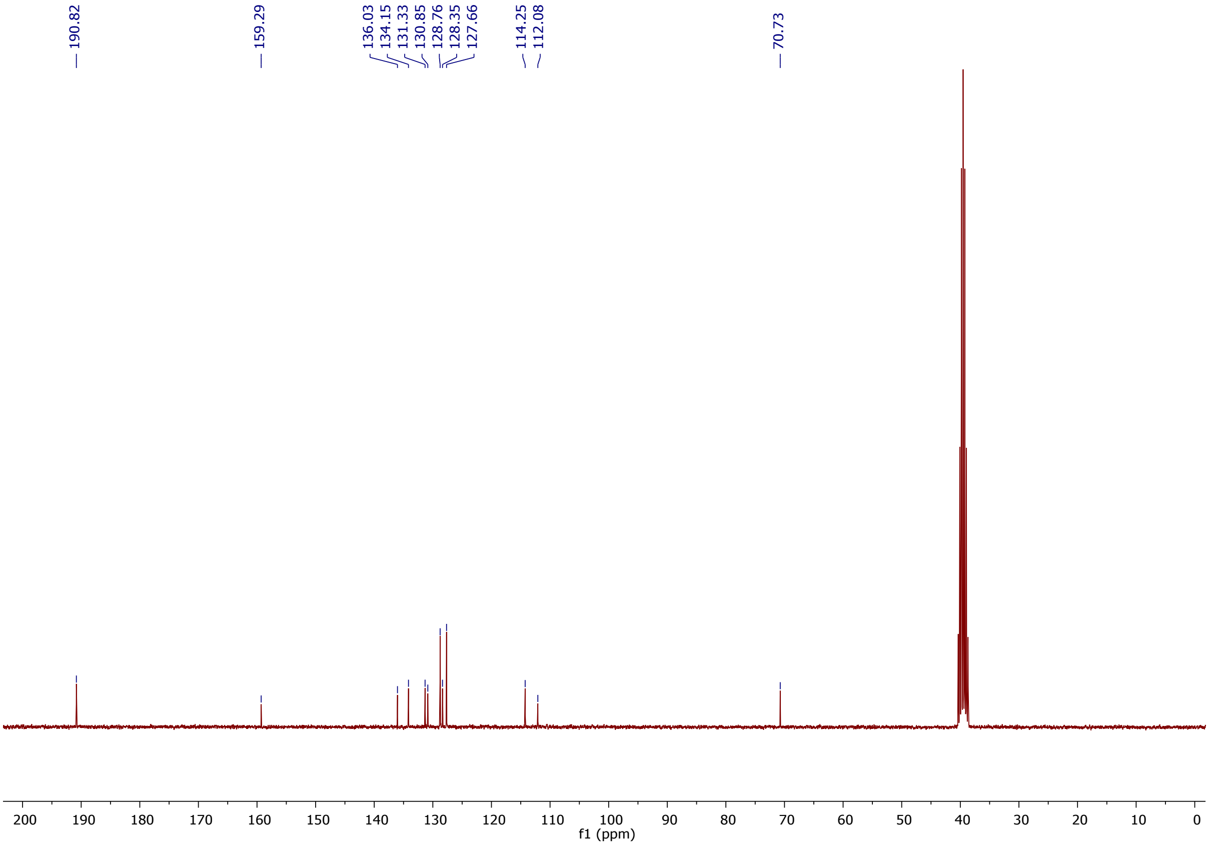
190.82
159.29
136.03
134.15
131.33
130.85
128.76
128.35
127.66
114.25
112.08
70.73
200 190 180 170 160 150 140 130 120 110 100 90 80 70 60 50 40 30 20 10 0
f1 (ppm)

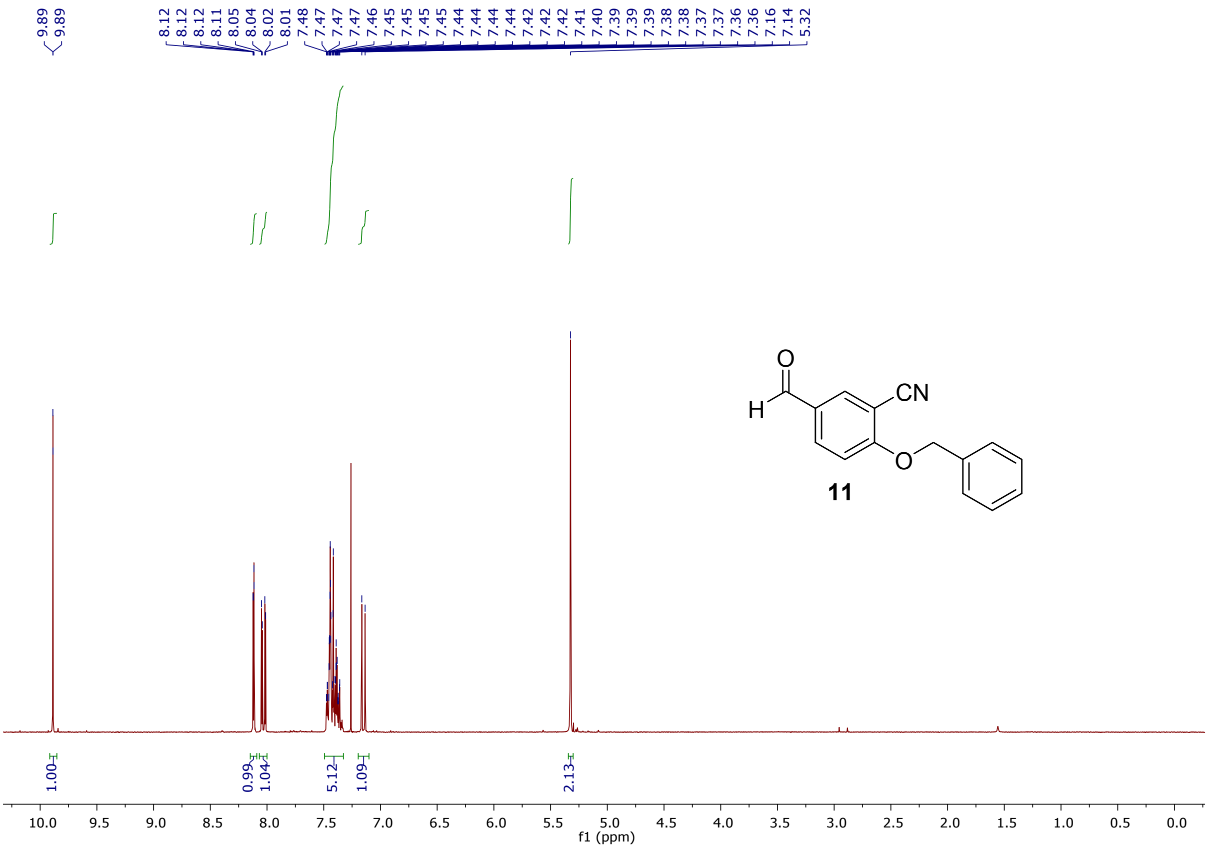

9.89
9.89
8.12
8.12
8.12
8.11
8.05
8.04
8.02
8.01
7.48
7.47
7.47
7.47
7.46
7.45
7.45
7.45
7.44
7.44
7.44
7.44
7.42
7.42
7.42
7.41
7.40
7.39
7.39
7.39
7.38
7.38
7.37
7.37
7.36
7.16
7.14
5.32
O
H
CN
O
11
1.00
0.99
1.04
5.12
1.09
2.13
10.0 9.5 9.0 8.5 8.0 7.5 7.0 6.5 6.0 5.5 5.0 4.5 4.0 3.5 3.0 2.5 2.0 1.5 1.0 0.5 0.0
f1 (ppm)

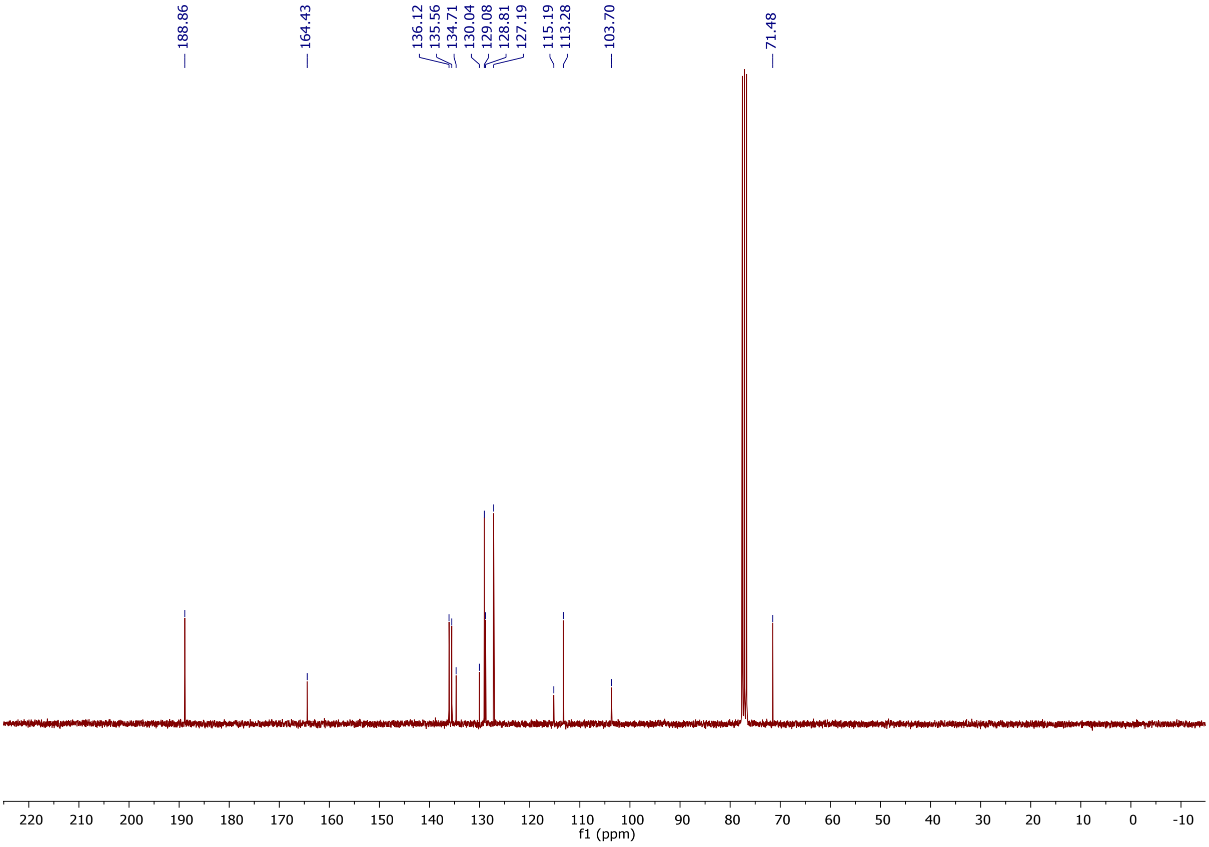

188.86
164.43
136.12
135.56
134.71
130.04
129.08
128.81
127.19
115.19
113.28
103.70
71.48
220 210 200 190 180 170 160 150 140 130 120 110 100 90 80 70 60 50 40 30 20 10 0 -10
f1 (ppm)

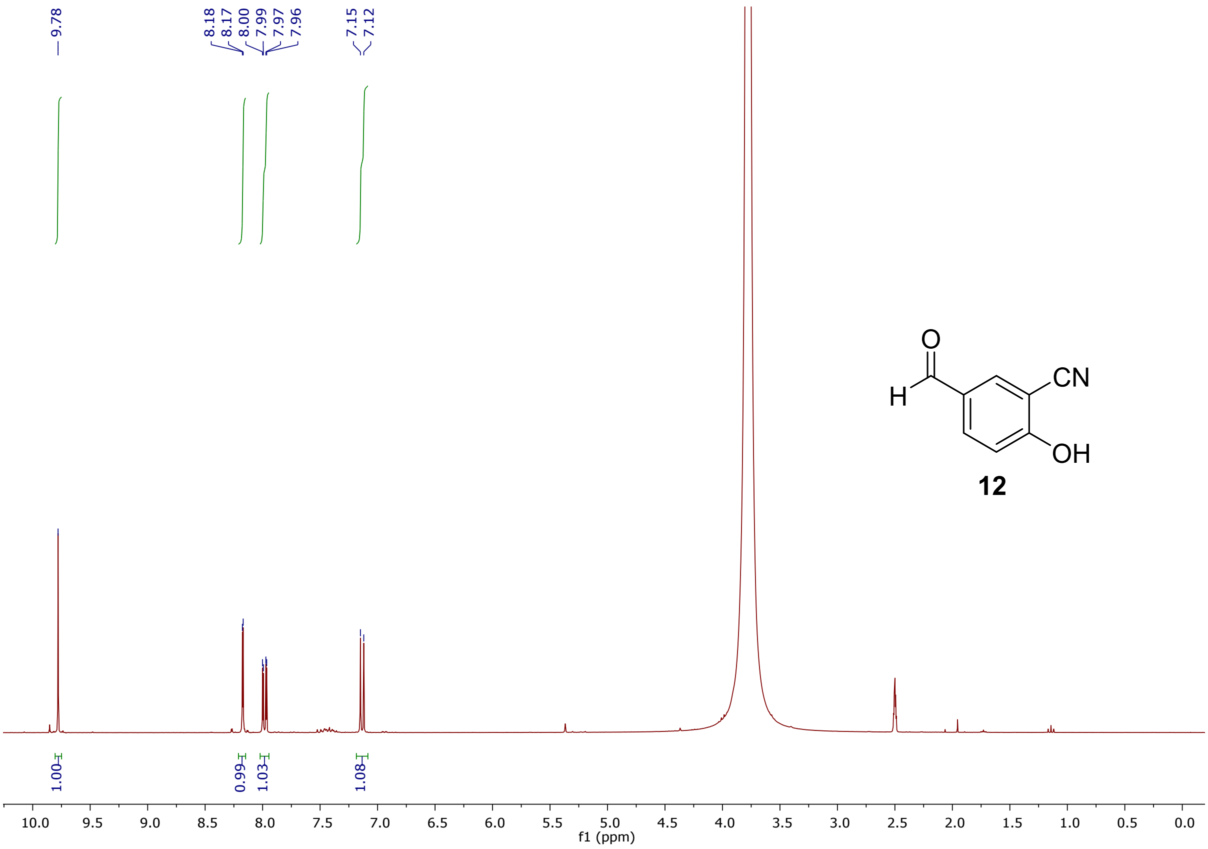
9.78
8.18
8.17
8.00
7.99
7.97
7.96
7.15
7.12
1.00
0.99
1.03
1.08
f1 (ppm)
O
H
CN
OH
12

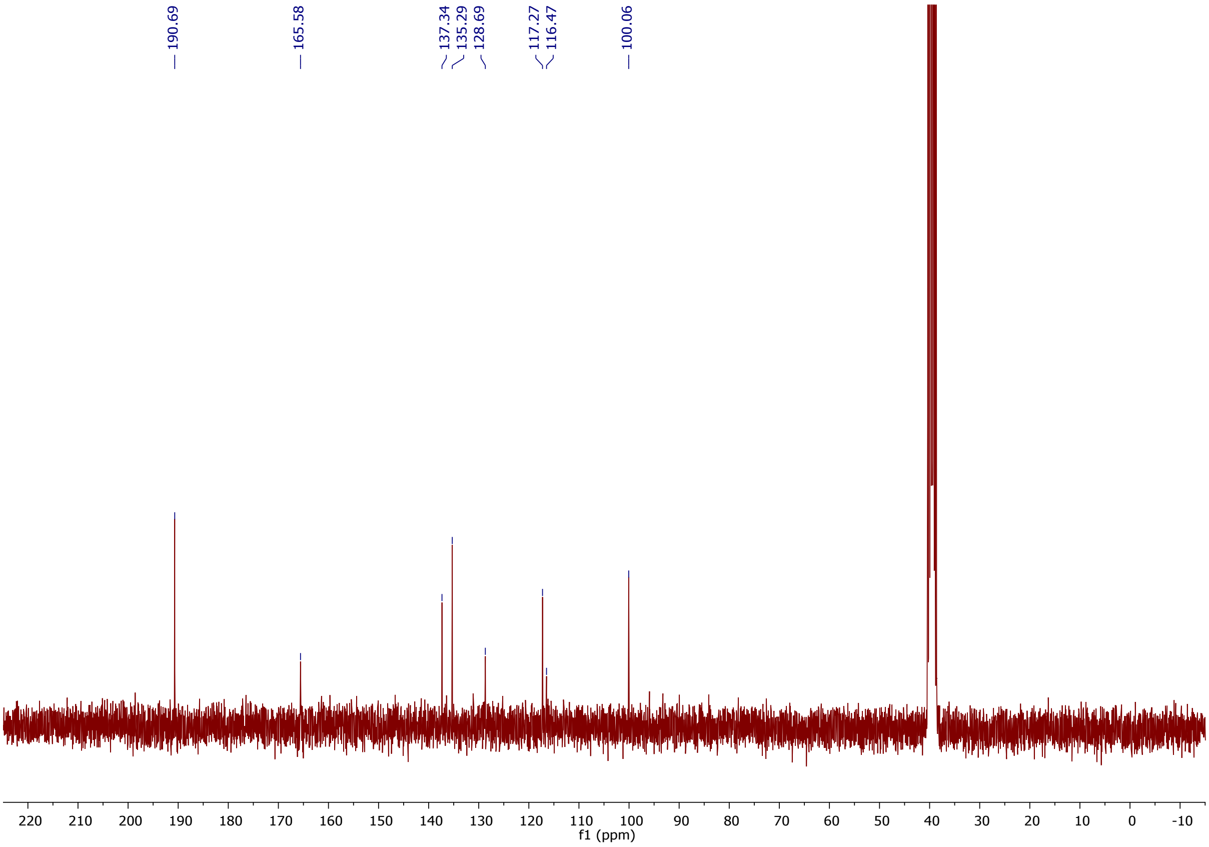
190.69
165.58
137.34
135.29
128.69
117.27
116.47
100.06
f1 (ppm)

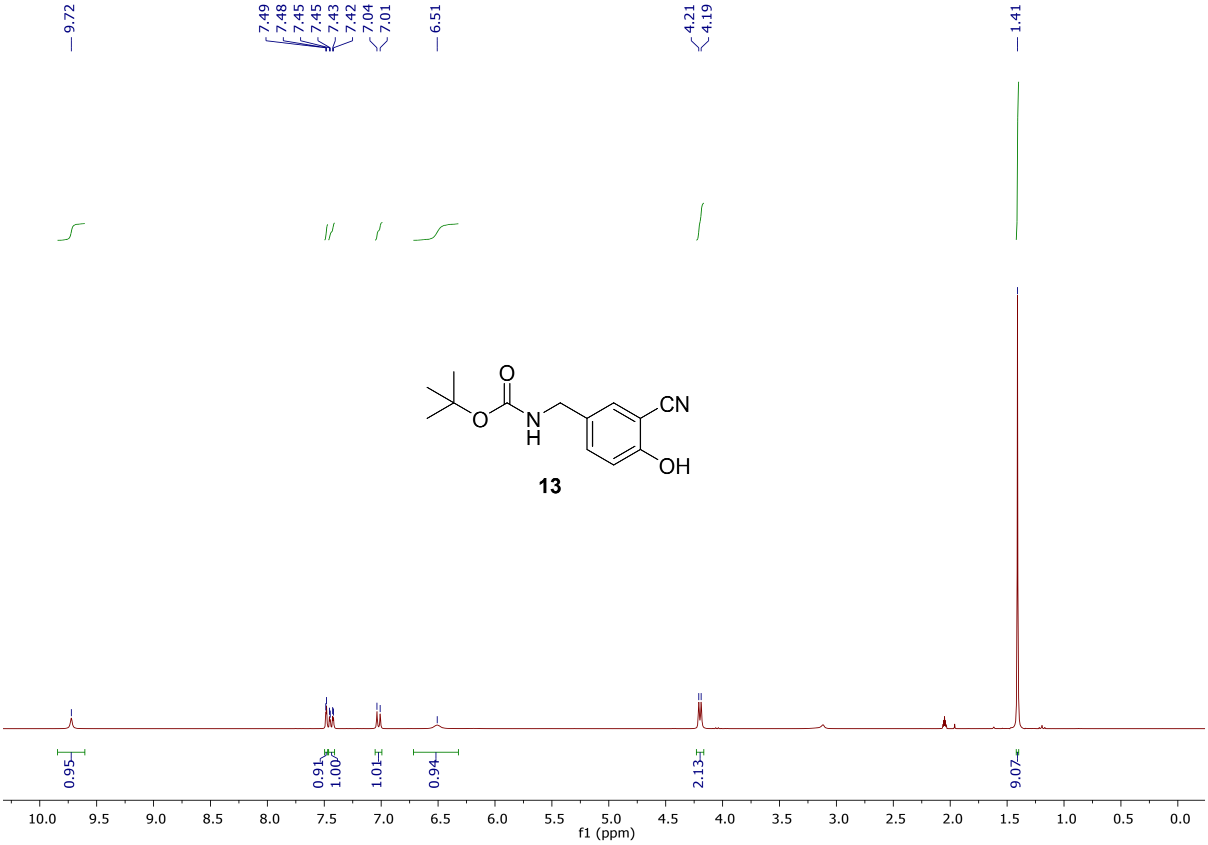
9.72
7.49
7.48
7.45
7.45
7.43
7.42
7.04
7.01
6.51
4.21
4.19
1.41
O
O
N
H
CN
OH
13
0.95
0.91
1.00
1.01
0.94
2.13
9.07
10.0 9.5 9.0 8.5 8.0 7.5 7.0 6.5 6.0 5.5 5.0 4.5 4.0 3.5 3.0 2.5 2.0 1.5 1.0 0.5 0.0
f1 (ppm)

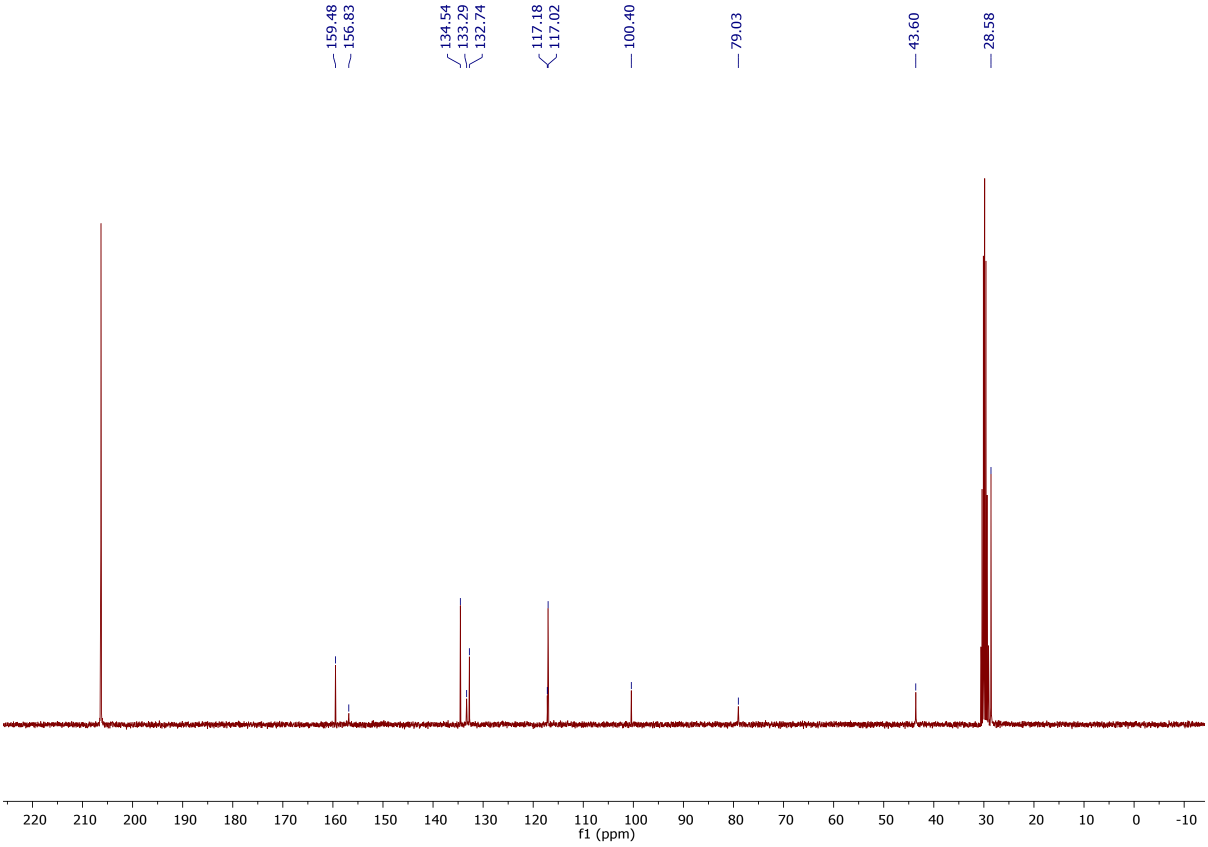
159.48
156.83
134.54
133.29
132.74
117.18
117.02
100.40
79.03
43.60
28.58
220 210 200 190 180 170 160 150 140 130 120 110 100 90 80 70 60 50 40 30 20 10 0 -10
f1 (ppm)

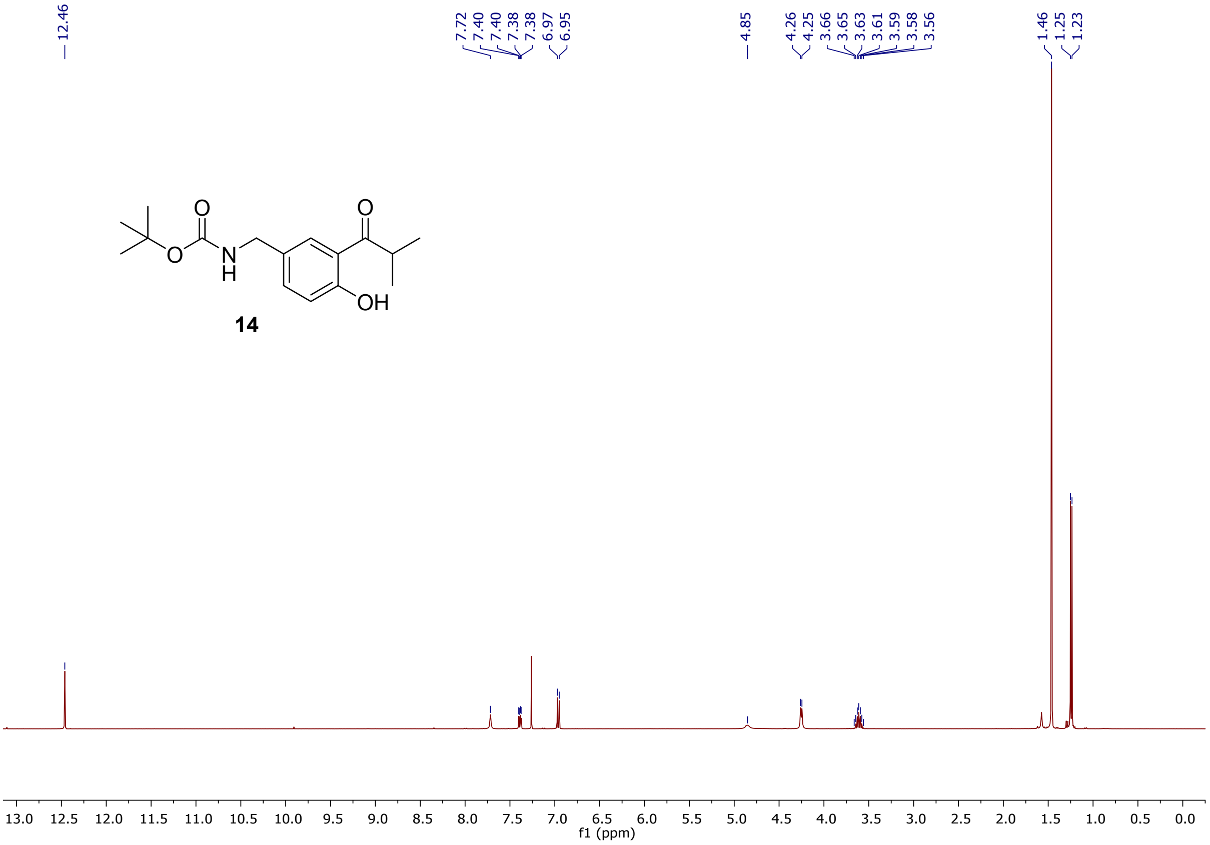
O
O
O
N
H
OH
14
12.46
7.72
7.40
7.40
7.38
7.38
6.97
6.95
4.85
4.26
4.25
3.66
3.65
3.63
3.61
3.59
3.58
3.56
1.46
1.25
1.23
13.0 12.5 12.0 11.5 11.0 10.5 10.0 9.5 9.0 8.5 8.0 7.5 7.0 6.5 6.0 5.5 5.0 4.5 4.0 3.5 3.0 2.5 2.0 1.5 1.0 0.5 0.0
f1 (ppm)

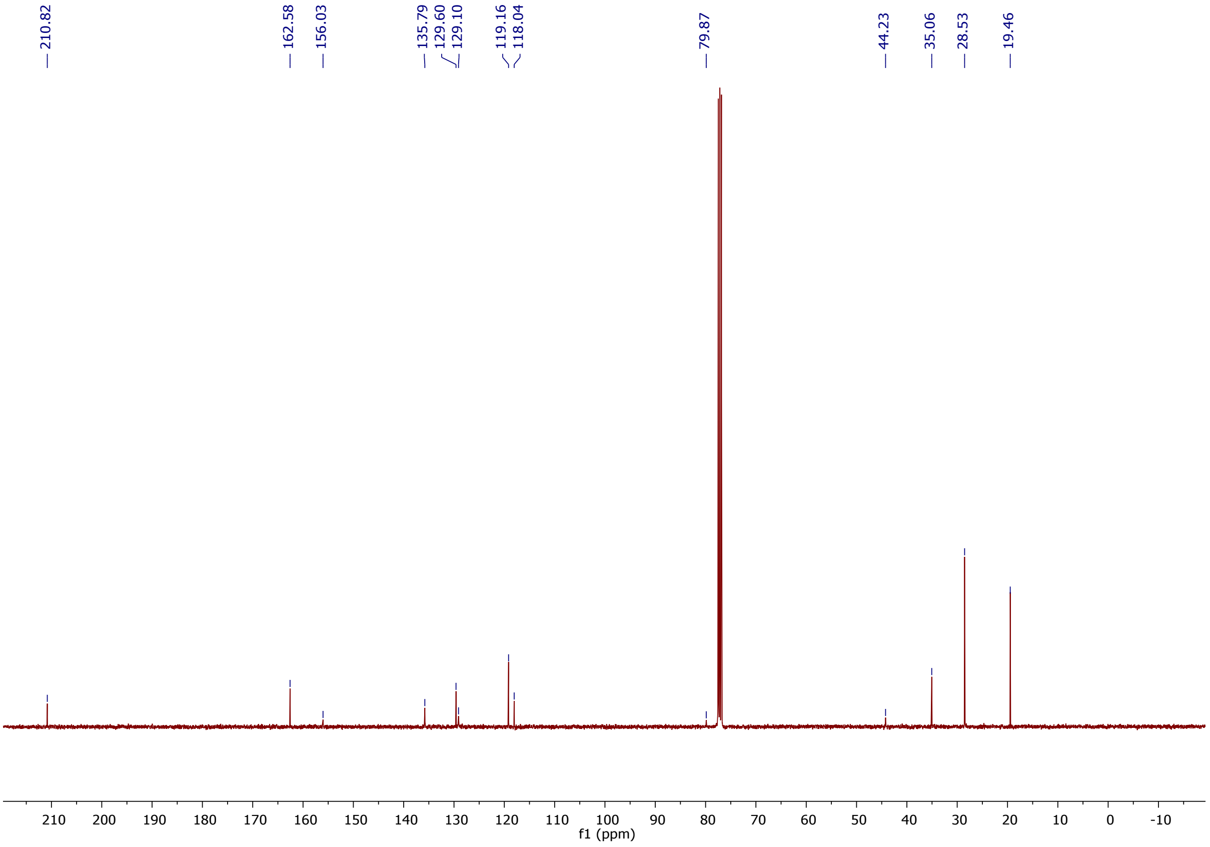
210.82
162.58
156.03
135.79
129.60
129.10
119.16
118.04
79.87
44.23
35.06
28.53
19.46
210 200 190 180 170 160 150 140 130 120 110 100 90 80 70 60 50 40 30 20 10 0 -10
f1 (ppm)

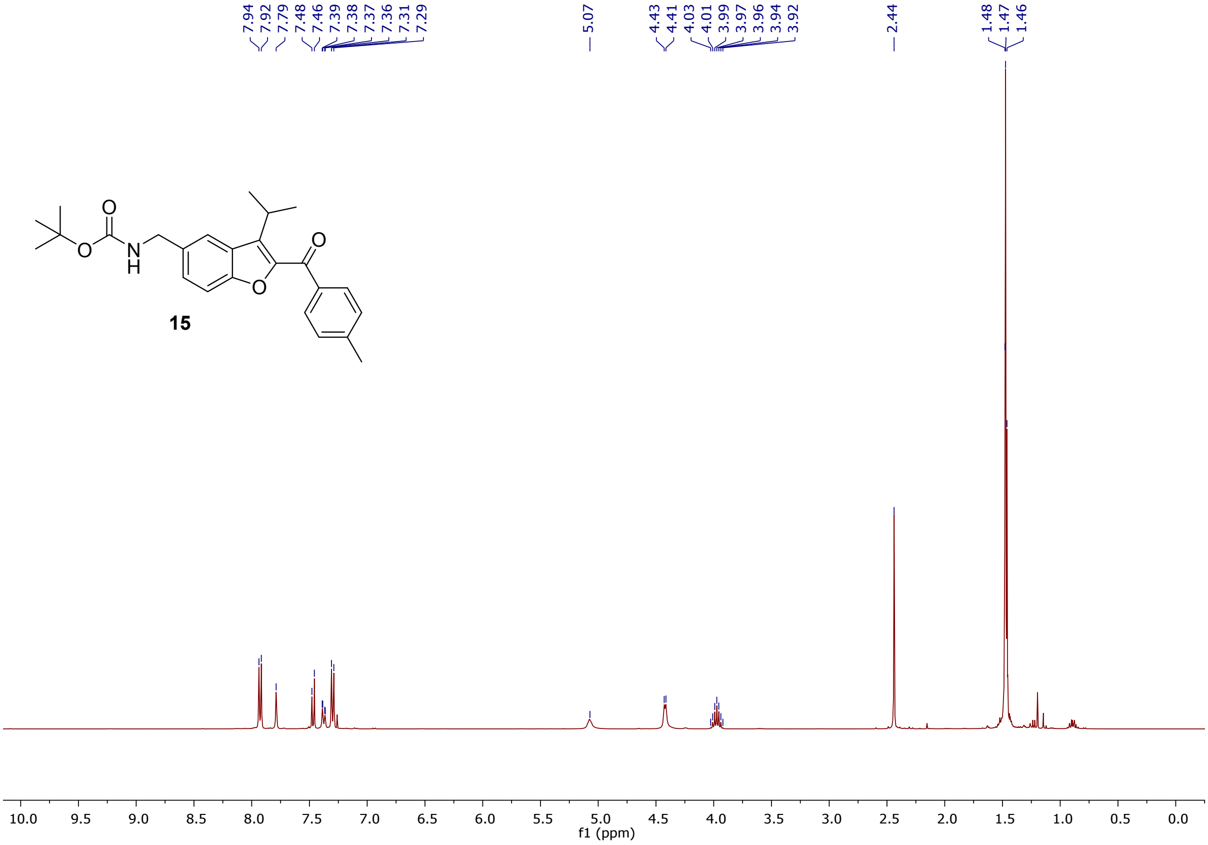
7.94
7.92
7.79
7.48
7.46
7.39
7.38
7.37
7.36
7.31
7.29
5.07
4.43
4.41
4.03
4.01
3.99
3.97
3.96
3.94
3.92
2.44
1.48
1.47
1.46
10.0 9.5 9.0 8.5 8.0 7.5 7.0 6.5 6.0 5.5 5.0 4.5 4.0 3.5 3.0 2.5 2.0 1.5 1.0 0.5 0.0
f1 (ppm)
O
O
N
H
O
O

15

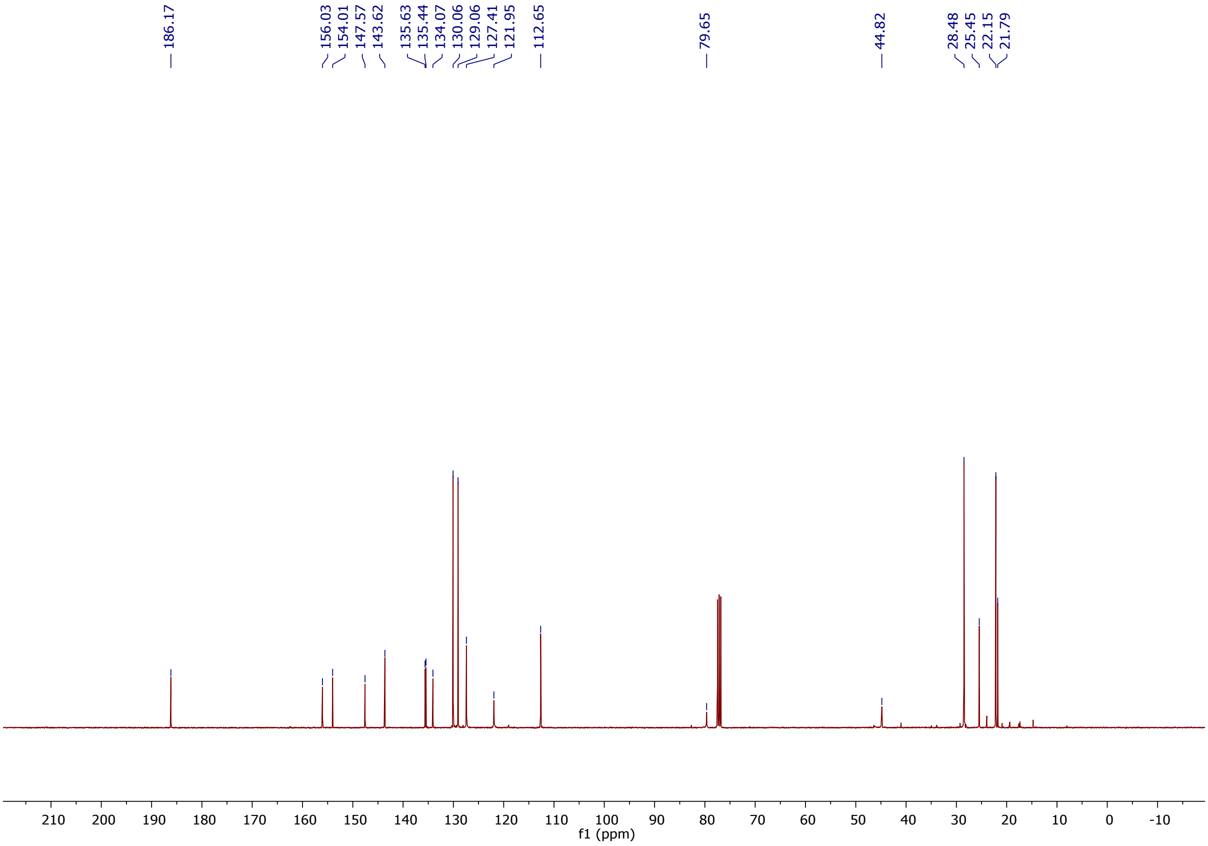
186.17
156.03
154.01
147.57
143.62
135.63
135.44
134.07
130.06
129.06
127.41
121.95
112.65
79.65
44.82
28.48
25.45
22.15
21.79
210 200 190 180 170 160 150 140 130 120 110 100 90 80 70 60 50 40 30 20 10 0 -10
f1 (ppm)

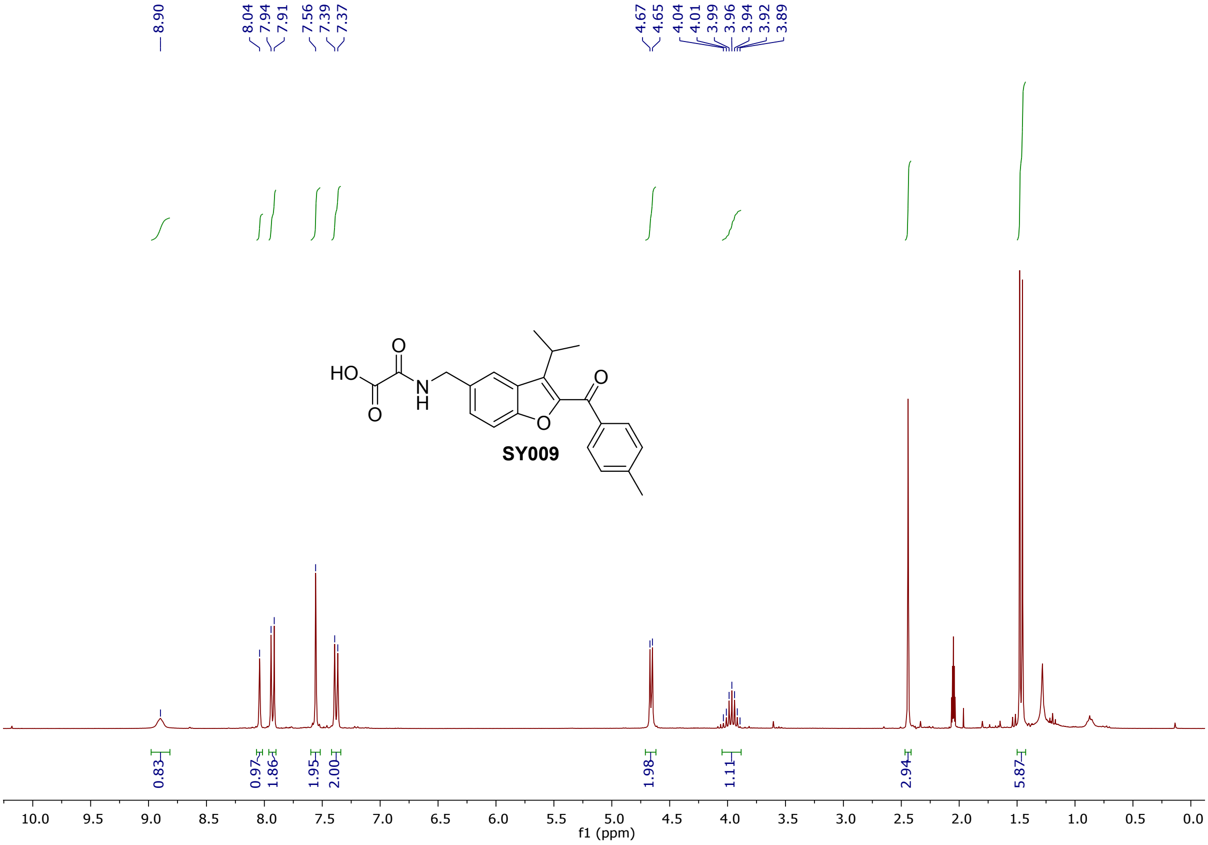

8.90
8.04
7.94
7.91
7.56
7.39
7.37
4.67
4.65
4.04
4.01
3.99
3.96
3.94
3.92
3.89
HO
O
O
N
H
O
O
SY009
0.83
0.97
1.86
1.95
2.00
1.98
1.11
2.94
5.87
10.0 9.5 9.0 8.5 8.0 7.5 7.0 6.5 6.0 5.5 5.0 4.5 4.0 3.5 3.0 2.5 2.0 1.5 1.0 0.5 0.0
f1 (ppm)

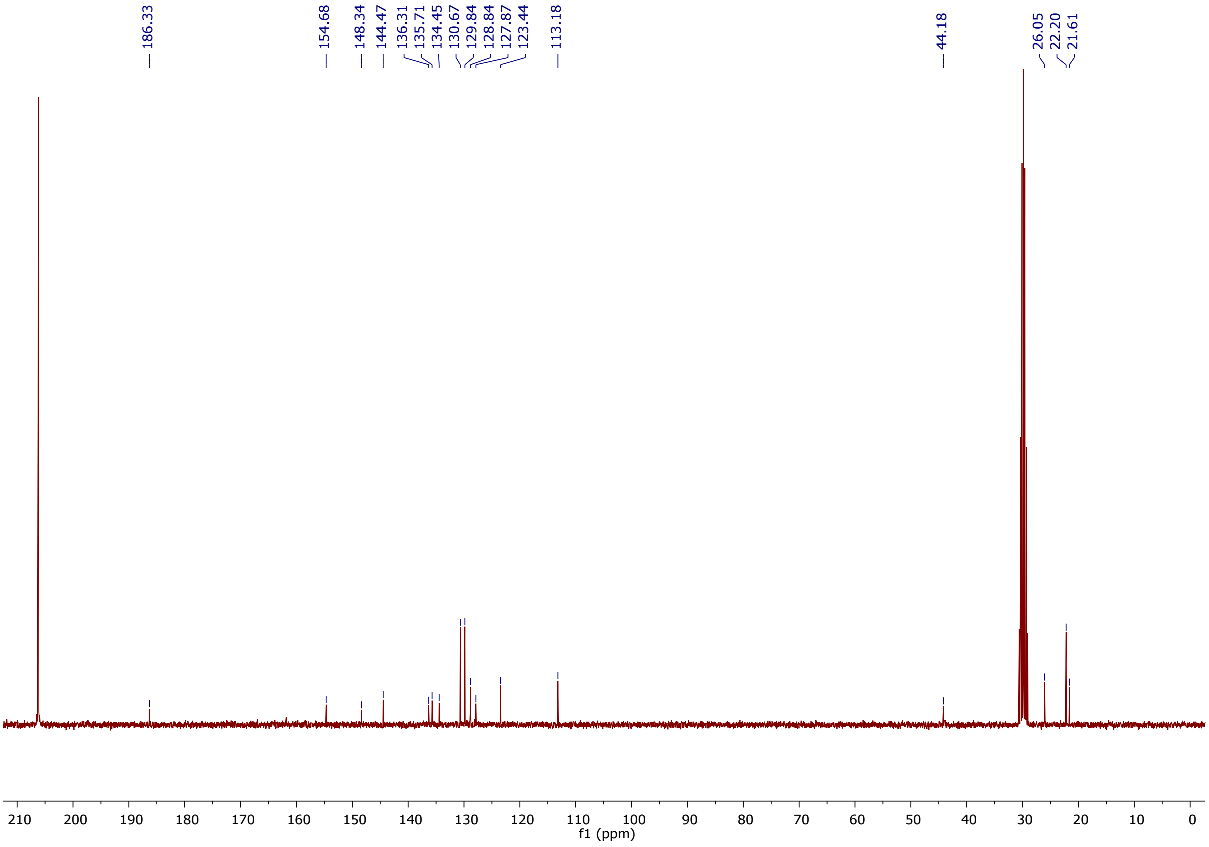

186.33
154.68
148.34
144.47
136.31
135.71
134.45
130.67
129.84
128.84
127.87
123.44
113.18
44.18
26.05
22.20
21.61
210 200 190 180 170 160 150 140 130 120 110 100 90 80 70 60 50 40 30 20 10 0
f1 (ppm)

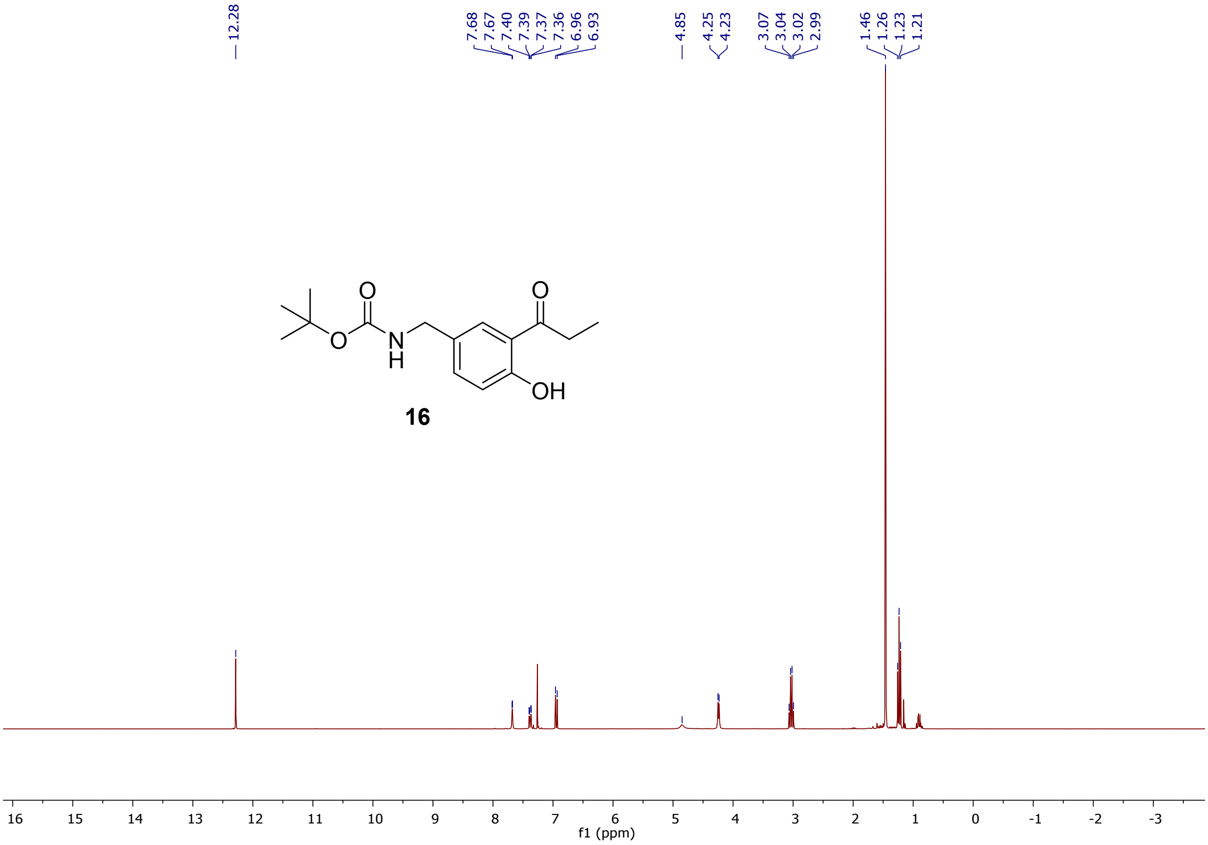
12.28
7.68
7.67
7.40
7.39
7.37
7.36
6.96
6.93
4.85
4.25
4.23
3.07
3.04
3.02
2.99
1.46
1.26
1.23
1.21
O
O
N
H
O
OH
16 15 14 13 12 11 10 9 8 7 6 5 4 3 2 1 0 -1 -2 -3
f1 (ppm)

16

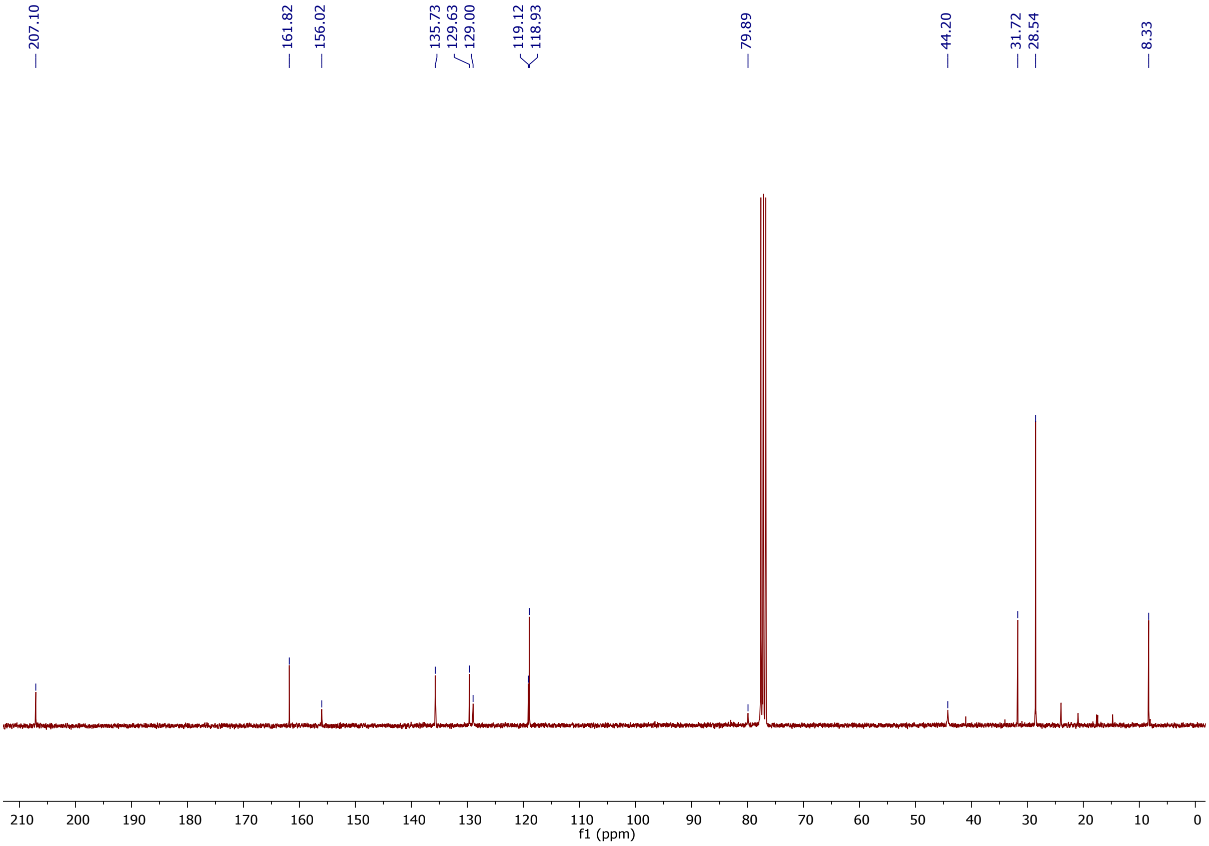
207.10
161.82
156.02
135.73
129.63
129.00
119.12
118.93
79.89
44.20
31.72
28.54
8.33
210 200 190 180 170 160 150 140 130 120 110 100 90 80 70 60 50 40 30 20 10 0
f1 (ppm)

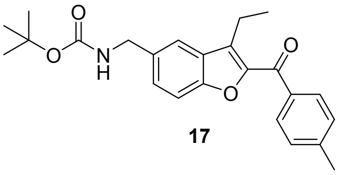

17

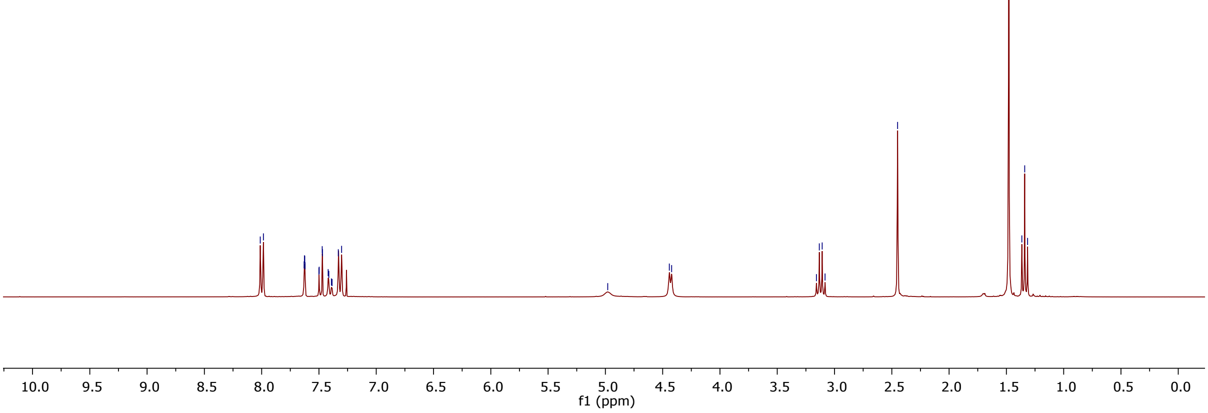
8.01
7.98
7.63
7.63
7.62
7.62
7.50
7.50
7.47
7.47
7.42
7.41
7.39
7.39
7.33
7.30
4.98
4.44
4.42
3.16
3.13
3.11
3.08
2.45
1.48
1.37
1.34
1.32
f1 (ppm)

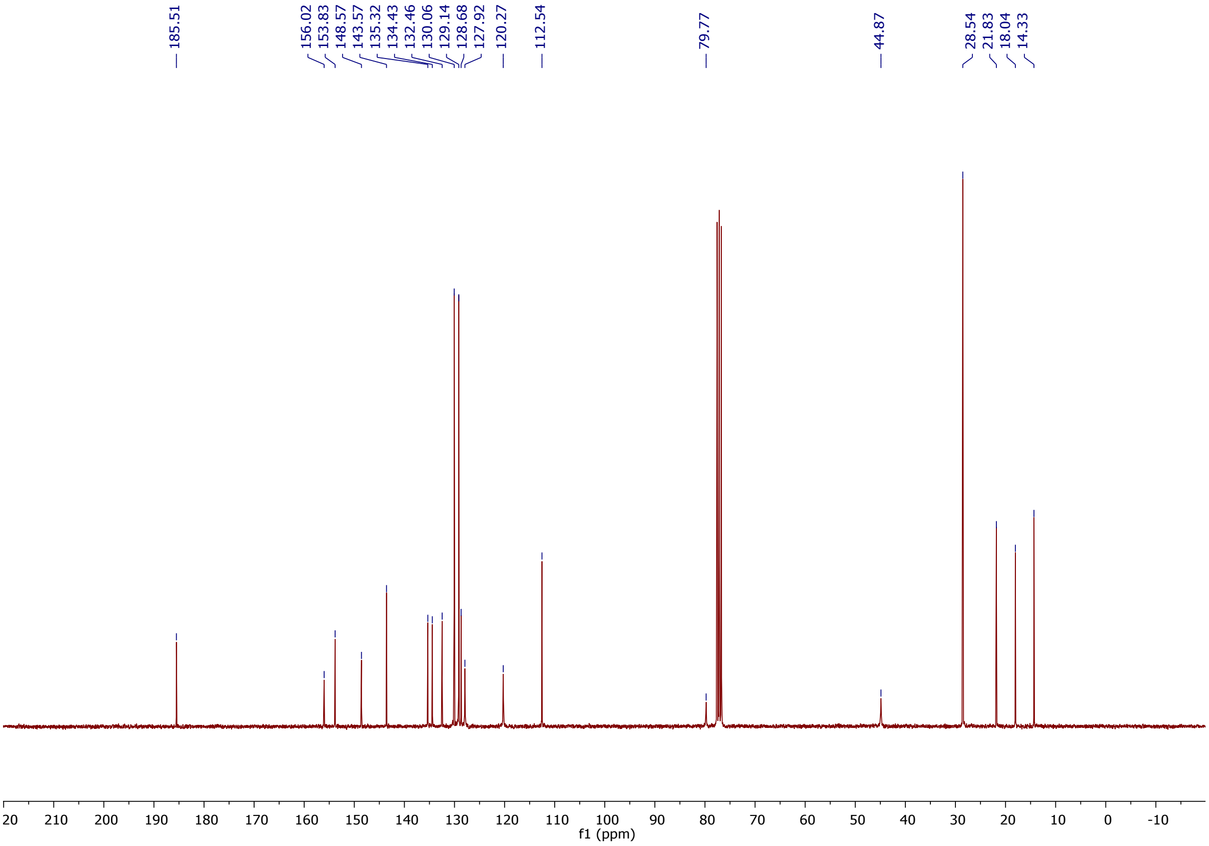
185.51
156.02
153.83
148.57
143.57
135.32
134.43
132.46
130.06
129.14
128.68
127.92
120.27
112.54
79.77
44.87
28.54
21.83
18.04
14.33
f1 (ppm)

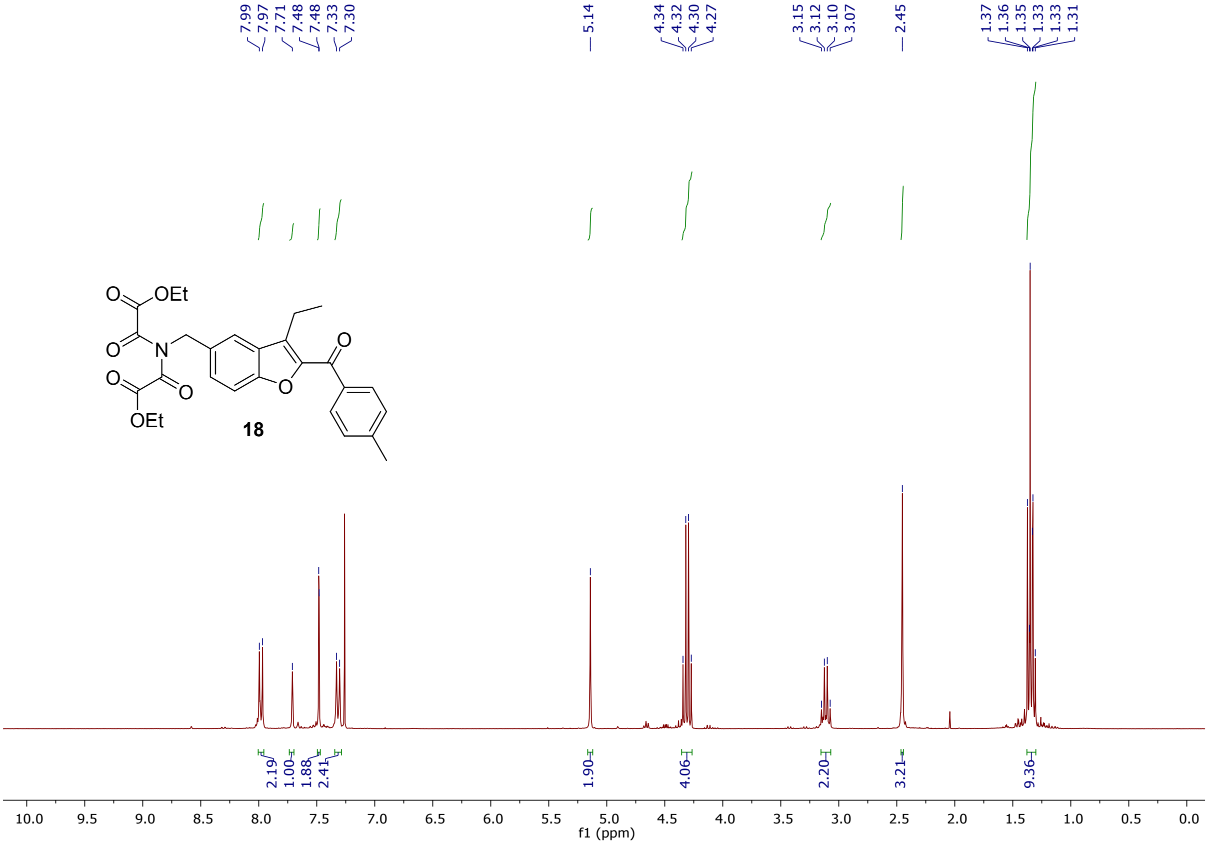
7.99
7.97
7.71
7.48
7.48
7.33
7.30
5.14
4.34
4.32
4.30
4.27
3.15
3.12
3.10
3.07
2.45
1.37
1.36
1.35
1.33
1.33
1.31
OEt
O
O
N
O
O
OEt
O
O
18
2.19
1.00
1.88
2.41
1.90
4.06
2.20
3.21
9.36
10.0 9.5 9.0 8.5 8.0 7.5 7.0 6.5 6.0 5.5 5.0 4.5 4.0 3.5 3.0 2.5 2.0 1.5 1.0 0.5 0.0
f1 (ppm)

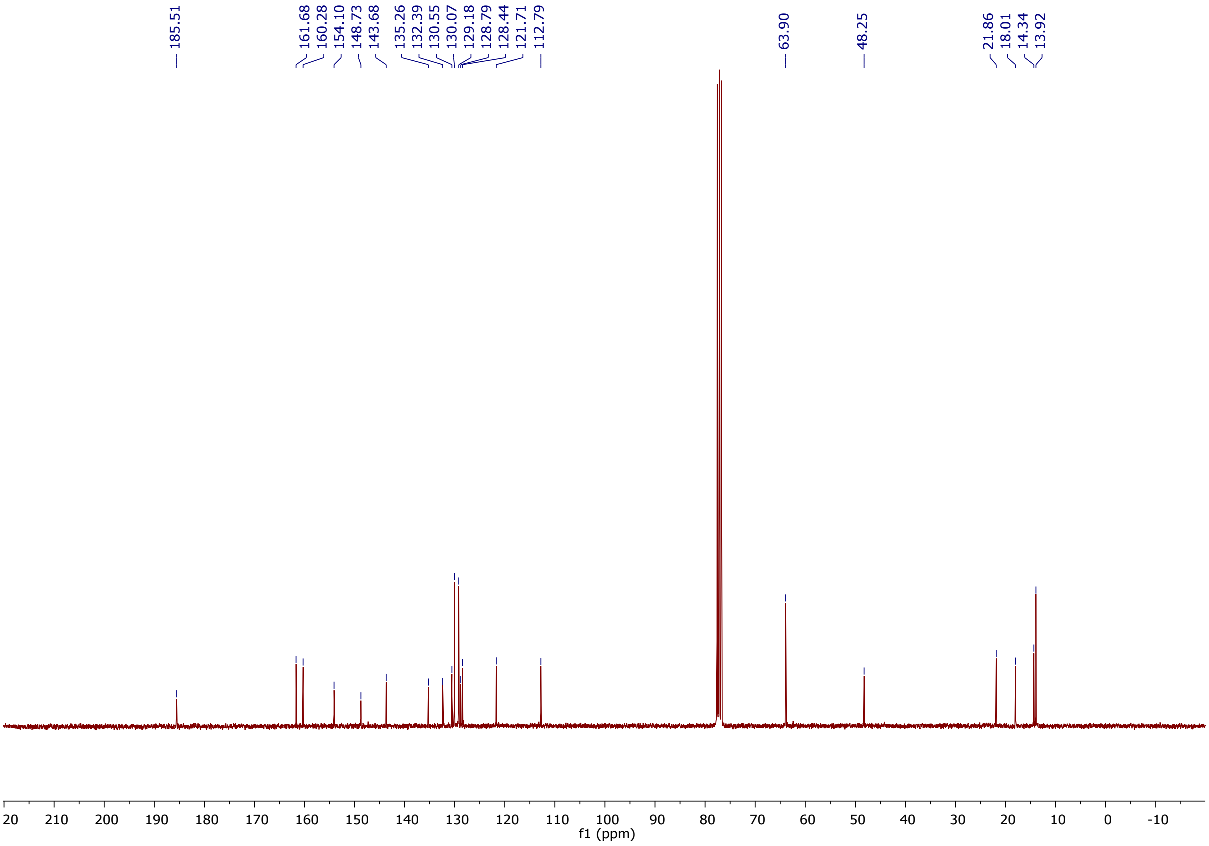
185.51
161.68
160.28
154.10
148.73
143.68
135.26
132.39
130.55
130.07
129.18
128.79
128.44
121.71
112.79
63.90
48.25
21.86
18.01
14.34
13.92
20 210 200 190 180 170 160 150 140 130 120 110 100 90 80 70 60 50 40 30 20 10 0 -10
f1 (ppm)

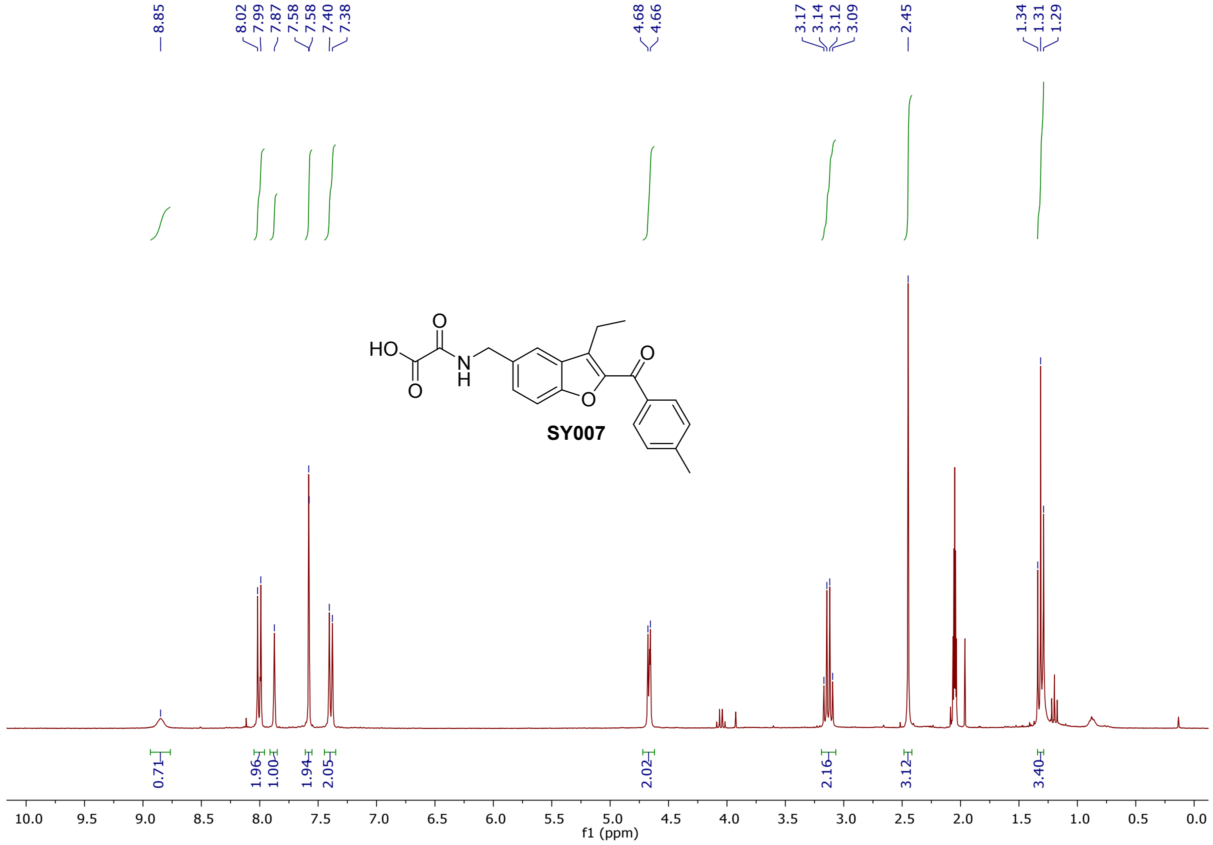
8.85
8.02
7.99
7.87
7.58
7.58
7.40
7.38
4.68
4.66
3.17
3.14
3.12
3.09
2.45
1.34
1.31
1.29
HO
O
N
H
O
O
SY007
0.71
1.96
1.00
1.94
2.05
2.02
2.16
3.12
3.40
10.0 9.5 9.0 8.5 8.0 7.5 7.0 6.5 6.0 5.5 5.0 4.5 4.0 3.5 3.0 2.5 2.0 1.5 1.0 0.5 0.0
f1 (ppm)

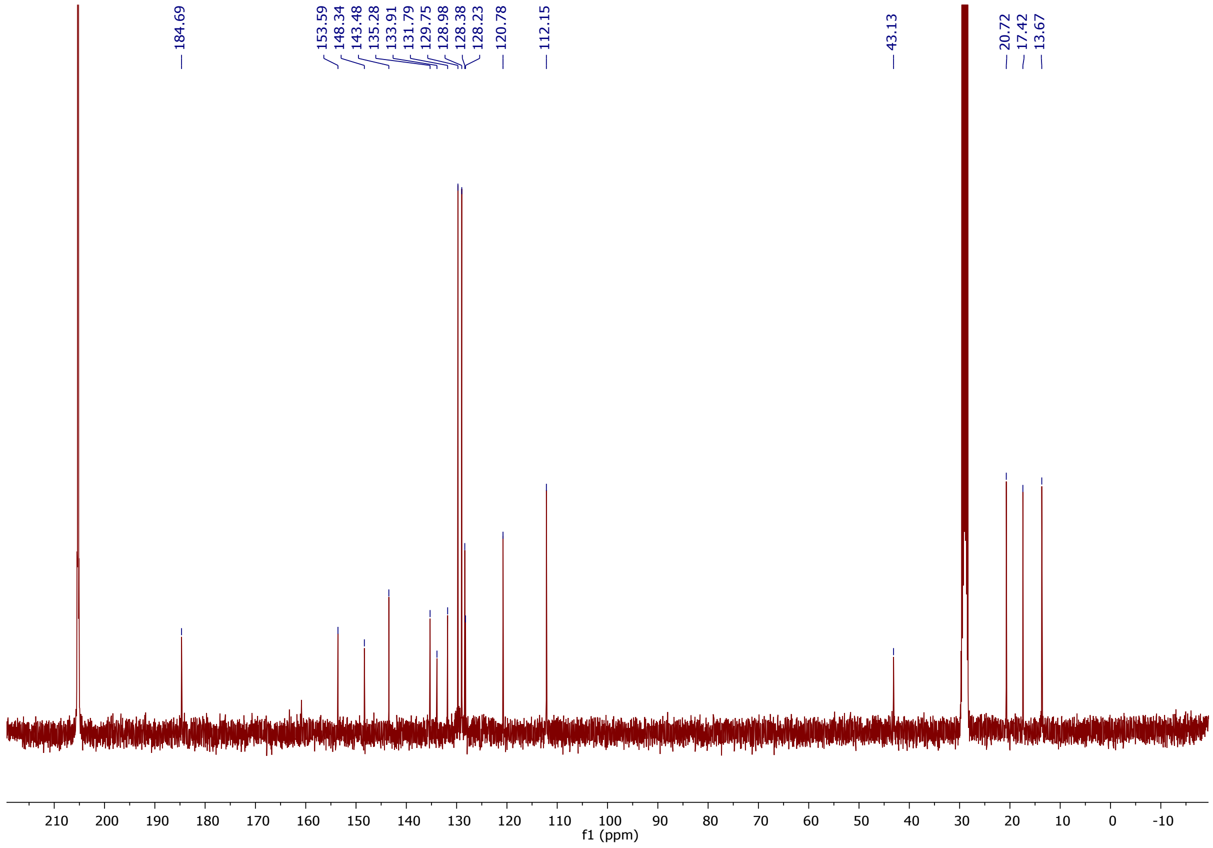
184.69
153.59
148.34
143.48
135.28
133.91
131.79
129.75
128.98
128.38
128.23
120.78
112.15
43.13
20.72
17.42
13.67
210 200 190 180 170 160 150 140 130 120 110 100 90 80 70 60 50 40 30 20 10 0 -10
f1 (ppm)

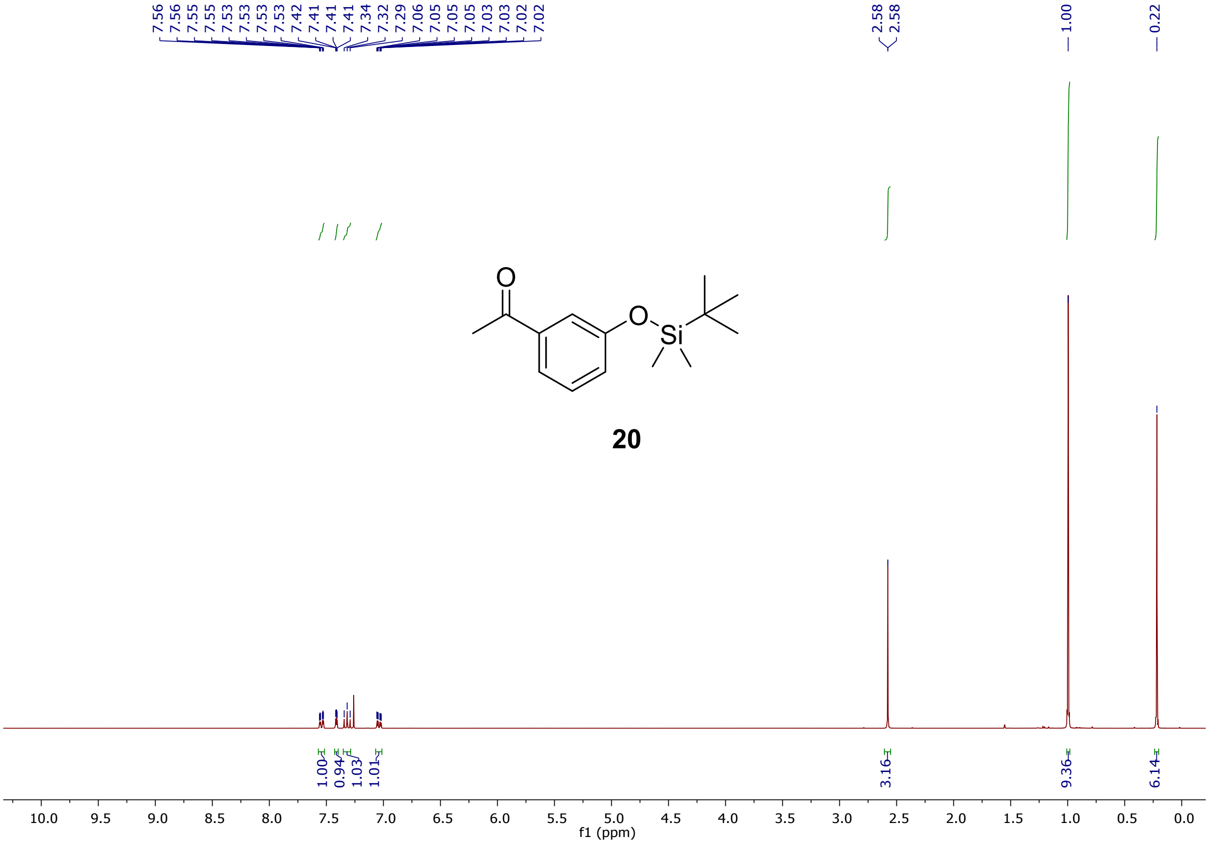
7.56 7.56 7.55 7.55 7.53 7.53 7.53 7.53 7.42 7.41 7.41 7.41 7.34 7.32 7.29 7.06 7.05 7.05 7.05 7.03 7.03 7.02 7.02
2.58 2.58
1.00
0.22
1.00 0.94 1.03 1.01
3.16
9.36
6.14
10.0 9.5 9.0 8.5 8.0 7.5 7.0 6.5 6.0 5.5 5.0 4.5 4.0 3.5 3.0 2.5 2.0 1.5 1.0 0.5 0.0
f1 (ppm)

20

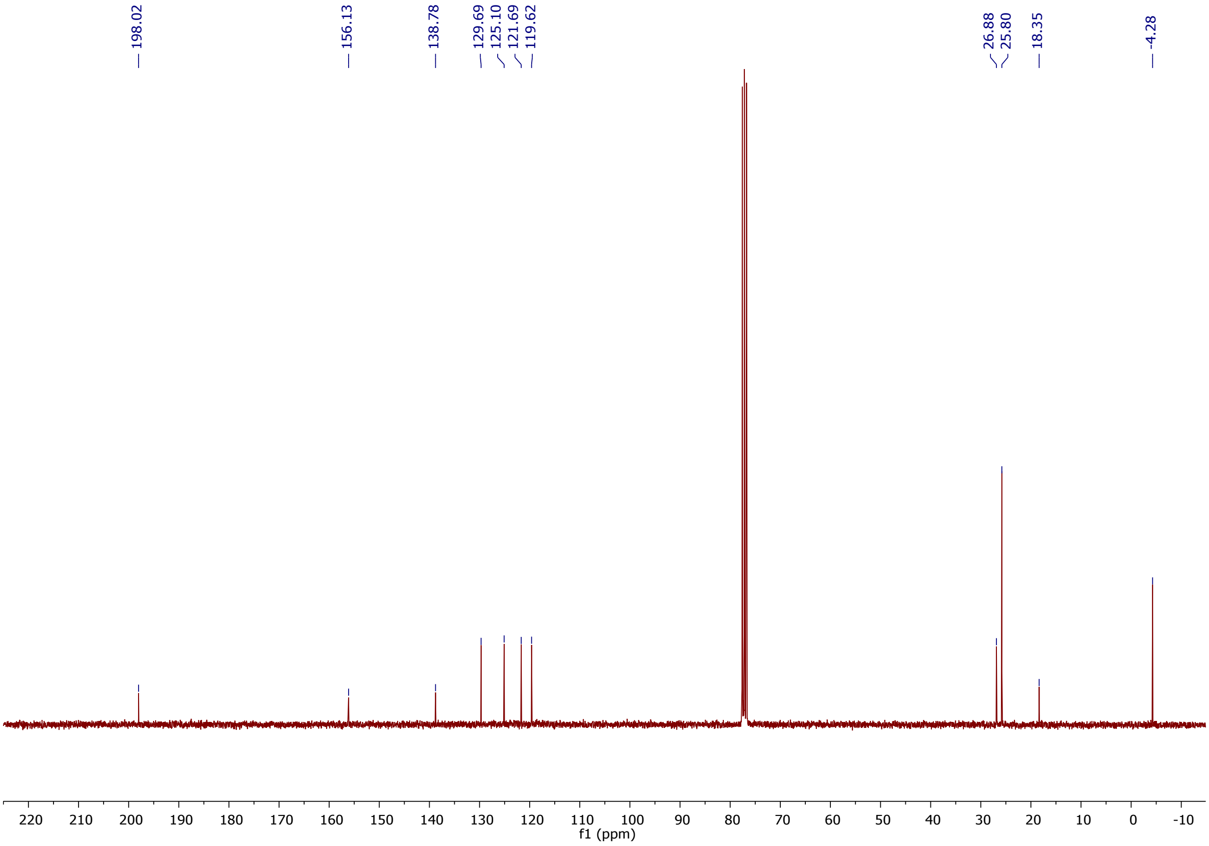
198.02
156.13
138.78
129.69 125.10 121.69 119.62
26.88 25.80
18.35
-4.28
220 210 200 190 180 170 160 150 140 130 120 110 100 90 80 70 60 50 40 30 20 10 0 -10
f1 (ppm)

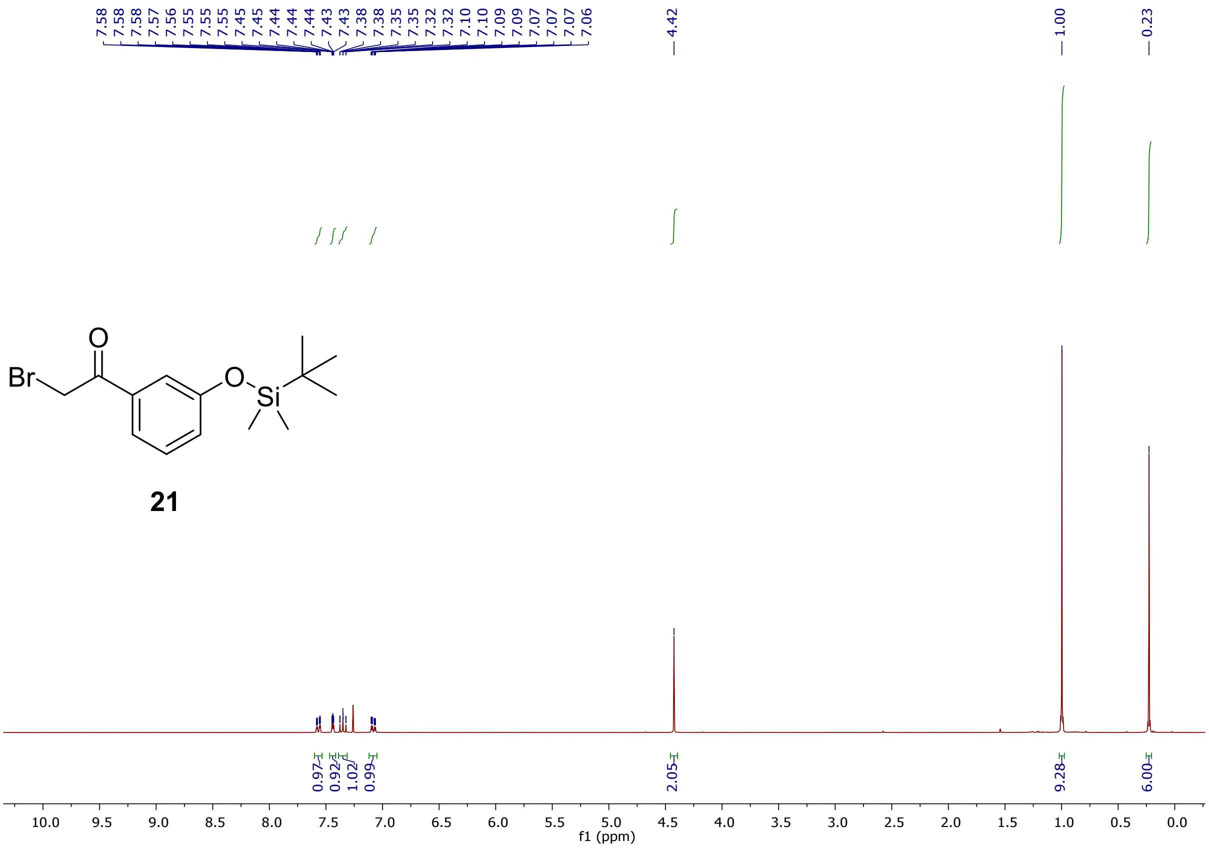
7.58
7.58
7.58
7.57
7.56
7.55
7.55
7.55
7.45
7.45
7.44
7.44
7.43
7.43
7.38
7.38
7.35
7.35
7.32
7.32
7.10
7.10
7.09
7.09
7.07
7.07
7.06
4.42
1.00
0.23
0.97
0.92
1.02
0.99
2.05
9.28
6.00
10.0
9.5
9.0
8.5
8.0
7.5
7.0
6.5
6.0
5.5
5.0
4.5
4.0
3.5
3.0
2.5
2.0
1.5
1.0
0.5
0.0
f1 (ppm)

21

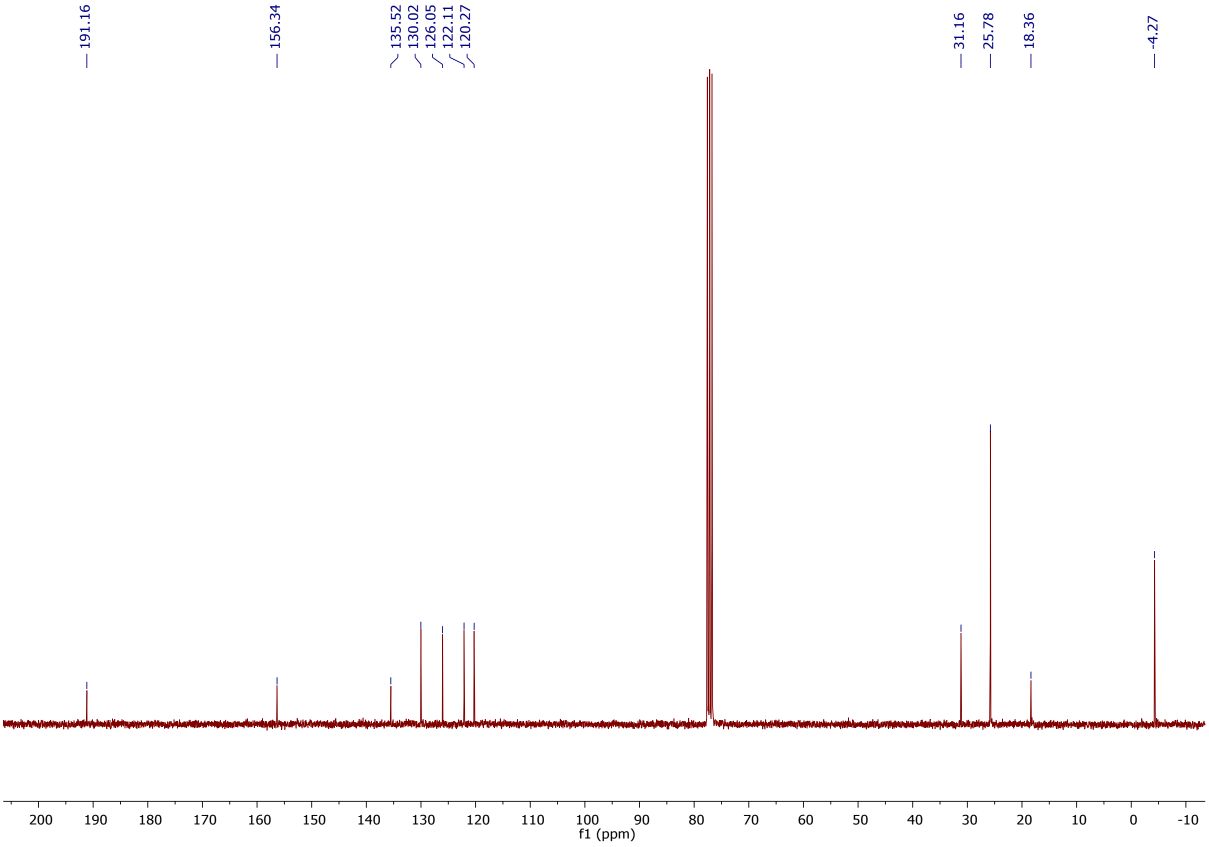
191.16
156.34
135.52
130.02
126.05
122.11
120.27
31.16
25.78
18.36
-4.27
200
190
180
170
160
150
140
130
120
110
100
90
80
70
60
50
40
30
20
10
0
-10
f1 (ppm)

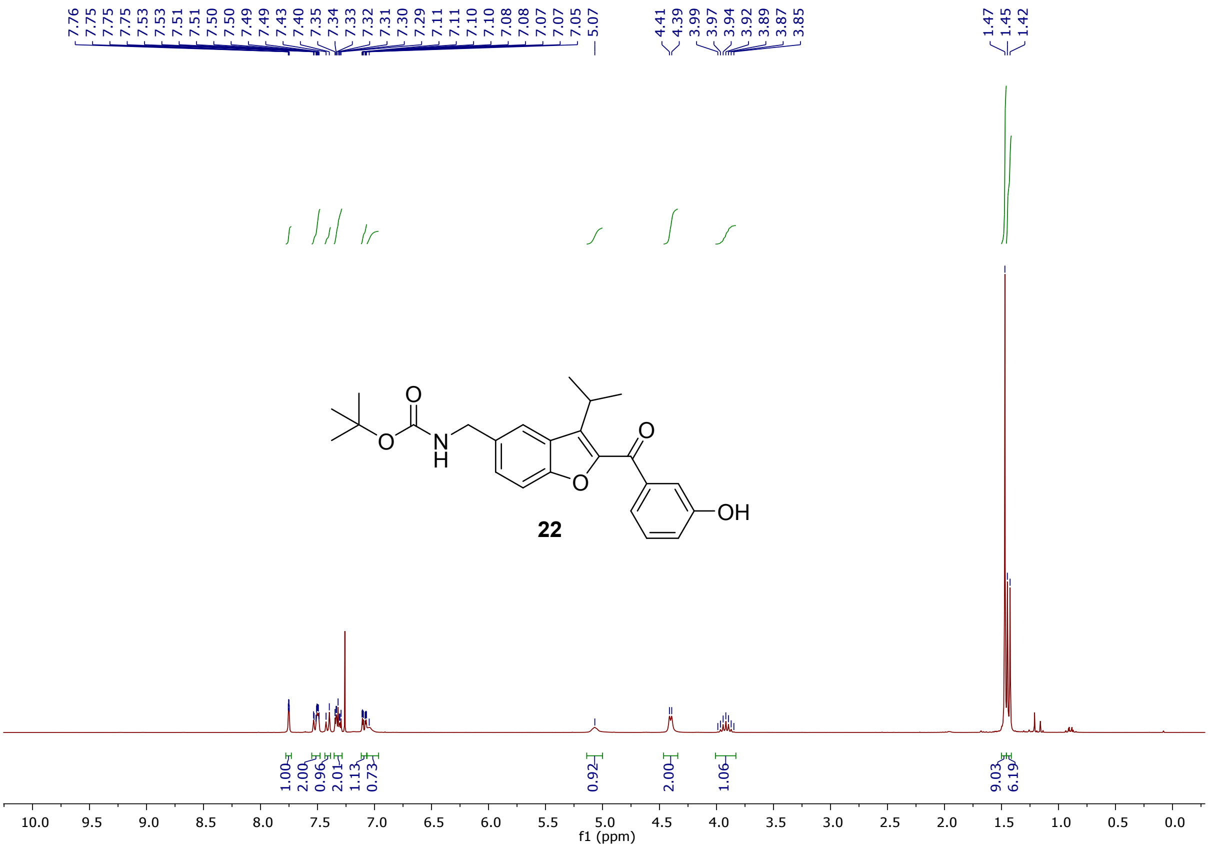
7.76 7.75 7.75 7.53 7.51 7.51 7.50 7.50 7.49 7.49 7.43 7.40 7.35 7.34 7.33 7.32 7.31 7.30 7.29 7.11 7.11 7.10 7.08 7.08 7.07 7.07 7.05 5.07 4.41 4.39 3.99 3.97 3.94 3.92 3.89 3.87 3.85 1.47 1.45 1.42
OH
O
N
H
22
1.00 2.00 0.96 2.01 1.13 0.73 0.92 2.00 1.06 9.03 6.19
f1 (ppm)

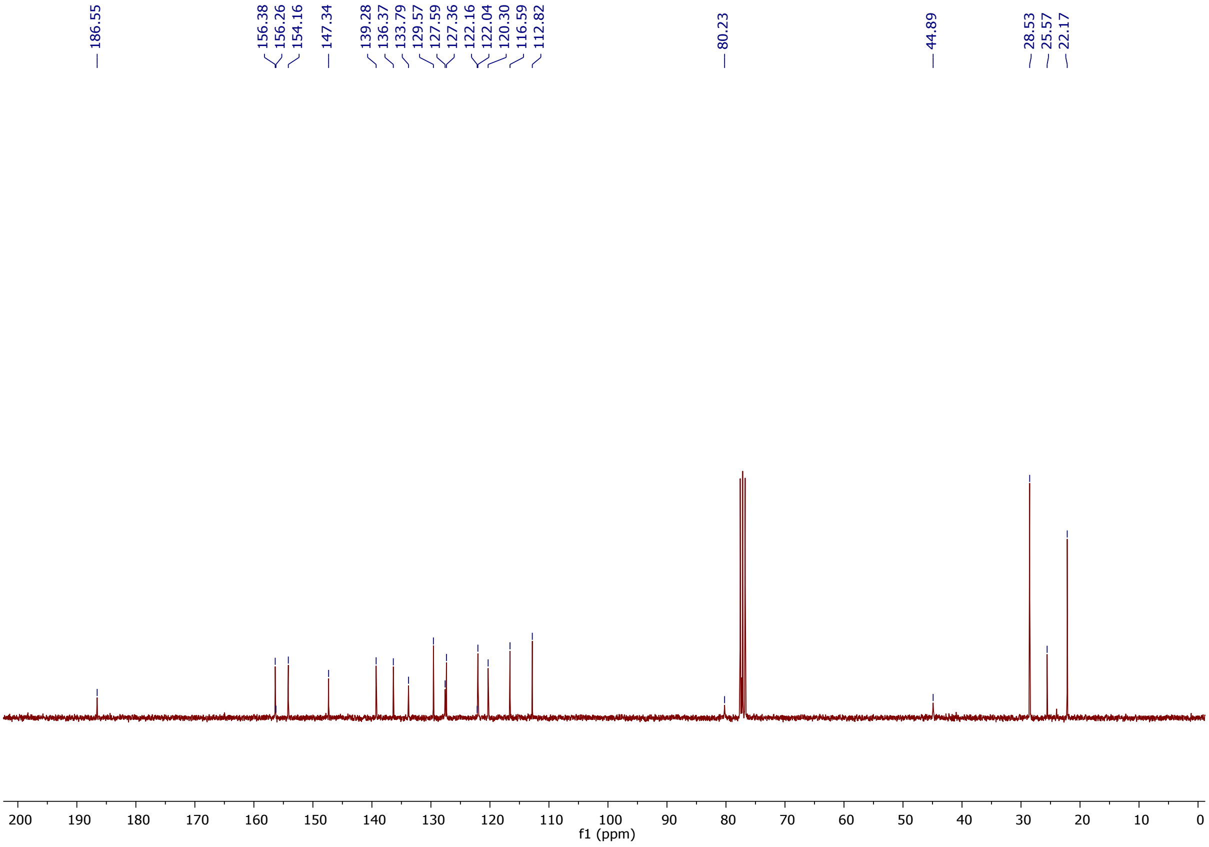
186.55 156.38 156.26 154.16 147.34 139.28 136.37 133.79 129.57 127.59 127.36 122.16 122.04 120.30 116.59 112.82 80.23 44.89 28.53 25.57 22.17
f1 (ppm)

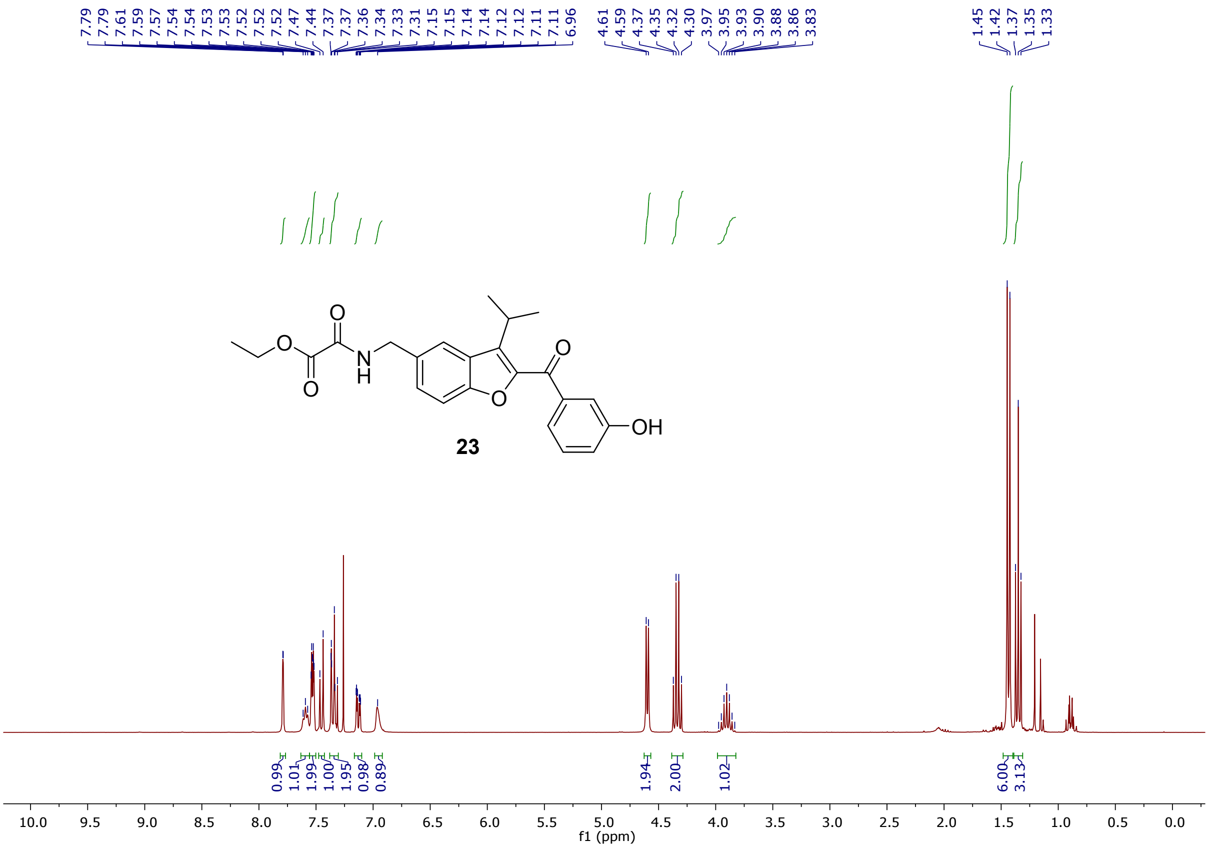

7.79 7.79 7.61 7.59 7.57 7.54 7.54 7.53 7.53 7.52 7.52 7.52 7.47 7.44 7.37 7.37 7.36 7.34 7.33 7.31 7.15 7.15 7.14 7.14 7.12 7.12 7.11 7.11 6.96
4.61 4.59 4.37 4.35 4.32 4.30 3.97 3.95 3.93 3.90 3.88 3.86 3.83
1.45 1.42 1.37 1.35 1.33
O
N
H
O
O
O
O
OH
23
0.99 1.01 1.99 1.00 1.95 0.98 0.89
1.94 2.00 1.02
6.00 3.13
10.0 9.5 9.0 8.5 8.0 7.5 7.0 6.5 6.0 5.5 5.0 4.5 4.0 3.5 3.0 2.5 2.0 1.5 1.0 0.5 0.0
f1 (ppm)

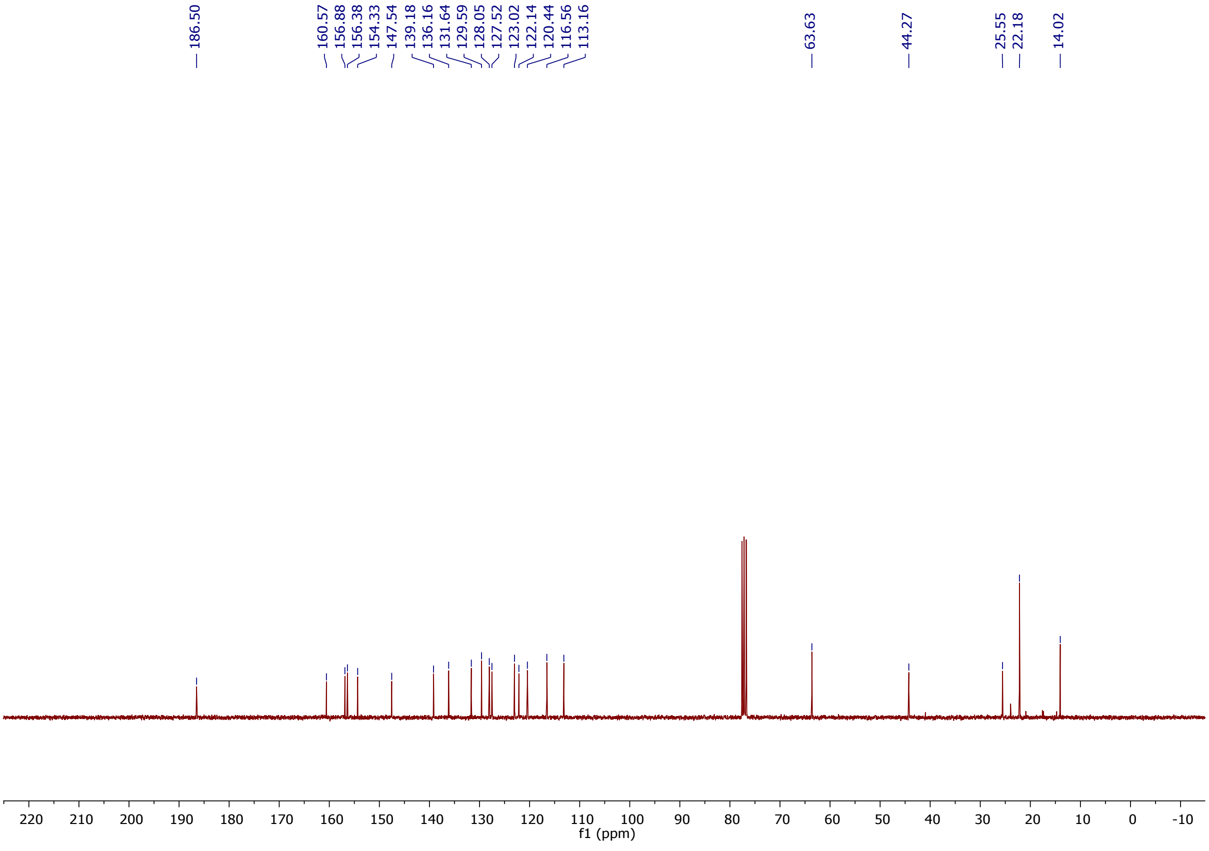

186.50
160.57 156.88 156.38 154.33 147.54 139.18 136.16 131.64 129.59 128.05 127.52 123.02 122.14 120.44 116.56 113.16
63.63
44.27
25.55 22.18
14.02
220 210 200 190 180 170 160 150 140 130 120 110 100 90 80 70 60 50 40 30 20 10 0 -10
f1 (ppm)

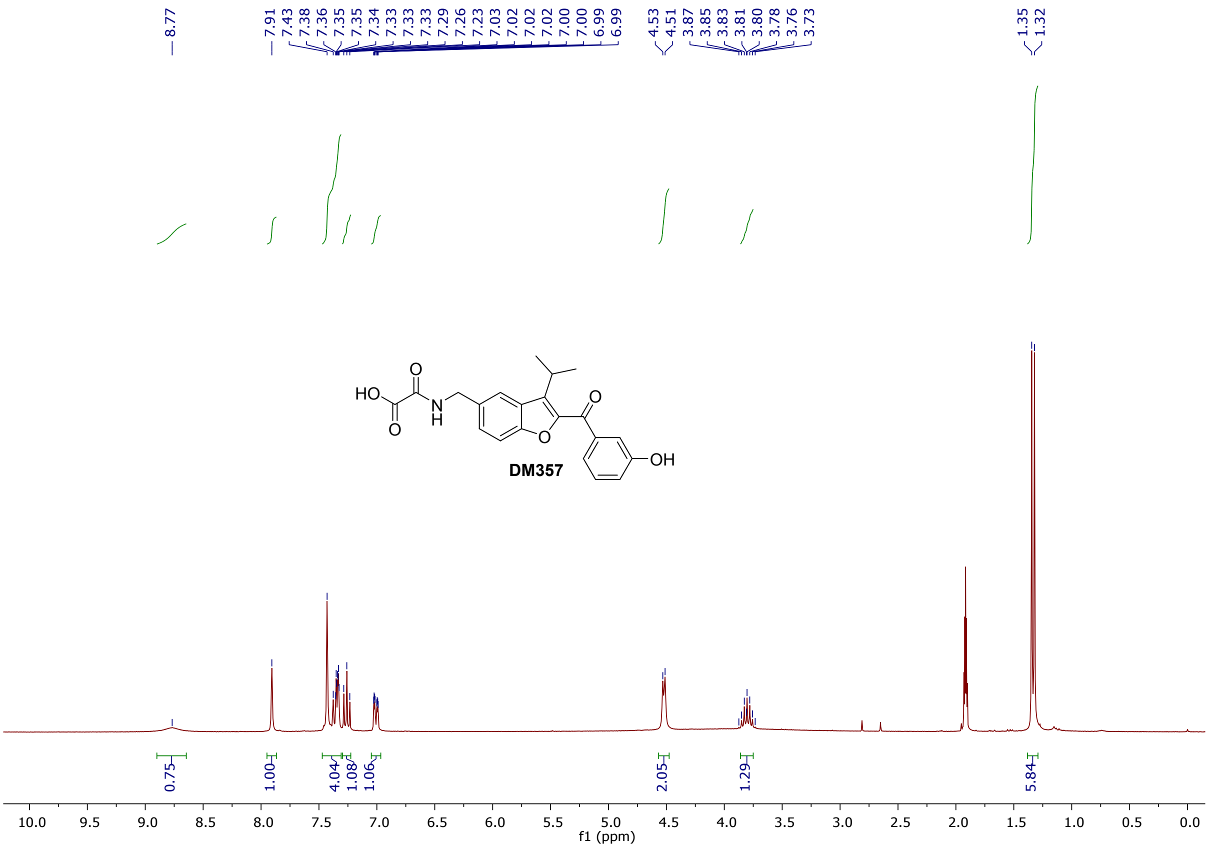
8.77
7.91
7.43
7.38
7.36
7.35
7.35
7.34
7.33
7.33
7.33
7.29
7.26
7.23
7.03
7.02
7.02
7.02
7.00
7.00
6.99
6.99
4.53
4.51
3.87
3.85
3.83
3.81
3.80
3.78
3.76
3.73
1.35
1.32
HO
O
N
H
O
O
O
OH
DM357
0.75
1.00
4.04
1.08
1.06
2.05
1.29
5.84
f1 (ppm)

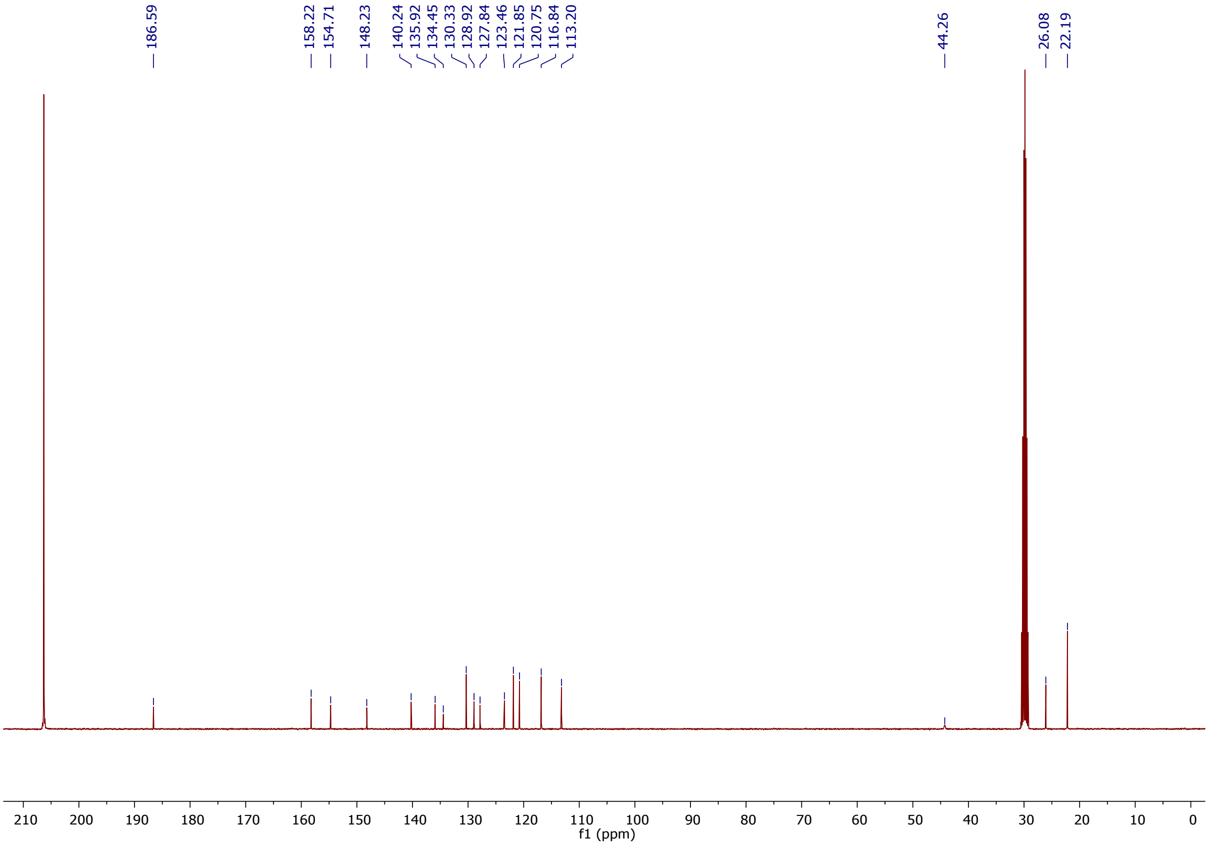
186.59
158.22
154.71
148.23
140.24
135.92
134.45
130.33
128.92
127.84
123.46
121.85
120.75
116.84
113.20
44.26
26.08
22.19
f1 (ppm)

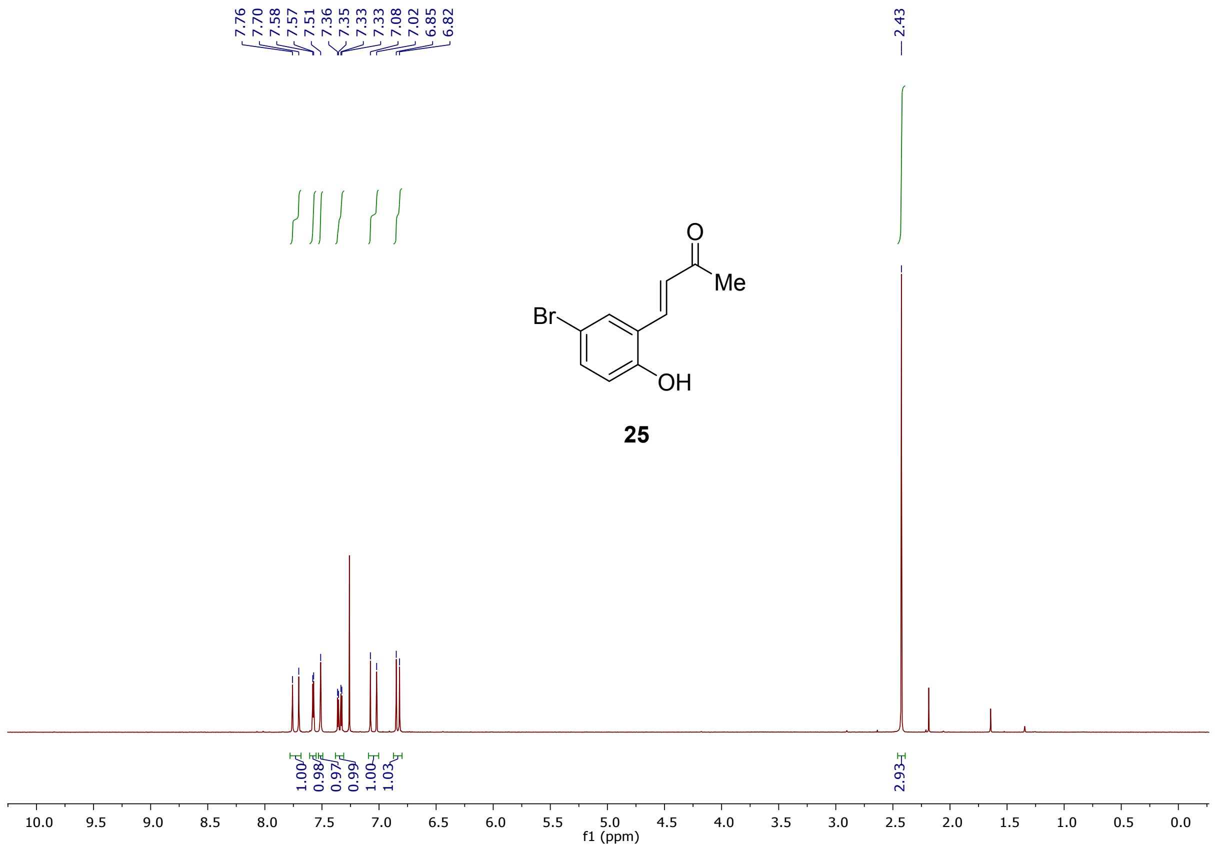
7.76
7.70
7.58
7.57
7.51
7.36
7.35
7.33
7.33
7.08
7.02
6.85
6.82
2.43
O
Me
Br
OH
25
1.00
0.98
0.97
0.99
1.00
1.03
2.93
f1 (ppm)

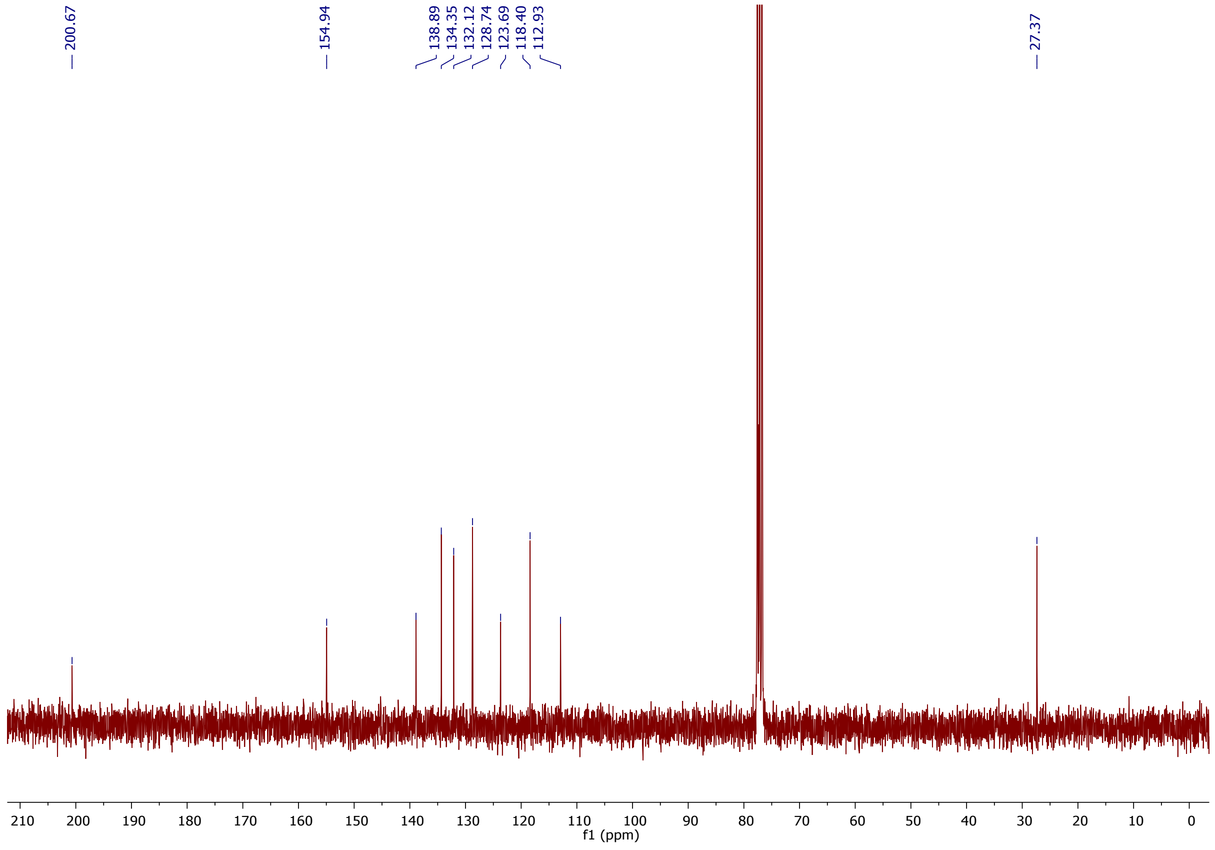
200.67
154.94
138.89
134.35
132.12
128.74
123.69
118.40
112.93
27.37
f1 (ppm)

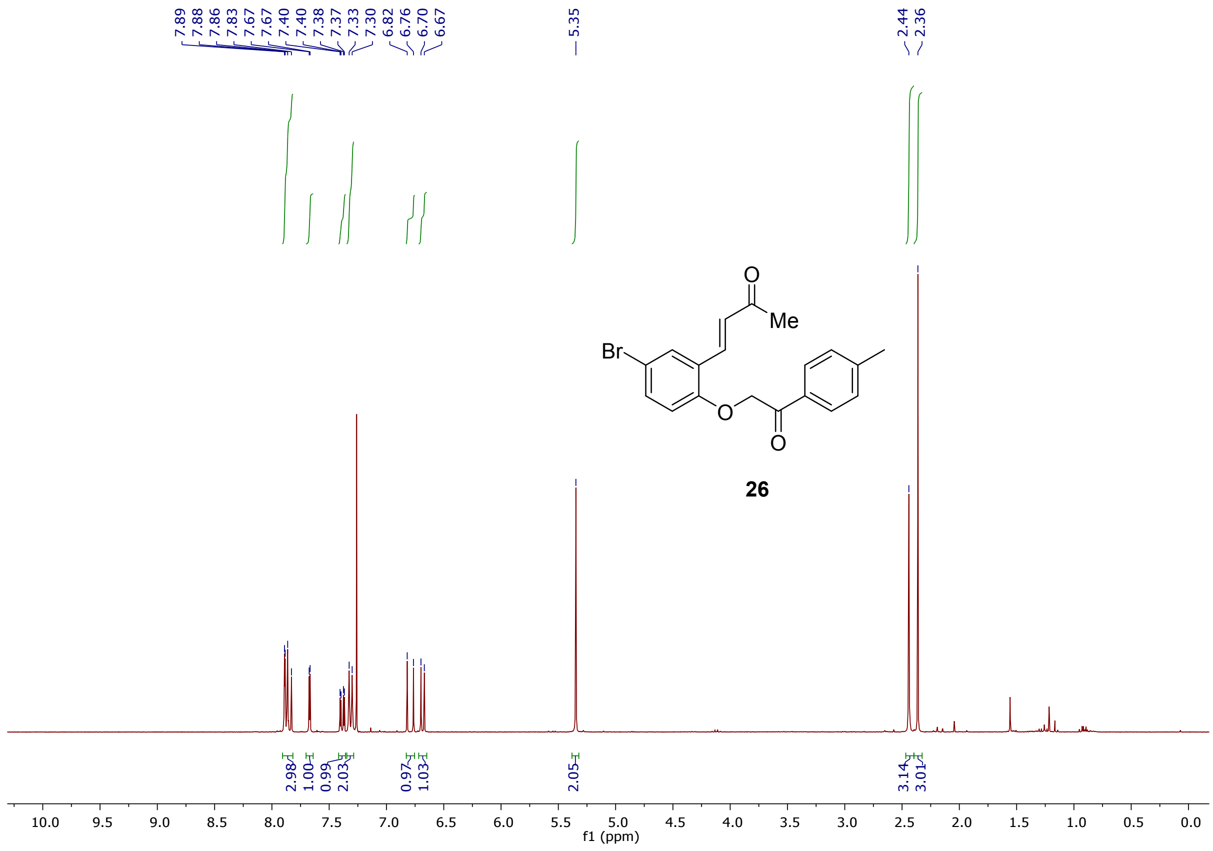
7.89
7.88
7.86
7.83
7.67
7.67
7.40
7.40
7.38
7.37
7.33
7.30
6.82
6.76
6.70
6.67
5.35
2.44
2.36
O
Me
Br
O
O
26
2.98
1.00
0.99
2.03
0.97
1.03
2.05
3.14
3.01
10.0
9.5
9.0
8.5
8.0
7.5
7.0
6.5
6.0
5.5
5.0
4.5
4.0
3.5
3.0
2.5
2.0
1.5
1.0
0.5
0.0
f1 (ppm)

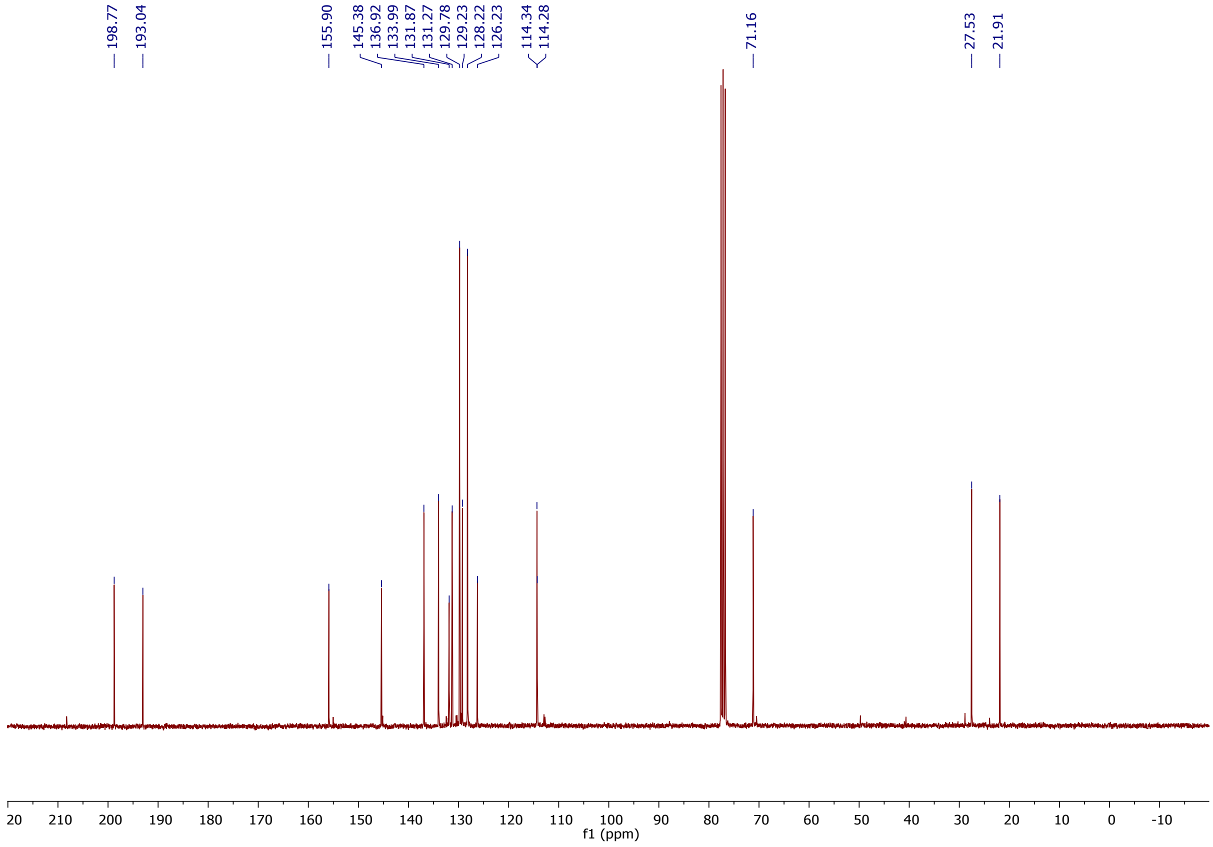
198.77
193.04
155.90
145.38
136.92
133.99
131.87
131.27
129.78
129.23
128.22
126.23
114.34
114.28
71.16
27.53
21.91
20
210
200
190
180
170
160
150
140
130
120
110
100
90
80
70
60
50
40
30
20
10
0
-10
f1 (ppm)

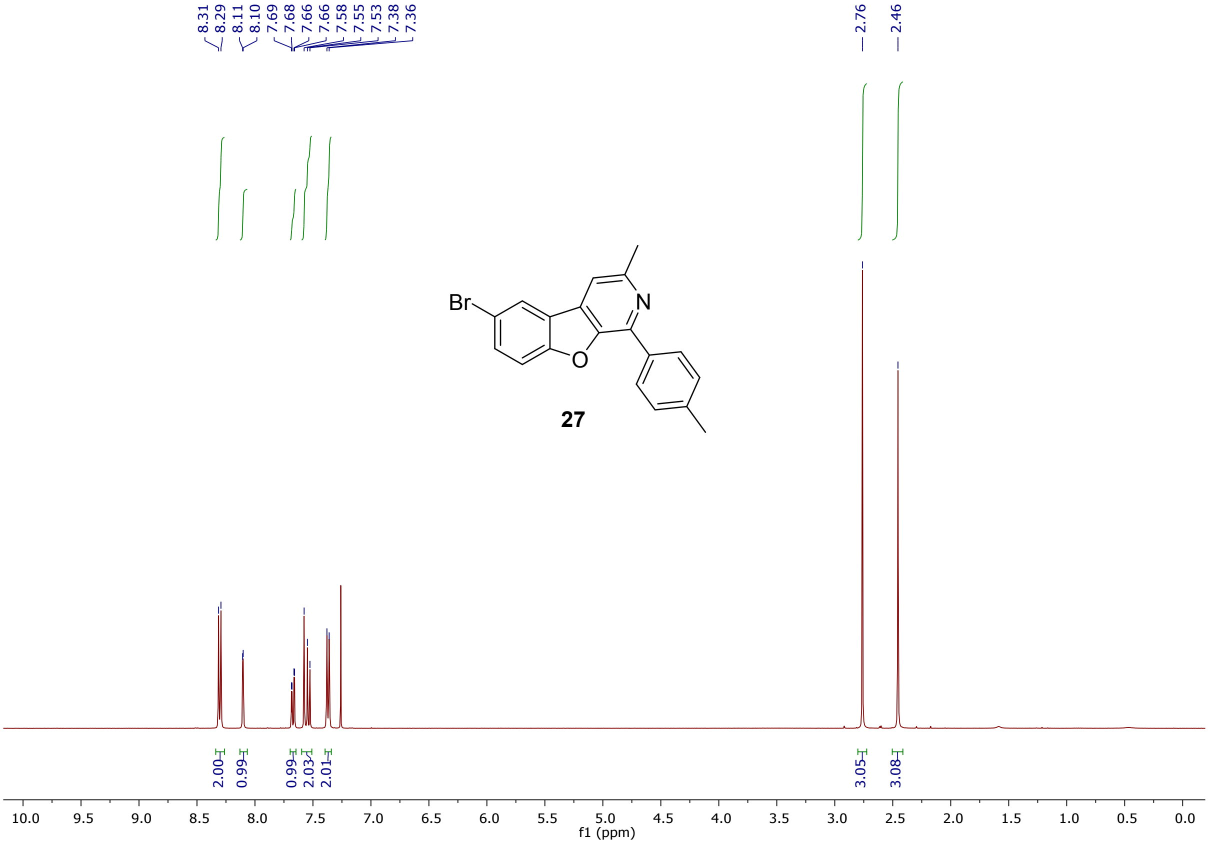
8.31
8.29
8.11
8.10
7.69
7.68
7.66
7.66
7.58
7.55
7.53
7.38
7.36
2.76
2.46
Br
N
O
27
2.00
0.99
0.99
2.03
2.01
3.05
3.08
10.0 9.5 9.0 8.5 8.0 7.5 7.0 6.5 6.0 5.5 5.0 4.5 4.0 3.5 3.0 2.5 2.0 1.5 1.0 0.5 0.0
f1 (ppm)

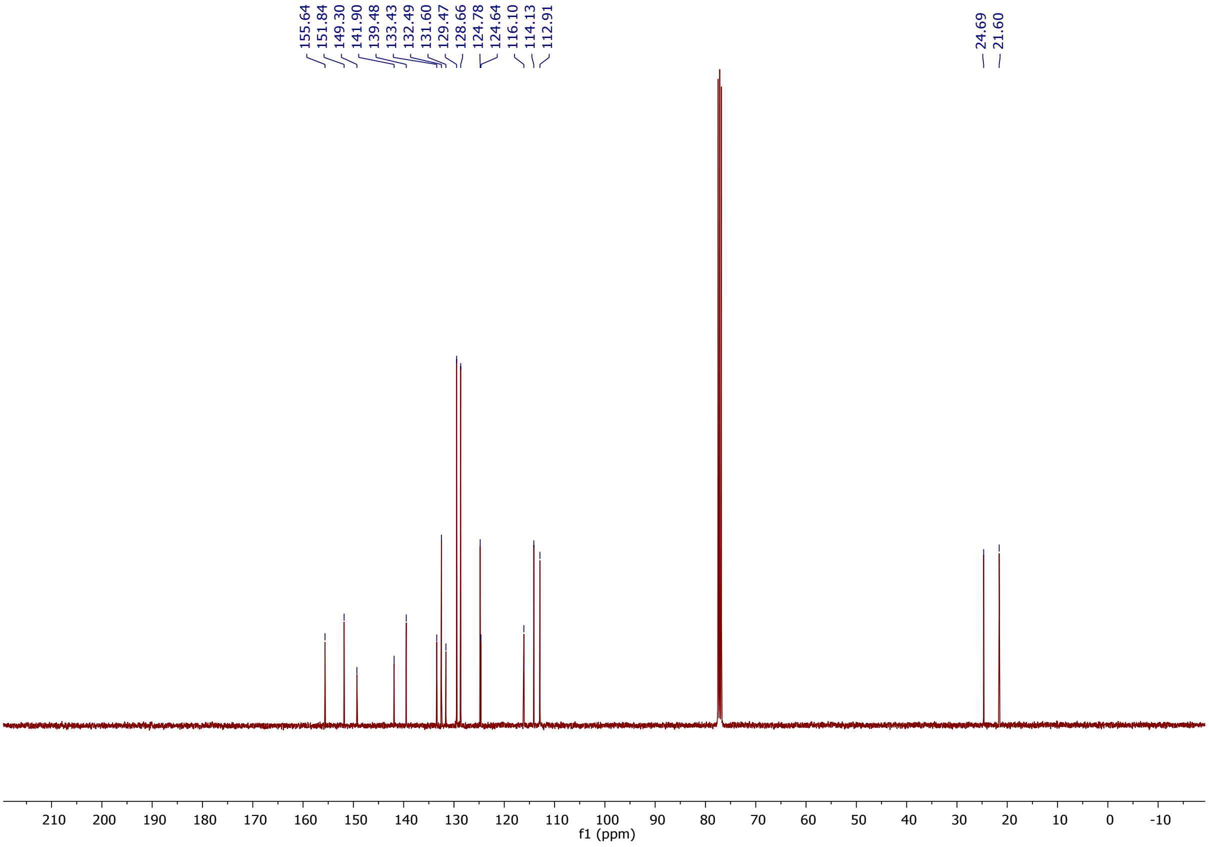
155.64
151.84
149.30
141.90
139.48
133.43
132.49
131.60
129.47
128.66
124.78
124.64
116.10
114.13
112.91
24.69
21.60
210 200 190 180 170 160 150 140 130 120 110 100 90 80 70 60 50 40 30 20 10 0 -10
f1 (ppm)

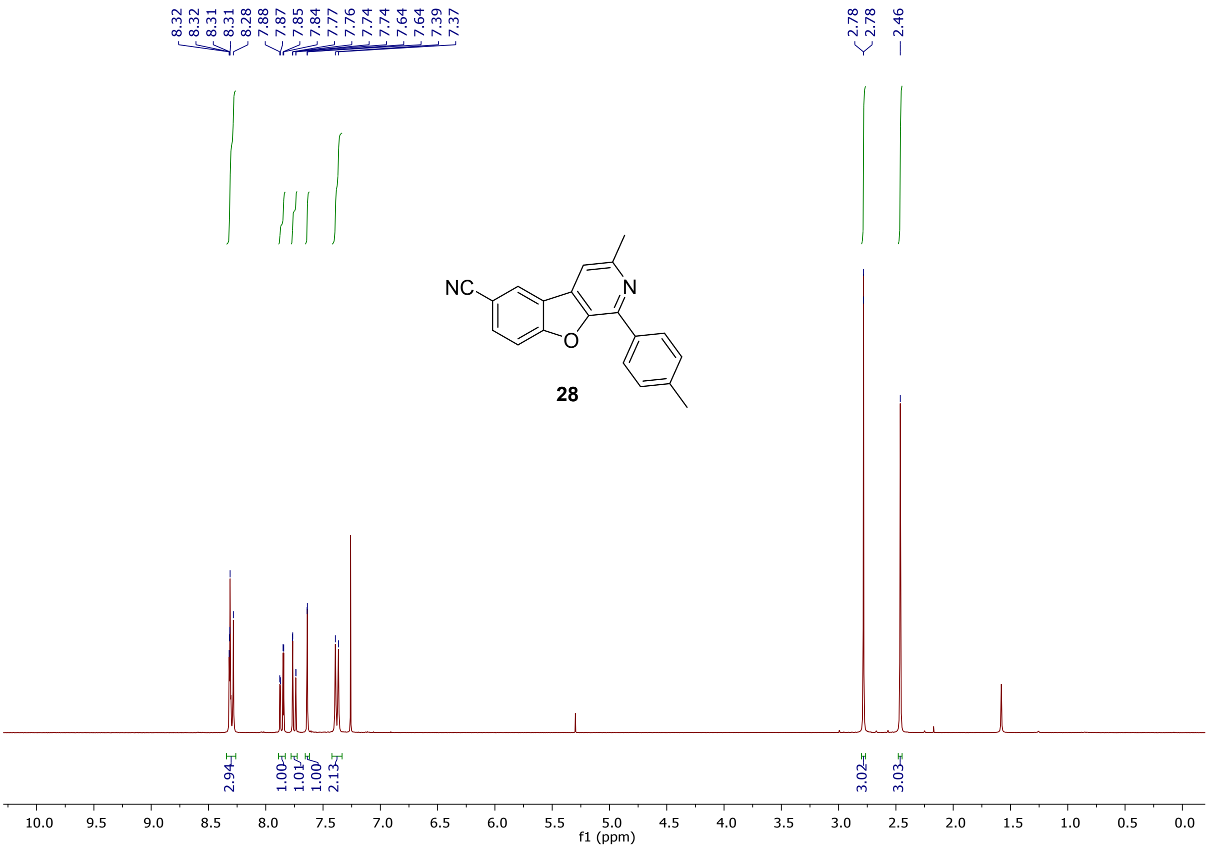
8.32
8.32
8.31
8.31
8.28
7.88
7.87
7.85
7.84
7.77
7.76
7.74
7.74
7.64
7.64
7.39
7.37
2.78
2.78
2.46
NC
N
O
28
2.94
1.00
1.01
1.00
2.13
3.02
3.03
10.0
9.5
9.0
8.5
8.0
7.5
7.0
6.5
6.0
5.5
5.0
4.5
4.0
3.5
3.0
2.5
2.0
1.5
1.0
0.5
0.0
f1 (ppm)

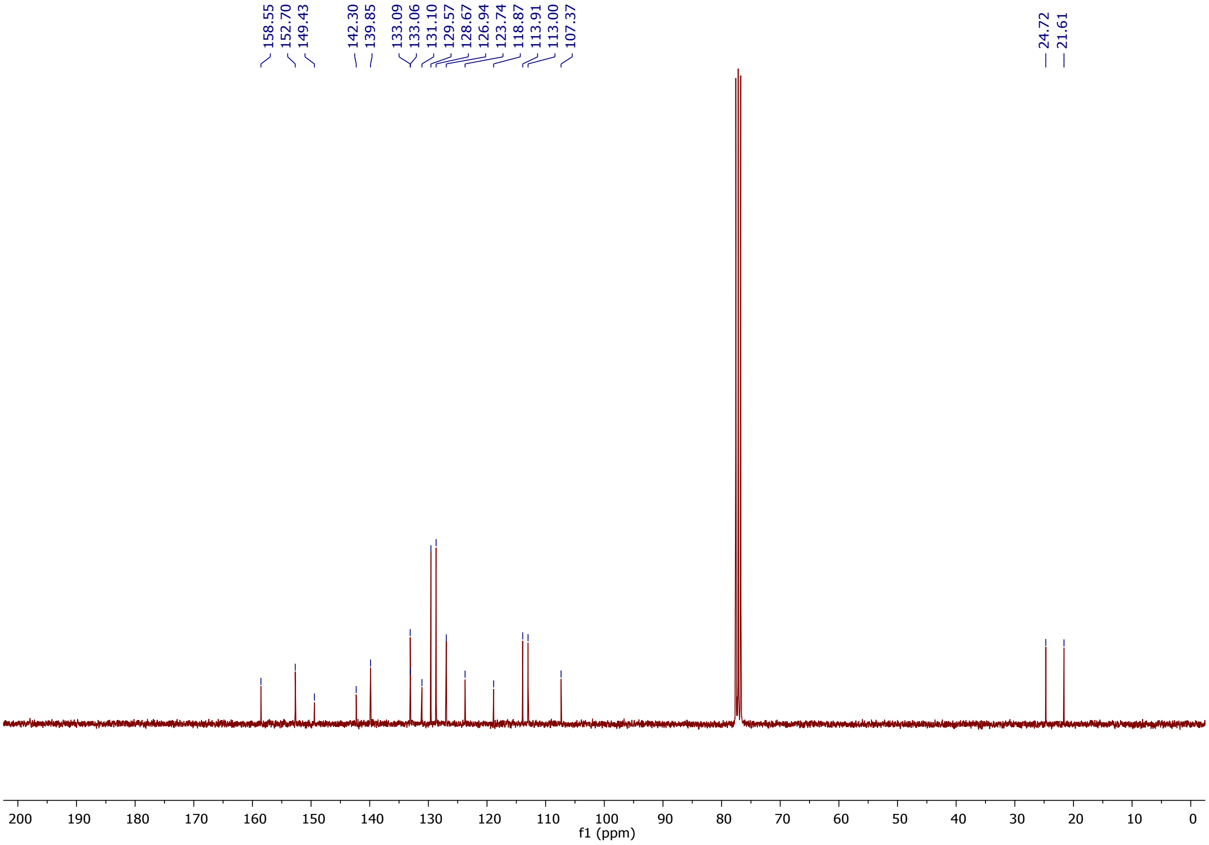
158.55
152.70
149.43
142.30
139.85
133.09
133.06
131.10
129.57
128.67
126.94
123.74
118.87
113.91
113.00
107.37
24.72
21.61
200
190
180
170
160
150
140
130
120
110
100
90
80
70
60
50
40
30
20
10
0
f1 (ppm)

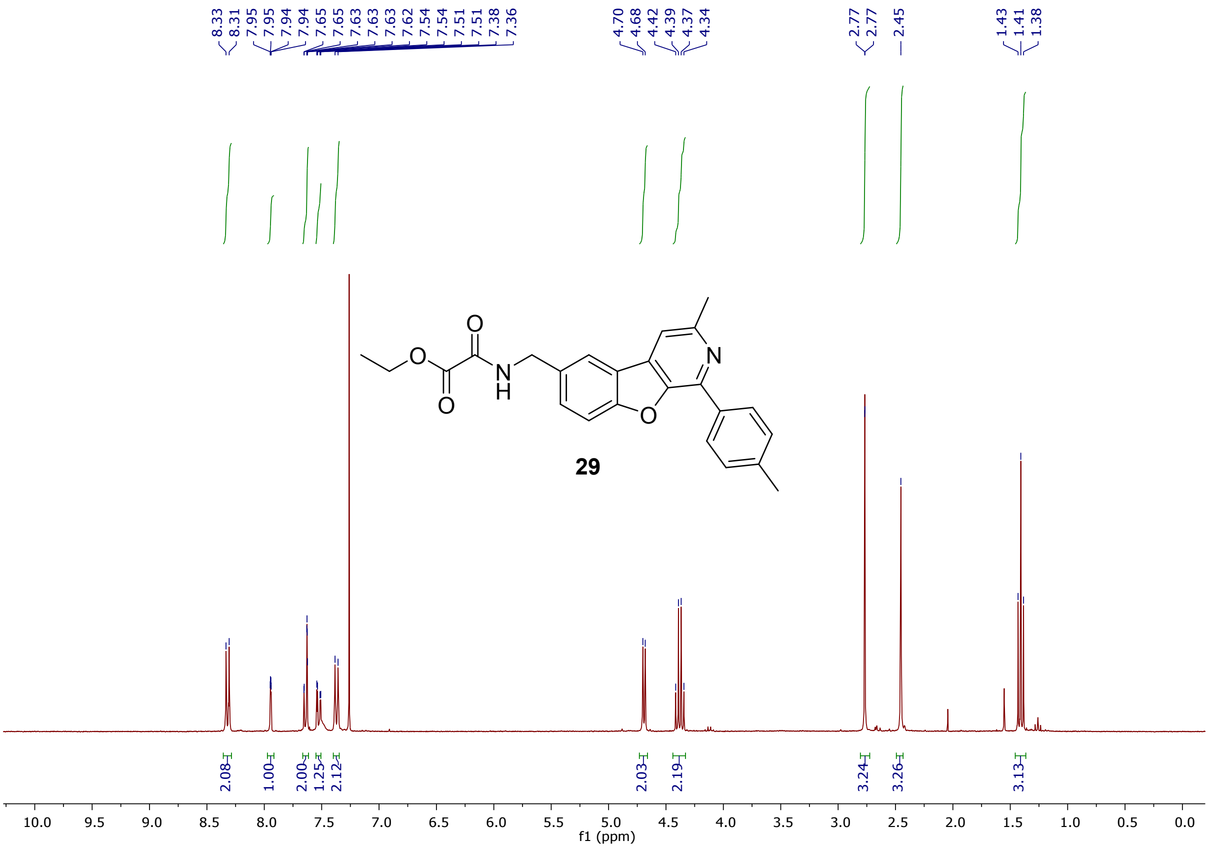

8.33
8.31
7.95
7.95
7.94
7.65
7.65
7.63
7.63
7.63
7.62
7.54
7.54
7.51
7.51
7.38
7.36
4.70
4.68
4.42
4.39
4.37
4.34
2.77
2.77
2.45
1.43
1.41
1.38
O
O
O
N
H
N
O
29
2.08
1.00
2.00
1.25
2.12
2.03
2.19
3.24
3.26
3.13
10.0
9.5
9.0
8.5
8.0
7.5
7.0
6.5
6.0
5.5
5.0
4.5
4.0
3.5
3.0
2.5
2.0
1.5
1.0
0.5
0.0
f1 (ppm)

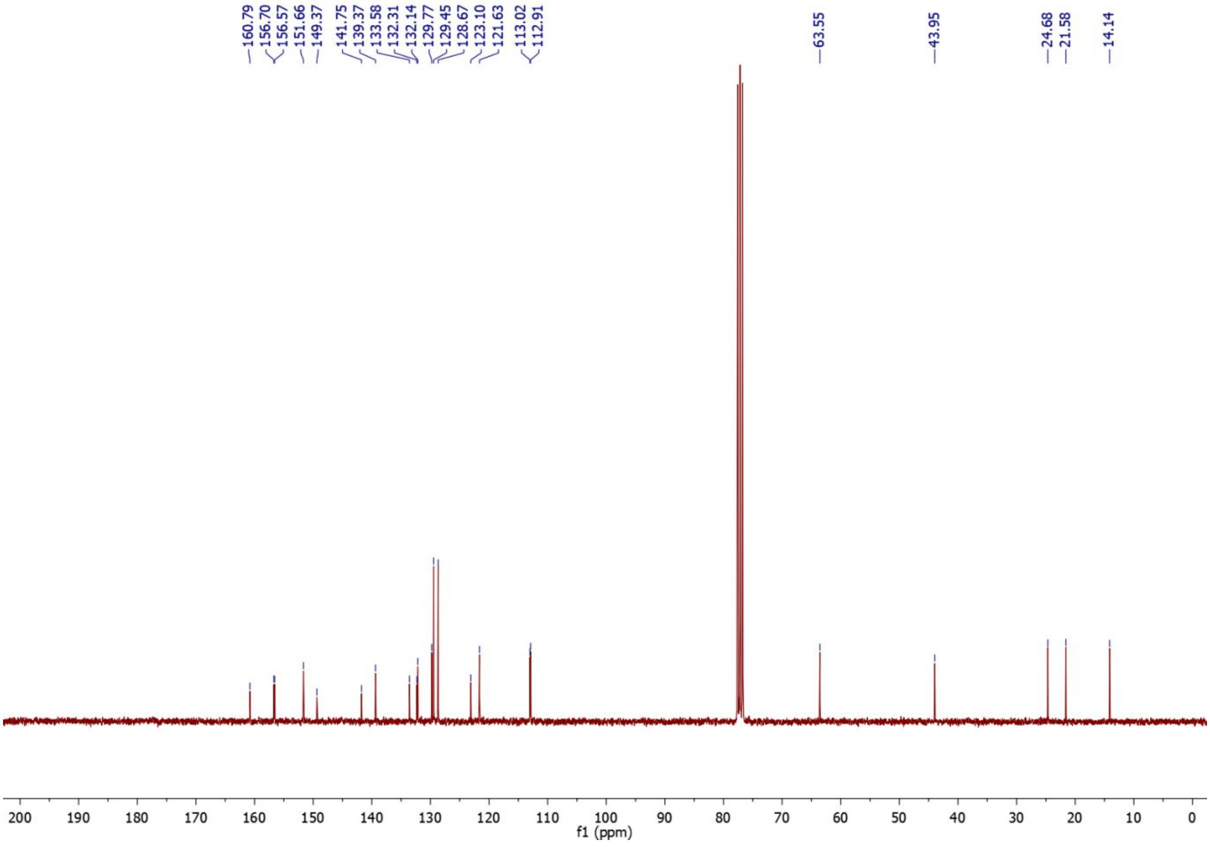

160.79
156.70
156.57
151.66
149.37
141.75
139.37
133.58
132.31
132.14
129.77
129.45
128.67
123.10
121.63
113.02
112.91
63.55
43.95
24.68
21.58
14.14
200
190
180
170
160
150
140
130
120
110
100
90
80
70
60
50
40
30
20
10
0
f1 (ppm)

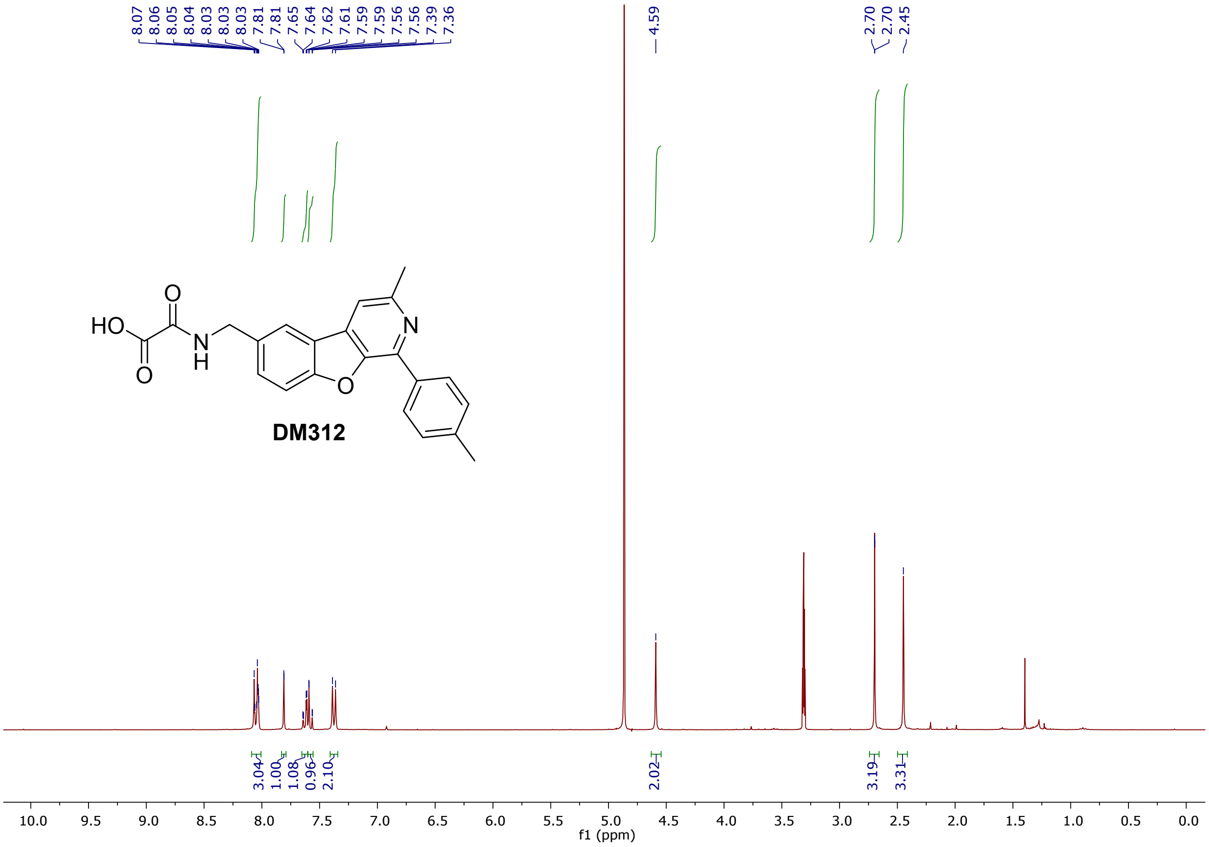
8.07 8.06 8.05 8.04 8.03 8.03 8.03 7.81 7.81 7.65 7.64 7.62 7.61 7.59 7.59 7.56 7.56 7.39 7.36
4.59
2.70 2.70 2.45
HO
O
N
H
O
N
O
DM312
3.04 1.00 1.08 0.96 2.10
2.02
3.19 3.31
10.0 9.5 9.0 8.5 8.0 7.5 7.0 6.5 6.0 5.5 5.0 4.5 4.0 3.5 3.0 2.5 2.0 1.5 1.0 0.5 0.0
f1 (ppm)

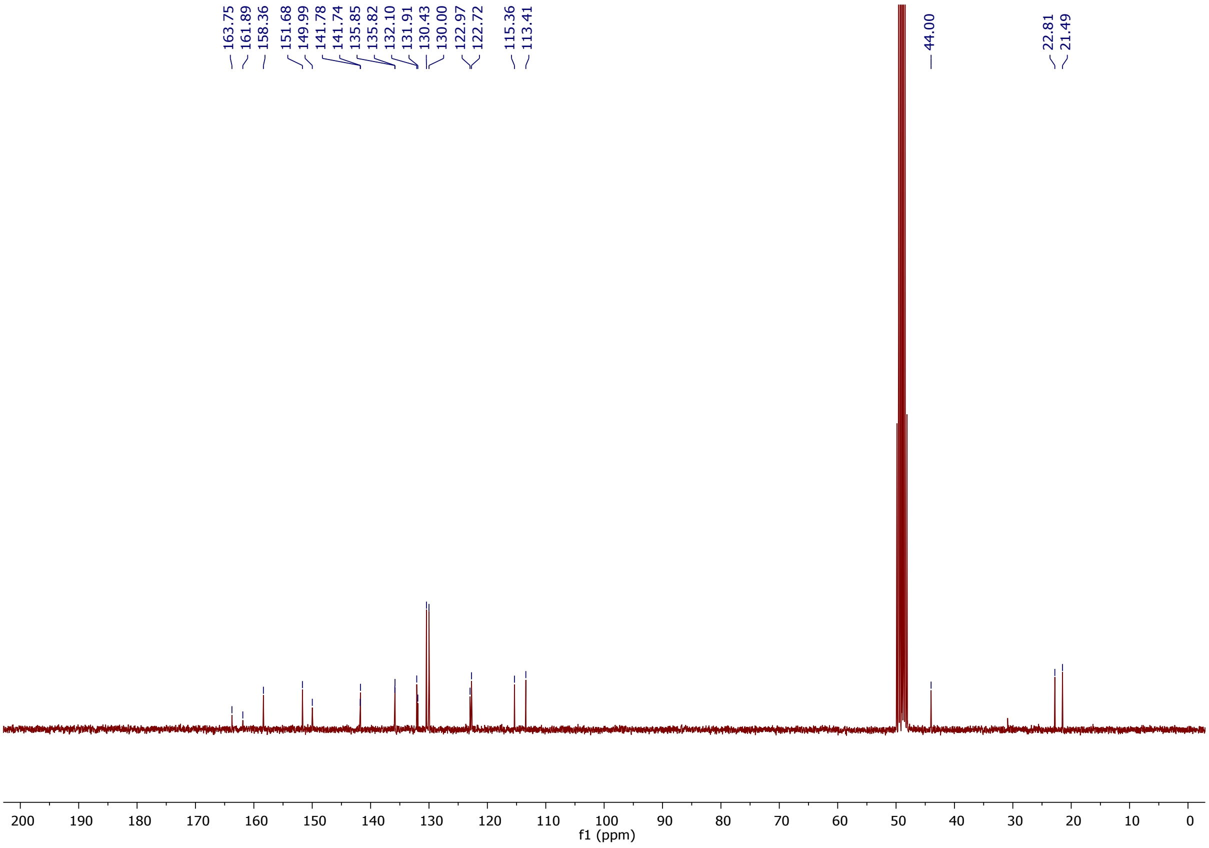
163.75 161.89 158.36 151.68 149.99 141.78 141.74 135.85 135.82 132.10 131.91 130.43 130.00 122.97 122.72 115.36 113.41
44.00
22.81 21.49
200 190 180 170 160 150 140 130 120 110 100 90 80 70 60 50 40 30 20 10 0
f1 (ppm)
